# Identification and gene expression analysis of serine proteases and their homologs in the Asian corn borer *Ostrinia furnacalis*

**DOI:** 10.1038/s41598-023-31830-2

**Published:** 2023-03-23

**Authors:** Lei Yang, Xiaoli Xu, Wei wei, Xiaoyun Chen, Cheng Peng, Xiaofu Wang, Junfeng Xu

**Affiliations:** State Key Laboratory for Managing Biotic and Chemical Threats to the Quality and Safety of Agro-products, Zhejiang Academy of Agricultural Sciences, Hangzhou, 310021 China

**Keywords:** Biochemistry, Computational biology and bioinformatics, Immunology, Molecular biology

## Abstract

Serine proteases (SPs) and their homologs (SPHs) are among the best-characterized gene families. They are involved in several physiological processes, including digestion, embryonic development and immunity. In the current study, a total of 177 SPs-related genes were characterized in the genome of *Ostrinia furnacalis*. The activation site of SPs/SPHs and enzyme specificity of SPs were identified, and the findings showed that most of the SPs analyzed possessed trypsin substrate specificity. Several SPs/SPHs with similar simple gene structures had tandem repeat-like distributions on the scaffold, indicated that gene expansion has occurred in this large family. Furthermore, we constructed 30 RNA sequencing libraries including four with developmental stage and four middle larval stage tissues to study the transcript levels of these genes. Differentially upregulated and downregulated genes were obtained via data analysis. More than one-quarter of the genes were specifically identified as highly expressed in the midgut in compared to the other three tissues evaluated. In the current study, the domain structure, gene location and phylogenetic relationship of genes in *O. furnacalis* were explored. Orthologous comparisons of SPs/SPHs between model insects and *O. furnacalis* indicated their possible functions. This information provides a basis for understanding the functional roles of this large family.

## Introduction

Chymotrypsin-like serine proteases (SPs) and their homologs constitute a large gene family that plays essential roles in various insect physiological processes. SP-related peptides in inactive zymogen form are converted into their active forms by proteolytic cleavage of a particular peptide bond^1,2^. A specific catalytic triad comprising His57, Ser195, and Asp102 amino acid residues is harbored in the active center of SPs. Amino acid residues 189, 216 and 226 in chymotrypsin near the active site form the primary substrate-binding pocket, which is the predominant factor for determining SP substrate specificity^3,4^. This specific molecular recognition ability enables SPs to activate downstream enzymes, amplify signals, and form a cascade pathway. In addition to SPs, some members are enzymatically inactive owing to mutation of one or more catalytic residues. These proteins are known as serine protease homologs (SPHs) and are predicted to be cofactors of SPs. The roles played by SPs and SPHs have been extensively studied in invertebrates and found to range from the digestion of ingested proteins to immune defenses directed against various microbes and the establishment of embryo dorsoventral polarity^5–14^. SPs dominate the larval gut environment and contribute to approximately 95% of total digestive activity^5^. The functions of SPs and SPHs involved in epithelial morphogenesis of imaginal discs^6,7^ and somatic muscle attachment in embryos^8,9^ have been illustrated in *Drosophila*. The immune responses induced by SPs and SPHs have been reported in insect species^10–14^ and include melanotic encapsulation and induction of antimicrobial peptides.

Most SPs comprise fewer than 300 residues and harbor a single serine protease domain. These enzymes are mainly found in the gut and are involved in food digestion^5^. In addition, some SPs have disulfide-stabilized structures at the N-terminus; this structure is known as the clip domain and is formed by six conserved cycteine residues^15,16^. SPs and SPHs with clip domains are called CLIPs^17^. The roles played by CLIPs in the extracellular SP cascade, in which one SP activates the zymogen of another SP to trigger a rapid response, have been extensively explored in several insects^18^. In tobacco hornworm, HP6 activates HP8 to convert proSpatzle into Spatzle, thus inducing the synthesis of antimicrobial peptides (AMPs). Autoactivated HP14 activates proHP21, which further activates proPAP2 and PAP3 to induce proPO activation cascade. SPH1 and SPH2 are essential to this cascade, as they accelerate the production of high Mr active PO^10–12^. *Holotrichia diomphalia* PPAF-II and *Tenebrio molitor* SPH1 are involved in the sequential activation of proPOs to produce a melanization complex^13,14^. Some SPs present with other structural characteristics, such as a low-density lipoprotein receptor class A (LDLA) domain, Frizzled (Fz) domain, complement control protein (CCP) domain, or gastrulation defective (Gd) domain. These additional domains enable SPs to interact with other enzymes and thus participate in additional processes. For instance, *Manduca sexta* HP14 and *Drosophila melanogaster* modular serine protease (ModSP) have repeated LDLAs and CCP domain and, when initiated, receive signals from recognition receptors and activate proPAP in Toll pathway^11,19^. Limulus clotting factor C found in crayfish and horseshoe crab can form insoluble coagulin and plays a role in crosslinking^20^. Multidomain SPs, Nudel and Gd are involved in dorsal–ventral axis establishment^21,22^.

Advances in sequencing technology have increased the availability of genome data from different organisms. The number of SP-related genes in *D. melanogaster*, *Apis mellifera*, *Bombyx mori*, *M. sexta*, *Anopheles gambiae* and *Pteromalus puparum* has been identified at the genome level^4,23–27^. Sequencing technology has accelerated the identification of SP genes, in addition to the cloning and classification of single SPs. Orthologous comparison of these genes has enabled the prediction of the functional roles of newly discovered SPs/SPHs.

The Asian corn borer, *O. furnacalis* (Guenée), is an agricultural pest in Asia that causes significant damage in corn-producing countries with 20–80% yield losses^28^. The Asian corn borer has developed resistance to chemical and biotic pesticides, even in Bt crops^28–30^ making pest population control challenging. A previous study identified 13 CLIPs through transcriptome analysis^31^. The roles of four *O. furnacalis* SPs involved in the melanization pathway have been verified through biological experiments^32–34^. Although some SPs have been explored previously, the roles played by most SPs have not been elucidated. The roles of *O. furnacalis* SPs in digestion, development and immunity have not been fully elucidated. The *O. furnacalis* genome is currently available; thus, SP and SPH genes from *O. furnacalis* were characterized in the present study. Systematic analyses were conducted by determining catalytic characteristics, gene structure, and scaffold information; construction of evolution tree; and identifying expression patterns. This information enabled us to predict possible biological functions of *O. furnacalis* SP genes and provides a basis for further research.

## Results

### Overview of *O. furnacalis* SP and SPH sequences

Before the characterization of SP/SPH genes, we evaluated the completeness of the *O. furnacalis* genome by calculating the coverage for the set of single-copy orthologous genes in Arthropoda and Lepidoptera using BUSCO; the results showed genome coverage rates of 96.8% (981/1013) and 97.2% (5137/5286), respectively (Supplementary Figure S1). We also identified 971 and 5073 conserved single-copy Arthropoda and Lepidoptera genes in a BUSCO analysis of protein sequences, with 95.9% and 96.0% sequence similarity, respectively (Supplementary Figure S1).

Genomic evaluation of *O. furnacalis* indicated a total of 177 SP and SPH genes (Supplementary Table S1). The amino acid sequences of these SPs/SPHs are presented in Supplementary file in FASTA format. Twenty-eight SP/SPH genes were expressed as 2 to 4 isoforms, which were identified on the basis of structural gene annotation. The total number of SP-related genes was similar to that in *Tribolium castaneum* (175), and was significantly fewer than that in *D. melanogaster* (257) or *A. gambiae* (337) (Table 1). SPs and SPHs are synthesized as zymogens^1^. The amino acid at the P1 position of the activation site determines the enzyme specificity for SP/SPH catalysis. SPs/SPHs with Arg or Lys residue at P1 position are cleaved by trypsin-like proteases; SPs/SPHs containing Phe/Tyr/Leu residues at this position are substrates of chymotrypsin-like proteases, whereas SPs/SPHs with Ala/Gly/Val/Ile/Met/Ser residues at this position are activated by elastase-like proteases^26^. An analysis showed that 114 of 177 SPs/SPHs were activated by trypsin-like proteases, whereas 16 and 21 were presumably activated by chymotrypsin-like and elastase-like proteases, respectively. The residue at the P1 position was replaced by other amino acids in 21 SPs/SPHs, implying that they have unique features. The remaining 5 genes lacked residue at P1 position (Supplementary Table S1).

**Table 1:** Gene counts for clip-domain and non-clip-domain SPs/SPHs.

	cSP	SP	cSPH	SPH	Total
*Drosophila melanogaster*	34	156	18	49	257
*Apis mellifera*	14	32	7	6	59
*Tribolium castaneum*	34	71	20	50	175
*Bombyx mori*	7	44	11	81	143
*Anopheles gambiae*	63	157	47	70	337
*Ostrinia furnilicas*	29	105	9	34	177

*cSP* clip-domain serine protease, *cSPH* clip-domain serine protease homologs.

Notably, 43 of 177 SP-related genes were considered SPHs owing to the substitution of at least one conserved residue in the catalytic triad. These genes may encode cofactors for reactions. The remaining 134 SPs with enzymatic activity cleaved and activated downstream SPs/SPHs. An analysis showed that 80, 34 and 6 of the 134 SPs showed trypsin, chymotrypsin, and elastase substrate specificity (Supplementary Table S1).

### Clip domain SP/SPH

**Figure 1:**
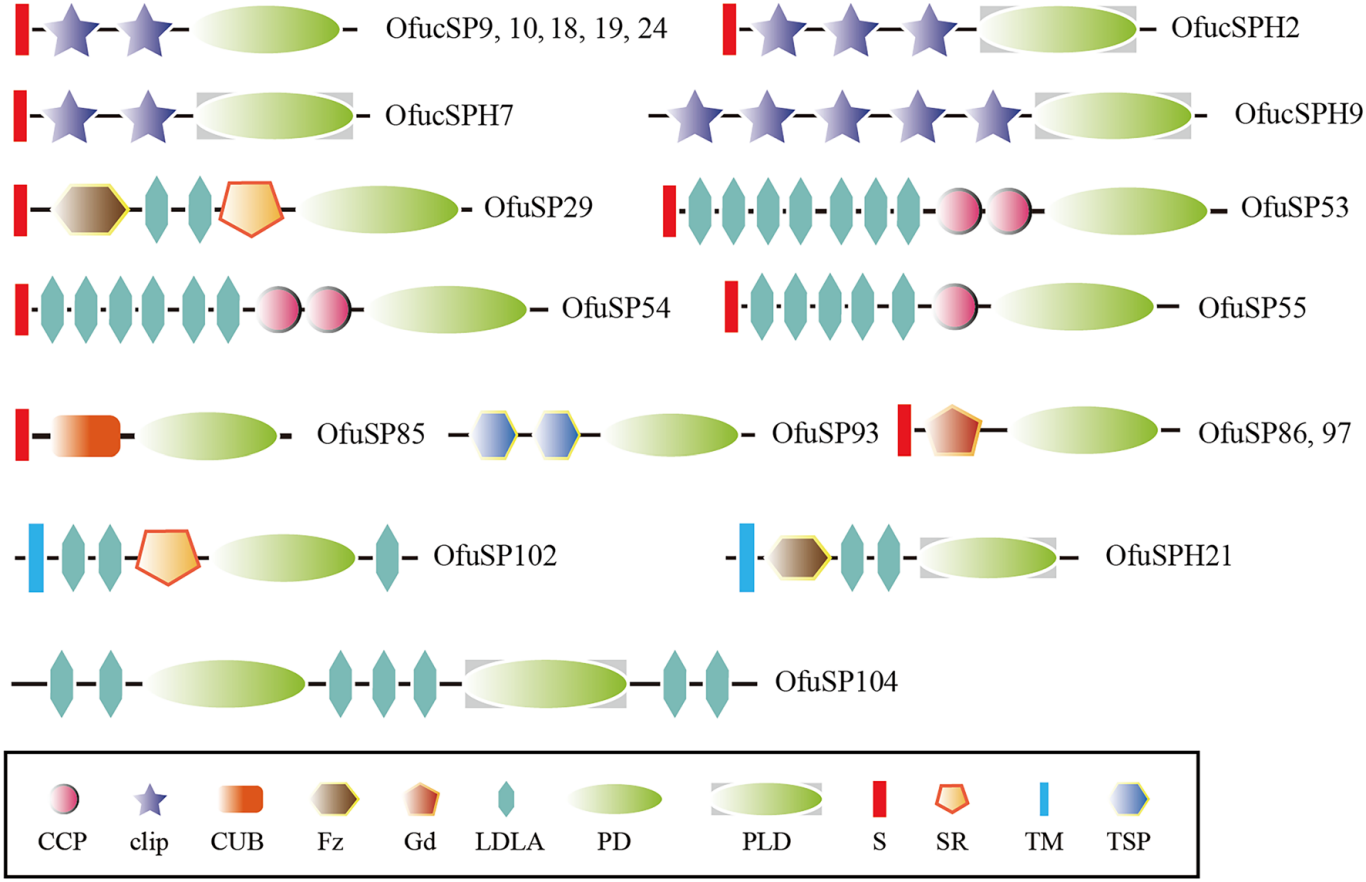
Domain organizations of SPs/SPHs with other structural elements in *Ostrinia furnacalis*. The schematic diagrams are not drawn to scale. *CCP* Sushi domain, also known as Sushi or SCR, *CUB* A domain identified in complement 1r/s, uegf, and bmp1, *Fz* Frizzled domain, *Gd* Gastrulation defective, *LDLA* LDLA-receptor class A domain, *PD* Serine protease domain, *PLD* serine protease-like domain, *S* Signal peptide, *SR* Scavenger receptor domain, *TM* Transmembrane region, *TSP* Thrombospondin.

**Figure 2:**
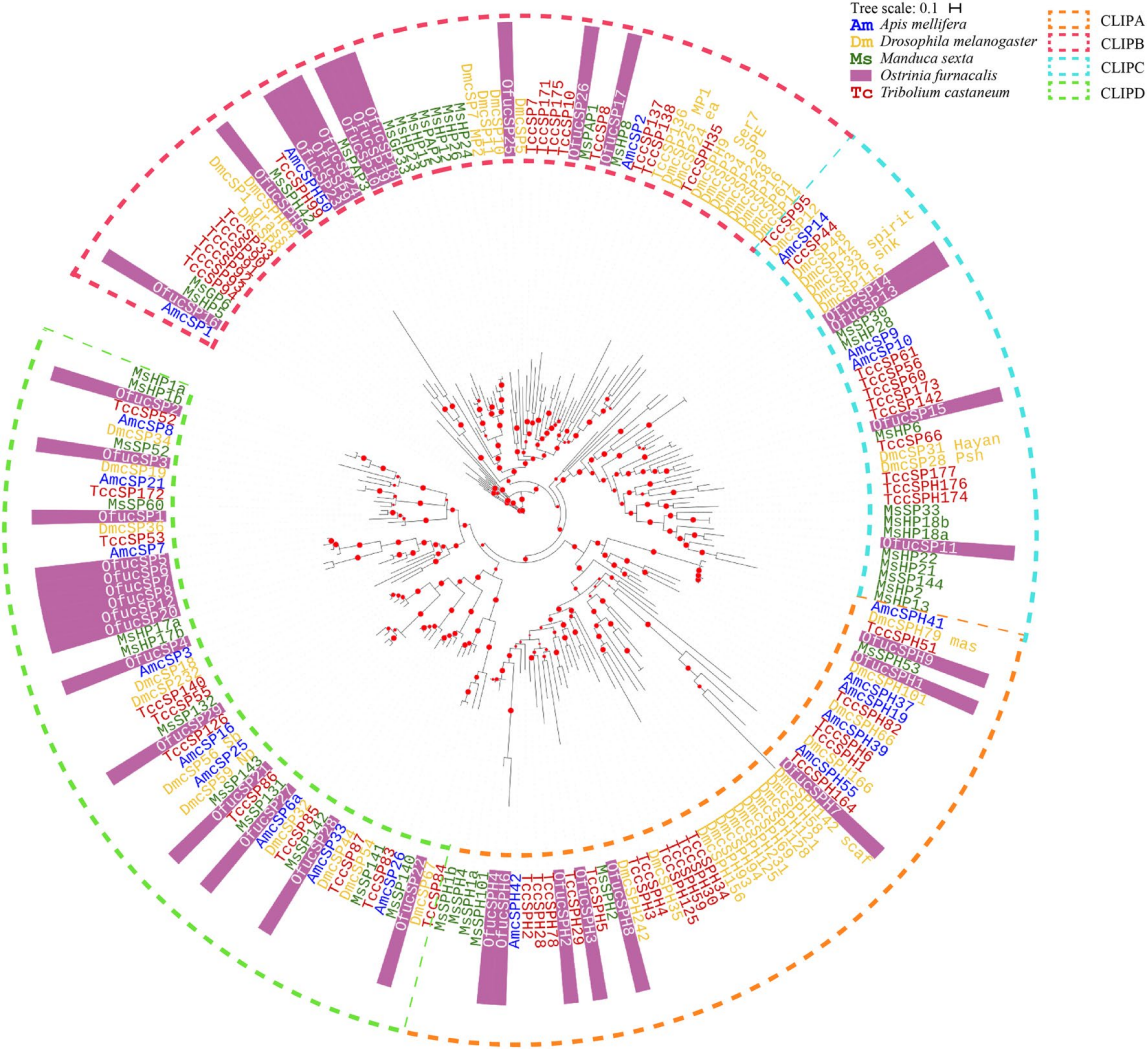
Phylogenetic relationship of CLIPAs, CLIPBs, CLIPCs and CLIPDs in five insects. The serine protease-like domains of *Ostrinia furnacalis* (Ofu), *Apis mellifera* (Am*), Manduca sexta* (Ms), *Drosophila melanogaster* (Dm) and *Tribolium castaneum* (Tc) were aligned. The phylogenetic tree was constructed by IQ-TREE. SPs/SPHs from different species were marked with different color. Red spots at the nodes denoted bootstrap values were greater than 90 from 100 trials.

The clip domain can be sequentially activated to form a cascade pathway and play an important function in the immune response and embryonic development. An analysis revealed 29 cSP and 9 cSPH genes in the genome of *O. furnacalis*. Most CLIPs (30) carried a single clip domain, whereas 5 OfucSPs had 2 clip domains, and OfucSPH2, OfucSPH7, OfucSP9 had 3, 2, and 5 clip domains (Fig. 1). SP/SPH genes were assigned to four distinct clades based on the phylogenetic calculation results and were classified as CLIPAs to CLIPDs based on homologous serine protease-like genes that have been previously characterized (Fig. 2). The *O. furnacalis* genome included 8 CLIPAs, 11CLIPBs, 4CLIPCs and 15 CLIPDs. The total number of CLIPs in the Asian corn borer was within the median range compared with CLIPs in other related insect species. The ratio of *O. furnacalis* CLIPCs was the lowest among these species, whereas the number of *O. furnacalis* CLIPDs was highest among other species (Supplementary Table S2).

Based on the phylogenetic analysis, all OfucSPHs belonged to the CLIPA group except OfucSPH5, which formed clusters with cSPH genes in the CLIPB group (Fig. 2). OfucSP26 and OfucSP17 were orthologous to MsPAP1 and MsHP8, respectively. OfucSP10, 18, 19, and 24 clustered together with MsPAP3. OfucSP15 in the CLIPC group shared high amino acid sequence identity with MsHP6. There were 24 orthologous groups characterized by bioinformatics calculation, including 7 single copy orthologous sets (six in the CLIPD group and one in the CLIPA group) and 2 *O. furnacalis* specific orthologous sets (Supplementary Table S3).

### SPs/SPHs with other domains

Eleven of the 177 SPs/SPHs with other domains were observed in the *O. furnacalis* genome. These domains included the LDLA domain, Fz domain, CCP domain, CUB domain, thrombospondin (TSP) domain, scavenger receptor Cys-rich (SR) domain and Gd domain (Fig. 1). The domain structure of *O. furnacalis* SP-like proteases was compared with that of proteases in fruit flies (Table 2). The findings showed that *O. furnacalis* SPs/SPHs were classified into 8 types of multidomain proteins, which was fewer than the number identified in *D. melanogaster*. OfuSP104 was orthologous to the Nudel gene that contains a serine protease-like domain between LDLA domains. OfuSP86 and OfuSP97 harbored Gd-like domains. OfuSP53/54/55 carried LDLAs followed by a CCP domain, and were probably ModSP-like proteases. OfuSP29 had Fz, LDLA and SR domains and shared domain similarity with Corin^35^.

**Table 2:** Domain organization of the multi-domain serine proteases and their homologs in *Ostrinia furnacalis* and *Drosophila melanogaster*.

Name	*Drosophila melanogaster*		*Ostrinia furnacalis*	
	Seq name	domain structure	Seq name	domain structure
Nudel	Ndl/SP89	TM-3LDLA-PD-2-4LDLA-PLD-3/4LDLA	OfuSP104	2LDLA-PD-3LDLA- SPH-2LDLA
Tequila	Tequila/SP72	S-2/15CBD-LDLA-SR-LDLA-SR-PD	/	/
Corin	Corin/SP75	TM-Fz-2LDLA-SR-PD	OfuSP29	S-Fz-2LDLA-SR-PD
SEA	SPH198	TM-SEA-Fz-2LDLA-PLD	OfuSPH21	TM-Fz-2LDLA-PLD
ModSP	ModSP/SP53	S-4/5 LDLA-CCP-Wonton-PD	OfuSP53	S-7LDLA-2CCP-PD
			OfuSP54	S-6LDLA-2CCP-PD
			OfuSP55	S-5LDLA-CCP-PD
TSP	SP30	S-2TSP-PD	OfuSP93	2TSP-PD
Masquerade	Mas/cSPH79	S-4/5 clip-PLD	OfucSPH9	5clip-PLD
CUB	SP120, SP80a-b	S-CUB-PD	OfuSP85	S-CUB-PD
Gd	SP186/Gd, 55, 60, 212, SPH144	S-Gd-PD	OfuSP86	S-Gd-PD
			OfuSP97	S-Gd-PD

*CBD* Type-2 chitin binding domain, *CCP* Sushi domain, also known as Sushi or SCR, *CUB* A domain identified in complement 1r/s, uegf, and bmp1, *Fz* Frizzled domain, *Gd* Gastrulation defective, *LDLA* LDLA-receptor class A domain, *PD* Serine protease domain, *PLD* Serine protease-like domain, *S* Signal peptide, *SEA* A domain identified in a sperm protein, enterokinase and agrin, *SR* Scavenger receptor domain, *TM* Transmembrane region, *TSP* Thrombospondin, *Wonton* A Sushi-like module first identified in M. sexta HP14 with six instead of four Cys residues.

### Scaffold location of SPs/SPHs

**Figure 3:**
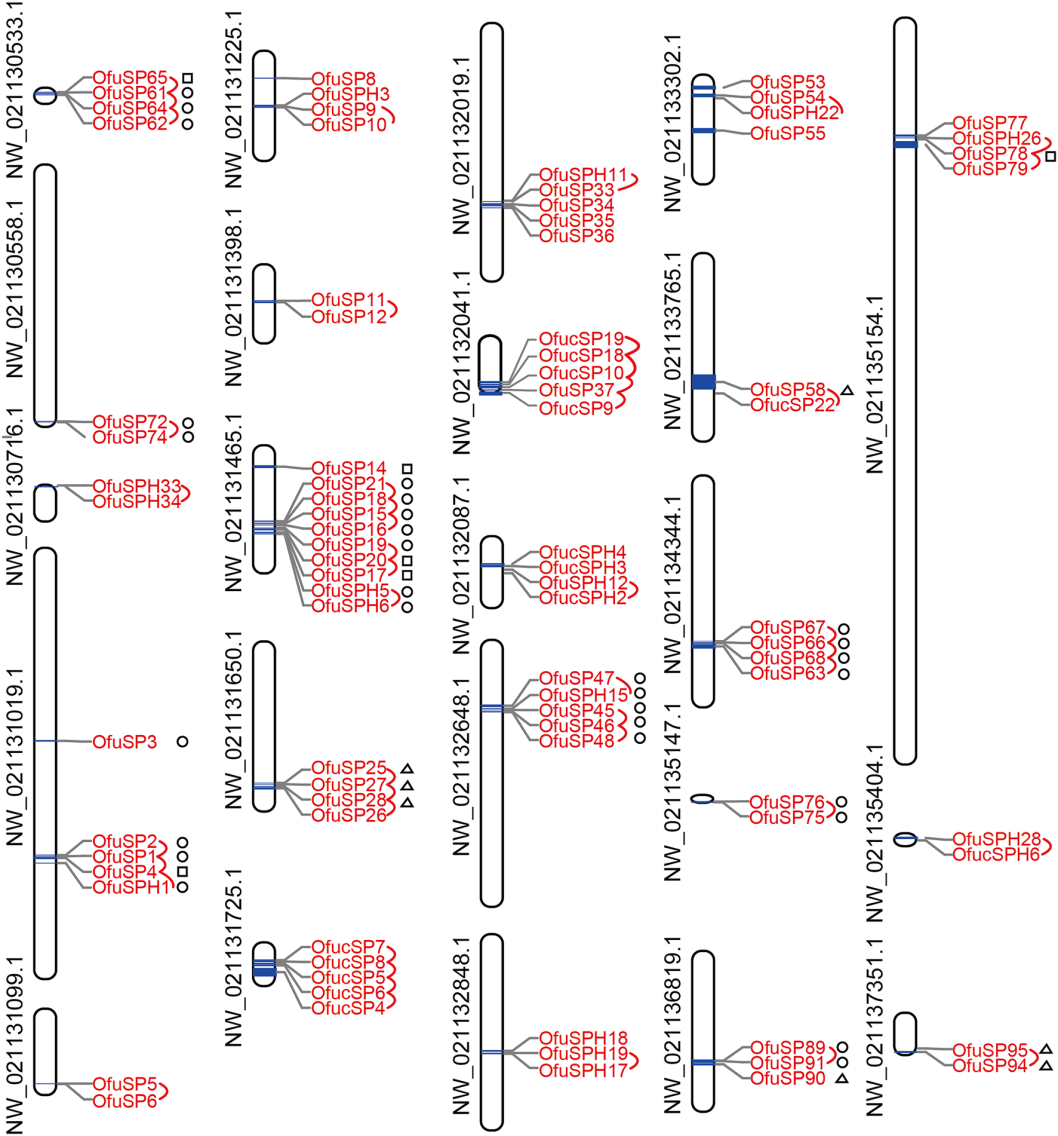
Scaffold location of *Ostrinia furnacalis* SP and SPH genes. Scaffold name and gene names were shown on the left and right of the bar, respectively. The length of scaffold and the distance of two adjacent genes were scaled in figure. Adjacent genes underwent duplications were associated with red curve. Genes differentially upregulated in midgut were marked by symbols. “○”presented genes upregulated in midgut compared to genes in other three tissues; “□”presented genes upregulated in midgut compared to genes in either two of three tissues; “△”presented genes upregulated in midgut compared to genes in one of three tissue.

Gene duplication is common in the serine protease family. Gene duplication of 177 *O. furnacalis* SP and SPH genes was identified in the current study. Duplicated gene pairs were generated and are presented in Fig. 3. Based on this analysis, more than 40% (71/177) of the genes in the SP gene family underwent gene duplication (Fig. 3). Analysis showed that 9 SP/SPH genes on NW_021131465.1 were duplicated genes and formed the largest gene cluster. OfucSP5-8 formed a clade in the phylogenetic tree (Fig. 2), while they are also considered as duplicate genes and clustered in one scaffold.

### The expression patterns of SP/SPH genes

We performed deep RNA sequencing (RNA-seq) libraries of *O. furnacalis* with samples obtained from four developmental stages and from four different tissues to obtain the comprehensive expression profiles of *O. furnacalis* SPs and SPHs. Approximately 1.4 billion raw reads were obtained from 30 libraries, and approximately 43–51 million reads were generated per library. After removing the adaptor sequences, ambiguous reads and low-quality reads, approximately 1.36 billion clean reads were obtained. The average ratio of clean reads mapped to the reference genome was 83.48% (Supplementary Table S4).

**Figure 4:**
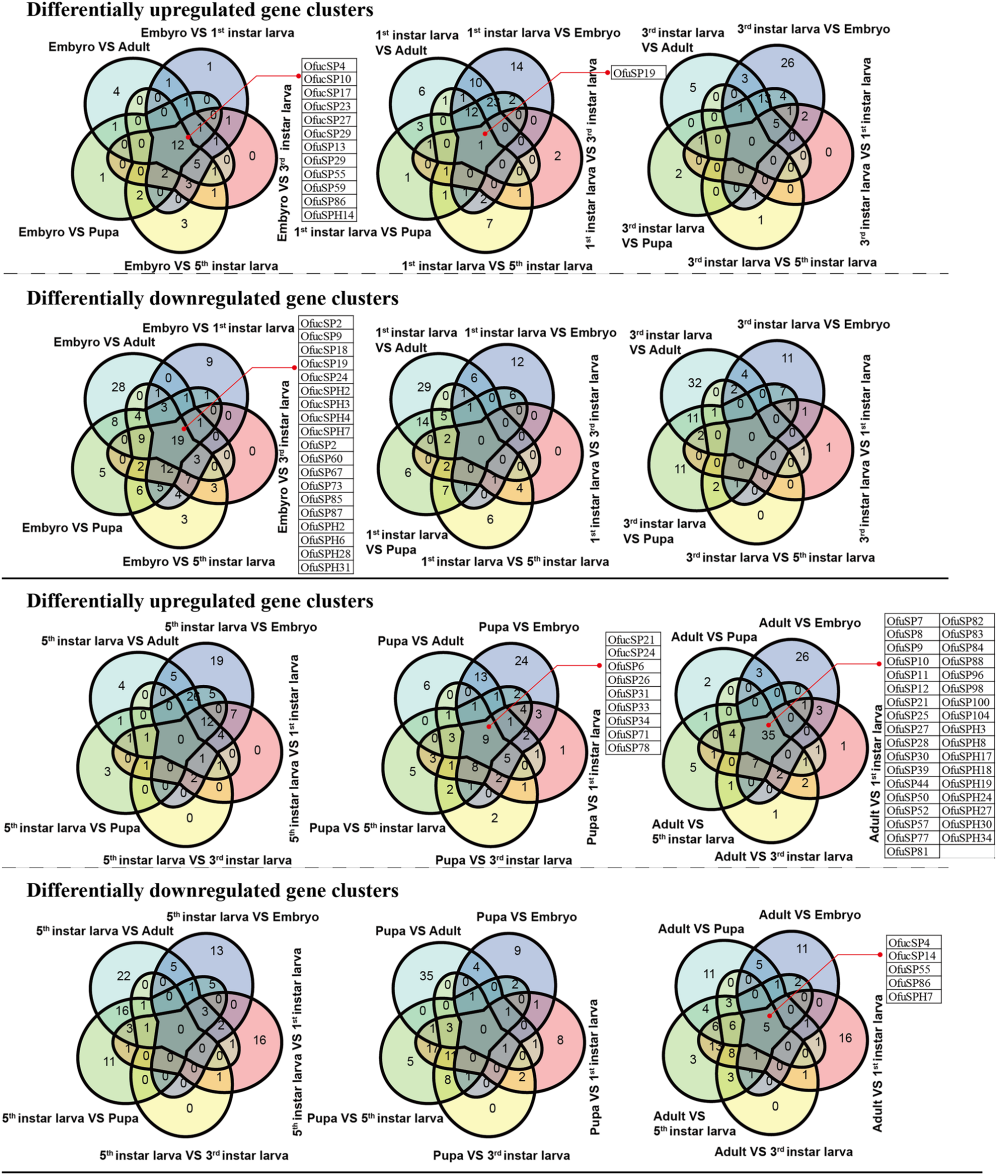
Venn diagram presented the number of upregulated or downregulated genes in embryo stage, newly hatched larval stage, middle larval stage, mature larval stage, pupal stage, and adult stage. The genes commonly upregulated or downregulated in one stage were listed. 1st instar larva, 3rd instar larva and 5th instar larva presented the newly hatched larvae, middle stage larvae and mature larvae.

**Figure 5:**
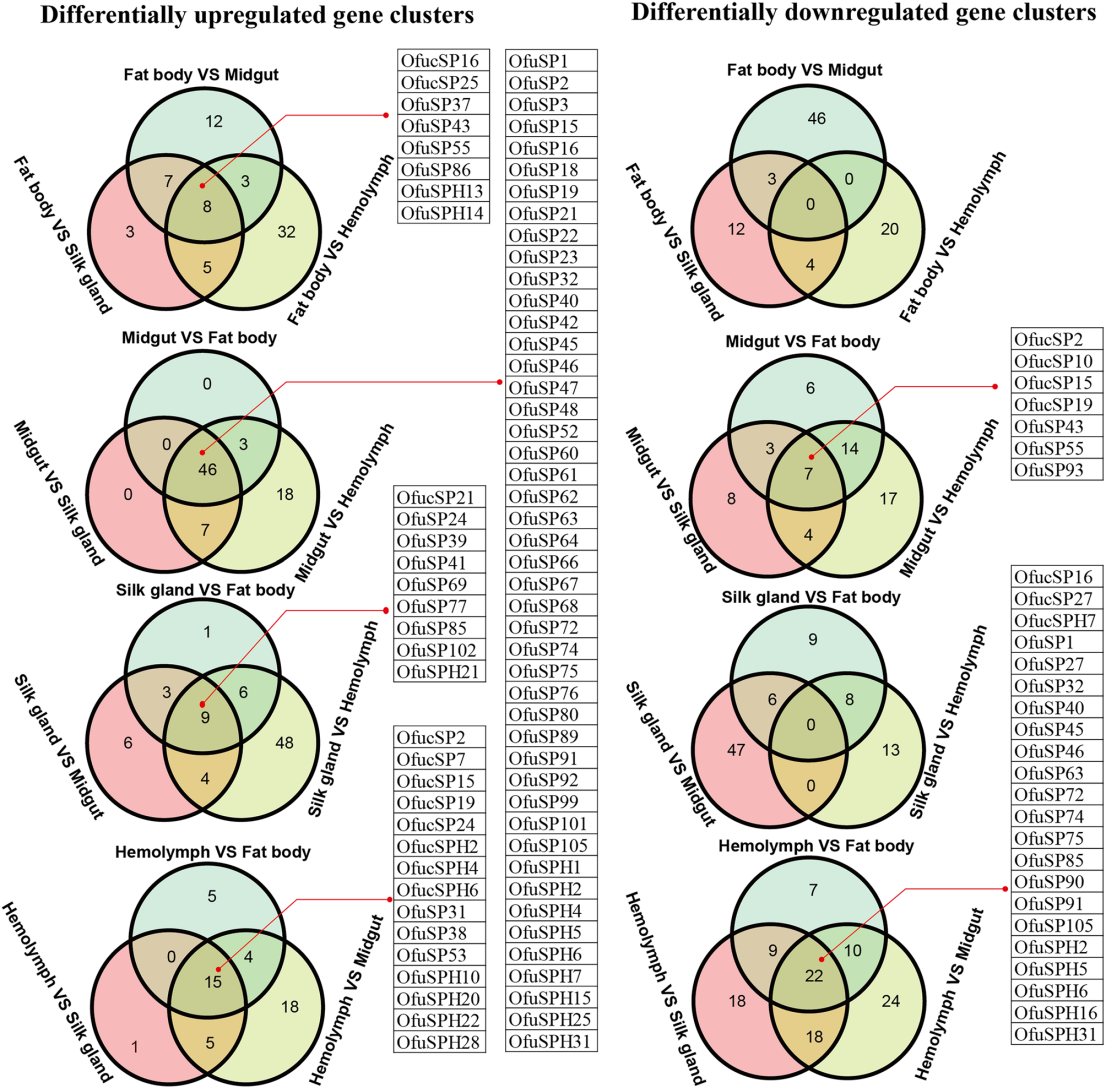
Venn diagram presented the number of upregulated or downregulated genes in midgut, fat body, silk gland and hemolymph. The genes commonly upregulated or downregulated in one stage were listed.

A series of genome-wide expression profiling comparisons were conducted. Via pairwise comparison between samples at all these developmental stages and tissues, the number of differentially upregulated and downregulated genes were documented (Table 3). A Venn diagram shows the common or uniquely regulated genes at one stage or in one tissue (Figs. 4 and 5).

**Table 3:** A summary of significantly upregulated and downregulated genes in pairwise comparison in different developmental stages (A) and different tissues (B).

(A)						
	Embryo	1st instar larva	3rd instar larva	5th instar larva	Pupa	Adult
**Embryo**		66↑ 28↓	56↑ 27↓	81↑ 29↓	76↑ 20↓	77↑ 27↓
**1st instar Larva**			9↑ 6 ↓	30↑ 28↓	44↑ 46↓	58↑ 58↓
**3rd instar Larva**				6↑3 ↓	36↑ 27↓	53↑ 28↓
**5th instar Larva**					42↑ 50↓	55↑ 56↓
**Pupa**						47↑ 43↓
**Adult**						

(B)						
	Fat body	Hemolymph	Silk gland		Midgut	
**Fat body**		24↑ 48↓	19↑ 23↓		49↑ 30↓	
**Hemolymph**			67↑ 21↓		74↑ 42↓	
**Silk gland**					53↑ 22↓	
**Midgut**						

“↑” represent specifically upregulated genes in row compared to that in column. “↓” represent specifically downregulated genes in row compared to that in column. 1st instar larva, 3rd instar larva and 5th instar larva present the newly hatched larvae, middle stage larvae and mature larvae, respectively.

There were more downregulated genes than upregulated genes between the embryo stage and other developmental stages. The results showed that 66, 56, 81, 76 and 77 SP/SPH genes were downregulated and 28, 27, 29, 20, and 27 SP/SPH genes were upregulated in the embryo stage compared to the newly hatched larval stage, middle larval stage, mature larval stage, pupal stage, and adult stage, respectively (Table 3). Among these genes, 31 SP/SPH genes were commonly differentially up- or downregulated in the embryo compared to the genes in the other four developmental stages, and approximately 50% (15/31) of these genes carried clip domains (Fig. 4). A comparative analysis of differentially expressed genes in the pupal stage revealed limited commonality of specifically expressed genes (only nine upregulated genes). Insight into gene expression profiles in the adult stage resulted in a total of 35 commonly and specifically highly expressed genes in comparison to genes expressed in other developmental stages, and none of these SP/SPH genes carried a clip domain (Fig. 4). Since there were few genes significantly expressed between either of the two larval stages, no common genes were identified at the middle- or mature-larval stage compared to the genes in the other four developmental stages, and only one commonly upregulated gene was identified in the newly hatched larval stage.

Analysis of gene expression patterns in different tissues showed that the number of SP/SPH genes that were differentially upregulated in the midgut was greater than the number of downregulated genes. Moreover, 46 commonly regulated genes and 7 genes shared with low expression were identified in the midgut (Fig. 5). All the upregulated genes formed simple gene structures (carrying only signal peptides and serine protease-like domains). There were 24, 21 and 42 SP/SPH genes highly expressed respectively in both the hemolymph and fat body; hemolymph and silk gland; hemolymph and midgut; the number of genes with low expression in these pairs was 48, 67 and 74, respectively (Table 3). In the fat body and silk gland, we only found common upregulated genes in comparison to genes in other three tissues (Fig. 5).

The expression patterns of SP/SPH genes in the midgut seemed to be correlated with their location in the scaffold. The genes highly expressed in the midgut are marked in Fig. 3, and a trend was found in the expression patterns, i.e., genes clustering on the same scaffold showed similar expression profiles. The transcript levels of SP and SPH genes on scaffold NW_021131465.1 were high in the midgut (Fig. 3). All these genes were single protease domain SPs/SPHs at amino acid residues 256–260 (Supplementary Table S1). Genes located on NW_021132648.1, NW_021130533.1 and NW_021134344.1 showed similar simple gene structures and expression profiles.

## Discussion

A total of 177 SP/SPH genes were identified in *O. furnacalis* genome. Since the genome of *O. furnacalis* was sequenced by second-generation sequencing technology, not in combination with third-generation sequencing technology, the quality of *O. furnacalis* gene models was not as good as that in some model insects*.* The quality of gene models is partly reflected in the percentage of sequences with signal peptides. There are 141 of the 177 (80%) *O. furnacalis* SPs/SPHs possess signal peptides. The percentage of SPs/SPHs with signal peptide in *O. furnacalis* is lower than that in *D. melanogaster* (248/257) and *A. gambiae* (324/337), similar to that in *Pteromalus puparum* (152/183)^36^, but higher than that in *Plutella xylostella* (105/221) and *T. molitor* (142/200)^37,38^. With the cost of sequencing the genome decreased, we can improve *O. furnacalis* serine protease gene models by the use of the third-generation sequencing technology in the future work.

SPs are catalyzed into active enzymes that activate downstream SPs and SPHs. The findings showed that more than 60% (114/177) of SPs/SPHs were catalyzed by trypsin-like proteases. Most SPs exhibited trypsin substrate specificity (80/134). The majority of SPs/SPHs with trypsin-like properties were observed in insects such as *D. melanogaster*, *A. gambiae* and *M. sexta*^4,26,27^. Notably, the analysis showed a tandem repeat distribution in *O. furnacalis* SP/SPH genes (71/177). Genes adjacent to scaffold are essential for the expansion of the gene family, and unequal cross over may be an important mechanism for the generation of these clusters^1^. Gene duplication events have been reported in several insect species in this gene family^4,23,24,26,27^. An entire 11.1-kb region that contains 7 trypsin genes were amplified by PCR in *A. gambiae*^39^. Long segments harboring 5 trypsin genes also have been found in *D. melanogaster* and *Drosophila erecta* by PCR amplification^40^. We considered that gene duplication occurred in the serine protease family of *O. furnacalis* and some serine proteases were duplicated genes. In addition, an analysis of tandem repeat genes was based on the location information in the scaffold, not the chromosome, implying that some duplicated genes may have been left out.

The functions of SPs/SPHs were predicted by gene structures, construction of phylogenetic tree and determination of relative expression levels. Proteases in the CLIPB and CLIPC groups of several related species were included in the analysis. The findings showed that the CLIPC group comprised serine proteases upstream of SP cascades with members serving as activators of terminal proteases. Activated by CLIPCs, CLIPBs were cleaved and activated downstream proSatzles or proPOs^15^. An analysis revealed that the sequence of OfucSP17 showed high similarity with that of MsHP8, implying that OfucSP17 exhibits functions similar to those of MsHP8 and may play a role in the Toll pathway and induce the expression of AMP genes. OfucSP19 and OfucSP24, also known as *O. furnacalis* SP105 and OfPAP were components of the PPO activation system^33,34^. In addition, OfucSP19, OfucSP24 and MsPAP3 all comprised 2 clip domains. Similar sequence structures and functions of PAP-like genes in *M. sexta* and *O. furnacalis* indicate that these genes were involved in similar protease pathways. OfucSP10 and OfucSP18, formed a cluster with MsPAP3, OfucSP19 and OfucSP24 and harbored 2 clip domains. These findings suggested that OfucSP10 and OfucSP18 may exhibit functions similar to those of MsPAP3, OfucSP19 and OfucSP24 and may be involved in the melanization cascade.

An analysis showed OfucSP5-8 were different from other Clip D genes and displayed lineage-specific expansion. These four genes in this group showed similar expression patterns and were specifically upregulated in the middle and mature larval stages. The roles played by OfucSP5-8 should be explored further. Except 6 CLIPDs (OfucSP5-8, OfucSP12 and OfucSP20) considered as paralogous genes, the remaining 9 *O. furnacalis* CLIPDs were identified in 9 orthologous sets with CLIPDs from other four insects, indicating that most of CLIPDs were conserved.

In addition, several other genes that shared a high identity with known functional genes were identified through phylogenetic calculations. OfucSPH9 clustered with 5-clip SPHs in other species, indicating that the domain structure has been conserved and is similar across species. OfucSPH8 shared high identity with *M. sexta* SPH-2. MsSPH2 was a cofactor of PAP3 and has been implicated in mediating PO activity levels and can interact with immunlectine-2 by binding to lipopolysaccharide^41,42^. OfucSP2 presented high similarity with MsHP1a/b and clustered with TccSP52, DmcSP34 and AmcSP8. *M. sexta* proHP1 exerts its activity without proteolytic activation and can stimulate proHP6 to induce antimicrobial peptide synthesis and melanization^43^. These known functional serine protease-like proteins provide the potential function and characteristic of serine proteases in *O. furnacalis*.

Multidomain SPs can interact with other proteins through various structural modules to participate in more physiological processes^4,16^. SP cascades induce the dorsal–ventral axis reported in *Drosophila*. OfuSP104 is homologous to the Nudel gene and has been implicated in the processing of Gd. OfuSP86 and OfuSP97 were predicted to be Gd genes. These two genes play a role in cleaving and activating Snake, which subsequently processes Easter, and then cleaves Spatzle to form the active ligand for the Toll receptor^22,44^. The domain structures of OfuSP53/54/55 were similar to those of ModSP-like proteases, implying that they may interact with pattern recognition molecules and indirectly activate downstream proteins^16^.

Differentially expressed genes among different developmental stages and tissues were acquired and gave us comprehensive insight into their properties and functions. Approximately one-half of the genes highly expressed in embryos were CLIPs, including 3 CLIPBs and 3 CLIPDs. These genes may play essential roles in embryonic development. Hemolymph and fat bodies are important organs involved in immune defense. Five clip-domain SP genes and 3 clip-domain SPH genes, which are commonly highly expressed in the hemolymph compared to genes expressed in other tissues, may be involved in immune signaling pathways. More SPs/SPHs showed specifically high expression in the midgut in pairwise comparisons with genes in other tissues. Since serine-like proteases formed dominate population in digestive environment^5^, these midgut-specific upregulated genes may exert important roles in food digestion. An interesting phenomenon was that most of the tandem repeat SP/SPH genes with simple gene structures displayed similar upregulated expression profiles in the midgut. The common expression values were whether due to the multi-mapping of RNA-seq reads on homologous region of the genome, or the result of gene duplicated events, should be explored in further studies.

In summary, the present study classified the SP-related genes of *O. furnacalis*, and explored their gene structures, sequence characteristics, and expression patterns, thus providing a comprehensive understanding of *O. furnacalis* SP/SPH genes. Orthologous comparisons of SP/SPH genes between *D. melanogaster*, *M. sexta* and *O. furnacalis* provide a basis for functional studies on *O. furnacalis* SPs/SPHs.

## Materials and methods

### Insect rearing

Asian corn borers were reared under standard insectary conditions at 25 ± 1 °C and 75 ± 5% relative humidity with a photoperiod of 14 h: 10 h (light: darkness). Newly hatched larvae were fed with an artificial diet until they pupated, and adults were fed with 10% sucrose solution^30^.

### Identification of *O. furnacalis* SP/SPH genes

The genome of *O. furnacalis* (accession number: ASM419383v1) was retrieved from NCBI. BUSCO was used to estimate genome assembly and for annotation completeness^45^. Arthropoda_odb10 and Lepidoptera_odb10 were chosen for performing this assessment. The genome file and GTF file of *O. furnacalis* were used to extract coding sequence regions as nucleotide sequences, and these sequences were batch translated into proteins with TBtools^46^. Serine protease domain sequences from known *D. melanogaster* and *M. sexta* serine protease sequences^4,26^ were used as queries for the BLAST search (E-value 1e-5). Identified genes were scanned by NCBI online BLASTP to remove sequences without serine protease domains. The remaining sequences were crosschecked with the *O. furnacalis* protein database via BLASTP to ensure that all the SP-like genes have been obtained. The identification of SPs and SPHs was based on the conserved His, Asp and Ser residues in catalytic triad residues (conserve region) of the serine protease domain. Sequences containing all three residues in TAA**H**C, **D**IAL and GD**S**GGP motifs were considered SPs. Sequences with amino acid substitution in one or more of these three residues were considered SPHs ^23,26^. The catalytic triad residues of these genes were manually checked, and severely incomplete sequences (sequence incomplete due to a missing catalytic triad residue in the serine protease domain) were removed. Fgenesh + was used for gene prediction to verify the accuracy of the assembly artifacts and annotation^47^. We retained only the longest sequence when one gene encoded more than one isoform. Finally, the amino acid sequences are listed in the Supplementary file in FASTA format. Signal peptides were predicted using SignalP 6.0 with default parameters. Pfam and ScanProsite were used to predict the domain structure of the protein sequences. Sequences with four cysteine residues and one cysteine doublet upstream of a serine protease domain were called clip SPs or SPHs. Residues 189, 216, and 226 in SPs determined the main specificity of the S1 pocket. SPs with Asp189, Gly216, and Glu/Ala/Ser226 played a role in the activation of trypsin-like proteases. SPs with Ser/Thr189, Gly216 and Gly/Ala/Ser226 residues conferred chymotrypsin-like protease activity. The SPs catalyzed elastase-like proteases when larger and nonpolar residues occur at position 216 or 226^3,4,26^. Three residues in the SP genes mentioned above were identified by sequence alignment, and the possible enzymatic activities of these SPs were predicted. Clip-domain serine protease-like sequences of *O. furnacalis, D. melanogaster*, *T. castaneum*, *A. mellifera* and *M. sexta*^4,26^ were obtained and used to characterize orthologs via OrthoFinder2^48^.

### Sequence alignment and phylogenetic analysis

SP/SPH protease-like domains of *O. furnacalis* were aligned with those of *D. melanogaster*, *T. castaneum*, *A. mellifera* and *M. sexta* sequences^4,26^*.* Amino acid sequence alignment were first analyzed by MAFFT and then trimmed by trimAI via default parameters^49,50^. ModelFinder^51^ implemented in IQ-TREE^52^ was used to seek the best substitution model according to the Bayesian Information Criterion (BIC). A phylogenetic tree was constructed by IQ-TREE with 1000 bootstrap replicates using the LG + I + G4 model suggested by ModelFinder. All the amino sequences used to construct phylogenetic tree has been listed in Supplementary file in FASTA format. The multiple sequence alignment of these serine protease-like sequences and the phylogenetic tree in newick format were also presented in Supplementary file.

### Scaffold location and gene duplication of SP/SPH genes

Genomic location information of SP/SPHs of *O. furnacalis* was retrieved from the NCBI genome database, visualized using TBtools^41^, and then improved with Adobe Illustrator. MCScan with default parameters was used to analyze SP/SPH sequences and provide an integrated view of gene duplication^53^. Genes commonly differentially upregulated in the midgut in comparison with other tissues are marked with symbols.

### Sample collection and RNA extraction

A graphic presentation of RNA-seq sample preparation is illustrated in Supplementary Figure S2.* O. furnacalis* embryos were collected less than 12 h post-oviposition. The 1st, 3rd and 5th instars of the Asian corn borer, which represent the newly hatched larvae, middle stage larvae and mature larvae, were harvested for subsequent experiments. Pupae and adults were grouped into females and males, and then, female and male samples were mixed at a ratio of 1:1. The samples were anesthetized at −70 °C for 8 min, washed three times in DEPC water, and then frozen in liquid nitrogen. Every 20 embryos or 20 1st instar larvae were pooled as one sample, and every six individuals in the other 4 insect stages were pooled as one sample.

The 3rd instar larvae were anesthetized on an ice plate for tissue extraction. The midgut, fat body and silk gland were isolated and transferred to 2-ml centrifuge tubes containing TriPure reagent. Twenty midguts, fat bodies or silk glands were pooled as one sample. Larvae and curved scissors were sterilized with alcohol-containing DEPC, and then bled onto Parafilm by cutting away the proleg. One hundred microliters of hemolymph from 20 larvae was homogenized using TriPure reagent. Three replicates of each tissue sample and three replicates of each developmental stage sample were obtained and processed for RNA extraction according to the manufacturer’s instructions.

### Transcriptome sequencing and data analysis

Total RNA was used as input material for library preparation. Briefly, mRNA was purified from total RNA by using poly-T oligo-attached magnetic beads. First-strand cDNA was then synthesized using mRNA fragments as templates, followed by second-strand cDNA synthesis using DNA polymerase I and RNaseH. After the construction of the library, the library was tested to ensure its quality. Qualified libraries were sequenced by the Illumina NovaSeq 6000 to generate 150-bp paired-end short reads. Raw FASTQ format data were processed by fastp 0.19.7 with the parameter -q 5 -u 50 -n 15 -l 150^54^. In this step, clean data were obtained by removing reads containing adapters, reads containing N-bases and low-quality reads from the raw data. The Q20, Q30 and GC contents of the clean data were calculated. The reference genome and gene model annotation files of *O. furnacalis* were downloaded. The index of the reference genome was built using HISAT2 2.0.5^55^, and paired-end clean reads were aligned to the reference genome using HISAT2 with the parameter -p 4 –dta -t –phred33. All the transcriptome data are available on the website of the NCBI GEO database (GEO accession: GSE197663).

Nucleotide sequences extracted from the genome of *O. furnacalis* were used as reference sequences, and the expression levels of these sequences were quantified by mapping the Illumina reads to the reference sequences with RSEM software using default parameters. Differential gene expression analysis was conducted using the DESeq2 1.16.1^56^. DEGs with log2(ratio) ≥ 1 & q-value < 0.05 or log2(ratio) ≤ -1 & q-value < 0.05 were classified as significantly upregulated or downregulated genes^57,58^ (Supplementary Table S5).

## Supplementary Information

Supplementary Information.

## Data Availability

The RNA-seq datasets generated in this study have been submitted to Gene Expression Omnibus (GEO) database at the National Center for Biotechnology Information (NCBI) under accession no. GSE197663.
