# Supplementary material for: Identification and gene expression analysis of serine proteases and their homologs in the Asian corn borer Ostrinia furnacalis
Source: Sci Rep. 2023 Mar 23;13:4766. doi: 10.1038/s41598-023-31830-2 (PMC10036332; doi:10.1038/s41598-023-31830-2)
Supplement: Supplementary file 1 — Supplementary Information. [file 41598_2023_31830_MOESM1_ESM.pdf]

Identification and gene expression analysis of serine proteases and their homologs in the Asian corn borer *Ostrinia furnacalis*

Lei Yang [1], Xiaoli Xu[1], Wei wei [1], Xiaoyun Chen [1], Cheng Peng [1], Xiaofu Wang [1] and Junfeng Xu [1]*

[1] Laboratory for Managing Biotic and Chemical Threats to the Quality and Safety of Agro-products, Zhejiang Academy of Agricultural Sciences, Hangzhou, 310021, China.

*Corresponding author: JF Xu, e-mail: njjfxu@163.com

Supplementary data

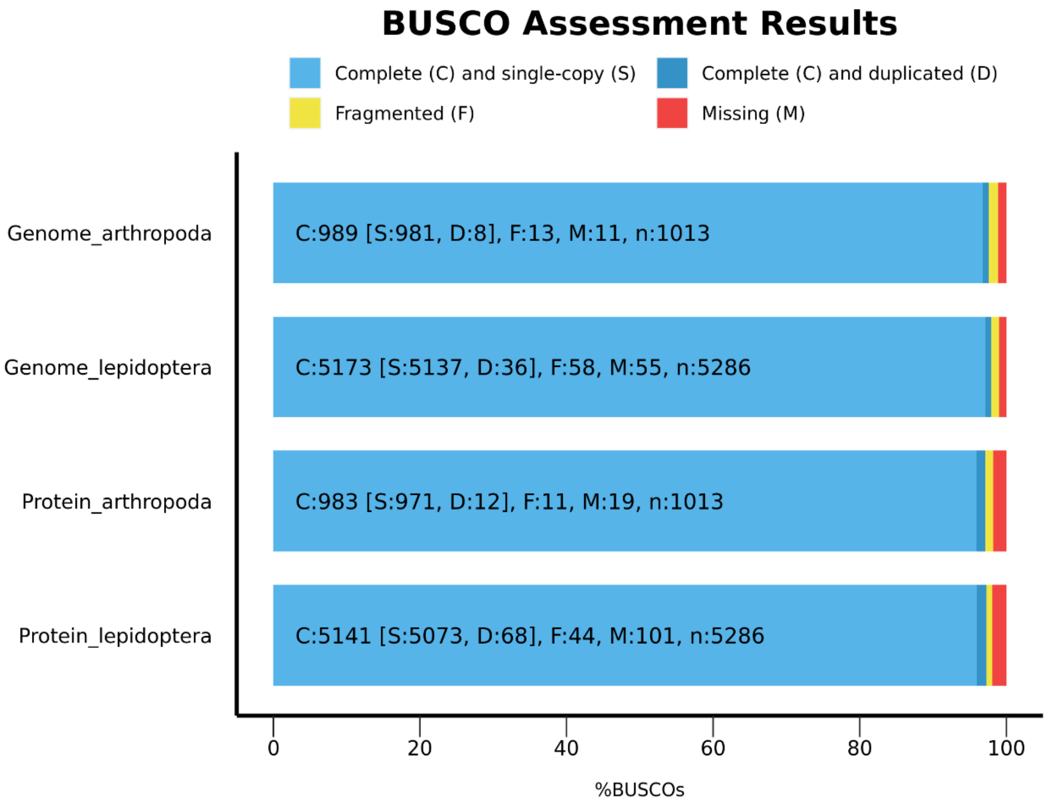

Supplementary Figure S1. Illustration of the BUSCO bar plot as produced by the plotting script. The genome and protein sequences of *Ostrinia furnacalis* were evaluated with Arthropoda and Lepidoptera lineage database. The result is depicted with varying degrees of completeness.

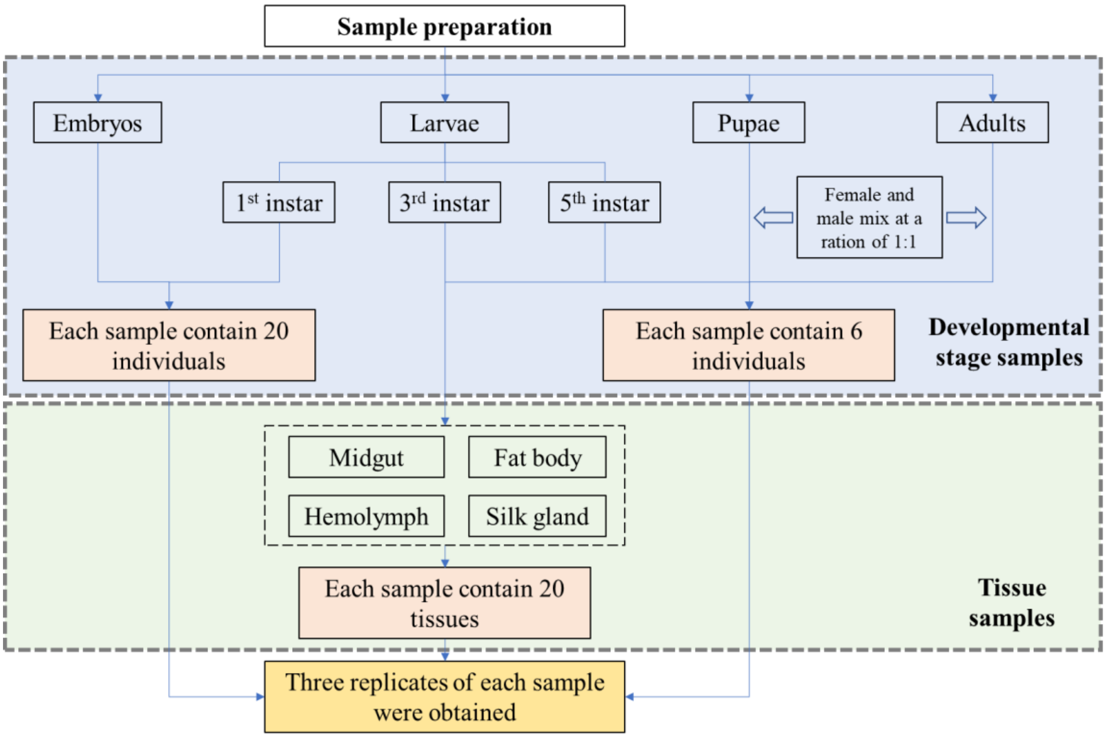

Supplementary Figure S2. A graphical representation of RNA-seq sample preparation.

Supplementary Table S1. Prediction of serine proteases and their homologs in *Ostrinia furnacalis*.

Gene name[a]	Gene ID[b]	RNA ID	Length (aa)	Conserved regions[c]			Activation site[d]	Enzyme specificity[e]	Domain structure[f]	Signal peptide[g]	Previous Orthologs[h]
				TAAHC	DIAL	GDSGGP					
**OfucSP1**	114349741	XM_028300221.1	533	SAAHC	DIAI		EDER^IVGG	T(DGG)	S-clip-PD	SP(1-24)	OfSP17
**OfucSP2**	114349965	XM_028300571.1	422	SAAHC	DIAI		PHGR^VTGA	T(DGG)	clip-PD	N	OfSP37
**OfucSP3**	114351430	XM_028302644.1	579	TAAHC	DIAL	GDSGGP	EGGR^IVGG	T(DGG)	clip-PD	N	
**OfucSP4**	114352940	XM_028304705.1	725		DIGI		TFSR^VVGG	T(DGG)	S-clip-PD	SP(1-19)	
**OfucSP5**	114352941	XM_028304708.1	420		DIAI		LKYV^ISNG	T(DGG)	S-clip-PD	SP(1-18)	
**OfucSP6**	114352943	XM_028304711.1	392		DIAI		DIFS^VYNG	T(DGG)	S-clip-PD	SP(1-18)	
**OfucSP7**	114352944	XM_028304712.1	385		DIAI		WMFV^ISNG	T(DGG)	S-clip-PD	SP(1-17)	OfSP5
**OfucSP8**	114352947	XM_028304717.1	342		DIAI		FMYV^IANG	?(D??)	S-clip-PD	SP(1-18)	
**OfucSP9**	114353727	XM_028305809.1	421				SASR^IFGG	T(DGG)	S-2clip-PD	SP(1-19)	OfSP7
**OfucSP10**	114353730	XM_028305811.1	424		DIGL		ADNK^ITGG	T(DGG)	S-2clip-PD	SP(1-19)	OfSP8
**OfucSP11**	114355003	XM_028307647.1	423	TAGHC			AEDL^IIGG	T(DGG)	S-clip-PD	SP(1-17)	OfSP2
**OfucSP12**	114358020	XM_028311876.1	385	TAGHC	DIGL		DEFP^LVCG	T(DGG)	S-clip-PD	SP(1-20)	
**OfucSP13**	114360991	XM_028315888.1	400	TAGHC		GDSGSP	SVEL^IIGG	T(DGA)	S-clip-PD	SP(1-21)	OfSP3
**OfucSP14**	114361541	XM_028316604.1	444	TAGHC		GDSGSP	GVKL^IVGG	T(DGA)	S-clip-PD	SP(1-17)	OfSP4
**OfucSP15**	114362371	XM_028317753.1	358				LDLH^IIGG	T(DGG)	S-clip-PD	SP(1-20)	OfSP1
**OfucSP16**	114352615.1	XM_028304278.1.1	420		DLAL		DSDR^IFGG	T(DGG)	S-clip-PD	SP(1-20)	OfSP14
**OfucSP17**	114352615.2	XM_028304278.1.2	429			GDSGGA	NDDR^IVGG	T(DGG)	clip-PD	N	OfSP12
**OfucSP18**	114353725.1	XM_028305803.1.1	422		DIGL	GSSGGP	THDK^IIGG	T(DGG)	S-2clip-PD	SP(1-19)	
**OfucSP19**	114353725.2	XM_028305803.1.2	424		DIGL		ADNK^ITGG	T(DGG)	S-2clip-PD	SP(1-19)	OfSP105
**OfucSP20**	114355408	XM_028308240.1	380	TAGHC	DIGL		ETSF^LLAS	T(DGG)	S-clip-PD	SP(1-20)	
**OfucSP21**	114357407	XM_028311004.1	1007		DLAL		PQGR^IVGG	T(DGG)	clip-PD	N	
**OfucSP22**	114357864	XM_028311651.1	439				LQKR^IIGG	T(DGG)	clip-PD	N	

**OfucSP23**	114358598	XM_028312617.1	658	TAGHC			LDNR^IAGG	T(DGS)	S-clip-PD	SP(1-39)	
**OfucSP24**	114359031	XM_028313261.1	400				AGDR^IMGG	T(DSA)	S-2clip-PD	SP(1-18)	OfSP10/OfP AP
**OfucSP25**	114362071.1	XM_028317328.1.1	384		DIGL		FTNK^IYGG	T(DGA)	S-clip-PD	SP(1-17)	
**OfucSP26**	114362071.2	XM_028317328.1.2	443		DIGL		NGDR^IYGG	T(DGG)	clip-PD	N	OfSP13
**OfucSP27**	114362171	XM_028317460.1	534		DVAL		RSNR^IVGG	T(DGG)	S-clip-PD	SP(1-39)	
**OfucSP28**	114365448	XM_028322027.1	1053		DLAL		KTGR^IVGG	T(DGG)	clip-PD	N	
**OfucSP29**	114366774	XM_028323752.1	564	TAGHC	DLAL		PETR^IMGG	T(DGG)	S-clip-PD	SP(1-18)	
**OfucSPH1**	114349870	XM_028300462.1	506	TVTHR	DITV	GDGGSG	DVRI^VTPG		S-clip-PLD	SP(1-17)	
**OfucSPH2**	114353861	XM_028305986.1	671	TVAHY		LDGGGP	EGCG^VANP		S-3clip-PLD	SP(1-17)	
**OfucSPH3**	114353865	XM_028305993.1	398	TVANR	DVGL	GDGGAP	TVAP^NIGG		S-clip-PLD	SP(1-20)	
**OfucSPH4**	114353866	XM_028305994.1	364	TAAHI	NYAL	GGGGSP	RRVD^IPGY		S-clip-PLD	SP(1-18)	
**OfucSPH5**	114359523	XM_028313957.1	365			SFGGSP	CGQS^MVRG		S-clip-PLD	SP(1-20)	
**OfucSPH6**	114360752	XM_028315543.1	370	TAAHV	NFAL	GAGGSP	KRAK^VSNF		S-clip-PLD	SP(1-20)	
**OfucSPH7**	114363065	XM_028318671.1	1445	TSASC	DAAI	VDIGSA	GQCG^LLNG		S-2clip-PLD	SP(1-20)	
**OfucSPH8**	114365830	XM_028322508.1	411	TTAHV	DVAL	GDGGAP	LDFT^ISGG		S-clip-PLD	SP(1-15)	
**OfucSPH9**	114366589	XM_028323509.1	647			GDGGGP	RSGR^VMGG		5clip-PLD	N	
**OfuSP1**	114350043	XM_028300676.1	288		DIAV		TDLR^IVGG	C (GGA)	S-PD	SP(1-19)	
**OfuSP2**	114350044	XM_028300677.1	287		DIAV		VDLR^IVGG	C (GGA)	S-PD	SP(1-19)	
**OfuSP3**	114350086	XM_028300731.1	600	SAAHC	DIGV		QQQR^IVGG	C (GGA)	S-PD-PD	SP(1-16)	
					DIAV			C(GGT)			
**OfuSP4**	114350090	XM_028300737.1	580		DIAV		VTGR^IVGG	C (GGA)	S-PD-PD	SP(1-19)	
					DIAV			C (GGA)			
**OfuSP5**	114350291	XM_028301024.1	325		DLAL		VHPT^VING	?(D?T)	S-PD	SP(1-17)	
**OfuSP6**	114350292	XM_028301025.1	315		DIAI		VKPV^VVNG	?(D?T)	S-PD	SP(1-16)	

**OfuSP7**	114350726	XM_028301633.1	293	TAGHC	DVAL		AALR^IFGG	?(???)	S-PD	SP(1-17)	
**OfuSP8**	114350793	XM_028301725.1	336		DLGL		LTGY^ILNG	T (DGS)	PD	N	
**OfuSP9**	114350795	XM_028301727.1	275			GDSGGG	AEGK^IVGG	T (DGS)	S-PD	SP(1-16)	
**OfuSP10**	114350798	XM_028301728.1	232		DMAM	GDSGGG	FEDR^VLGG	?(D??)	S-PD	SP(1-22)	
**OfuSP11**	114351498	XM_028302742.1	237	TSAHC	DVQL	GDSGGA	VVHG^ILGG	T(DGG)	PD	N	
**OfuSP12**	114351499	XM_028302743.1	337	TAGHC	DFQL		GEDK^IVGG	T(DGG)	S-PD	SP(1-21)	
**OfuSP13**	114351653	XM_028302936.1	787	TAGHC			KIPL^VVGG	T(DGA)	S-PD	SP(1-20)	
**OfuSP14**	114351915	XM_028303262.1	843		DVSI		TSER^IVGG	T(DGG)	S-PD-PD-PD	SP(1-16)	
				SAAHC	DISI			T(DGG)			
				SAAHC	DIGI			T(DGG)			
**OfuSP15**	114351933	XM_028303287.1	260		DICV		SVER^IIGG	T(DGG)	S-PD	SP(1-16)	
**OfuSP16**	114351935	XM_028303288.1	258		DISI		VIQR^IVGG	T(DGG)	S-PD	SP(1-16)	
**OfuSP17**	114351936	XM_028303290.1	258		DISI		KNNR^IVGG	T(DGG)	S-PD	SP(1-16)	
**OfuSP18**	114351937	XM_028303293.1	256		DVSI		NIQR^IVGG	T(DGG)	S-PD	SP(1-16)	
**OfuSP19**	114351940	XM_028303297.1	256		DIAI		NDHR^IIGG	T(DGG)	S-PD	SP(1-16)	
**OfuSP20**	114351942	XM_028303298.1	256		DIAI		NDHR^IIGG	T(DGG)	S-PD	SP(1-16)	
**OfuSP21**	114351943	XM_028303299.1	255		DVAI		RQTK^IVGG	T(DGG)	S-PD	SP(1-16)	
**OfuSP22**	114352487	XM_028304114.1	281		DVAV		DGGR^IWGG	C (SGG)	S-PD	SP(1-16)	
**OfuSP23**	114352553	XM_028304183.1	265		DVSI		TSER^IVGG	T(DGG)	S-PD	SP(1-16)	
**OfuSP24**	114352617	XM_028304279.1	674		DMAI		AADR^IIGG	T(DGG)	S-PD-PD	SP(1-21)	
					DISL	GDSGGS		T(DGG)			
**OfuSP25**	114352626	XM_028304287.1	264		DIAI		HNDG^IVGG	C(NGA)	S-PD	SP(1-18)	
**OfuSP26**	114352627	XM_028304288.1	262		DVAI		HDGR^IVGG	T(DGG)	S-PD	SP(1-16)	
**OfuSP27**	114352650	XM_028304324.1	301		DIGI	FDSGGP	HNDG^IVGG	C(NSA)	S-PD	SP(1-18)	
**OfuSP28**	114352651	XM_028304325.1	262		DIGI		HNHG^IVGG	C(NGA)	S-PD	SP(1-18)	

**OfuSP29**	114352910	XM_028304675.1	978	TASHC			ASKR^IVGG	T(DGG)	S-Fz-2LDLA-SR-PD	SP(1-26)	
**OfuSP30**	114353152	XM_028304999.1	290	TAGHC	DIGL		SALR^VLLG	C(SGG)	S-PD	SP(1-17)	
**OfuSP31**	114353202	XM_028305087.1	917				DPST^VFGG	T(DGG)	PD-PD	N	
					DIGL		DTSE^IFGG	T(DGG)			
**OfuSP32**	114353634	XM_028305693.1	283		DIMI		SMTR^IIGG	C(GGS)	S-PD	SP(1-18)	
**OfuSP33**	114353668	XM_028305737.1	288	SAAHC	DVAV		LIPR^IVNG	?(D??)	PD	N	
**OfuSP34**	114353670	XM_028305739.1	487		DIAV		LIPR^IVNG	?(D??)	S-PD-PLD	SP(1-23)	
					DIAV	/					
**OfuSP35**	114353671	XM_028305740.1	239		DLAI		/	T(DGG)	S-PD	SP(1-23)	
**OfuSP36**	114353672	XM_028305741.1	230	TQRHI	DIAI	GDSGGP	/	?(DG？)	PD	N	
**OfuSP37**	114353734	XM_028305816.1	277				SASR^IFGG	T(DGG)	PD	N	
**OfuSP38**	114353957	XM_028306155.1	390		DFLL	GDSGSG	FHPG^SIAG	E(DEV))	S-PD	SP(1-23)	
**OfuSP39**	114353942	XM_028306167.1	1010		DVGL		STRR^IVSG	T(DGG)	PD	N	
**OfuSP40**	114354716	XM_028307252.1	313	SAAHC	DVAL		PDSR^IIGG	T(DGG)	S-PD	SP(1-18)	
**OfuSP41**	114354727	XM_028307271.1	272		DIAI		RIRR^IVNG	T(DGG)	S-PD	SP(1-26)	
**OfuSP42**	114354906	XM_028307499.1	230		DIGV	GDSGSA	/	C(GGD)	PD	N	
**OfuSP43**	114355117	XM_028307798.1	438	TAGHC	DVAI	GDSGGG	HHKH^ICGR	E(NGV)	S-PD	SP(1-20)	
**OfuSP44**	114355283	XM_028308048.1	266		DVAV		NGGK^IVGG	T(DGG)	S-PD	SP(1-23)	
**OfuSP45**	114355418	XM_028308251.1	269	SAAHC	DITV		AFHR^IIGG	T(DGG)	S-PD	SP(1-25)	
**OfuSP46**	114355419	XM_028308252.1	257	SAAHC	DITV		AAPR^IVGG	T(DGG)	S-PD	SP(1-18)	
**OfuSP47**	114355440	XM_028308280.1	527	SAAHC	DITV	GDGGGP	NPAR^IIGG		S-PLD-PD	SP(1-18)	
				SAAHC	DITV			T(DGG)			
**OfuSP48**	114355467	XM_028308315.1	275	SAAHC	DITV		ASNR^IVGG	T(DGG)	S-PD	SP(1-32)	
**OfuSP49**	114355864	XM_028308898.1	246		DIAV		ATAR^LVGG	C(GGD)	S-PD	SP(1-20)	

**OfuSP50**	114355959	XM_028309009.1	702	TAGHC	DYSL	HDSGGP	AVRR^IVGG	T(DGG)	S-PD	SP(1-27)	
**OfuSP51**	114357151	XM_028310652.1	277	TATHC	DIGV	GDSGSA	LNPH^IVGG	C(GGD)	S-PD	SP(1-19)	
**OfuSP52**	114357173	XM_028310676.1	275	TAGHC	DIAM		GETR^ITGG	C(GGT)	S-PD	SP(1-15)	
**OfuSP53**	114357179	XM_028310682.1	766	SAAHC	DIAI	GDSGGG	GDGL^ISGG	E (SAT)	S-7LDLA-2CCP-PD	SP(1-18)	
**OfuSP54**	114357180	XM_028310685.1	705	SAAHC	DIAI	GDSGGG	VKHS^LTSG	C(AAT)	S-6LDLA-2CCP-PD	SP(1-19)	
**OfuSP55**	114357183	XM_028310689.1	669	SAAHC	DIAI	GDSGGG	GVEL^VIGG	C(AAT)	S-5LDLA-CCP-PD	SP(1-17)	
**OfuSP56**	114357646	XM_028311362.1	265		DLSI		TSER^IIGG	T(DGG)	S-PD	SP(1-17)	
**OfuSP57**	114357731	XM_028311479.1	268		DIAI		GNNE^IVGG	T(DGG)	S-PD	SP(1-18)	
**OfuSP58**	114357879	XM_028311673.1	509	TAGHC	DVAV	GDSGGP	AQRR^IVGG	T(DGG)	S-PD	SP(1-25)	
**OfuSP59**	114358321	XM_028312256.1	385	TAGHC			FKHL^VIGG	T(DGG)	S-PD	SP(1-17)	
**OfuSP60**	114358647	XM_028312665.1	261	SAAHC	DIAV		LSAR^IVNG	T(DGG)	S-PD	SP(1-16)	
**OfuSP61**	114358690	XM_028312716.1	282		DVAI		NSTR^IVGG	E(NVH)	S-PD	SP(1-17)	
**OfuSP62**	114358715	XM_028312753.1	283		DVAI		NSSR^IVGG	?(NG？)	S-PD	SP(1-16)	
**OfuSP63**	114358725	XM_028312761.1	297		DVAI		DGSR^ITGG	C (SVA)	S-PD	SP(1-24)	
**OfuSP64**	114358723	XM_028312763.1	283		DIAI		DSSR^IVGG	C(NSA)	S-PD	SP(1-16)	
**OfuSP65**	114358734	XM_028312774.1	284		DIAI		NYTR^IVGG	C(NGA))	S-PD	SP(1-17)	
**OfuSP66**	114358765	XM_028312809.1	280		DVAV		DGGR^IWGG	C(SGA)	S-PD	SP(1-16)	
**OfuSP67**	114358766	XM_028312811.1	280		DVAV		DGGR^IWGG	C(SGA)	S-PD	SP(1-16)	
**OfuSP68**	114358773	XM_028312817.1	287		DVAI		FDGR^VAGG	C(SGA)	S-PD	SP(1-22)	
**OfuSP69**	114358796	XM_028312842.1	270	STAHC	DIAV		QFYR^VVGG	T(DGG)	S-PD	SP(1-20)	
**OfuSP70**	114359395	XM_028313786.1	240	TSAHC	DLAL	GDSGGS	LLLC^VFFG	?(???)	S-PD	SP(1-24)	
**OfuSP71**	114359765	XM_028314264.1	254		DVSL		QSPR^IVGG	C(GGD)	S-PD	SP(1-17)	

**OfuSP72**	114359887	XM_028314420.1	292		DIAI		ASGR^IIGG	C(SGA)	S-PD	SP(1-22)	
**OfuSP73**	114359977	XM_028314525.1	787				GPYY^AIGG	T(DGG)	S-PD-PD	SP(1-16)	
								T(DGG)			
**OfuSP74**	114360046	XM_028314605.1	283		DIAI		RDSR^ISLG	C(SGA)	S-PD	SP(1-18)	
**OfuSP75**	114360150	XM_028314731.1	411	SAAHC	DISL		DGSR^IVSG	C(SGG)	S-PD	SP(1-18)	
**OfuSP76**	114360153	XM_028314733.1	404	SAAHC	DISL		SDSR^IVSG	C(SGA)	S-PD	SP(1-18)	
**OfuSP77**	114360178	XM_028314757.1	451	TAGHC	DIAV			T(DGG)	PD-PD	N	
				SAGHC	DIAV			T(DGG)			
**OfuSP78**	114360246	XM_028314864.1	613	SAAHC	DVAL		EEPR^VVGG	T(DGG)	S-PD-PD	SP(1-30)	
								T(DGG)			
**OfuSP79**	114360247	XM_028314866.1	658				QENR^IVGG	T(DGG)	S-PD-PD	SP(1-19)	
								T(DGG)			
**OfuSP80**	114360409	XM_028315128.1	599	TTAHC	DAAI	GDSGAP	PDTR^IVGG	?(S?D)	S-PD-PLD	SP(1-19)	
				TAASC	DIAV	GDVGAP					
**OfuSP81**	114360793	XM_028315590.1	233	TAGHC	DIGL		PKFR^VIQG	?(???)	PD	N	
**OfuSP82**	114360913	XM_028315777.1	300				/	T(DGG)	S-PD	SP(1-16)	
**OfuSP83**	114361027	XM_028315938.1	272		DVAI		CLCE^AVAG	T(DGG)	S-PD	SP(1-15)	
**OfuSP84**	114361047	XM_028315964.1	300		DYQL		FKWR^IVGG	T(DGG)	S-PD	SP(1-18)	
**OfuSP85**	114361239	XM_028316233.1	424		DIAI	DDSGGP	KTNR^IVGG	T(DGG)	S-CUB-PD	SP(1-47)	
**OfuSP86**	114361464	XM_028316496.1	545		DLAI	GDSGGG	LNPL^IVNG	?(S?V)	S-Gd-PD	SP(1-19)	
**OfuSP87**	114361838	XM_028317038.1	332	TAGHC	DIAV		PPSL^VIGS	T(DGG)	PD	N	
**OfuSP88**	114362121	XM_028317390.1	282			GDSGSP	QVPR^IAYG	E(GIT)	S-PD	SP(1-22)	
**OfuSP89**	114362537	XM_028317983.1	298		DIAV		FEDR^VVGG	C (GSS)	S-PD	SP(1-17)	
**OfuSP90**	114362539	XM_028317985.1	298		DVAM		NIHR^IVGG	C (GAS)	S-PD	SP(1-17)	

**OfuSP91**	114362549	XM_028317996.1	1159		DVAM		FDNR^VVGG	C (GAS)	S-PD-PD-PD-PLD	SP(1-17)	
					DVAV			C (GAS)			
					DIAV			?(G??)			
					DLAL	GDNGGP					
**OfuSP92**	114363223	XM_028318889.1	346		DIGV		GAAR^IVSG	C (SVA)	S-PD	SP(1-18)	
**OfuSP93**	114363368	XM_028319057.1	467		DVAL		DMIR^IIGG	T(DGG)	2TSP-PD	N	
**OfuSP94**	114363402	XM_028319103.1	338				TRRR^IVGG	T(DGG)	S-PD	SP(1-17)	
**OfuSP95**	114363403	XM_028319106.1	265	TATHC	DIAV		RGAR^VVGG	T(DGG)	S-PD	SP(1-16)	
**OfuSP96**	114363650	XM_028319446.1	289	TAGHC	DVAV	GDSGGA	GSDR^IVGG	C(GGS)	S-PD	SP(1-16)	
**OfuSP97**	114363743	XM_028319559.1	618		DILM	GDSGGG	ECGV^IAGG	?(GG?)	S-Gd-PD	SP(1-18)	
**OfuSP98**	114364185	XM_028320207.1	324	SAAHC	DIGL		AALR^VIHG	E(QGT)	PD	N	
**OfuSP99**	114364244	XM_028320330.1	292		DIAV		ALGP^IVGG	C (GAS)	S-PD	SP(1-17)	
**OfuSP100**	114364377	XM_028320515.1	268				PTPR^VRDG	T (DGV)	S-PD	SP(1-18)	
**OfuSP101**	114365702	XM_028322335.1	283		DIAI		NSSR^VVGG	C(NGS)	S-PD	SP(1-16)	
**OfuSP102**	114365766	XM_028322427.1	1670	SASHC	DIAM		RWSR^VVGG	T(DGG)	TM-2LDLA-SR-PD-LDL	N	
**OfuSP103**	114365944	XM_028322672.1	349	SAAHC	DIAL	GDSGGP	RTGK^IVGG	T(DGG)	S-PD	SP(1-32)	
**OfuSP104**	114366233	XM_028323031.1	1873	SAAHC	DLSL		AQAR^VVGG	T(DGG)	2LDLA-PD-3LDLA-PLD-2LDL	N	
				VSHSC	NVAL	DGRDEP					
**OfuSP105**	114366773	XM_028323751.1	262	SAAHC	DITV		ASDR^IVGG	T(DGG)	S-PD	SP(1-19)	
**OfuSPH1**	114350078	XM_028300725.1	284	TGAHC	DVAV	GDTGGP	NEQR^VAGG		S-PLD	SP(1-17)	
**OfuSPH2**	114350616	XM_028301483.1	261	SSASC	DIAV	GDNGGP	GDGR^IAGG		S-PLD	SP(1-17)	

**OfuSPH3**	114350794	XM_028301726.1	271	TTASC		SDNGGP	PSKP^ALHK		S-PLD	SP(1-15)	
**OfuSPH4**	114351451	XM_028302673.1	254	TAASC	DIAM	GDTGGP	ADLR^IQGG		S-PLD	SP(1-17)	
**OfuSPH5**	114351938	XM_028303295.1	256	TAAYC	DISL	GDEGGP	PSSR^IIGG		S-PLD	SP(1-19)	
**OfuSPH6**	114351939	XM_028303296.1	256	TAAYC	DISL	GDEGGP	PSSR^IIGG		S-PLD	SP(1-19)	
**OfuSPH7**	114351954	XM_028303317.1	298	TSASC		FDLGSP	STGV^EIGR		S-PLD	SP(1-17)	
**OfuSPH8**	114352095	XM_028303555.1	486	TSAAC	DIAV	NDHGGP	SRRI^IVGS		PLD	N	
**OfuSPH9**	114352259	XM_028303785.1	268	SSAQC	DIAV	QDEGGP	ASQR^ISGG		S-PLD	SP(1-18)	
**OfuSPH10**	114353196	XM_028305075.1	313			GSGGSP	KPDF^SAPG		S-PLD	SP(1-21)	
**OfuSPH11**	114353667	XM_028305735.1	291	TSSHC	NLAV	ESEGGG	IRPR^QFYG		PLD	N	
**OfuSPH12**	114353859	XM_028305984.1	328	TVAHY		RDGGGP	KGEF^RIGG		S-PLD	SP(1-16)	
**OfuSPH13**	114354769	XM_028307360.1	473	TAPRC	ESLE		EAKP^LPPG		TM-PLD	N	
**OfuSPH14**	114355129	XM_028307816.1	356	TTAHY		GDGGSP	GVGF^MTTG		PLD	N	
**OfuSPH15**	114355420	XM_028308253.1	256	SAAHC	DITV	GDGGGP	NPAR^IIGG		S-PLD	SP(1-18)	
**OfuSPH16**	114355759	XM_028308773.1	500	TAARC	DLAV	PSYGGL	ALAE^VVENV		S-PLD	SP(1-19)	
**OfuSPH17**	114356034	XM_028309120.1	764	TASSC	NICI	HDVGGP	SGRR^IYRG		S-PLD	SP(1-20)	
**OfuSPH18**	114356038	XM_028309123.1	381		NFAV	HDAGGP	RYRR^VYNP		PLD	N	
**OfuSPH19**	114356039	XM_028309124.1	591	TSASC	NLAI	KDVGDP	INRR^IYNS		PLD	N	
**OfuSPH20**	114356061	XM_028309143.1	266	TAASS	NIGL	CDEGGP	TEDE^ILKG		S-PLD	SP(1-23)	
**OfuSPH21**	114356733	XM_028310071.1	738	TTGSC	MLAL	EEEYAG	RARS^GVEG		TM-Fz-2LDLA-PLD	N	
**OfuSPH22**	114357204	XM_028310716.1	383	SAAHC	DLAV	GDGGAG	MPIP^LDSG		S-PLD	SP(1-21)	
**OfuSPH23**	114357723	XM_028311473.1	1118		DLAI	GDGGGP	KTPS^YVDG		S-PLD	SP(1-20)	
**OfuSPH24**	114358357	XM_028312303.1	502	TTANC	DVAL	RDTGAP	EARR^LIAA		S-PLD	SP(1-18)	
**OfuSPH25**	114359801	XM_028314307.1	250			GKYQGR	VSSR^IVAG		S-PLD	SP(1-18)	

**OfuSPH26**	114360177	XM_028314756.1	279	TAASP		DDVGAP	LFDF^LLGR		S-PLD	SP(1-19)	
**OfuSPH27**	114360414	XM_028315130.1	697	TSAAC	NIAV	HDAGGP	DGRR^IFKG		PLD	N	
**OfuSPH28**	114360754	XM_028315545.1	320	TAAHY	DAAL	GDGGSA	CGTG^MVQG		S-PLD	SP(1-20)	
**OfuSPH29**	114364003	XM_028319979.1	225	AASNC	DVGL		/		PLD	N	
**OfuSPH30**	114364795	XM_028321098.1	329		DVAL	FDAGAP	GNLR^IRGG		PLD	N	
**OfuSPH31**	114365057	XM_028321509.1	257	TAALC	NLSI	GDTGGP	KIQR^IVGG		S-PLD	SP(1-16)	
**OfuSPH32**	114365314	XM_028321845.1	503	SVAHP		AVPGAP	LNRK^VFRA		PLD	N	
**OfuSPH33**	114366005	XM_028322764.1	265	TTAGV	DLAL	PDVGAP	FVEN^ITSN		PLD	N	
**OfuSPH34**	114366010	XM_028322767.1	274	SAAQC	NLAM	GDTGDP	KRLT^TKNG		PLD	N	

[a]: cSP, clip-domain serine protease; cSPH, clip-domain serine protease homolog; SP, serine protease; SPH, serine protease homolog.

[b]: When gene has more than one isoform, the longest amino acid sequence was selected.

[c]: not listed, sequences are identical to the conserved TAAHC, DIAL, or GDSGGP.

[d]: ^, putative activation cleavage site; /, no putative activation cleavage site; blank, no prediction.

[e]: Enzyme specificity was predicted based on the previous papers[3,4]. T, trypsin; C, chymotrypsin; E, elastase; ?, unpredictable type; blank, no prediction (SPH); letters in parentheses, amino acid residues determining the enzyme specificity of a serine protease.

[f] CCP, Sushi domain, also known as Sushi or SCR; CUB, a domain identified in complement 1r/s, uegf, and bmp1; Fz, frizzled domain; Gd, gastrulation defective; LDLA, LDLA-receptor class A domain; PD, serine protease domain; PLD, serine protease-like domain; S, signal peptide; SR, scavenger receptor domain; TM, transmembrane region; TSP, thrombospondin.

[g]: SP, signal peptide; N, no signal peptide detected or incomplete sequence.

[h]: Genes have been identified in the previous papers[1,2,5].

Supplementary Table S2. Gene counts for CLIPA-E genes in 6 insects.

	CLIPA	CLIPB	CLIPC	CLIPD	CLIPE
Drosophila melanogaster	16	18	7	11	
Apis mellifera	6	3	3	9	
Tribolium castaneum	16	17	10	11	
Manduca sexta	7	12	11	12	
Anopheles gambiae	22	29	12	14	33
Ostrinia furnilicas	8	11	4	15	

Supplementary Table S3. A summary of the orthogroups of CLIPs among five insect species. The amino acid sequences of CLIPs in *Ostrinia furnacalis*, *Apis mellifera, Manduca sexta*, *Drosophila melanogaster* and *Tribolium castaneum* have been analyzed via OrthoFinder2. Single-copy orthologs were marked in bold and *O. furnacalis*-specific orthologs were marked with underline.

Orthogroup	Category	*Apis mellifera*	*Drosophila melanogaster*	*Manduca sexta*	*Ostrinia furnacalis*	*Tribolium castaneum*
OG000000	CLIPA	AmcSPH19, AmcSPH37, AmcSPH39, AmcSPH42	DmcSPH101, DmcSPH121, DmcSPH242, DmcSPH35, DmcSPH58, DmcSPH66	MsSPH101, MsSPH1a, MsSPH1b, MsSPH2, MsSPH4	OfucSPH1, OfucSPH2, OfucSPH8	TccSPH1, TccSPH125, TccSPH2, TccSPH28, TccSPH29, TccSPH3, TccSPH30, TccSPH34, TccSPH4, TccSPH5, TccSPH59, TccSPH6, TccSPH78, TccSPH82
OG000001	CLIPB	AmcSP2	DmcSP10, DmcSP24/ea, DmcSP25/MP1, DmcSP4/SPE, DmcSP7/MP2	MsHP8, MsPAP1, MsPAP3	OfucSP10, OfucSP17, OfucSP18, OfucSP19, OfucSP23, OfucSP24, OfucSP25, OfucSP26, OfucSP9	TccSP136, TccSP137, TccSP8, TccSPH35
OG000002	CLIPC	AmcSP10, AmcSP14, AmcSP9	DmcSP28/Psh, DmcSP31/Hayan, DmcSP42, DmcSP48	MsHP28, MsHP6, MsSP30	OfucSP13, OfucSP14, OfucSP15	TccSP142, TccSP173, TccSP44, TccSP56, TccSP60, TccSP61, TccSP66
OG000003	CLIPC			MsHP13, MsHP18a, MsHP18b, MsHP2, MsHP21, MsHP22, MsSP144, MsSP33	OfucSP11	TccSP177, TccSPH174, TccSPH176
OG000004	CLIPB	AmcSP1	DmcSP5	MsGP6, MsHP5	OfucSP16	TccSP91, TccSP93, TccSP94
OG000005	CLIPD	AmcSP3	DmcSP18, DmcSP232	MsHP17a, MsHP17b	OfucSP4	TccSP140, TccSP55

OG000006	CLIPB			MsGP33, MsHP12, MsHP15, MsHP23, MsHP24, MsHP26, MsPAP2		
OG000007	CLIPD	AmcSP8	DmcSP34	MsHP1a, MsHP1b	OfucSP2	TccSP52
OG000008	CLIPB	AmcSPH50	DmcSP11, DmcSPH64	MsSPH42	OfucSPH5	TccSPH99
OG000009	CLIPD				OfucSP12, OfucSP20, OfucSP5, OfucSP6, OfucSP7, OfucSP8	
OG000010	CLIPD	**AmcSP16**	**DmcSP56/Sb**	**MsSP132**	**OfucSP29**	**TccSP126**
OG000011	CLIPD	**AmcSP21**	**DmcSP19**	**MsSP52**	**OfucSP3**	**TccSP172**
OG000012	CLIPD	**AmcSP25**	**DmcSP59/Np**	**MsSP143**	**OfucSP21**	**TccSP86**
OG000013	CLIPD	**AmcSP33**	**DmcSP44**	**MsSP142**	**OfucSP28**	**TccSP87**
OG000014	CLIPD	**AmcSP6a**	**DmcSP32**	**MsSP131**	**OfucSP27**	**TccSP85**
OG000015	CLIPD	**AmcSP7**	**DmcSP36**	**MsSP60**	**OfucSP1**	**TccSP53**
OG000016	CLIPA	**AmcSPH41**	**DmcSPH79/mas**	**MsSPH53**	**OfucSPH9**	**TccSPH51**
OG000017	CLIPB		DmcSP14, DmcSP16, DmcSP229, DmcSP38, DmcSP61			
OG000018	CLIPD	AmcSP26	DmcSP54	MsSP141		TccSP83
OG000019	CLIPA	AmcSPH55	DmcSPH142/scaf		OfucSPH7	TccSPH164
OG000020	CLIPD		DmcSP67	MsSP140	OfucSP22	TccSP84
OG000021	CLIPC		DmcSP115, DmcSP26/snk, DmcSP33/spirit			
OG000022	CLIPA		DmcSPH128, DmcSPH231, DmcSPH69			

OG000023	CLIPA		DmcSPH156, DmcSPH93			
OG000024	CLIPA				OfucSPH4, OfucSPH6	

Supplementary Table S4. A summary of RNA sequencing and mapping using the *Ostrinia furnacalis* genome as the reference. Columns represent number of raw sequencing reads, number of clean reads, number of clean bases, GC content, percentage of bases with Phred values score more than 20 or 30 to total bases, and ratio of sequences mapped to *O. furnacalis* genome. 1st instar larva, 3rd instar larva and 5th instar larva present the newly hatched larvae, middle stage larvae and mature larvae.

sample	Raw_reads	Clean_reads	Clean_bases	GC_pct	Q20	Q30	Total mapping	Unique mapping	Multi mapping
Embryo1	47277406	46310704	6.95G	44.1	97.69	93.15	38256078(82.61%)	36717941(79.29%)	1538137(3.32%)
Embryo2	47355624	46313542	6.95G	43.76	97.28	92.25	38197733(82.48%)	36616336(79.06%)	1581397(3.41%)
Embryo3	49942218	48912068	7.34G	44.54	97.71	93.15	40650345(83.11%)	38967590(79.67%)	1682755(3.44%)
1st instar Larva1	46462146	45336858	6.8G	43.38	97.81	93.45	39982187(88.19%)	37861182(83.51%)	2121005(4.68%)
1st instar Larva2	46429582	45007790	6.75G	45.11	97.68	93.16	39291447(87.3%)	37301506(82.88%)	1989941(4.42%)
1st instar Larva3	50764268	49526850	7.43G	42.74	97.87	93.57	43803520(88.44%)	41278642(83.35%)	2524878(5.1%)
3rd instar Larva1	46446916	44863872	6.73G	44.88	97.59	92.96	40161121(89.52%)	37213600(82.95%)	2947521(6.57%)
3rd instar Larva2	45464090	44328908	6.65G	47.26	97.51	92.87	38725027(87.36%)	36322552(81.94%)	2402475(5.42%)
3rd instar Larva3	44632178	41175430	6.18G	41.97	97.73	93.25	36015747(87.47%)	33777948(82.03%)	2237799(5.43%)
5th instar Larva1	47527840	46465472	6.97G	43.78	97.73	93.29	39787307(85.63%)	37789995(81.33%)	1997312(4.3%)
5th instar Larva2	48356108	46617192	6.99G	42.47	97.55	92.82	41341029(88.68%)	38695981(83.01%)	2645048(5.67%)
5th instar Larva3	47522106	46394770	6.96G	46.14	97.78	93.43	38881005(83.8%)	36424705(78.51%)	2456300(5.29%)
Pupa1	43211754	42189886	6.33G	45.26	97.5	92.78	34486836(81.74%)	32904576(77.99%)	1582260(3.75%)
Pupa2	47946958	46694826	7.0G	44.92	97.51	92.86	37926823(81.22%)	36124604(77.36%)	1802219(3.86%)
Pupa3	46345548	44824874	6.72G	45.93	97.66	93.24	36137237(80.62%)	34326578(76.58%)	1810659(4.04%)
Adult1	48569540	47214032	7.08G	40.1	97.47	92.74	38978285(82.56%)	36794927(77.93%)	2183358(4.62%)
Adult2	46839344	45480100	6.82G	40.03	97.54	92.84	38028291(83.62%)	36065664(79.3%)	1962627(4.32%)
Adult3	46603428	45277320	6.79G	37.87	97.52	92.79	36215372(79.99%)	33995939(75.08%)	2219433(4.9%)

Hemolymph1	45410452	44442508	6.67G	46.24	97.46	92.71	37167744(83.63%)	35469367(79.81%)	1698377(3.82%)
Hemolymph2	44377936	43459788	6.52G	45.66	97.72	93.25	36469390(83.92%)	34809719(80.1%)	1659671(3.82%)
Hemolymph3	45664808	44786468	6.72G	46.42	97.8	93.46	37471949(83.67%)	35661486(79.63%)	1810463(4.04%)
Midgut1	45303696	44286796	6.64G	45.14	97.6	93.05	36370403(82.12%)	34619411(78.17%)	1750992(3.95%)
Midgut2	46222664	45045852	6.76G	43.67	97.21	92.17	37154463(82.48%)	35439018(78.67%)	1715445(3.81%)
Midgut3	47721876	46714050	7.01G	45.71	97.52	92.89	38363116(82.12%)	36440786(78.01%)	1922330(4.12%)
Fat body1	47347014	46355232	6.95G	42.81	97.48	92.7	38965574(84.06%)	37114719(80.07%)	1850855(3.99%)
Fat body2	49525780	48552684	7.28G	45.43	97.76	93.34	42394426(87.32%)	40574532(83.57%)	1819894(3.75%)
Fat body3	48128642	46437806	6.97G	47.66	98.17	94.32	40334857(86.86%)	38707709(83.35%)	1627148(3.5%)
Silk gland 1	46389366	42182542	6.33G	51.08	96.57	91.37	30480855(72.26%)	27428346(65.02%)	3052509(7.24%)
Silk gland 2	46255644	43397546	6.51G	49.04	96.97	92	33089272(76.25%)	30646423(70.62%)	2442849(5.63%)
Silk gland 3	44002104	40919530	6.14G	49.39	96.55	91.09	30821961(75.32%)	28609764(69.92%)	2212197(5.41%)
Total or average	1404047036	1359515296	203.94G	44.75	97.53	92.90	37864980(83.48%)	35823385(78.96%)	2041595(4.52%)

Supplementary Table S5. Differentially expressed genes in pairwise comparison in different developmental stages (A) and different tissues (B) using DESeq2. $|log_2(ratio)| \geq 1$ & q-value < 0.05 were set as a threshold. M: midgut; SG: silk gland; H: hemolymph; FB: fat body; E: embryo; 1st L: new hatch larva; 3rd L: middle stage larva; 5th L: mature larva; P: pupa; A: adult.

(A)

E vs 1st L	E mean	1st L mean	log2(fc)	PValue	q-value[a]
OfuSP70	13640.967	0.001	-23.701	4.87E-14	5.99E-13
OfucSP20	1452.843	0.001	-20.470	1.25E-08	5.58E-08
OfuSPH33	766.773	0.001	-19.548	8.28E-04	1.95E-03
OfucSP23	114176.987	37.027	-11.590	1.26E-23	1.01E-21
OfucSP27	10439.360	20.293	-9.007	7.69E-10	4.56E-09
OfuSP79	9859.747	28.407	-8.439	3.25E-15	4.72E-14
OfucSP3	562.660	7.170	-6.294	5.08E-05	1.48E-04
OfucSPH9	1524.947	56.103	-4.765	2.33E-07	9.56E-07
OfuSP59	22906.040	1069.810	-4.420	4.88E-21	2.60E-19
OfuSPH34	593.367	31.027	-4.257	6.31E-03	1.16E-02
OfucSP29	12989.743	754.063	-4.107	1.80E-15	2.88E-14
OfucSP10	195245.803	13841.037	-3.818	7.68E-27	1.23E-24
OfucSP28	1245.423	100.913	-3.625	4.55E-06	1.55E-05
OfuSP55	22919.833	2122.140	-3.433	9.30E-14	1.06E-12
OfuSP86	66857.750	6314.143	-3.404	1.28E-16	2.92E-15
OfucSP21	1022.137	116.340	-3.135	5.00E-06	1.63E-05
OfuSP29	5074.203	644.107	-2.978	1.78E-06	6.48E-06
OfucSP17	86694.063	12053.513	-2.847	5.23E-18	2.09E-16
OfucSP4	23996.353	3374.810	-2.830	8.72E-10	4.84E-09
OfuSPH14	76076.600	11183.347	-2.766	1.30E-17	4.16E-16
OfucSP15	23375.193	4118.220	-2.505	3.91E-16	7.81E-15
OfucSP22	537.113	96.413	-2.478	2.57E-03	5.20E-03
OfucSP16	31368.190	6261.823	-2.325	8.77E-10	4.84E-09
OfuSP38	40618.650	8505.727	-2.256	1.23E-12	1.15E-11
OfuSP13	26556.350	6020.887	-2.141	2.04E-12	1.82E-11
OfucSP11	54573.250	18363.213	-1.571	5.48E-06	1.76E-05
OfucSPH8	96801.563	45784.637	-1.080	3.13E-06	1.11E-05
OfuSPH21	2377.633	1138.563	-1.062	3.16E-03	6.16E-03
OfucSP9	332.403	2335.077	2.813	4.99E-03	9.28E-03
OfucSP19	902.013	6662.657	2.885	3.07E-04	7.80E-04
OfuSP42	3490.167	29337.513	3.071	1.51E-03	3.31E-03
OfucSP2	162.047	1527.893	3.237	8.62E-03	1.52E-02
OfucSP26	50.973	509.593	3.322	2.95E-02	4.82E-02
OfuSP40	549.687	6503.683	3.565	3.32E-03	6.40E-03
OfucSPH5	51.387	613.873	3.579	8.07E-03	1.45E-02
OfuSP91	415.773	5255.373	3.660	9.26E-04	2.12E-03
OfucSP1	163.913	2476.420	3.917	7.77E-04	1.87E-03
OfuSP43	276.540	4349.033	3.975	2.69E-03	5.39E-03
OfuSP26	44.223	717.317	4.020	2.49E-03	5.10E-03
OfuSP14	234.510	3851.740	4.038	1.25E-03	2.79E-03
OfuSP21	28.653	488.553	4.092	2.08E-02	3.47E-02
OfuSP3	194.843	3824.200	4.295	1.11E-03	2.51E-03
OfuSP76	88.757	1915.390	4.432	5.89E-04	1.45E-03

OfucSP18	82.733	2123.303	4.682	6.47E-05	1.85E-04
OfuSP15	74.843	1997.500	4.738	1.43E-04	3.81E-04
OfuSP97	329.697	9649.920	4.871	3.40E-07	1.36E-06
OfuSPH28	37.370	1487.190	5.315	1.26E-04	3.41E-04
OfucSP24	36.127	1451.390	5.328	8.62E-04	2.00E-03
OfuSP17	7.577	315.583	5.380	3.45E-03	6.56E-03
OfuSP61	7.163	300.253	5.389	3.58E-03	6.74E-03
OfuSP4	44.947	1924.887	5.420	1.98E-05	5.86E-05
OfuSP87	22.320	1446.337	6.018	6.44E-06	2.02E-05
OfuSP80	15.157	1044.573	6.107	2.63E-04	6.90E-04
OfuSP95	7.163	720.917	6.653	7.83E-04	1.87E-03
OfuSPH16	45.467	5281.187	6.860	1.55E-05	4.69E-05
OfuSP22	36.957	4877.743	7.044	2.20E-09	1.14E-08
OfucSPH4	14.950	2386.633	7.319	7.24E-06	2.23E-05
OfuSP19	7.163	1285.770	7.488	7.52E-05	2.11E-04
OfuSP66	14.637	2678.217	7.516	4.41E-06	1.54E-05
OfuSP64	21.903	4119.107	7.555	1.23E-07	5.16E-07
OfuSP101	22.733	4321.473	7.571	1.24E-06	4.60E-06
OfucSPH2	29.277	6461.673	7.786	2.46E-10	1.51E-09
OfucSPH7	416.373	114993.677	8.110	1.65E-11	1.20E-10
OfuSPH31	29.067	10914.980	8.553	2.77E-09	1.38E-08
OfuSPH2	7.473	4322.567	9.176	5.43E-08	2.35E-07
OfuSP85	22.320	15300.950	9.421	3.96E-12	3.34E-11
OfuSP73	30.000	23406.600	9.608	8.49E-17	2.26E-15
OfuSP2	7.163	6792.140	9.889	9.65E-09	4.54E-08
OfuSP45	21.490	20536.453	9.900	3.55E-11	2.47E-10
OfuSP47	28.963	28304.687	9.933	1.43E-11	1.14E-10
OfucSPH3	7.163	7600.360	10.051	1.07E-09	5.72E-09
OfuSP18	95.193	140766.637	10.530	2.02E-13	2.16E-12
OfuSP72	22.320	33676.610	10.559	7.62E-11	5.08E-10
OfuSP105	14.740	29773.500	10.980	1.01E-12	1.01E-11
OfuSP67	21.490	48246.610	11.133	4.58E-14	5.99E-13
OfuSPH6	15.050	46270.053	11.586	1.40E-15	2.48E-14
OfuSP23	0.001	138.973	17.084	1.48E-02	2.49E-02
OfuSP48	0.001	159.320	17.282	1.13E-02	1.94E-02
OfuSP41	0.001	253.483	17.952	2.44E-03	5.07E-03
OfuSP60	0.001	276.260	18.076	2.39E-03	5.03E-03
OfuSP20	0.001	311.843	18.251	1.60E-03	3.46E-03
OfucSP6	0.001	354.350	18.435	8.38E-03	1.49E-02
OfucSP8	0.001	395.657	18.594	1.67E-03	3.56E-03
OfuSPH1	0.001	419.923	18.680	2.72E-04	7.03E-04
OfucSP5	0.001	430.237	18.715	5.23E-04	1.31E-03
OfuSP99	0.001	664.967	19.343	2.97E-03	5.87E-03
OfuSP65	0.001	720.677	19.459	8.99E-05	2.48E-04
OfuSP1	0.001	1674.107	20.675	4.82E-06	1.61E-05
OfuSP68	0.001	2961.900	21.498	7.38E-09	3.58E-08
OfuSP62	0.001	3059.687	21.545	1.18E-06	4.51E-06
OfuSP46	0.001	4408.917	22.072	4.48E-07	1.75E-06
OfuSP16	0.001	4581.413	22.127	1.24E-08	5.58E-08
OfuSP63	0.001	5959.900	22.507	1.62E-11	1.20E-10
OfuSPH9	0.001	11045.413	23.397	1.65E-10	1.05E-09
E vs 3rd L	E mean	3rd L mean	log2(fc)	PValue	q-value
OfuSPH33	766.773	0.001	-19.548	1.40E-03	3.02E-03
OfucSP22	537.113	0.001	-19.035	3.56E-05	1.19E-04

OfucSP27	10439.360	2.400	-12.087	5.49E-09	5.76E-08
OfucSPH9	1524.947	3.600	-8.727	3.61E-07	1.97E-06
OfuSP70	13640.967	36.980	-8.527	2.78E-08	2.41E-07
OfuSP29	5074.203	14.043	-8.497	6.36E-12	1.34E-10
OfucSP23	114176.987	341.047	-8.387	3.82E-30	5.61E-28
OfuSP58	361.783	1.200	-8.236	1.47E-05	5.52E-05
OfuSP79	9859.747	45.337	-7.765	3.42E-15	1.00E-13
OfuSP59	22906.040	292.273	-6.292	1.93E-15	7.09E-14
OfucSP29	12989.743	182.540	-6.153	1.41E-20	1.04E-18
OfucSP28	1245.423	24.487	-5.669	1.79E-07	1.10E-06
OfucSP3	562.660	30.133	-4.223	6.00E-05	1.80E-04
OfucSP10	195245.803	12442.747	-3.972	2.75E-10	4.04E-09
OfuSP86	66857.750	8173.307	-3.032	5.23E-11	9.61E-10
OfuSP13	26556.350	3311.267	-3.004	5.42E-06	2.21E-05
OfuSP55	22919.833	3540.977	-2.694	2.71E-07	1.53E-06
OfucSP4	23996.353	4857.137	-2.305	1.93E-04	5.06E-04
OfucSP21	1022.137	210.070	-2.283	6.11E-04	1.50E-03
OfucSP17	86694.063	18551.583	-2.224	1.62E-05	5.97E-05
OfuSPH14	76076.600	17172.583	-2.147	5.13E-07	2.60E-06
OfuSPH17	494.660	117.413	-2.075	3.21E-03	6.83E-03
OfuSP38	40618.650	9643.813	-2.075	4.79E-05	1.53E-04
OfucSP16	31368.190	9358.627	-1.745	2.42E-04	6.25E-04
OfucSP15	23375.193	7647.827	-1.612	6.52E-04	1.54E-03
OfuSPH21	2377.633	895.027	-1.410	1.12E-02	2.23E-02
OfucSP11	54573.250	20652.360	-1.402	8.11E-03	1.63E-02
OfuSP54	489.997	3559.043	2.861	1.81E-02	3.41E-02
OfuSP53	1046.647	12380.103	3.564	1.99E-02	3.66E-02
OfucSP19	902.013	11026.877	3.612	1.37E-04	3.67E-04
OfuSPH22	262.423	3520.230	3.746	1.51E-02	2.93E-02
OfuSP75	66.127	1009.830	3.933	1.64E-02	3.14E-02
OfuSPH7	515.520	9167.953	4.153	5.20E-03	1.08E-02
OfuSP26	44.223	816.277	4.206	1.32E-02	2.58E-02
OfuSPH10	695.970	13660.547	4.295	1.91E-02	3.56E-02
OfuSP97	329.697	6800.897	4.367	1.37E-04	3.67E-04
OfuSP43	276.540	6129.040	4.470	5.47E-03	1.12E-02
OfucSP12	192.257	4694.397	4.610	6.47E-04	1.54E-03
OfucSP2	162.047	3966.450	4.613	1.14E-03	2.51E-03
OfucSP9	332.403	9212.620	4.793	1.08E-07	7.57E-07
OfuSP76	88.757	2562.790	4.852	1.04E-03	2.38E-03
OfuSP64	21.903	656.670	4.906	2.29E-02	4.11E-02
OfuSP37	146.793	4895.023	5.060	5.35E-04	1.33E-03
OfucSP18	82.733	2816.807	5.089	4.26E-04	1.08E-03
OfucSP1	163.913	5966.517	5.186	4.35E-05	1.42E-04
OfuSP80	15.157	848.337	5.807	2.21E-02	4.01E-02
OfuSP3	194.843	15738.990	6.336	1.01E-04	2.90E-04
OfuSPH28	37.370	3297.120	6.463	2.25E-05	7.89E-05
OfuSPH31	29.067	3362.970	6.854	2.00E-05	7.17E-05
OfucSP26	50.973	6363.850	6.964	5.02E-07	2.60E-06
OfucSPH6	7.577	975.500	7.008	1.30E-04	3.61E-04
OfuSPH12	60.620	7876.907	7.022	1.08E-03	2.40E-03
OfuSP101	22.733	3126.437	7.104	5.38E-05	1.65E-04
OfucSP24	36.127	5068.893	7.133	6.38E-07	3.13E-06
OfuSPH16	45.467	6433.903	7.145	1.05E-03	2.38E-03
OfuSP87	22.320	3798.873	7.411	1.78E-07	1.10E-06

OfuSPH26	195.897	35249.360	7.491	1.23E-06	5.85E-06
OfucSP7	104.230	19842.007	7.573	4.74E-09	5.36E-08
OfucSPH7	416.373	91765.207	7.784	3.46E-08	2.83E-07
OfucSPH4	14.950	3874.387	8.018	3.56E-06	1.54E-05
OfuSPH6	15.050	4224.713	8.133	8.18E-05	2.40E-04
OfuSPH2	7.473	2525.967	8.401	1.29E-05	4.99E-05
OfuSP85	22.320	8422.173	8.560	9.30E-11	1.52E-09
OfuSP47	28.963	12121.963	8.709	5.79E-08	4.48E-07
OfuSP73	30.000	27021.620	9.815	5.99E-12	1.34E-10
OfuSP67	21.490	20431.087	9.893	8.57E-08	6.30E-07
OfuSP18	95.193	108814.987	10.159	9.55E-09	8.77E-08
OfucSPH3	7.163	10970.307	10.581	3.85E-09	4.71E-08
OfucSPH2	29.277	48249.507	10.687	6.29E-16	3.08E-14
OfuSP2	7.163	20362.797	11.473	1.27E-07	8.46E-07
OfuSP60	0.001	305.833	18.222	2.34E-02	4.15E-02
OfuSP65	0.001	484.967	18.888	7.39E-04	1.72E-03
OfuSP1	0.001	846.937	19.692	5.17E-03	1.08E-02
OfuSP41	0.001	883.730	19.753	5.20E-05	1.63E-04
OfucSP8	0.001	1536.540	20.551	4.16E-06	1.75E-05
OfuSP68	0.001	1803.327	20.782	1.02E-04	2.90E-04
OfucSP6	0.001	2247.290	21.100	3.29E-06	1.47E-05
OfuSP16	0.001	2623.013	21.323	1.17E-05	4.63E-05
OfuSPH5	0.001	3223.293	21.620	3.30E-05	1.13E-04
OfuSP46	0.001	5091.167	22.280	2.45E-06	1.12E-05
OfuSPH9	0.001	6413.070	22.613	8.80E-09	8.62E-08
OfuSP74	0.001	8822.163	23.073	2.39E-07	1.41E-06
OfucSP5	0.001	9168.890	23.128	2.62E-09	3.50E-08
E vs 5th L	E mean	5th L mean	log2(fc)	PValue	q-value
OfuSPH33	766.773	0.001	-19.548	2.03E-02	3.25E-02
OfuSP70	13640.967	2.347	-12.505	8.69E-16	1.32E-14
OfucSP27	10439.360	2.347	-12.119	5.03E-12	3.23E-11
OfuSP29	5074.203	16.460	-8.268	1.18E-13	1.03E-12
OfuSP59	22906.040	158.570	-7.175	1.60E-15	2.23E-14
OfucSP29	12989.743	162.903	-6.317	5.01E-21	1.19E-19
OfucSP23	114176.987	2167.963	-5.719	3.08E-14	2.86E-13
OfucSP10	195245.803	8053.973	-4.599	1.33E-24	7.43E-23
OfucSPH9	1524.947	86.057	-4.147	3.77E-08	1.17E-07
OfucSP28	1245.423	75.410	-4.046	5.75E-06	1.35E-05
OfuSPH19	111.073	7.280	-3.931	2.40E-02	3.78E-02
OfucSPH1	562.130	38.563	-3.866	7.00E-03	1.18E-02
OfuSP55	22919.833	2281.013	-3.329	2.88E-09	1.20E-08
OfuSP58	361.783	36.153	-3.323	9.44E-04	1.79E-03
OfucSP22	537.113	54.027	-3.314	1.34E-02	2.20E-02
OfuSPH21	2377.633	277.870	-3.097	2.73E-05	6.08E-05
OfuSP86	66857.750	8729.437	-2.937	3.40E-09	1.38E-08
OfucSP16	31368.190	4365.567	-2.845	8.26E-03	1.37E-02
OfuSP13	26556.350	3805.807	-2.803	1.52E-06	3.79E-06
OfuSPH14	76076.600	11289.987	-2.752	1.06E-06	2.73E-06
OfucSP3	562.660	84.290	-2.739	8.22E-03	1.37E-02
OfucSP17	86694.063	13889.330	-2.642	1.29E-08	4.32E-08
OfucSP4	23996.353	3887.843	-2.626	1.35E-06	3.42E-06
OfuSP30	155.403	25.257	-2.621	1.78E-02	2.89E-02
OfuSPH17	494.660	100.490	-2.299	1.52E-03	2.75E-03
OfuSP24	2850.187	690.177	-2.046	3.16E-02	4.79E-02

OfucSP15	23375.193	6534.943	-1.839	2.62E-04	5.40E-04
OfuSPH13	667.190	199.933	-1.739	3.13E-02	4.79E-02
OfuSP38	40618.650	14395.043	-1.497	5.11E-03	8.98E-03
OfucSP14	1317.180	5856.893	2.153	6.07E-04	1.19E-03
OfuSPH11	73.500	357.730	2.283	2.36E-02	3.75E-02
OfuSP91	415.773	2059.890	2.309	4.75E-03	8.44E-03
OfuSPH20	96.543	517.270	2.422	6.43E-03	1.09E-02
OfuSP40	549.687	3132.920	2.511	6.17E-03	1.06E-02
OfuSP54	489.997	2863.667	2.547	2.60E-04	5.40E-04
OfuSP77	22.320	187.857	3.073	2.86E-02	4.46E-02
OfucSP12	192.257	1730.457	3.170	3.35E-04	6.82E-04
OfuSP75	66.127	618.923	3.227	1.92E-03	3.45E-03
OfuSP43	276.540	2672.733	3.273	1.04E-03	1.94E-03
OfuSP35	14.327	139.137	3.280	2.99E-02	4.63E-02
OfuSP42	3490.167	36530.390	3.388	1.78E-07	4.87E-07
OfuSP76	88.757	1004.647	3.501	1.09E-04	2.30E-04
OfuSP53	1046.647	14100.977	3.752	6.78E-04	1.32E-03
OfucSP19	902.013	12592.173	3.803	9.53E-13	7.23E-12
OfuSP97	329.697	5579.150	4.081	4.89E-08	1.46E-07
OfucSP9	332.403	5980.903	4.169	3.84E-08	1.17E-07
OfuSP33	7.577	152.647	4.333	6.07E-03	1.06E-02
OfuSP37	146.793	3240.530	4.464	6.21E-05	1.33E-04
OfuSPH16	45.467	1037.967	4.513	4.28E-04	8.61E-04
OfucSP2	162.047	3752.753	4.534	3.68E-06	8.77E-06
OfuSPH22	262.423	6596.690	4.652	1.28E-07	3.76E-07
OfuSP64	21.903	652.063	4.896	1.96E-05	4.43E-05
OfuSP32	96.023	3593.537	5.226	3.15E-07	8.36E-07
OfuSP80	15.157	568.417	5.229	4.98E-05	1.08E-04
OfuSPH10	695.970	26155.297	5.232	4.12E-05	9.04E-05
OfucSP7	104.230	3973.403	5.253	1.88E-08	6.17E-08
OfuSPH7	515.520	20607.247	5.321	6.54E-10	3.04E-09
OfuSP4	44.947	2728.923	5.924	9.65E-10	4.24E-09
OfuSP87	22.320	1589.593	6.154	6.08E-09	2.36E-08
OfucSP26	50.973	3691.913	6.179	9.16E-09	3.40E-08
OfucSP1	163.913	12915.270	6.300	4.27E-12	2.85E-11
OfuSP105	14.740	1196.500	6.343	1.71E-07	4.75E-07
OfucSPH7	416.373	34444.727	6.370	3.48E-09	1.39E-08
OfuSPH6	15.050	1277.527	6.407	5.99E-06	1.39E-05
OfucSP18	82.733	7581.293	6.518	2.22E-14	2.18E-13
OfuSPH31	29.067	2893.243	6.637	1.98E-10	1.07E-09
OfuSPH26	195.897	19783.267	6.658	2.11E-09	9.03E-09
OfuSP3	194.843	20605.447	6.725	3.98E-12	2.77E-11
OfuSPH28	37.370	4307.083	6.849	8.57E-12	5.30E-11
OfuSPH12	60.620	7384.323	6.929	3.58E-08	1.13E-07
OfuSP47	28.963	3580.423	6.950	5.96E-11	3.43E-10
OfucSP24	36.127	4923.113	7.090	2.47E-10	1.29E-09
OfuSPH15	22.423	4154.337	7.534	9.75E-09	3.54E-08
OfuSP89	14.327	2902.260	7.662	6.43E-09	2.44E-08
OfucSPH6	7.577	1559.450	7.685	3.14E-07	8.36E-07
OfuSP45	21.490	5790.667	8.074	2.62E-10	1.29E-09
OfuSPH2	7.473	2316.937	8.276	2.05E-08	6.58E-08
OfucSPH4	14.950	4711.363	8.300	1.19E-08	4.06E-08
OfuSP72	22.320	9249.720	8.695	2.55E-10	1.29E-09
OfuSP101	22.733	9509.967	8.709	1.08E-08	3.82E-08

OfuSP22	36.957	19052.433	9.010	1.07E-17	2.23E-16
OfuSP73	30.000	18623.403	9.278	2.77E-22	9.25E-21
OfuSP85	22.320	14554.747	9.349	2.80E-16	5.20E-15
OfuSP92	14.740	12076.037	9.678	7.52E-15	8.38E-14
OfucSPH2	29.277	37963.290	10.341	5.47E-24	2.28E-22
OfuSP18	95.193	131409.550	10.431	2.68E-27	4.48E-25
OfucSPH3	7.163	14360.453	10.969	5.03E-15	6.00E-14
OfuSP2	7.163	22373.287	11.609	7.31E-16	1.22E-14
OfuSP66	14.637	46621.987	11.637	2.12E-21	5.89E-20
OfuSP67	21.490	74613.490	11.762	5.95E-25	4.97E-23
OfuSP5	0.001	173.160	17.402	1.11E-03	2.04E-03
OfuSP6	0.001	175.723	17.423	1.05E-03	1.94E-03
OfuSP99	0.001	186.833	17.511	5.73E-04	1.14E-03
OfuSPH9	0.001	189.893	17.535	7.28E-04	1.40E-03
OfuSP60	0.001	387.853	18.565	1.74E-05	3.98E-05
OfuSP62	0.001	1072.877	20.033	1.94E-06	4.77E-06
OfuSP65	0.001	1249.380	20.253	1.59E-07	4.49E-07
OfuSP1	0.001	1395.077	20.412	1.43E-07	4.13E-07
OfucSP8	0.001	1398.727	20.416	1.13E-08	3.94E-08
OfuSP41	0.001	1518.070	20.534	3.84E-07	1.00E-06
OfuSP78	0.001	1649.183	20.653	2.70E-06	6.54E-06
OfucSP6	0.001	2141.050	21.030	2.80E-10	1.33E-09
OfuSP46	0.001	2456.053	21.228	6.76E-10	3.05E-09
OfuSPH1	0.001	3797.547	21.857	2.24E-12	1.63E-11
OfuSP16	0.001	4337.413	22.048	1.12E-10	6.22E-10
OfucSP5	0.001	4344.947	22.051	8.32E-13	6.62E-12
OfuSPH5	0.001	5807.440	22.470	5.23E-13	4.36E-12
OfuSP68	0.001	6555.960	22.644	1.88E-14	1.96E-13
OfuSP74	0.001	9229.273	23.138	1.29E-11	7.72E-11
OfuSP63	0.001	10543.300	23.330	2.55E-15	3.28E-14
E vs P	E mean	P mean	log2(fc)	PValue	q-value
OfuSP70	13640.967	0.001	-23.701	2.35E-17	3.09E-16
OfucSP23	114176.987	50.230	-11.150	4.27E-53	7.30E-51
OfuSP29	5074.203	64.790	-6.291	2.63E-11	1.73E-10
OfucSP27	10439.360	180.947	-5.850	3.34E-06	1.21E-05
OfuSP79	9859.747	217.057	-5.505	5.32E-14	4.79E-13
OfucSP20	1452.843	35.730	-5.346	8.01E-08	3.70E-07
OfuSP59	22906.040	647.263	-5.145	4.54E-13	3.24E-12
OfucSP29	12989.743	910.853	-3.834	7.48E-06	2.51E-05
OfucSP10	195245.803	14088.443	-3.793	5.26E-09	2.73E-08
OfucSP4	23996.353	2072.287	-3.534	1.74E-06	6.66E-06
OfuSPH25	756.983	71.427	-3.406	3.69E-05	1.09E-04
OfuSP55	22919.833	3000.527	-2.933	2.03E-07	8.91E-07
OfuSP42	3490.167	497.817	-2.810	1.57E-03	3.63E-03
OfuSP24	2850.187	453.820	-2.651	2.28E-02	4.06E-02
OfuSPH14	76076.600	12121.967	-2.650	2.16E-04	5.69E-04
OfuSP13	26556.350	4267.327	-2.638	2.25E-05	6.86E-05
OfuSPH13	667.190	112.893	-2.563	2.05E-03	4.43E-03
OfuSP86	66857.750	11341.287	-2.560	2.53E-05	7.58E-05
OfucSP17	86694.063	18980.683	-2.191	2.48E-04	6.43E-04
OfucSP16	31368.190	7182.683	-2.127	3.60E-03	7.43E-03
OfucSP13	2805.370	5798.850	1.048	1.56E-02	2.86E-02
OfucSP14	1317.180	4473.680	1.764	3.50E-03	7.31E-03
OfuSP102	6291.897	21531.913	1.775	2.97E-03	6.27E-03

OfuSP3	194.843	689.040	1.822	1.37E-02	2.57E-02
OfuSPH26	195.897	1112.620	2.506	1.46E-03	3.43E-03
OfucSP7	104.230	645.183	2.630	5.58E-03	1.12E-02
OfuSP4	44.947	291.093	2.695	1.49E-02	2.78E-02
OfucSP21	1022.137	7464.457	2.868	7.21E-07	2.94E-06
OfuSP77	22.320	176.643	2.984	6.64E-03	1.29E-02
OfuSP83	29.067	239.700	3.044	1.79E-03	4.03E-03
OfuSP22	36.957	312.300	3.079	8.39E-04	2.05E-03
OfuSP40	549.687	4669.520	3.087	7.82E-04	1.94E-03
OfuSPH11	73.500	641.900	3.127	1.39E-03	3.30E-03
OfuSPH31	29.067	255.793	3.138	5.15E-03	1.05E-02
OfuSP53	1046.647	12572.410	3.586	7.16E-06	2.45E-05
OfuSP31	609.373	7761.293	3.671	3.61E-06	1.29E-05
OfuSP54	489.997	6641.897	3.761	1.44E-10	7.97E-10
OfuSP97	329.697	4942.570	3.906	1.95E-08	9.83E-08
OfuSP34	21.800	330.897	3.924	2.04E-04	5.44E-04
OfuSP52	15.050	230.957	3.940	1.65E-03	3.77E-03
OfuSP92	14.740	245.210	4.056	2.02E-03	4.42E-03
OfuSP96	7.163	134.593	4.232	1.96E-02	3.57E-02
OfuSP35	14.327	301.947	4.398	2.79E-04	7.12E-04
OfuSPH2	7.473	157.710	4.399	2.34E-03	5.01E-03
OfucSP2	162.047	3520.243	4.441	9.53E-13	6.52E-12
OfuSP101	22.733	530.843	4.545	9.70E-05	2.76E-04
OfucSP19	902.013	22413.407	4.635	3.93E-26	1.12E-24
OfuSP95	7.163	195.767	4.772	1.16E-03	2.79E-03
OfucSPH7	416.373	12084.850	4.859	8.45E-11	4.81E-10
OfuSP85	22.320	648.813	4.861	1.28E-05	4.20E-05
OfuSPH32	7.577	225.387	4.895	4.09E-04	1.03E-03
OfuSP18	95.193	2942.440	4.950	6.81E-08	3.33E-07
OfuSPH10	695.970	22651.160	5.024	4.53E-07	1.89E-06
OfuSP43	276.540	10303.477	5.220	2.30E-10	1.23E-09
OfuSPH22	262.423	10092.143	5.265	3.95E-13	2.94E-12
OfuSPH6	15.050	746.050	5.631	1.23E-04	3.45E-04
OfuSP33	7.577	438.103	5.854	2.13E-05	6.61E-05
OfuSP66	14.637	878.867	5.908	2.20E-06	8.17E-06
OfuSP32	96.023	6668.170	6.118	2.31E-13	1.79E-12
OfuSP94	37.473	2625.617	6.131	6.84E-06	2.39E-05
OfuSP2	7.163	526.977	6.201	1.68E-05	5.34E-05
OfucSP18	82.733	6501.607	6.296	3.06E-17	3.73E-16
OfucSP9	332.403	26708.967	6.328	2.34E-36	1.33E-34
OfuSP87	22.320	1858.680	6.380	6.39E-11	3.90E-10
OfuSP37	146.793	13337.533	6.506	4.52E-16	5.15E-15
OfuSP67	21.490	2334.023	6.763	4.48E-11	2.84E-10
OfuSPH28	37.370	5461.693	7.191	1.46E-15	1.47E-14
OfucSP1	163.913	33191.880	7.662	1.89E-34	8.09E-33
OfucSPH2	29.277	6906.503	7.882	1.44E-15	1.47E-14
OfuSP21	28.653	8149.347	8.152	7.52E-14	6.12E-13
OfuSP72	22.320	8276.037	8.535	1.13E-14	1.08E-13
OfucSPH6	7.577	2872.307	8.566	7.43E-11	4.38E-10
OfuSPH12	60.620	30206.607	8.961	4.01E-19	6.85E-18
OfuSP73	30.000	24135.217	9.652	1.92E-24	4.10E-23
OfucSP24	36.127	30147.890	9.705	9.78E-27	3.34E-25
OfucSPH4	14.950	32491.183	11.086	1.71E-18	2.46E-17
OfuSP26	44.223	179489.810	11.987	3.42E-45	2.92E-43

OfucSPH3	7.163	71444.190	13.284	1.90E-22	3.60E-21
OfuSP71	7.163	120628.933	14.040	7.11E-25	1.74E-23
OfuSPH9	0.001	72.963	16.155	1.37E-02	2.57E-02
OfucSP8	0.001	82.763	16.337	8.20E-03	1.58E-02
OfuSP16	0.001	84.263	16.363	2.22E-02	3.99E-02
OfuSP1	0.001	112.693	16.782	6.03E-03	1.19E-02
OfuSP65	0.001	124.987	16.931	5.88E-03	1.17E-02
OfuSPH1	0.001	155.257	17.244	1.88E-03	4.18E-03
OfuSP100	0.001	189.547	17.532	1.91E-04	5.18E-04
OfuSP104	0.001	241.873	17.884	1.64E-04	4.53E-04
OfuSP60	0.001	256.487	17.969	5.75E-05	1.67E-04
OfucSP5	0.001	295.407	18.172	1.58E-05	5.09E-05
OfuSP63	0.001	404.820	18.627	1.75E-06	6.66E-06
OfuSP6	0.001	435.217	18.731	8.04E-07	3.20E-06
OfuSP5	0.001	580.583	19.147	9.52E-08	4.28E-07
OfuSPH5	0.001	1016.863	19.956	7.94E-08	3.70E-07
OfuSP62	0.001	1205.333	20.201	4.42E-07	1.89E-06
OfuSP74	0.001	8764.983	23.063	5.92E-14	5.06E-13
OfuSP78	0.001	12221.897	23.543	1.73E-18	2.46E-17
E vs A	E mean	A mean	log2(fc)	PValue	q-value
OfucSP27	10439.360	0.001	-23.316	7.76E-12	4.20E-11
OfucSP12	192.257	0.001	-17.553	8.89E-04	1.74E-03
OfucSPH9	1524.947	5.063	-8.235	2.02E-08	6.41E-08
OfucSP20	1452.843	15.190	-6.580	3.79E-07	1.04E-06
OfucSP23	114176.987	1610.907	-6.147	1.68E-31	2.83E-29
OfucSP28	1245.423	19.647	-5.986	1.74E-08	5.63E-08
OfucSP4	23996.353	392.620	-5.934	4.40E-18	4.35E-17
OfuSP55	22919.833	426.260	-5.749	8.42E-27	2.83E-25
OfuSP86	66857.750	1250.100	-5.741	5.02E-25	1.05E-23
OfucSP29	12989.743	324.297	-5.324	9.76E-16	6.56E-15
OfuSP79	9859.747	252.653	-5.286	1.18E-11	5.99E-11
OfuSP29	5074.203	157.457	-5.010	6.76E-06	1.62E-05
OfuSP58	361.783	20.250	-4.159	1.18E-03	2.22E-03
OfucSP3	562.660	31.630	-4.153	2.25E-04	4.73E-04
OfuSPH14	76076.600	5435.157	-3.807	3.90E-10	1.49E-09
OfucSP10	195245.803	14356.293	-3.766	3.58E-14	2.15E-13
OfuSPH7	515.520	39.287	-3.714	3.21E-03	5.74E-03
OfucSP21	1022.137	84.200	-3.602	6.43E-07	1.66E-06
OfucSP15	23375.193	3258.327	-2.843	6.16E-09	2.11E-08
OfuSP38	40618.650	5894.027	-2.785	8.23E-07	2.09E-06
OfuSPH25	756.983	112.927	-2.745	2.35E-04	4.87E-04
OfucSP17	86694.063	14822.917	-2.548	9.05E-10	3.38E-09
OfuSP13	26556.350	6294.910	-2.077	3.46E-06	8.55E-06
OfucSP14	1317.180	339.300	-1.957	5.01E-03	8.59E-03
OfucSP13	2805.370	795.800	-1.818	9.49E-04	1.83E-03
OfuSP59	22906.040	6937.603	-1.723	6.99E-03	1.17E-02
OfucSPH8	96801.563	44445.413	-1.123	3.00E-02	4.85E-02
OfuSPH22	262.423	1024.893	1.966	1.53E-02	2.52E-02
OfuSP31	609.373	2382.237	1.967	2.62E-03	4.84E-03
OfuSP40	549.687	2166.363	1.979	1.98E-02	3.23E-02
OfuSP54	489.997	2344.977	2.259	2.07E-04	4.46E-04
OfuSP53	1046.647	5340.080	2.351	1.29E-02	2.15E-02
OfucSP18	82.733	504.220	2.608	3.51E-03	6.21E-03
OfucSP26	50.973	404.160	2.987	1.29E-03	2.41E-03

OfucSPH7	416.373	3349.103	3.008	6.82E-04	1.36E-03
OfucSP19	902.013	7936.903	3.137	3.39E-08	1.03E-07
OfuSPH10	695.970	7538.287	3.437	4.07E-03	7.13E-03
OfucSP2	162.047	1900.580	3.552	2.07E-05	4.84E-05
OfuSPH17	494.660	6098.843	3.624	3.46E-09	1.21E-08
OfuSP30	155.403	2164.840	3.800	2.15E-07	6.03E-07
OfuSPH19	111.073	1551.077	3.804	4.43E-07	1.20E-06
OfuSPH16	45.467	769.500	4.081	2.21E-04	4.69E-04
OfuSPH31	29.067	558.787	4.265	4.08E-04	8.27E-04
OfuSP39	148.347	3434.833	4.533	2.51E-09	9.15E-09
OfuSPH34	593.367	13957.037	4.556	4.36E-05	9.63E-05
OfuSP2	7.163	184.673	4.688	5.32E-03	9.03E-03
OfuSP98	96.437	2643.047	4.777	8.37E-09	2.81E-08
OfucSPH6	7.577	209.623	4.790	2.75E-03	5.01E-03
OfuSP105	14.740	494.997	5.070	8.78E-04	1.73E-03
OfuSPH2	7.473	263.553	5.140	1.15E-03	2.20E-03
OfuSPH32	7.577	274.353	5.178	3.16E-04	6.47E-04
OfuSPH6	15.050	558.900	5.215	3.33E-05	7.45E-05
OfuSP32	96.023	3674.290	5.258	1.07E-07	3.15E-07
OfuSP87	22.320	991.797	5.474	1.39E-07	3.96E-07
OfuSP83	29.067	1507.183	5.696	2.84E-05	6.44E-05
OfucSP9	332.403	18277.097	5.781	4.90E-27	2.06E-25
OfuSPH28	37.370	2321.567	5.957	8.22E-11	3.76E-10
OfuSP67	21.490	1508.550	6.133	1.05E-07	3.15E-07
OfucSPH2	29.277	2226.000	6.249	2.47E-05	5.69E-05
OfuSP88	58.963	7093.677	6.911	1.58E-12	8.85E-12
OfuSP85	22.320	3084.933	7.111	8.51E-11	3.76E-10
OfuSP37	146.793	21176.647	7.173	2.80E-23	5.22E-22
OfuSP7	22.527	3250.410	7.173	1.08E-10	4.66E-10
OfuSP72	22.320	3789.943	7.408	1.81E-13	1.05E-12
OfuSP73	30.000	5253.887	7.452	2.17E-16	1.59E-15
OfuSP25	14.327	2752.507	7.586	2.28E-08	7.10E-08
OfucSP24	36.127	7199.260	7.639	1.12E-16	8.66E-16
OfuSP50	136.933	28933.697	7.723	2.32E-14	1.44E-13
OfuSPH18	72.257	16090.803	7.799	2.91E-16	2.04E-15
OfuSP52	15.050	4266.107	8.147	1.05E-11	5.50E-11
OfuSPH12	60.620	17188.087	8.147	1.52E-11	7.52E-11
OfuSP26	44.223	13545.953	8.259	3.58E-22	6.02E-21
OfuSP82	7.577	3031.403	8.644	8.34E-11	3.76E-10
OfuSP96	7.163	3108.517	8.761	2.77E-09	9.91E-09
OfuSP57	88.860	47505.863	9.062	9.64E-18	9.00E-17
OfuSP27	29.483	17921.783	9.248	1.86E-15	1.20E-14
OfuSPH30	37.783	30912.607	9.676	4.95E-28	2.77E-26
OfucSPH4	14.950	20071.280	10.391	8.68E-19	9.11E-18
OfuSP77	22.320	40187.777	10.814	1.04E-28	8.71E-27
OfuSP71	7.163	17042.980	11.216	7.65E-17	6.43E-16
OfuSP21	28.653	74151.723	11.338	1.95E-26	4.67E-25
OfuSPH24	7.473	32955.913	12.107	6.82E-21	8.82E-20
OfucSPH3	7.163	34845.390	12.248	5.69E-21	7.96E-20
OfuSPH8	7.163	39811.260	12.440	2.71E-19	3.04E-18
OfuSP41	0.001	138.240	17.077	4.41E-03	7.64E-03
OfuSP60	0.001	140.060	17.096	3.09E-03	5.58E-03
OfuSP5	0.001	236.537	17.852	2.03E-04	4.42E-04
OfuSPH3	0.001	349.297	18.414	8.68E-06	2.05E-05

OfuSP63	0.001	468.900	18.839	1.79E-06	4.50E-06
OfuSP81	0.001	561.443	19.099	4.99E-07	1.31E-06
OfuSP78	0.001	604.097	19.204	5.05E-06	1.23E-05
OfuSPH5	0.001	651.147	19.313	4.56E-07	1.22E-06
OfuSP8	0.001	898.010	19.776	1.31E-07	3.80E-07
OfuSP100	0.001	1215.710	20.213	1.17E-08	3.87E-08
OfuSP10	0.001	1376.200	20.392	2.08E-10	8.33E-10
OfuSP11	0.001	1422.550	20.440	1.97E-10	8.09E-10
OfuSP44	0.001	1660.727	20.663	2.71E-10	1.06E-09
OfuSP9	0.001	1803.900	20.783	2.43E-11	1.16E-10
OfuSP74	0.001	2350.487	21.165	1.14E-10	4.78E-10
OfuSPH27	0.001	11825.927	23.495	2.09E-17	1.85E-16
OfuSP104	0.001	31412.013	24.905	2.21E-21	3.38E-20
OfuSP28	0.001	33177.077	24.984	1.13E-16	8.66E-16
OfuSP12	0.001	33834.950	25.012	3.52E-20	4.22E-19
OfuSP84	0.001	64667.007	25.947	1.85E-26	4.67E-25
1st L vs 3rd L	1st L mean	3rd L mean	log2(fc)	PValue	q-value
OfuSP98	275.097	0.001	-18.070	2.58E-04	4.30E-03
OfuSP88	174.553	0.001	-17.413	1.31E-03	1.79E-02
OfuSP58	187.533	1.200	-7.288	3.22E-03	3.45E-02
OfuSP56	826.077	9.243	-6.482	1.02E-06	3.83E-05
OfuSP29	644.107	14.043	-5.519	9.66E-06	2.07E-04
OfuSP19	1285.770	28.790	-5.481	3.53E-03	3.53E-02
OfuSPH10	2432.487	13660.547	2.490	2.85E-03	3.29E-02
OfucSPH2	6461.673	48249.507	2.901	1.22E-03	1.79E-02
OfuSPH12	881.203	7876.907	3.160	2.38E-03	2.97E-02
OfucSP26	509.593	6363.850	3.643	3.51E-05	6.58E-04
OfucSP5	430.237	9168.890	4.414	5.92E-06	1.48E-04
OfucSP12	163.540	4694.397	4.843	1.68E-06	5.03E-05
OfucSP7	299.233	19842.007	6.051	9.99E-10	7.49E-08
OfuSPH26	482.130	35249.360	6.192	5.02E-12	7.53E-10
OfucSP20	0.001	6647.087	22.664	1.78E-09	8.91E-08
1st L vs 5th L	1st L mean	5th L mean	log2(fc)	PValue	q-value
OfuSP88	174.553	0.001	-17.413	1.01E-04	6.40E-04
OfuSP7	73.213	0.001	-16.160	1.04E-02	3.45E-02
OfucSPH1	4954.023	38.563	-7.005	9.99E-11	3.30E-09
OfuSPH9	11045.413	189.893	-5.862	5.35E-15	8.84E-13
OfuSP17	315.583	5.693	-5.793	1.70E-05	1.52E-04
OfuSP95	720.917	13.127	-5.779	2.44E-06	2.88E-05
OfuSP29	644.107	16.460	-5.290	1.09E-06	1.50E-05
OfuSP19	1285.770	33.870	-5.247	5.11E-07	8.40E-06
OfuSPH6	46270.053	1277.527	-5.179	4.91E-11	2.02E-09
OfuSP105	29773.500	1196.500	-4.637	1.78E-11	9.79E-10
OfuSP15	1997.500	88.483	-4.497	2.86E-08	5.90E-07
OfuSPH19	161.003	7.280	-4.467	7.11E-03	2.55E-02
OfuSP14	3851.740	271.327	-3.827	6.40E-05	4.40E-04
OfuSP90	119.523	8.940	-3.741	1.35E-02	4.13E-02
OfuSP26	717.317	67.190	-3.416	1.54E-05	1.50E-04
OfuSP93	368.880	37.983	-3.280	9.39E-04	4.84E-03
OfuSP48	159.320	17.967	-3.149	1.72E-02	4.88E-02
OfuSP47	28304.687	3580.423	-2.983	4.50E-05	3.38E-04
OfuSP59	1069.810	158.570	-2.754	5.09E-03	2.00E-02
OfuSP64	4119.107	652.063	-2.659	8.43E-05	5.57E-04
OfuSP23	138.973	22.213	-2.645	1.28E-02	3.97E-02

OfuSPH13	1196.030	199.933	-2.581	4.18E-03	1.77E-02
OfuSP50	1162.127	213.563	-2.444	2.62E-03	1.23E-02
OfuSPH16	5281.187	1037.967	-2.347	5.13E-03	2.00E-02
OfuSPH25	3406.707	887.283	-1.941	9.93E-03	3.34E-02
OfuSPH31	10914.980	2893.243	-1.916	3.46E-03	1.51E-02
OfuSP72	33676.610	9249.720	-1.864	1.63E-02	4.82E-02
OfuSP45	20536.453	5790.667	-1.826	8.78E-03	3.02E-02
OfucSP8	395.657	1398.727	1.822	1.68E-02	4.85E-02
OfucSP18	2123.303	7581.293	1.836	3.47E-03	1.51E-02
OfuSP37	809.430	3240.530	2.001	1.64E-02	4.82E-02
OfuSPH22	1306.653	6596.690	2.336	4.33E-04	2.46E-03
OfucSP1	2476.420	12915.270	2.383	3.00E-03	1.38E-02
OfuSP3	3824.200	20605.447	2.430	2.05E-03	9.94E-03
OfucSPH2	6461.673	37963.290	2.555	1.84E-04	1.08E-03
OfuSP41	253.483	1518.070	2.582	5.22E-03	2.00E-02
OfucSP6	354.350	2141.050	2.595	1.51E-03	7.53E-03
OfuSP32	578.903	3593.537	2.634	1.18E-02	3.81E-02
OfucSP26	509.593	3691.913	2.857	1.38E-04	8.46E-04
OfuSPH11	46.543	357.730	2.942	5.84E-03	2.19E-02
OfuSPH12	881.203	7384.323	3.067	2.21E-05	1.82E-04
OfuSPH1	419.923	3797.547	3.177	6.03E-05	4.32E-04
OfucSPH6	158.807	1559.450	3.296	4.52E-04	2.48E-03
OfuSP33	15.513	152.647	3.299	1.24E-02	3.94E-02
OfucSP5	430.237	4344.947	3.336	3.49E-06	3.84E-05
OfucSP12	163.540	1730.457	3.403	2.91E-05	2.29E-04
OfuSPH10	2432.487	26155.297	3.427	2.56E-07	4.69E-06
OfuSP5	15.513	173.160	3.481	8.26E-03	2.90E-02
OfucSP21	116.340	1424.100	3.614	1.75E-05	1.52E-04
OfucSP7	299.233	3973.403	3.731	1.54E-06	1.95E-05
OfuSP78	120.417	1649.183	3.776	7.89E-04	4.20E-03
OfuSP66	2678.217	46621.987	4.122	1.14E-05	1.17E-04
OfuSPH26	482.130	19783.267	5.359	1.42E-14	1.17E-12
OfucSP23	37.027	2167.963	5.872	5.60E-07	8.40E-06
OfuSP6	2.390	175.723	6.200	7.12E-03	2.55E-02
OfuSP79	28.407	4472.280	7.299	3.32E-10	9.13E-09
OfuSP35	0.001	139.137	17.086	4.54E-03	1.87E-02
OfucSP20	0.001	1984.647	20.921	1.06E-09	2.50E-08
1st L vs P	1st L mean	P mean	log2(fc)	PValue	q-value
OfuSP19	1285.770	0.001	-20.294	8.56E-08	4.41E-07
OfuSP17	315.583	0.001	-18.268	1.34E-05	4.42E-05
OfuSP23	138.973	0.001	-17.084	1.02E-03	2.37E-03
OfuSP45	20536.453	22.487	-9.835	2.67E-21	7.34E-20
OfuSP15	1997.500	2.870	-9.443	1.23E-09	7.81E-09
OfuSP47	28304.687	61.707	-8.841	1.28E-16	2.34E-15
OfuSP105	29773.500	82.253	-8.500	3.21E-14	4.07E-13
OfuSP68	2961.900	13.553	-7.772	1.18E-14	1.62E-13
OfuSP46	4408.917	22.160	-7.636	9.30E-10	6.67E-09
OfuSP14	3851.740	24.717	-7.284	1.42E-13	1.56E-12
OfuSPH9	11045.413	72.963	-7.242	1.51E-15	2.48E-14
OfuSP64	4119.107	30.780	-7.064	2.09E-10	1.57E-09
OfuSP61	300.253	2.713	-6.790	3.92E-05	1.10E-04
OfuSPH6	46270.053	746.050	-5.955	1.27E-07	6.37E-07
OfuSP89	2488.223	40.690	-5.934	1.06E-05	3.58E-05
OfuSP42	29337.513	497.817	-5.881	1.81E-21	5.98E-20

OfuSP20	311.843	5.427	-5.845	3.10E-04	7.87E-04
OfuSP16	4581.413	84.263	-5.765	8.70E-06	2.99E-05
OfuSP18	140766.637	2942.440	-5.580	1.87E-07	8.57E-07
OfuSPH31	10914.980	255.793	-5.415	1.37E-07	6.65E-07
OfuSP48	159.320	4.063	-5.293	8.14E-04	1.95E-03
OfuSP56	826.077	24.380	-5.083	3.23E-04	8.08E-04
OfuSP99	664.967	20.317	-5.033	1.19E-02	2.26E-02
OfuSP27	121.490	4.063	-4.902	9.80E-03	1.90E-02
OfuSPH16	5281.187	179.843	-4.876	7.13E-07	2.99E-06
OfuSPH2	4322.567	157.710	-4.777	1.14E-09	7.53E-09
OfuSPH7	13286.670	556.533	-4.577	6.00E-06	2.11E-05
OfuSP85	15300.950	648.813	-4.560	1.77E-07	8.34E-07
OfuSP67	48246.610	2334.023	-4.370	4.99E-11	3.92E-10
OfuSP80	1044.573	51.410	-4.345	3.91E-06	1.43E-05
OfuSP76	1915.390	94.500	-4.341	3.77E-05	1.07E-04
OfuSP22	4877.743	312.300	-3.965	1.75E-06	6.89E-06
OfuSP1	1674.107	112.693	-3.893	1.15E-03	2.59E-03
OfuSP63	5959.900	404.820	-3.880	1.23E-12	1.12E-11
OfuSP91	5255.373	364.437	-3.850	2.84E-07	1.27E-06
OfuSP2	6792.140	526.977	-3.688	8.70E-05	2.35E-04
OfuSPH13	1196.030	112.893	-3.405	1.37E-05	4.44E-05
OfuSP29	644.107	64.790	-3.314	2.37E-05	6.87E-05
OfucSPH7	114993.677	12084.850	-3.250	5.41E-18	1.12E-16
OfuSP101	4321.473	530.843	-3.025	7.43E-04	1.80E-03
OfuSP41	253.483	33.480	-2.921	5.22E-03	1.09E-02
OfuSPH5	7359.403	1016.863	-2.856	1.04E-02	2.00E-02
OfucSPH5	613.873	88.813	-2.789	1.24E-04	3.25E-04
OfucSP26	509.593	80.363	-2.665	1.23E-03	2.71E-03
OfuSP65	720.677	124.987	-2.528	1.87E-02	3.29E-02
OfuSP3	3824.200	689.040	-2.473	7.66E-03	1.54E-02
OfucSP7	299.233	645.183	1.108	7.50E-03	1.53E-02
OfuSPH26	482.130	1112.620	1.207	1.62E-03	3.43E-03
OfuSP54	2853.283	6641.897	1.219	1.10E-03	2.53E-03
OfucSP15	4118.220	9677.383	1.233	2.23E-05	6.57E-05
OfuSPH21	1138.563	3362.930	1.563	1.50E-03	3.21E-03
OfucSP18	2123.303	6501.607	1.615	1.05E-08	5.96E-08
OfuSPH23	594.280	1960.993	1.722	1.42E-02	2.64E-02
OfucSP19	6662.657	22413.407	1.750	3.87E-08	2.06E-07
OfucSP22	96.413	332.860	1.788	1.63E-02	2.99E-02
OfuSPH28	1487.190	5461.693	1.877	1.73E-04	4.47E-04
OfuSP83	64.447	239.700	1.895	8.35E-03	1.66E-02
OfuSP31	1820.030	7761.293	2.092	3.31E-07	1.44E-06
OfuSP69	31.967	139.800	2.129	1.78E-02	3.17E-02
OfuSP79	28.407	217.057	2.934	1.93E-02	3.35E-02
OfuSPH22	1306.653	10092.143	2.949	5.15E-12	4.47E-11
OfuSPH10	2432.487	22651.160	3.219	3.35E-11	2.77E-10
OfucSPH3	7600.360	71444.190	3.233	4.20E-09	2.57E-08
OfucSP9	2335.077	26708.967	3.516	3.99E-18	9.39E-17
OfuSP32	578.903	6668.170	3.526	1.40E-03	3.03E-03
OfuSP96	11.673	134.593	3.527	1.78E-02	3.17E-02
OfucSP1	2476.420	33191.880	3.745	4.79E-13	4.65E-12
OfucSPH4	2386.633	32491.183	3.767	1.01E-09	6.96E-09
OfuSPH11	46.543	641.900	3.786	4.86E-05	1.34E-04
OfuSP52	14.343	230.957	4.009	2.00E-02	3.44E-02

OfuSP37	809.430	13337.533	4.042	4.82E-23	1.99E-21
OfuSP21	488.553	8149.347	4.060	1.11E-08	6.08E-08
OfucSPH6	158.807	2872.307	4.177	7.66E-09	4.51E-08
OfuSP104	11.673	241.873	4.373	8.86E-04	2.09E-03
OfucSP24	1451.390	30147.890	4.377	3.17E-13	3.27E-12
OfucSPH9	56.103	1317.203	4.553	3.32E-04	8.18E-04
OfucSP3	7.170	175.670	4.615	7.20E-03	1.49E-02
OfuSP33	15.513	438.103	4.820	1.42E-05	4.50E-05
OfuSPH12	881.203	30206.607	5.099	2.63E-24	1.45E-22
OfuSP5	15.513	580.583	5.226	7.24E-07	2.99E-06
OfucSP28	100.913	4654.473	5.527	1.24E-04	3.25E-04
OfucSP21	116.340	7464.457	6.004	6.71E-14	7.91E-13
OfuSP78	120.417	12221.897	6.665	2.62E-15	3.93E-14
OfuSP6	2.390	435.217	7.509	5.38E-06	1.93E-05
OfuSP26	717.317	179489.810	7.967	1.73E-44	2.85E-42
OfuSP71	2.390	120628.933	15.623	3.12E-26	2.57E-24
OfuSP82	0.001	52.773	15.688	2.70E-02	4.49E-02
OfuSPH32	0.001	225.387	17.782	1.55E-05	4.74E-05
OfuSP35	0.001	301.947	18.204	2.66E-06	1.02E-05
OfuSP34	0.001	330.897	18.336	1.68E-06	6.76E-06
1st L vs A	1st L mean	A mean	log2(fc)	PValue	q-value
OfuSP45	20536.453	0.001	-24.292	1.43E-16	1.35E-15
OfuSP16	4581.413	0.001	-22.127	8.69E-11	3.60E-10
OfuSP46	4408.917	0.001	-22.072	3.46E-08	1.03E-07
OfuSP68	2961.900	0.001	-21.498	7.77E-12	3.72E-11
OfuSP89	2488.223	0.001	-21.247	1.66E-08	5.24E-08
OfuSP19	1285.770	0.001	-20.294	2.13E-06	5.04E-06
OfuSP65	720.677	0.001	-19.459	9.08E-06	2.06E-05
OfuSP99	664.967	0.001	-19.343	1.39E-03	2.31E-03
OfucSPH5	613.873	0.001	-19.228	2.41E-07	6.31E-07
OfucSP8	395.657	0.001	-18.594	1.15E-04	2.24E-04
OfucSP6	354.350	0.001	-18.435	1.32E-03	2.22E-03
OfuSP17	315.583	0.001	-18.268	1.40E-04	2.70E-04
OfuSP20	311.843	0.001	-18.251	6.20E-05	1.30E-04
OfuSP61	300.253	0.001	-18.196	6.80E-05	1.39E-04
OfucSP12	163.540	0.001	-17.319	2.92E-03	4.69E-03
OfuSP18	140766.637	108.603	-10.340	8.78E-22	1.49E-20
OfuSP47	28304.687	42.747	-9.371	9.03E-13	4.95E-12
OfuSPH9	11045.413	20.900	-9.046	4.30E-14	3.17E-13
OfuSP101	4321.473	9.520	-8.826	3.18E-09	1.10E-08
OfuSPH7	13286.670	39.287	-8.402	2.18E-11	9.74E-11
OfuSP22	4877.743	17.697	-8.107	1.16E-11	5.32E-11
OfuSP14	3851.740	19.037	-7.661	1.31E-07	3.59E-07
OfuSP64	4119.107	31.583	-7.027	1.82E-06	4.36E-06
OfuSP62	3059.687	31.973	-6.580	4.66E-07	1.18E-06
OfuSPH6	46270.053	558.900	-6.371	9.65E-17	9.65E-16
OfuSP105	29773.500	494.997	-5.911	4.50E-07	1.16E-06
OfuSP97	9649.920	184.840	-5.706	5.39E-35	3.06E-33
OfuSP80	1044.573	22.153	-5.559	1.13E-05	2.50E-05
OfuSP2	6792.140	184.673	-5.201	6.90E-07	1.70E-06
OfucSPH7	114993.677	3349.103	-5.102	1.48E-27	3.59E-26
OfuSP67	48246.610	1508.550	-4.999	5.56E-10	2.10E-09
OfuSP1	1674.107	53.267	-4.974	3.00E-04	5.54E-04
OfuSP4	1924.887	62.350	-4.948	5.07E-07	1.27E-06

OfuSP23	138.973	4.760	-4.868	9.86E-03	1.47E-02
OfuSP66	2678.217	109.640	-4.610	7.68E-05	1.56E-04
OfuSPH31	10914.980	558.787	-4.288	1.11E-04	2.20E-04
OfuSP43	4349.033	232.773	-4.224	5.10E-08	1.49E-07
OfuSPH2	4322.567	263.553	-4.036	1.62E-05	3.49E-05
OfuSP76	1915.390	121.880	-3.974	2.48E-04	4.64E-04
OfuSP63	5959.900	468.900	-3.668	7.46E-10	2.76E-09
OfuSP3	3824.200	317.587	-3.590	7.82E-05	1.56E-04
OfuSPH5	7359.403	651.147	-3.499	1.62E-03	2.68E-03
OfuSP91	5255.373	475.347	-3.467	6.60E-05	1.37E-04
OfucSPH1	4954.023	454.370	-3.447	5.30E-03	8.04E-03
OfuSP15	1997.500	191.360	-3.384	4.64E-04	8.21E-04
OfucSP14	3494.083	339.300	-3.364	4.14E-09	1.41E-08
OfuSP56	826.077	86.060	-3.263	1.60E-05	3.49E-05
OfuSP42	29337.513	3251.223	-3.174	8.10E-08	2.26E-07
OfuSP72	33676.610	3789.943	-3.152	2.71E-03	4.39E-03
OfucSP4	3374.810	392.620	-3.104	2.29E-05	4.87E-05
OfuSPH16	5281.187	769.500	-2.779	1.46E-04	2.79E-04
OfuSPH1	419.923	64.163	-2.710	1.24E-02	1.80E-02
OfucSP13	4275.727	795.800	-2.426	4.06E-03	6.33E-03
OfuSP86	6314.143	1250.100	-2.337	6.08E-04	1.05E-03
OfuSP55	2122.140	426.260	-2.316	2.32E-04	4.39E-04
OfuSP85	15300.950	3084.933	-2.310	9.95E-03	1.47E-02
OfuSP73	23406.600	5253.887	-2.156	7.88E-04	1.34E-03
OfucSP18	2123.303	504.220	-2.074	3.73E-03	5.88E-03
OfucSP11	18363.213	37262.807	1.021	1.87E-03	3.05E-03
OfucSP16	6261.823	15757.900	1.331	1.12E-08	3.66E-08
OfuSPH10	2432.487	7538.287	1.632	4.35E-04	7.78E-04
OfuSP24	1923.747	8160.613	2.085	9.41E-10	3.40E-09
OfucSPH3	7600.360	34845.390	2.197	2.20E-08	6.81E-08
OfucSP24	1451.390	7199.260	2.310	8.98E-06	2.06E-05
OfuSP78	120.417	604.097	2.327	6.72E-03	1.01E-02
OfuSPH11	46.543	280.857	2.593	5.25E-03	8.03E-03
OfuSP32	578.903	3674.290	2.666	2.59E-02	3.73E-02
OfuSP59	1069.810	6937.603	2.697	8.38E-13	4.75E-12
OfuSP69	31.967	223.510	2.806	3.15E-03	5.01E-03
OfuSP39	449.543	3434.833	2.934	6.14E-04	1.05E-03
OfucSP9	2335.077	18277.097	2.969	8.07E-13	4.73E-12
OfucSPH4	2386.633	20071.280	3.072	4.25E-10	1.64E-09
OfuSP79	28.407	252.653	3.153	3.35E-02	4.79E-02
OfuSP98	275.097	2643.047	3.264	3.90E-11	1.70E-10
OfuSPH19	161.003	1551.077	3.268	3.84E-10	1.52E-09
OfuSP5	15.513	236.537	3.931	3.35E-04	6.06E-04
OfuSP44	89.387	1660.727	4.216	1.60E-07	4.25E-07
OfuSP26	717.317	13545.953	4.239	9.90E-13	5.26E-12
OfuSPH12	881.203	17188.087	4.286	5.27E-11	2.24E-10
OfuSPH17	300.120	6098.843	4.345	4.66E-12	2.40E-11
OfuSP30	94.727	2164.840	4.514	1.65E-08	5.24E-08
OfuSP83	64.447	1507.183	4.548	3.18E-04	5.82E-04
OfuSP50	1162.127	28933.697	4.638	1.10E-31	4.66E-30
OfuSP100	46.687	1215.710	4.703	9.63E-06	2.15E-05
OfuSP37	809.430	21176.647	4.709	1.43E-38	1.22E-36
OfuSP25	95.620	2752.507	4.847	1.05E-08	3.51E-08
OfuSP88	174.553	7093.677	5.345	4.54E-14	3.21E-13

OfucSP23	37.027	1610.907	5.443	1.36E-07	3.66E-07
OfuSP7	73.213	3250.410	5.472	2.40E-13	1.51E-12
OfuSP27	121.490	17921.783	7.205	1.67E-13	1.09E-12
OfuSP21	488.553	74151.723	7.246	1.36E-30	4.62E-29
OfuSP77	154.770	40187.777	8.021	1.39E-51	2.36E-49
OfuSP96	11.673	3108.517	8.057	2.50E-09	8.85E-09
OfuSP52	14.343	4266.107	8.216	2.04E-10	8.25E-10
OfuSPH27	38.857	11825.927	8.250	4.19E-19	5.48E-18
OfuSPH34	31.027	13957.037	8.813	6.80E-15	5.78E-14
OfuSPH18	31.027	16090.803	9.019	2.82E-16	2.52E-15
OfuSP57	42.700	47505.863	10.120	3.00E-18	3.64E-17
OfuSPH30	15.513	30912.607	10.961	3.45E-22	6.51E-21
OfuSP104	11.673	31412.013	11.394	5.28E-20	7.47E-19
OfuSPH24	11.673	32955.913	11.463	8.23E-23	1.75E-21
OfuSP71	2.390	17042.980	12.800	1.59E-17	1.69E-16
OfuSP28	2.390	33177.077	13.761	1.73E-14	1.40E-13
OfuSP12	2.390	33834.950	13.789	8.25E-18	9.35E-17
OfuSPH32	0.001	274.353	18.066	2.29E-06	5.33E-06
OfuSPH3	0.001	349.297	18.414	7.56E-07	1.84E-06
OfuSP81	0.001	561.443	19.099	5.58E-08	1.61E-07
OfuSP8	0.001	898.010	19.776	7.44E-08	2.11E-07
OfuSP10	0.001	1376.200	20.392	6.84E-12	3.42E-11
OfuSP11	0.001	1422.550	20.440	7.89E-12	3.72E-11
OfuSP9	0.001	1803.900	20.783	7.45E-13	4.52E-12
OfuSPH33	0.001	2295.450	21.130	2.68E-08	8.14E-08
OfuSP82	0.001	3031.403	21.532	1.06E-13	7.19E-13
OfuSP70	0.001	6051.190	22.529	2.38E-14	1.84E-13
OfuSPH8	0.001	39811.260	25.247	1.61E-21	2.49E-20
OfuSP84	0.001	64667.007	25.947	1.17E-28	3.31E-27
3rd L vs 5th L	3rd L mean	5th L mean	log2(fc)	PValue	q-value
OfuSP100	208.040	3.640	-5.837	8.33E-04	1.65E-02
OfuSPH9	6413.070	189.893	-5.078	2.73E-09	4.13E-07
OfuSP26	816.277	67.190	-3.603	3.10E-04	1.17E-02
OfucSP23	341.047	2167.963	2.668	7.36E-04	1.65E-02
OfucSP21	210.070	1424.100	2.761	1.61E-04	8.09E-03
OfuSP78	51.023	1649.183	5.015	1.17E-03	1.97E-02
OfuSP79	45.337	4472.280	6.624	8.77E-08	6.62E-06
OfuSP6	1.200	175.723	7.194	8.74E-04	1.65E-02
OfuSP35	0.001	139.137	17.086	5.31E-04	1.60E-02
3rd L vs P	3rd L mean	P mean	log2(fc)	PValue	q-value
OfuSP47	12121.963	61.707	-7.618	5.33E-09	8.82E-08
OfucSP20	6647.087	35.730	-7.539	3.78E-13	1.13E-11
OfuSP68	1803.327	13.553	-7.056	1.40E-05	8.66E-05
OfucSP12	4694.397	44.463	-6.722	1.07E-07	1.23E-06
OfuSPH9	6413.070	72.963	-6.458	2.22E-11	5.50E-10
OfucSP26	6363.850	80.363	-6.307	1.90E-16	1.42E-14
OfucSP6	2247.290	40.847	-5.782	7.22E-07	5.98E-06
OfuSP2	20362.797	526.977	-5.272	9.69E-05	4.25E-04
OfuSPH16	6433.903	179.843	-5.161	7.67E-05	3.69E-04
OfuSPH26	35249.360	1112.620	-4.986	1.72E-08	2.56E-07
OfuSP16	2623.013	84.263	-4.960	4.59E-04	1.56E-03
OfucSP5	9168.890	295.407	-4.956	1.18E-07	1.26E-06
OfucSP7	19842.007	645.183	-4.943	1.98E-08	2.69E-07
OfuSP41	883.730	33.480	-4.722	8.79E-07	6.90E-06

OfuSP75	1009.830	44.167	-4.515	1.70E-04	6.70E-04
OfuSP3	15738.990	689.040	-4.514	1.64E-04	6.70E-04
OfucSP8	1536.540	82.763	-4.215	9.77E-08	1.21E-06
OfuSP80	848.337	51.410	-4.045	1.01E-02	2.60E-02
OfuSPH7	9167.953	556.533	-4.042	9.97E-04	3.23E-03
OfuSPH2	2525.967	157.710	-4.002	2.12E-04	8.08E-04
OfuSPH31	3362.970	255.793	-3.717	7.02E-04	2.33E-03
OfuSP85	8422.173	648.813	-3.698	4.82E-07	4.49E-06
OfucSPH7	91765.207	12084.850	-2.925	1.79E-09	3.33E-08
OfuSPH13	827.583	112.893	-2.874	1.60E-03	4.96E-03
OfucSPH2	48249.507	6906.503	-2.805	4.95E-03	1.34E-02
OfucSP23	341.047	50.230	-2.763	1.42E-02	3.40E-02
OfuSP101	3126.437	530.843	-2.558	3.23E-03	9.45E-03
OfucSP19	11026.877	22413.407	1.023	4.70E-03	1.32E-02
OfucSP18	2816.807	6501.607	1.207	1.13E-02	2.81E-02
OfuSPH22	3520.230	10092.143	1.520	2.52E-04	9.40E-04
OfucSP9	9212.620	26708.967	1.536	3.99E-04	1.41E-03
OfucSPH6	975.500	2872.307	1.558	3.65E-03	1.04E-02
OfuSP31	2575.263	7761.293	1.592	4.77E-03	1.32E-02
OfuSPH21	895.027	3362.930	1.910	1.83E-03	5.57E-03
OfuSPH12	7876.907	30206.607	1.939	2.13E-03	6.36E-03
OfuSP83	51.023	239.700	2.232	1.62E-02	3.78E-02
OfucSP29	182.540	910.853	2.319	1.49E-02	3.53E-02
OfucSP1	5966.517	33191.880	2.476	9.36E-05	4.22E-04
OfucSP3	30.133	175.670	2.543	1.06E-02	2.68E-02
OfucSP24	5068.893	30147.890	2.572	1.15E-06	8.14E-06
OfuSP102	3541.343	21531.913	2.604	1.48E-04	6.29E-04
OfucSPH3	10970.307	71444.190	2.703	2.92E-04	1.06E-03
OfuSPH11	81.920	641.900	2.970	9.06E-05	4.22E-04
OfucSPH4	3874.387	32491.183	3.068	6.12E-05	3.13E-04
OfuSP5	19.690	580.583	4.882	1.43E-07	1.42E-06
OfuSP34	9.537	330.897	5.117	8.33E-06	5.47E-05
OfucSP21	210.070	7464.457	5.151	3.44E-11	7.32E-10
OfuSP21	138.533	8149.347	5.878	1.45E-05	8.66E-05
OfuSP94	39.380	2625.617	6.059	1.71E-04	6.70E-04
OfucSP27	2.400	180.947	6.236	1.75E-02	4.01E-02
OfuSP33	4.800	438.103	6.512	4.04E-05	2.23E-04
OfuSP58	1.200	145.450	6.921	1.44E-03	4.56E-03
OfucSP28	24.487	4654.473	7.571	1.64E-05	9.42E-05
OfuSP26	816.277	179489.810	7.781	1.71E-14	6.39E-13
OfuSP78	51.023	12221.897	7.904	1.78E-15	8.84E-14
OfuSP6	1.200	435.217	8.503	5.64E-07	4.94E-06
OfucSPH9	3.600	1317.203	8.515	6.31E-05	3.13E-04
OfuSP88	0.001	101.527	16.632	9.35E-03	2.44E-02
OfuSP98	0.001	117.747	16.845	4.62E-04	1.56E-03
OfuSPH32	0.001	225.387	17.782	8.44E-06	5.47E-05
OfuSP35	0.001	301.947	18.204	9.69E-07	7.22E-06
OfucSP22	0.001	332.860	18.345	4.23E-05	2.25E-04
OfuSP71	0.001	120628.933	26.846	1.90E-27	2.83E-25
3rd L vs A	3rd L mean	A mean	log2(fc)	PValue	q-value
OfucSP12	4694.397	0.001	-22.163	1.17E-09	6.01E-09
OfuSP16	2623.013	0.001	-21.323	6.11E-06	1.74E-05
OfucSP6	2247.290	0.001	-21.100	3.65E-07	1.31E-06
OfuSP68	1803.327	0.001	-20.782	6.41E-05	1.59E-04

OfucSP8	1536.540	0.001	-20.551	4.64E-07	1.59E-06
OfucSPH5	249.160	0.001	-17.927	7.92E-03	1.52E-02
OfucSP20	6647.087	15.190	-8.774	1.12E-07	4.09E-07
OfuSP101	3126.437	9.520	-8.359	2.84E-06	8.59E-06
OfuSPH9	6413.070	20.900	-8.261	1.24E-09	6.14E-09
OfuSP47	12121.963	42.747	-8.148	8.48E-07	2.78E-06
OfuSPH7	9167.953	39.287	-7.866	5.38E-06	1.56E-05
OfucSP7	19842.007	99.640	-7.638	5.24E-13	3.84E-12
OfuSPH26	35249.360	253.927	-7.117	4.80E-12	2.96E-11
OfucSP5	9168.890	66.367	-7.110	1.46E-05	4.01E-05
OfuSP2	20362.797	184.673	-6.785	2.19E-05	5.73E-05
OfuSP3	15738.990	317.587	-5.631	4.51E-05	1.14E-04
OfuSP80	848.337	22.153	-5.259	1.80E-02	3.30E-02
OfuSP97	6800.897	184.840	-5.201	4.63E-16	5.94E-15
OfuSP75	1009.830	34.527	-4.870	3.43E-03	6.94E-03
OfucSPH7	91765.207	3349.103	-4.776	9.09E-13	6.36E-12
OfuSP43	6129.040	232.773	-4.719	3.53E-06	1.05E-05
OfucSP14	6364.663	339.300	-4.230	1.57E-08	6.89E-08
OfucSP26	6363.850	404.160	-3.977	1.78E-05	4.80E-05
OfucSP4	4857.137	392.620	-3.629	6.81E-04	1.44E-03
OfucSP1	5966.517	502.207	-3.571	2.32E-02	4.21E-02
OfuSPH2	2525.967	263.553	-3.261	1.15E-02	2.19E-02
OfuSP55	3540.977	426.260	-3.054	1.60E-04	3.74E-04
OfuSP86	8173.307	1250.100	-2.709	9.60E-04	2.00E-03
OfuSPH12	7876.907	17188.087	1.126	1.30E-02	2.44E-02
OfucSPH3	10970.307	34845.390	1.667	2.56E-04	5.87E-04
OfuSP102	3541.343	11340.340	1.679	1.58E-04	3.74E-04
OfuSP24	2382.527	8160.613	1.776	2.02E-06	6.48E-06
OfuSPH11	81.920	280.857	1.778	4.60E-03	9.20E-03
OfuSP37	4895.023	21176.647	2.113	3.19E-04	7.11E-04
OfucSP23	341.047	1610.907	2.240	2.51E-08	1.02E-07
OfucSPH4	3874.387	20071.280	2.373	2.82E-06	8.59E-06
OfuSP79	45.337	252.653	2.478	2.55E-02	4.57E-02
OfuSP100	208.040	1215.710	2.547	3.16E-04	7.11E-04
OfuSP56	9.243	86.060	3.219	6.37E-03	1.26E-02
OfuSP78	51.023	604.097	3.566	6.52E-04	1.39E-03
OfuSP5	19.690	236.537	3.587	4.70E-04	1.03E-03
OfuSP26	816.277	13545.953	4.053	1.03E-05	2.89E-05
OfuSP30	127.487	2164.840	4.086	2.05E-05	5.44E-05
OfuSP59	292.273	6937.603	4.569	5.22E-12	3.09E-11
OfuSP96	128.577	3108.517	4.596	3.62E-05	9.29E-05
OfuSP83	51.023	1507.183	4.885	1.18E-04	2.85E-04
OfuSP50	856.420	28933.697	5.078	8.13E-17	1.25E-15
OfuSP39	87.153	3434.833	5.301	9.59E-08	3.60E-07
OfuSPH17	117.413	6098.843	5.699	3.15E-14	2.85E-13
OfuSP25	39.380	2752.507	6.127	1.41E-08	6.37E-08
OfuSP7	33.340	3250.410	6.607	2.76E-08	1.09E-07
OfuSP82	23.290	3031.403	7.024	9.35E-15	9.60E-14
OfuSP70	36.980	6051.190	7.354	5.02E-09	2.34E-08
OfuSPH34	75.923	13957.037	7.522	6.33E-11	3.48E-10
OfuSP52	17.993	4266.107	7.889	5.19E-07	1.74E-06
OfuSP27	55.467	17921.783	8.336	2.26E-08	9.68E-08
OfuSP77	113.190	40187.777	8.472	2.80E-16	3.92E-15
OfuSPH19	3.600	1551.077	8.751	3.79E-10	2.01E-09

OfuSP21	138.533	74151.723	9.064	9.09E-14	7.36E-13
OfuSP104	51.210	31412.013	9.261	9.73E-19	2.50E-17
OfuSP84	58.347	64667.007	10.114	9.01E-15	9.60E-14
OfuSPH30	18.490	30912.607	10.707	8.38E-22	4.30E-20
OfuSPH24	8.337	32955.913	11.949	4.19E-25	6.45E-23
OfuSPH27	1.200	11825.927	13.267	2.04E-17	3.50E-16
OfuSPH18	1.200	16090.803	13.711	4.67E-18	8.99E-17
OfuSPH32	0.001	274.353	18.066	2.39E-06	7.50E-06
OfuSPH3	0.001	349.297	18.414	4.49E-07	1.57E-06
OfuSP81	0.001	561.443	19.099	2.49E-08	1.02E-07
OfuSP8	0.001	898.010	19.776	3.77E-08	1.45E-07
OfuSP10	0.001	1376.200	20.392	2.40E-12	1.54E-11
OfuSP11	0.001	1422.550	20.440	1.82E-12	1.22E-11
OfuSP44	0.001	1660.727	20.663	2.83E-11	1.61E-10
OfuSP9	0.001	1803.900	20.783	2.16E-13	1.67E-12
OfuSPH33	0.001	2295.450	21.130	3.31E-09	1.59E-08
OfuSP98	0.001	2643.047	21.334	2.58E-14	2.48E-13
OfuSP88	0.001	7093.677	22.758	3.95E-14	3.38E-13
OfuSP71	0.001	17042.980	24.023	2.19E-20	8.41E-19
OfuSP28	0.001	33177.077	24.984	8.65E-16	1.02E-14
OfuSP12	0.001	33834.950	25.012	7.67E-20	2.36E-18
OfuSPH8	0.001	39811.260	25.247	5.98E-23	4.60E-21
OfuSP57	0.001	47505.863	25.502	2.28E-18	5.01E-17
5th L vs P	5th L mean	P mean	log2(fc)	PValue	q-value
OfuSP68	6555.960	13.553	-8.918	3.09E-20	1.04E-18
OfuSP45	5790.667	22.487	-8.009	4.26E-15	1.03E-13
OfuSP46	2456.053	22.160	-6.792	5.42E-13	1.14E-11
OfuSPH15	4154.337	37.977	-6.773	1.87E-09	1.37E-08
OfuSP42	36530.390	497.817	-6.197	5.92E-23	3.33E-21
OfuSP89	2902.260	40.690	-6.156	6.75E-09	4.57E-08
OfuSP22	19052.433	312.300	-5.931	3.23E-12	4.19E-11
OfuSP47	3580.423	61.707	-5.859	1.66E-12	2.75E-11
OfucSP20	1984.647	35.730	-5.796	3.39E-11	3.19E-10
OfuSP66	46621.987	878.867	-5.729	2.44E-15	6.86E-14
OfucSP6	2141.050	40.847	-5.712	6.15E-10	4.95E-09
OfuSP16	4337.413	84.263	-5.686	1.33E-06	6.43E-06
OfuSP92	12076.037	245.210	-5.622	1.06E-10	8.98E-10
OfucSP26	3691.913	80.363	-5.522	1.79E-12	2.75E-11
OfuSP41	1518.070	33.480	-5.503	4.73E-07	2.50E-06
OfuSP18	131409.550	2942.440	-5.481	4.20E-12	5.07E-11
OfucSP23	2167.963	50.230	-5.432	8.62E-10	6.62E-09
OfuSP2	22373.287	526.977	-5.408	1.04E-11	1.10E-10
OfucSP12	1730.457	44.463	-5.282	8.97E-08	5.05E-07
OfuSPH7	20607.247	556.533	-5.211	9.12E-12	1.03E-10
OfuSP61	89.403	2.713	-5.042	5.06E-03	1.17E-02
OfuSP67	74613.490	2334.023	-4.999	2.49E-20	1.04E-18
OfuSP15	88.483	2.870	-4.946	8.98E-03	1.95E-02
OfuSP3	20605.447	689.040	-4.902	2.45E-11	2.44E-10
OfuSP63	10543.300	404.820	-4.703	1.55E-12	2.75E-11
OfuSPH1	3797.547	155.257	-4.612	9.83E-07	4.89E-06
OfuSP85	14554.747	648.813	-4.488	1.21E-08	7.88E-08
OfuSP64	652.063	30.780	-4.405	6.34E-06	2.75E-05
OfuSP79	4472.280	217.057	-4.365	1.63E-08	9.86E-08
OfuSP101	9509.967	530.843	-4.163	8.98E-05	3.16E-04

OfuSPH26	19783.267	1112.620	-4.152	3.84E-11	3.41E-10
OfucSP8	1398.727	82.763	-4.079	1.11E-07	6.03E-07
OfucSP5	4344.947	295.407	-3.879	2.05E-08	1.19E-07
OfuSPH2	2316.937	157.710	-3.877	7.40E-07	3.79E-06
OfuSP105	1196.500	82.253	-3.863	9.68E-05	3.34E-04
OfuSP75	618.923	44.167	-3.809	5.01E-06	2.23E-05
OfuSPH25	887.283	71.427	-3.635	1.63E-08	9.86E-08
OfuSP1	1395.077	112.693	-3.630	1.03E-03	2.69E-03
OfuSPH31	2893.243	255.793	-3.500	1.07E-05	4.21E-05
OfuSP80	568.417	51.410	-3.467	7.64E-06	3.23E-05
OfuSP14	271.327	24.717	-3.457	1.68E-02	3.19E-02
OfuSP76	1004.647	94.500	-3.410	2.29E-05	8.81E-05
OfuSP65	1249.380	124.987	-3.321	7.38E-04	1.98E-03
OfuSP4	2728.923	291.093	-3.229	4.20E-03	1.01E-02
OfuSP99	186.833	20.317	-3.201	1.21E-02	2.46E-02
OfucSP7	3973.403	645.183	-2.623	6.05E-04	1.70E-03
OfuSPH16	1037.967	179.843	-2.529	4.58E-03	1.09E-02
OfuSPH5	5807.440	1016.863	-2.514	3.33E-03	8.40E-03
OfuSP91	2059.890	364.437	-2.499	1.72E-04	5.59E-04
OfucSPH2	37963.290	6906.503	-2.459	6.48E-04	1.80E-03
OfuSP54	2863.667	6641.897	1.214	9.52E-03	2.01E-02
OfuSP6	175.723	435.217	1.308	1.44E-02	2.80E-02
OfuSP33	152.647	438.103	1.521	1.24E-02	2.49E-02
OfuSP5	173.160	580.583	1.745	4.12E-03	1.01E-02
OfuSP43	2672.733	10303.477	1.947	5.18E-04	1.48E-03
OfuSP59	158.570	647.263	2.029	7.68E-03	1.69E-02
OfuSPH12	7384.323	30206.607	2.032	5.53E-05	1.99E-04
OfuSP37	3240.530	13337.533	2.041	5.42E-03	1.22E-02
OfuSP83	53.873	239.700	2.154	2.41E-02	4.47E-02
OfucSP9	5980.903	26708.967	2.159	1.11E-04	3.75E-04
OfuSP34	72.203	330.897	2.196	9.26E-03	1.98E-02
OfuSP31	1579.133	7761.293	2.297	2.27E-04	7.05E-04
OfuSP52	46.473	230.957	2.313	5.18E-03	1.18E-02
OfucSPH3	14360.453	71444.190	2.315	4.69E-05	1.72E-04
OfucSP21	1424.100	7464.457	2.390	2.01E-04	6.40E-04
OfuSP69	25.770	139.800	2.440	2.58E-02	4.74E-02
OfuSPH23	351.867	1960.993	2.479	5.00E-03	1.17E-02
OfucSP29	162.903	910.853	2.483	1.11E-02	2.28E-02
OfuSP102	3818.150	21531.913	2.496	2.57E-06	1.18E-05
OfucSP24	4923.113	30147.890	2.614	9.13E-06	3.67E-05
OfucSP22	54.027	332.860	2.623	2.64E-02	4.80E-02
OfucSPH4	4711.363	32491.183	2.786	3.47E-04	1.03E-03
OfuSP93	37.983	264.093	2.798	3.24E-04	9.77E-04
OfuSP78	1649.183	12221.897	2.890	2.53E-03	6.49E-03
OfuSP104	31.000	241.873	2.964	1.60E-02	3.07E-02
OfuSP96	14.030	134.593	3.262	1.31E-02	2.58E-02
OfuSPH32	22.153	225.387	3.347	2.30E-04	7.05E-04
OfuSPH21	277.870	3362.930	3.597	3.49E-09	2.46E-08
OfuSPH19	7.280	91.667	3.654	7.59E-03	1.69E-02
OfuSP95	13.127	195.767	3.899	3.79E-04	1.11E-03
OfucSPH9	86.057	1317.203	3.936	1.57E-04	5.19E-04
OfucSPH1	38.563	712.170	4.207	3.60E-03	8.95E-03
OfuSP82	1.897	52.773	4.798	1.29E-02	2.56E-02
OfuSP100	3.640	189.547	5.703	2.74E-05	1.03E-04

OfuSP21	150.150	8149.347	5.762	2.68E-12	3.77E-11
OfucSP28	75.410	4654.473	5.948	2.30E-06	1.08E-05
OfuSP94	37.400	2625.617	6.134	8.80E-06	3.63E-05
OfucSP27	2.347	180.947	6.269	8.51E-04	2.25E-03
OfuSP26	67.190	179489.810	11.383	5.50E-92	9.29E-90
OfuSP7	0.001	45.670	15.479	1.10E-02	2.28E-02
OfuSP88	0.001	101.527	16.632	6.97E-04	1.90E-03
OfuSP71	0.001	120628.933	26.846	7.02E-34	5.93E-32
5th L vs A	5th L mean	A mean	log2(fc)	PValue	q-value
OfuSP68	6555.960	0.001	-22.644	1.32E-13	5.72E-13
OfuSP45	5790.667	0.001	-22.465	3.93E-12	1.49E-11
OfuSP16	4337.413	0.001	-22.048	8.69E-11	3.03E-10
OfuSPH15	4154.337	0.001	-21.986	9.78E-11	3.34E-10
OfuSP89	2902.260	0.001	-21.469	2.04E-10	6.69E-10
OfuSP46	2456.053	0.001	-21.228	1.59E-09	4.94E-09
OfucSP6	2141.050	0.001	-21.030	1.52E-09	4.82E-09
OfucSP12	1730.457	0.001	-20.723	1.06E-08	3.02E-08
OfucSP8	1398.727	0.001	-20.416	4.31E-08	1.21E-07
OfuSP65	1249.380	0.001	-20.253	2.64E-07	6.87E-07
OfuSP99	186.833	0.001	-17.511	3.01E-03	5.36E-03
OfucSPH5	179.417	0.001	-17.453	3.60E-03	6.32E-03
OfuSP18	131409.550	108.603	-10.241	5.35E-38	3.10E-36
OfuSP22	19052.433	17.697	-10.072	1.67E-15	9.06E-15
OfuSP101	9509.967	9.520	-9.964	1.38E-09	4.46E-09
OfuSPH7	20607.247	39.287	-9.035	4.57E-20	3.46E-19
OfuSP92	12076.037	24.013	-8.974	2.88E-15	1.47E-14
OfuSP66	46621.987	109.640	-8.732	2.44E-24	2.66E-23
OfucSP20	1984.647	15.190	-7.030	3.75E-07	9.48E-07
OfuSP2	22373.287	184.673	-6.921	9.26E-14	4.13E-13
OfuSP47	3580.423	42.747	-6.388	8.16E-09	2.41E-08
OfuSPH26	19783.267	253.927	-6.284	2.48E-13	1.05E-12
OfucSP5	4344.947	66.367	-6.033	3.76E-07	9.48E-07
OfuSP3	20605.447	317.587	-6.020	1.26E-12	4.88E-12
OfuSPH1	3797.547	64.163	-5.887	1.06E-08	3.02E-08
OfuSP67	74613.490	1508.550	-5.628	6.71E-13	2.66E-12
OfuSP4	2728.923	62.350	-5.452	5.74E-08	1.58E-07
OfucSP7	3973.403	99.640	-5.318	7.43E-08	2.02E-07
OfuSP62	1072.877	31.973	-5.069	3.41E-04	6.83E-04
OfuSP97	5579.150	184.840	-4.916	2.60E-09	7.80E-09
OfuSP6	175.723	6.317	-4.798	1.77E-02	2.78E-02
OfuSP1	1395.077	53.267	-4.711	4.03E-04	7.87E-04
OfucSP1	12915.270	502.207	-4.685	1.29E-05	2.99E-05
OfuSP80	568.417	22.153	-4.681	6.16E-04	1.18E-03
OfuSP63	10543.300	468.900	-4.491	1.38E-07	3.69E-07
OfuSP64	652.063	31.583	-4.368	4.23E-03	7.28E-03
OfuSP75	618.923	34.527	-4.164	1.68E-03	3.11E-03
OfuSP79	4472.280	252.653	-4.146	1.90E-04	4.02E-04
OfucSP14	5856.893	339.300	-4.110	1.49E-07	3.94E-07
OfucSPH2	37963.290	2226.000	-4.092	4.52E-03	7.71E-03
OfucSP21	1424.100	84.200	-4.080	2.92E-05	6.59E-05
OfucSP18	7581.293	504.220	-3.910	6.98E-06	1.69E-05
OfuSP43	2672.733	232.773	-3.521	2.76E-04	5.65E-04
OfuSP42	36530.390	3251.223	-3.490	3.23E-05	7.21E-05
OfuSP41	1518.070	138.240	-3.457	1.30E-02	2.06E-02

OfucSPH7	34444.727	3349.103	-3.362	3.88E-04	7.67E-04
OfucSP4	3887.843	392.620	-3.308	2.55E-04	5.28E-04
OfucSP26	3691.913	404.160	-3.191	1.97E-03	3.58E-03
OfuSPH5	5807.440	651.147	-3.157	1.71E-03	3.14E-03
OfuSPH2	2316.937	263.553	-3.136	4.84E-03	8.18E-03
OfuSP76	1004.647	121.880	-3.043	8.53E-03	1.39E-02
OfuSPH25	887.283	112.927	-2.974	9.24E-03	1.47E-02
OfucSPH6	1559.450	209.623	-2.895	2.77E-02	4.26E-02
OfuSP86	8729.437	1250.100	-2.804	9.35E-04	1.77E-03
OfuSPH22	6596.690	1024.893	-2.686	6.60E-03	1.09E-02
OfuSP55	2281.013	426.260	-2.420	1.82E-02	2.82E-02
OfuSPH23	351.867	734.397	1.062	9.13E-03	1.47E-02
OfuSPH12	7384.323	17188.087	1.219	3.89E-03	6.77E-03
OfucSPH3	14360.453	34845.390	1.279	1.78E-04	3.83E-04
OfuSP102	3818.150	11340.340	1.571	1.55E-05	3.54E-05
OfucSP9	5980.903	18277.097	1.612	1.98E-04	4.15E-04
OfuSPH21	277.870	900.457	1.696	1.20E-04	2.61E-04
OfucSPH4	4711.363	20071.280	2.091	1.17E-04	2.58E-04
OfuSPH13	199.933	1232.243	2.624	1.11E-05	2.61E-05
OfuSP37	3240.530	21176.647	2.708	4.54E-06	1.11E-05
OfuSP69	25.770	223.510	3.117	5.80E-04	1.12E-03
OfuSP29	16.460	157.457	3.258	5.69E-03	9.52E-03
OfucSPH1	38.563	454.370	3.559	3.37E-04	6.81E-04
OfuSP24	690.177	8160.613	3.564	7.21E-15	3.48E-14
OfuSPH32	22.153	274.353	3.630	8.37E-06	2.00E-05
OfuSP95	13.127	167.517	3.674	3.02E-03	5.36E-03
OfuSP39	259.303	3434.833	3.728	1.55E-10	5.19E-10
OfuSP83	53.873	1507.183	4.806	8.99E-07	2.24E-06
OfuSP59	158.570	6937.603	5.451	5.88E-16	3.41E-15
OfuSP98	47.243	2643.047	5.806	5.44E-16	3.26E-15
OfuSPH17	100.490	6098.843	5.923	1.76E-22	1.62E-21
OfuSP30	25.257	2164.840	6.422	1.53E-17	1.03E-16
OfuSP52	46.473	4266.107	6.520	7.17E-23	6.93E-22
OfuSP50	213.563	28933.697	7.082	2.81E-34	7.42E-33
OfuSP26	67.190	13545.953	7.655	6.77E-40	5.89E-38
OfuSPH19	7.280	1551.077	7.735	2.54E-13	1.05E-12
OfuSP77	187.857	40187.777	7.741	2.99E-34	7.42E-33
OfuSP96	14.030	3108.517	7.792	1.72E-17	1.11E-16
OfuSPH34	46.853	13957.037	8.219	1.19E-20	9.83E-20
OfuSP100	3.640	1215.710	8.384	1.44E-11	5.22E-11
OfuSP25	7.043	2752.507	8.610	1.53E-14	7.18E-14
OfuSP21	150.150	74151.723	8.948	1.42E-34	4.95E-33
OfuSPH27	18.203	11825.927	9.344	2.83E-23	2.89E-22
OfuSP27	24.190	17921.783	9.533	1.52E-21	1.32E-20
OfuSP44	1.897	1660.727	9.774	3.43E-13	1.39E-12
OfuSP104	31.000	31412.013	9.985	3.13E-32	6.81E-31
OfuSPH18	14.563	16090.803	10.110	6.23E-26	8.34E-25
OfuSPH30	22.813	30912.607	10.404	1.61E-45	2.81E-43
OfuSP82	1.897	3031.403	10.642	7.26E-16	4.08E-15
OfuSP70	2.347	6051.190	11.332	2.82E-18	2.05E-17
OfuSP57	10.923	47505.863	12.087	4.80E-28	7.60E-27
OfuSP28	7.043	33177.077	12.202	1.78E-24	2.06E-23
OfuSP84	10.923	64667.007	12.531	1.21E-37	5.25E-36
OfuSPH3	0.001	349.297	18.414	2.52E-09	7.71E-09

OfuSP81	0.001	561.443	19.099	3.28E-11	1.16E-10
OfuSP8	0.001	898.010	19.776	5.70E-12	2.11E-11
OfuSP10	0.001	1376.200	20.392	3.39E-15	1.68E-14
OfuSP11	0.001	1422.550	20.440	2.51E-15	1.32E-14
OfuSP9	0.001	1803.900	20.783	2.27E-16	1.41E-15
OfuSPH33	0.001	2295.450	21.130	5.65E-14	2.59E-13
OfuSP7	0.001	3250.410	21.632	1.01E-17	7.04E-17
OfuSP88	0.001	7093.677	22.758	2.19E-20	1.73E-19
OfuSP71	0.001	17042.980	24.023	2.02E-25	2.51E-24
OfuSPH24	0.001	32955.913	24.974	3.08E-30	5.95E-29
OfuSP12	0.001	33834.950	25.012	5.84E-27	8.47E-26
OfuSPH8	0.001	39811.260	25.247	6.60E-29	1.15E-27
P vs A	P mean	A mean	log2(fc)	PValue	q-value
OfuSP34	330.897	0.001	-18.336	5.58E-06	1.71E-05
OfucSP27	180.947	0.001	-17.465	5.73E-03	1.22E-02
OfuSP65	124.987	0.001	-16.931	9.83E-03	2.00E-02
OfucSPH5	88.813	0.001	-16.439	9.52E-03	1.98E-02
OfucSP8	82.763	0.001	-16.337	1.26E-02	2.51E-02
OfucSPH9	1317.203	5.063	-8.023	8.69E-06	2.58E-05
OfucSP28	4654.473	19.647	-7.888	9.94E-07	3.22E-06
OfucSP21	7464.457	84.200	-6.470	1.58E-15	1.20E-14
OfuSP6	435.217	6.317	-6.106	1.78E-05	5.09E-05
OfucSP1	33191.880	502.207	-6.046	6.30E-17	6.12E-16
OfuSP101	530.843	9.520	-5.801	8.43E-05	2.27E-04
OfuSP43	10303.477	232.773	-5.468	2.86E-16	2.63E-15
OfuSP62	1205.333	31.973	-5.236	6.16E-05	1.71E-04
OfuSP18	2942.440	108.603	-4.760	1.65E-08	6.14E-08
OfuSP97	4942.570	184.840	-4.741	5.57E-15	3.90E-14
OfuSP94	2625.617	116.483	-4.495	1.73E-03	3.99E-03
OfuSP78	12221.897	604.097	-4.339	5.18E-06	1.62E-05
OfuSP22	312.300	17.697	-4.141	1.10E-03	2.64E-03
OfuSP35	301.947	18.950	-3.994	1.66E-03	3.88E-03
OfuSPH7	556.533	39.287	-3.824	1.00E-03	2.44E-03
OfucSPH6	2872.307	209.623	-3.776	1.05E-07	3.73E-07
OfuSP26	179489.810	13545.953	-3.728	5.95E-13	3.36E-12
OfucSP14	4473.680	339.300	-3.721	3.60E-13	2.17E-12
OfucSP18	6501.607	504.220	-3.689	1.33E-09	5.30E-09
OfuSP92	245.210	24.013	-3.352	2.12E-02	4.04E-02
OfuSPH22	10092.143	1024.893	-3.300	1.67E-10	7.12E-10
OfuSP86	11341.287	1250.100	-3.182	2.01E-09	7.84E-09
OfuSP66	878.867	109.640	-3.003	4.15E-03	9.08E-03
OfuSP33	438.103	56.033	-2.967	2.48E-03	5.56E-03
OfucSP13	5798.850	795.800	-2.865	2.41E-06	7.68E-06
OfuSP71	120628.933	17042.980	-2.823	6.20E-06	1.87E-05
OfuSP55	3000.527	426.260	-2.815	9.26E-08	3.38E-07
OfucSP7	645.183	99.640	-2.695	7.37E-04	1.82E-03
OfucSP4	2072.287	392.620	-2.400	4.11E-04	1.04E-03
OfuSP73	24135.217	5253.887	-2.200	8.68E-07	2.86E-06
OfuSPH26	1112.620	253.927	-2.132	8.27E-03	1.74E-02
OfucSP24	30147.890	7199.260	-2.066	5.03E-04	1.26E-03
OfuSPH21	3362.930	900.457	-1.901	1.56E-02	3.04E-02
OfucSPH7	12084.850	3349.103	-1.851	2.19E-04	5.64E-04
OfuSP31	7761.293	2382.237	-1.704	1.52E-03	3.61E-03
OfucSP15	9677.383	3258.327	-1.571	4.55E-03	9.82E-03

OfuSP54	6641.897	2344.977	-1.502	1.43E-02	2.81E-02
OfuSP38	16314.573	5894.027	-1.469	2.53E-02	4.76E-02
OfucSP16	7182.683	15757.900	1.134	1.48E-04	3.88E-04
OfuSPH16	179.843	769.500	2.097	2.27E-03	5.17E-03
OfuSP85	648.813	3084.933	2.249	1.04E-04	2.76E-04
OfucSP26	80.363	404.160	2.330	2.08E-05	5.87E-05
OfuSP83	239.700	1507.183	2.653	3.72E-03	8.24E-03
OfuSP100	189.547	1215.710	2.681	1.10E-05	3.22E-05
OfuSP42	497.817	3251.223	2.707	3.20E-12	1.65E-11
OfuSP21	8149.347	74151.723	3.186	1.34E-07	4.61E-07
OfuSP59	647.263	6937.603	3.422	4.44E-11	2.10E-10
OfuSPH13	112.893	1232.243	3.448	1.57E-10	6.86E-10
OfuSP39	248.793	3434.833	3.787	1.14E-07	3.98E-07
OfuSPH19	91.667	1551.077	4.081	6.68E-12	3.25E-11
OfuSP24	453.820	8160.613	4.169	5.15E-13	3.01E-12
OfuSP52	230.957	4266.107	4.207	8.96E-16	7.47E-15
OfuSP98	117.747	2643.047	4.488	1.03E-18	1.29E-17
OfuSP96	134.593	3108.517	4.530	2.69E-07	9.04E-07
OfuSPH17	224.520	6098.843	4.764	2.23E-15	1.63E-14
OfucSP23	50.230	1610.907	5.003	6.21E-16	5.43E-15
OfuSP30	61.860	2164.840	5.129	4.22E-10	1.72E-09
OfuSP44	37.560	1660.727	5.467	4.33E-14	2.71E-13
OfuSP82	52.773	3031.403	5.844	1.34E-21	1.95E-20
OfuSP15	2.870	191.360	6.059	7.21E-05	1.97E-04
OfuSP88	101.527	7093.677	6.127	2.75E-12	1.46E-11
OfuSP7	45.670	3250.410	6.153	4.21E-17	4.33E-16
OfuSP50	397.320	28933.697	6.186	2.12E-61	1.86E-59
OfuSPH34	114.593	13957.037	6.928	7.97E-11	3.67E-10
OfuSP104	241.873	31412.013	7.021	3.37E-26	7.37E-25
OfuSP10	8.127	1376.200	7.404	1.96E-12	1.07E-11
OfuSP25	12.360	2752.507	7.799	2.69E-14	1.75E-13
OfuSP77	176.643	40187.777	7.830	6.54E-64	1.14E-61
OfuSPH18	39.237	16090.803	8.680	9.86E-25	1.73E-23
OfuSP28	38.740	33177.077	9.742	2.84E-25	5.53E-24
OfuSPH30	29.417	30912.607	10.037	1.42E-49	8.28E-48
OfuSP84	42.787	64667.007	10.562	2.33E-41	8.16E-40
OfuSPH24	13.877	32955.913	11.214	1.47E-47	6.43E-46
OfuSP57	16.267	47505.863	11.512	4.37E-28	1.09E-26
OfuSPH8	9.490	39811.260	12.035	2.97E-32	8.66E-31
OfuSPH27	2.713	11825.927	12.090	8.24E-20	1.11E-18
OfuSP27	4.063	17921.783	12.107	1.06E-17	1.16E-16
OfuSP12	2.870	33834.950	13.525	7.58E-23	1.21E-21
OfuSPH3	0.001	349.297	18.414	1.09E-08	4.14E-08
OfuSP81	0.001	561.443	19.099	3.30E-10	1.37E-09
OfuSP8	0.001	898.010	19.776	1.36E-10	6.11E-10
OfuSP11	0.001	1422.550	20.440	1.28E-14	8.61E-14
OfuSP9	0.001	1803.900	20.783	1.04E-15	8.28E-15
OfuSPH33	0.001	2295.450	21.130	5.23E-12	2.62E-11
OfuSP70	0.001	6051.190	22.529	1.90E-18	2.22E-17

(B)

M vs SG	M mean	SG mean	log2(fc)	PValue	q-value
OfuSPH25	3100.043	0.001	-21.564	5.82E-23	2.63E-22
OfuSP76	1726.083	0.001	-20.719	3.28E-21	1.40E-20

OfuSP60	780.367	0.001	-19.574	8.99E-18	3.74E-17
OfuSP65	729.657	0.001	-19.477	5.39E-17	2.18E-16
OfuSP49	568.753	0.001	-19.117	7.21E-16	2.78E-15
OfuSP15	258.307	0.001	-17.979	5.73E-13	1.97E-12
OfuSP21	257.290	0.001	-17.973	8.18E-12	2.59E-11
OfuSP61	237.737	0.001	-17.859	1.86E-12	6.25E-12
OfuSP48	233.693	0.001	-17.834	7.00E-08	2.09E-07
OfuSP19	100.940	0.001	-16.623	3.81E-08	1.16E-07
OfuSP20	75.017	0.001	-16.195	1.78E-07	5.19E-07
OfuSP17	66.520	0.001	-16.022	8.16E-07	2.22E-06
OfuSP23	35.563	0.001	-15.118	5.70E-05	1.41E-04
OfuSP56	26.913	0.001	-14.716	8.10E-05	1.97E-04
OfuSP89	4224.980	14.050	-8.232	3.89E-23	1.81E-22
OfuSP74	50822.750	180.187	-8.140	3.66E-87	8.26E-86
OfuSP2	57599.197	220.073	-8.032	1.78E-116	9.36E-115
OfuSP101	6124.633	23.567	-8.022	7.22E-24	3.56E-23
OfuSP47	12533.267	53.937	-7.860	3.16E-33	1.92E-32
OfuSP32	5942.600	26.123	-7.830	3.30E-27	1.68E-26
OfuSP72	68144.093	408.877	-7.381	1.14E-108	4.50E-107
OfuSPH6	13364.083	83.357	-7.325	4.09E-45	3.80E-44
OfuSP16	2996.800	23.857	-6.973	5.17E-22	2.27E-21
OfuSP3	64893.103	541.560	-6.905	6.26E-131	9.89E-129
OfuSP18	338272.223	3021.213	-6.807	5.27E-129	4.17E-127
OfuSP64	1316.070	12.073	-6.768	1.32E-16	5.23E-16
OfuSPH5	26369.467	245.090	-6.749	4.05E-73	7.11E-72
OfuSP22	11058.753	104.207	-6.730	9.26E-41	7.31E-40
OfuSP99	1235.640	11.783	-6.712	1.06E-15	4.00E-15
OfuSPH31	16479.313	157.487	-6.709	2.83E-56	4.06E-55
OfuSP92	28742.630	282.707	-6.668	1.28E-100	3.37E-99
OfuSP1	5270.483	60.080	-6.455	1.32E-29	6.97E-29
OfuSPH2	4572.590	62.053	-6.203	1.33E-38	8.78E-38
OfuSP46	6019.820	83.067	-6.179	6.75E-44	5.93E-43
OfuSP62	2599.170	35.930	-6.177	1.30E-23	6.20E-23
OfuSP66	47496.610	719.110	-6.046	9.19E-51	1.12E-49
OfuSPH7	34600.087	559.143	-5.951	5.03E-84	9.94E-83
OfuSPH1	7067.497	135.027	-5.710	3.21E-57	5.07E-56
OfuSP42	26451.887	511.873	-5.691	1.39E-47	1.46E-46
OfuSPH15	12304.103	248.913	-5.627	3.11E-39	2.14E-38
OfuSP67	62113.357	1365.113	-5.508	9.10E-101	2.87E-99
OfuSP105	18609.047	421.323	-5.465	5.38E-48	6.07E-47
OfuSP91	8412.867	194.290	-5.436	1.38E-31	7.81E-31
OfuSPH4	560.493	14.050	-5.318	7.66E-12	2.47E-11
OfuSP68	2601.373	71.863	-5.178	7.01E-33	4.10E-32
OfuSP45	8724.463	252.627	-5.110	2.42E-42	2.01E-41
OfuSP14	477.383	14.050	-5.087	4.76E-13	1.67E-12
OfuSP80	1199.937	35.930	-5.062	1.65E-15	6.07E-15
OfuSP4	3803.537	119.580	-4.991	1.91E-38	1.21E-37
OfuSP75	1686.777	75.240	-4.487	1.21E-29	6.61E-29
OfuSP63	8451.600	382.783	-4.465	1.66E-39	1.25E-38
OfuSP40	6147.523	341.337	-4.171	3.66E-56	4.81E-55
OfuSP52	98.147	11.783	-3.058	2.26E-07	6.37E-07
OfuSP39	222.733	912.603	2.035	1.30E-02	2.71E-02
OfucSP15	23.290	1239.813	5.734	4.19E-04	9.74E-04
OfucSP11	66.473	3578.457	5.750	2.93E-05	7.46E-05

OfuSP55	29.430	1651.990	5.811	8.44E-03	1.78E-02
OfuSP31	7.780	462.787	5.894	2.44E-02	4.94E-02
OfucSP10	85.020	5274.400	5.955	1.37E-05	3.55E-05
OfuSP13	14.057	1118.313	6.314	4.60E-05	1.15E-04
OfuSPH21	12.773	1021.823	6.322	4.53E-04	1.04E-03
OfucSP2	13.633	1116.837	6.356	2.74E-04	6.46E-04
OfucSP21	6.313	556.293	6.461	1.88E-02	3.86E-02
OfucSP19	17.513	1589.903	6.504	3.19E-06	8.39E-06
OfuSP93	11.163	1160.857	6.700	9.80E-05	2.35E-04
OfuSP69	2.633	282.127	6.743	5.47E-03	1.22E-02
OfuSP85	7238.377	789861.160	6.770	2.29E-12	7.54E-12
OfuSP43	28.420	4087.850	7.168	4.71E-07	1.30E-06
OfuSP102	26.197	4877.837	7.541	2.16E-07	6.21E-07
OfuSP87	12.087	2326.990	7.589	2.53E-08	7.83E-08
OfuSP83	1.430	311.630	7.768	8.44E-04	1.90E-03
OfuSP24	6.403	17387.563	11.407	1.84E-39	1.32E-38
OfuSP77	0.477	4019.783	13.042	2.84E-14	1.02E-13
OfuSP103	0.001	103.917	16.665	7.95E-03	1.70E-02
OfuSP41	0.937	104529.973	16.768	9.13E-47	9.01E-46
M vs H[b]	M mean	H mean	log2(fc)	PValue	q-value
OfuSPH6	13364.083	0.001	-23.672	3.22E-49	4.01E-48
OfuSP32	5942.600	0.001	-22.503	7.28E-50	9.83E-49
OfuSP1	5270.483	0.001	-22.330	8.23E-42	4.94E-41
OfuSP89	4224.980	0.001	-22.011	4.23E-47	4.29E-46
OfuSPH9	2326.510	0.001	-21.150	2.53E-31	1.02E-30
OfuSP65	729.657	0.001	-19.477	1.25E-34	6.13E-34
OfuSP49	568.753	0.001	-19.117	9.23E-34	4.15E-33
OfuSPH4	560.493	0.001	-19.096	4.80E-27	1.77E-26
OfuSP15	258.307	0.001	-17.979	3.33E-30	1.32E-29
OfuSP21	257.290	0.001	-17.973	4.62E-26	1.66E-25
OfuSP61	237.737	0.001	-17.859	8.32E-29	3.13E-28
OfuSP48	233.693	0.001	-17.834	8.79E-16	2.30E-15
OfuSP19	100.940	0.001	-16.623	3.06E-20	9.54E-20
OfuSP20	75.017	0.001	-16.195	5.42E-20	1.66E-19
OfuSP17	66.520	0.001	-16.022	2.58E-18	7.73E-18
OfucSP16	39.597	0.001	-15.273	8.12E-16	2.16E-15
OfuSP23	35.563	0.001	-15.118	6.34E-15	1.58E-14
OfuSP90	23.353	0.001	-14.511	2.77E-11	6.14E-11
OfuSPH31	16479.313	1.210	-13.733	4.43E-50	6.53E-49
OfuSP78	12.253	0.001	-13.581	5.94E-05	1.00E-04
OfuSP74	50822.750	5.533	-13.165	6.98E-102	3.77E-100
OfuSP72	68144.093	7.893	-13.076	3.12E-53	5.62E-52
OfucSP27	7.370	0.001	-12.847	1.48E-07	2.85E-07
OfuSP22	11058.753	1.973	-12.452	5.26E-45	4.26E-44
OfuSP58	4.940	0.001	-12.270	7.37E-04	1.08E-03
OfuSPH2	4572.590	0.987	-12.178	3.38E-44	2.38E-43
OfuSP2	57599.197	14.800	-11.926	1.21E-35	6.14E-35
OfuSPH1	7067.497	1.973	-11.806	1.35E-51	2.18E-50
OfuSP42	26451.887	7.430	-11.798	5.73E-107	4.64E-105
OfuSPH7	34600.087	9.867	-11.776	4.65E-41	2.60E-40
OfuSPH5	26369.467	7.893	-11.706	1.53E-44	1.12E-43
OfuSP101	6124.633	1.973	-11.600	6.31E-43	4.09E-42
OfuSP28	3.087	0.001	-11.592	4.53E-05	7.80E-05
OfuSP105	18609.047	6.907	-11.396	1.73E-48	1.87E-47

OfuSP66	47496.610	17.760	-11.385	2.16E-31	8.99E-31
OfuSP68	2601.373	0.987	-11.364	4.31E-39	2.33E-38
OfuSP88	2.633	0.001	-11.363	2.30E-05	4.10E-05
OfuSP27	2.563	0.001	-11.324	9.60E-05	1.59E-04
OfuSP51	2.553	0.001	-11.318	5.19E-03	7.19E-03
OfuSP95	2.347	0.001	-11.196	3.29E-02	4.29E-02
OfuSP92	28742.630	13.043	-11.106	2.83E-76	7.63E-75
OfuSP4	3803.537	1.973	-10.913	1.04E-44	8.02E-44
OfuSP29	1.913	0.001	-10.902	3.17E-04	4.99E-04
OfuSP18	338272.223	185.083	-10.836	8.30E-34	3.84E-33
OfuSP16	2996.800	1.973	-10.569	4.86E-43	3.28E-42
OfuSP63	8451.600	5.920	-10.479	2.47E-46	2.22E-45
OfuSP67	62113.357	48.347	-10.327	7.55E-21	2.40E-20
OfuSP99	1235.640	0.987	-10.290	9.43E-32	4.13E-31
OfuSP45	8724.463	10.853	-9.651	7.24E-30	2.79E-29
OfuSP46	6019.820	7.893	-9.575	3.83E-36	2.00E-35
OfuSP75	1686.777	2.497	-9.400	8.73E-43	5.44E-42
OfuSP62	2599.170	3.947	-9.363	2.04E-41	1.18E-40
OfuSP3	64893.103	157.043	-8.691	9.21E-25	3.24E-24
OfuSP47	12533.267	31.573	-8.633	5.83E-18	1.72E-17
OfuSPH15	12304.103	34.533	-8.477	6.18E-18	1.79E-17
OfuSP91	8412.867	24.183	-8.442	4.20E-66	9.72E-65
OfuSP85	7238.377	22.317	-8.341	6.50E-116	1.05E-113
OfucSPH7	350.880	1.973	-7.474	1.44E-22	4.87E-22
OfuSP64	1316.070	7.810	-7.397	1.01E-46	9.62E-46
OfuSP60	780.367	5.233	-7.220	3.98E-49	4.60E-48
OfuSPH25	3100.043	22.617	-7.099	1.66E-80	5.37E-79
OfuSP76	1726.083	13.173	-7.034	6.18E-66	1.25E-64
OfuSP14	477.383	3.947	-6.918	7.82E-34	3.73E-33
OfuSP80	1199.937	10.370	-6.854	5.00E-45	4.26E-44
OfuSP40	6147.523	69.773	-6.461	2.33E-101	9.45E-100
OfuSPH16	42.173	0.987	-5.418	4.21E-17	1.15E-16
OfuSP52	98.147	2.417	-5.344	1.12E-21	3.69E-21
OfuSP94	14.573	0.987	-3.885	1.58E-07	3.00E-07
OfuSPH23	70.050	5.677	-3.625	2.01E-08	3.97E-08
OfuSP56	26.913	2.573	-3.387	1.12E-14	2.76E-14
OfuSP39	222.733	67.657	-1.719	4.71E-14	1.14E-13
OfucSP28	6.113	2.497	-1.292	3.01E-04	4.78E-04
OfuSP25	3.057	1.287	-1.248	2.16E-04	3.46E-04
OfuSP69	2.633	1.287	-1.033	3.76E-05	6.56E-05
OfuSP100	2.343	4.990	1.091	6.43E-04	9.46E-04
OfucSPH1	3.353	8.253	1.299	1.86E-05	3.38E-05
OfucSP29	2.567	7.123	1.473	1.47E-02	1.98E-02
OfuSP7	3.343	10.007	1.582	1.35E-04	2.18E-04
OfucSP21	6.313	30.110	2.254	5.91E-05	1.00E-04
OfuSP50	12.123	80.340	2.728	1.96E-09	4.02E-09
OfuSP93	11.163	76.610	2.779	3.69E-09	7.48E-09
OfuSPH17	9.750	85.093	3.126	6.32E-06	1.18E-05
OfuSP98	2.623	25.973	3.308	3.09E-03	4.36E-03
OfuSPH19	2.120	24.367	3.523	9.80E-03	1.33E-02
OfucSP4	44.983	539.703	3.585	9.49E-05	1.59E-04
OfucSP22	11.320	136.967	3.597	1.93E-02	2.59E-02
OfuSP55	29.430	453.897	3.947	4.79E-03	6.69E-03
OfuSP73	129.447	2669.143	4.366	6.39E-04	9.46E-04

OfuSP43	28.420	905.037	4.993	3.44E-02	4.45E-02
OfucSPH8	241.360	39332.620	7.348	2.58E-02	3.39E-02
OfuSPH14	84.040	21742.630	8.015	1.11E-03	1.60E-03
OfuSP87	12.087	3331.487	8.107	1.53E-03	2.18E-03
OfucSP10	85.020	24962.807	8.198	5.97E-04	8.95E-04
OfuSP37	18.240	5924.137	8.343	7.06E-03	9.69E-03
OfucSP13	10.880	4406.767	8.662	1.34E-04	2.18E-04
OfuSPH28	31.733	13147.523	8.695	3.67E-04	5.66E-04
OfuSP38	175.220	75847.843	8.758	3.95E-04	6.03E-04
OfucSP17	56.623	33063.893	9.190	2.47E-11	5.55E-11
OfuSP13	14.057	9727.047	9.435	2.88E-08	5.62E-08
OfuSP53	38.000	36713.233	9.916	1.05E-10	2.24E-10
OfuSPH10	14.540	14894.287	10.001	2.20E-07	4.14E-07
OfucSP18	1.170	1412.547	10.238	4.68E-04	7.08E-04
OfucSPH3	98.513	130582.923	10.372	1.08E-10	2.28E-10
OfucSP15	23.290	32427.150	10.443	5.79E-21	1.88E-20
OfucSP7	1.393	2012.377	10.496	3.23E-05	5.69E-05
OfuSPH22	13.413	21625.027	10.655	9.81E-12	2.27E-11
OfuSPH12	53.023	91184.860	10.748	5.28E-11	1.16E-10
OfucSP19	17.513	35292.847	10.977	1.43E-15	3.66E-15
OfucSP9	34.710	71541.197	11.009	2.01E-13	4.71E-13
OfucSPH2	7.237	16186.163	11.127	6.01E-11	1.30E-10
OfucSPH4	23.160	60597.620	11.353	9.74E-18	2.77E-17
OfucSPH6	4.700	15367.057	11.675	9.98E-14	2.38E-13
OfuSP31	7.780	30883.233	11.955	7.51E-16	2.03E-15
OfucSP24	18.437	78406.387	12.054	1.67E-31	7.12E-31
OfucSP2	13.633	92120.467	12.722	2.14E-24	7.39E-24
OfuSPH20	1.453	9826.557	12.723	2.81E-10	5.83E-10
M vs FB	M mean	FB mean	log2(fc)	PValue	q-value
OfuSP48	233.693	0.001	-17.834	7.23E-08	2.46E-07
OfuSP23	35.563	0.001	-15.118	3.07E-05	8.05E-05
OfuSP78	12.253	0.001	-13.581	1.97E-02	3.71E-02
OfuSP72	68144.093	610.380	-6.803	2.68E-34	1.43E-32
OfuSP74	50822.750	473.910	-6.745	2.06E-38	3.29E-36
OfuSPH9	2326.510	29.270	-6.313	4.73E-16	5.04E-15
OfuSPH2	4572.590	61.530	-6.216	5.08E-20	2.03E-18
OfuSPH5	26369.467	562.467	-5.551	1.32E-19	4.22E-18
OfuSP46	6019.820	138.753	-5.439	2.05E-19	4.85E-18
OfuSP105	18609.047	468.970	-5.310	2.11E-18	3.76E-17
OfuSP60	780.367	20.217	-5.271	3.23E-12	1.99E-11
OfuSP15	258.307	6.740	-5.260	1.74E-09	7.33E-09
OfuSP21	257.290	6.740	-5.255	6.79E-09	2.65E-08
OfuSPH31	16479.313	441.380	-5.223	4.80E-17	5.48E-16
OfuSP47	12533.267	356.477	-5.136	1.03E-10	4.98E-10
OfuSP2	57599.197	1646.827	-5.128	3.23E-13	2.25E-12
OfuSPH15	12304.103	407.543	-4.916	5.05E-18	8.08E-17
OfuSPH4	560.493	18.973	-4.885	3.92E-12	2.32E-11
OfuSP18	338272.223	14044.837	-4.590	3.54E-14	2.83E-13
OfuSP99	1235.640	54.277	-4.509	6.94E-18	1.01E-16
OfuSP1	5270.483	232.473	-4.503	5.73E-11	2.86E-10
OfuSP45	8724.463	391.563	-4.478	1.87E-15	1.76E-14
OfuSP63	8451.600	416.910	-4.341	1.99E-12	1.27E-11
OfuSP3	64893.103	3710.537	-4.128	7.57E-12	4.20E-11
OfuSP91	8412.867	503.220	-4.063	1.66E-15	1.66E-14

OfuSP40	6147.523	430.363	-3.836	2.77E-19	5.53E-18
OfuSP32	5942.600	418.317	-3.828	4.11E-10	1.88E-09
OfuSPH7	34600.087	2490.717	-3.796	2.24E-09	9.19E-09
OfuSP92	28742.630	2149.427	-3.741	1.15E-11	5.95E-11
OfuSP85	7238.377	700.187	-3.370	6.81E-08	2.37E-07
OfuSP42	26451.887	2572.200	-3.362	7.04E-10	3.05E-09
OfuSP61	237.737	27.647	-3.104	1.83E-06	5.34E-06
OfuSPH1	7067.497	877.423	-3.010	2.03E-07	6.62E-07
OfuSP67	62113.357	7778.260	-2.997	2.19E-08	8.16E-08
OfuSP66	47496.610	6623.037	-2.842	2.13E-07	6.81E-07
OfuSPH6	13364.083	1902.897	-2.812	7.76E-14	5.64E-13
OfuSP89	4224.980	643.173	-2.716	4.74E-05	1.20E-04
OfuSP62	2599.170	435.373	-2.578	3.47E-07	1.07E-06
OfuSP64	1316.070	236.193	-2.478	6.66E-06	1.84E-05
OfuSP68	2601.373	550.427	-2.241	1.50E-04	3.65E-04
OfuSP75	1686.777	373.617	-2.175	1.66E-08	6.34E-08
OfuSP52	98.147	23.233	-2.079	2.26E-06	6.46E-06
OfuSPH25	3100.043	817.613	-1.923	7.69E-07	2.28E-06
OfuSP16	2996.800	982.387	-1.609	4.21E-04	9.23E-04
OfuSP76	1726.083	685.917	-1.331	5.80E-07	1.75E-06
OfuSP19	100.940	40.430	-1.320	2.93E-04	6.61E-04
OfuSP80	1199.937	484.317	-1.309	3.61E-05	9.31E-05
OfuSP101	6124.633	2792.290	-1.133	1.33E-04	3.26E-04
OfuSP22	11058.753	5513.930	-1.004	5.90E-04	1.28E-03
OfucSPH6	4.700	227.567	5.598	1.71E-02	3.25E-02
OfucSPH2	7.237	505.083	6.125	7.71E-03	1.50E-02
OfuSP24	6.403	542.090	6.404	1.59E-03	3.34E-03
OfucSP19	17.513	1573.443	6.489	1.83E-04	4.37E-04
OfuSP54	8.250	746.740	6.500	4.18E-03	8.36E-03
OfucSPH8	241.360	23486.643	6.605	2.46E-05	6.55E-05
OfuSP93	11.163	1166.403	6.707	9.39E-05	2.35E-04
OfuSP25	3.057	410.670	7.070	2.18E-04	5.13E-04
OfucSP2	13.633	2533.780	7.538	1.31E-05	3.56E-05
OfuSP53	38.000	8028.020	7.723	1.32E-07	4.42E-07
OfuSPH20	1.453	307.250	7.724	3.82E-04	8.50E-04
OfucSP11	66.473	14606.220	7.780	4.22E-06	1.19E-05
OfucSP10	85.020	26020.860	8.258	5.44E-08	1.94E-07
OfucSP25	56.913	20489.110	8.492	7.61E-12	4.20E-11
OfuSP86	35.990	13480.213	8.549	2.35E-09	9.42E-09
OfucSP18	1.170	476.783	8.671	2.36E-04	5.47E-04
OfucSP15	23.290	9656.507	8.696	9.03E-12	4.81E-11
OfucSPH3	98.513	51511.220	9.030	4.78E-08	1.74E-07
OfucSP17	56.623	30044.073	9.052	5.80E-14	4.42E-13
OfuSPH14	84.040	46456.707	9.111	1.58E-14	1.41E-13
OfuSPH12	53.023	32890.653	9.277	2.21E-07	6.94E-07
OfucSPH4	23.160	16930.967	9.514	7.04E-10	3.05E-09
OfuSP37	18.240	23004.063	10.301	1.12E-10	5.29E-10
OfucSP16	39.597	52076.620	10.361	3.48E-13	2.32E-12
OfucSP24	18.437	29949.620	10.666	2.69E-14	2.27E-13
OfuSP55	29.430	64295.163	11.093	2.47E-17	3.04E-16
OfucSP9	34.710	109906.117	11.629	1.10E-17	1.47E-16
OfuSPH13	2.110	8054.007	11.898	2.12E-19	4.85E-18
OfuSP43	28.420	313536.287	13.429	8.32E-36	6.65E-34
OfuSP103	0.001	77.153	16.235	2.71E-03	5.57E-03

SG vs H	SG mean	H mean	log2(fc)	PValue	q-value
OfuSP77	4019.783	0.001	-21.939	4.88E-28	1.41E-26
OfuSPH9	1644.177	0.001	-20.649	2.56E-20	5.28E-19
OfucSP16	325.020	0.001	-18.310	5.21E-12	5.36E-11
OfuSP78	244.220	0.001	-17.898	3.00E-11	2.54E-10
OfuSP103	103.917	0.001	-16.665	4.66E-07	1.81E-06
OfuSPH27	86.203	0.001	-16.396	6.42E-06	2.05E-05
OfuSP90	85.333	0.001	-16.381	1.06E-05	3.12E-05
OfuSPH6	83.357	0.001	-16.347	1.29E-04	3.00E-04
OfuSP1	60.080	0.001	-15.875	5.69E-04	1.19E-03
OfucSP27	50.270	0.001	-15.617	8.79E-04	1.81E-03
OfuSP27	49.403	0.001	-15.592	3.79E-03	6.91E-03
OfuSP41	104529.973	2.417	-15.401	2.19E-50	1.57E-48
OfuSP85	789861.160	22.317	-15.111	3.66E-142	5.27E-140
OfuSP32	26.123	0.001	-14.673	1.39E-02	2.38E-02
OfucSPH7	9436.540	1.973	-12.223	3.42E-31	1.23E-29
OfuSPH16	368.413	0.987	-8.545	1.67E-12	1.85E-11
OfuSP69	282.127	1.287	-7.777	5.24E-11	4.20E-10
OfuSPH23	752.717	5.677	-7.051	1.23E-16	1.78E-15
OfuSPH31	157.487	1.210	-7.024	1.13E-07	5.05E-07
OfuSPH21	1021.823	8.477	-6.913	1.42E-23	3.41E-22
OfuSP24	17387.563	163.357	-6.734	4.54E-32	2.18E-30
OfuSPH30	102.523	0.987	-6.699	1.44E-05	4.07E-05
OfuSPH34	241.477	2.573	-6.552	1.80E-02	2.92E-02
OfuSP68	71.863	0.987	-6.187	2.83E-04	6.27E-04
OfucSP1	757.937	10.590	-6.161	9.24E-16	1.21E-14
OfuSP42	511.873	7.430	-6.106	5.98E-12	5.74E-11
OfuSPH1	135.027	1.973	-6.097	1.30E-06	4.68E-06
OfuSP63	382.783	5.920	-6.015	3.34E-08	1.60E-07
OfuSPH2	62.053	0.987	-5.975	1.31E-04	3.00E-04
OfuSP105	421.323	6.907	-5.931	9.07E-10	5.44E-09
OfuSP4	119.580	1.973	-5.921	4.34E-05	1.10E-04
OfuSPH7	559.143	9.867	-5.825	2.30E-10	1.65E-09
OfuSP22	104.207	1.973	-5.723	8.52E-06	2.67E-05
OfuSP72	408.877	7.893	-5.695	3.26E-10	2.13E-09
OfuSP66	719.110	17.760	-5.340	1.16E-07	5.05E-07
OfucSP28	98.567	2.497	-5.303	5.65E-05	1.40E-04
OfuSP74	180.187	5.533	-5.025	5.27E-07	2.00E-06
OfuSPH5	245.090	7.893	-4.957	4.26E-07	1.71E-06
OfuSP75	75.240	2.497	-4.913	4.79E-04	1.01E-03
OfuSP96	36.220	1.210	-4.904	1.42E-02	2.40E-02
OfuSP67	1365.113	48.347	-4.820	4.21E-07	1.71E-06
OfuSP45	252.627	10.853	-4.541	1.43E-05	4.07E-05
OfuSPH26	52.250	2.273	-4.523	9.92E-04	2.01E-03
OfucSP29	156.460	7.123	-4.457	2.35E-06	8.25E-06
OfuSP92	282.707	13.043	-4.438	9.91E-09	5.09E-08
OfucSP21	556.293	30.110	-4.208	2.79E-10	1.92E-09
OfuSP93	1160.857	76.610	-3.922	4.76E-18	8.56E-17
OfuSP2	220.073	14.800	-3.894	1.14E-04	2.74E-04
OfuSP79	120.447	8.253	-3.867	6.20E-05	1.51E-04
OfuSP39	912.603	67.657	-3.754	1.46E-12	1.76E-11
OfuSP46	83.067	7.893	-3.396	1.52E-02	2.49E-02
OfuSP100	49.403	4.990	-3.308	1.47E-02	2.46E-02
OfucSPH9	66.010	7.490	-3.140	1.38E-03	2.69E-03

OfucSPH1	71.283	8.253	-3.111	2.12E-03	4.02E-03
OfuSP91	194.290	24.183	-3.006	1.66E-05	4.61E-05
OfuSPH15	248.913	34.533	-2.850	1.29E-02	2.24E-02
OfuSP30	117.393	17.957	-2.709	3.49E-04	7.62E-04
OfuSP50	510.900	80.340	-2.669	2.08E-11	1.87E-10
OfucSP3	51.670	8.493	-2.605	2.00E-02	3.20E-02
OfuSPH19	137.877	24.367	-2.500	1.03E-05	3.09E-05
OfuSP40	341.337	69.773	-2.290	8.60E-08	4.00E-07
OfuSP43	4087.850	905.037	-2.175	1.55E-10	1.17E-09
OfuSP55	1651.990	453.897	-1.864	6.85E-09	3.65E-08
OfuSP84	96.010	27.553	-1.801	1.51E-02	2.49E-02
OfuSP3	541.560	157.043	-1.786	2.63E-03	4.92E-03
OfuSPH17	249.047	85.093	-1.549	9.76E-06	2.99E-05
OfuSP102	4877.837	2257.523	-1.112	4.03E-06	1.35E-05
OfucSP11	3578.457	7393.990	1.047	1.12E-02	1.96E-02
OfuSPH14	1287.167	21742.630	4.078	4.47E-03	8.05E-03
OfuSPH10	730.713	14894.287	4.349	3.23E-03	5.96E-03
OfucSP17	1531.330	33063.893	4.432	4.21E-05	1.08E-04
OfucSP19	1589.903	35292.847	4.472	1.05E-03	2.09E-03
OfucSP15	1239.813	32427.150	4.709	6.15E-06	2.01E-05
OfuSP38	2550.227	75847.843	4.894	1.21E-04	2.86E-04
OfucSPH3	3893.950	130582.923	5.068	2.00E-05	5.32E-05
OfuSP53	998.603	36713.233	5.200	1.84E-05	4.99E-05
OfucSPH2	392.110	16186.163	5.367	1.99E-04	4.47E-04
OfucSP9	1693.437	71541.197	5.401	1.24E-06	4.57E-06
OfuSPH28	281.970	13147.523	5.543	3.88E-05	1.02E-04
OfuSPH22	449.133	21625.027	5.589	3.85E-04	8.27E-04
OfuSP31	462.787	30883.233	6.060	2.60E-06	8.93E-06
OfucSPH4	867.793	60597.620	6.126	1.74E-09	1.00E-08
OfucSP2	1116.837	92120.467	6.366	8.69E-10	5.44E-09
OfuSPH12	973.447	91184.860	6.550	2.32E-09	1.28E-08
OfucSP24	618.773	78406.387	6.985	2.33E-17	3.73E-16
OfucSP7	11.783	2012.377	7.416	1.07E-03	2.11E-03
OfucSPH6	74.130	15367.057	7.696	1.36E-08	6.76E-08
OfuSPH20	14.050	9826.557	9.450	4.10E-07	1.71E-06
SG vs FB	SG mean	FB mean	log2(fc)	PValue	q-value
OfuSP41	104529.973	0.001	-26.639	6.05E-29	9.14E-27
OfuSP78	244.220	0.001	-17.898	6.43E-05	4.62E-04
OfuSPH30	102.523	0.001	-16.646	7.49E-03	2.76E-02
OfuSP85	789861.160	700.187	-10.140	4.38E-24	3.30E-22
OfuSP77	4019.783	25.893	-7.278	8.53E-14	4.30E-12
OfuSP87	2326.990	38.130	-5.931	3.26E-09	6.14E-08
OfuSPH9	1644.177	29.270	-5.812	2.64E-09	5.70E-08
OfuSPH19	137.877	3.017	-5.514	3.64E-03	1.77E-02
OfuSP24	17387.563	542.090	-5.003	2.73E-12	8.24E-11
OfuSP69	282.127	9.757	-4.854	2.68E-04	1.69E-03
OfuSPH21	1021.823	58.743	-4.121	3.22E-05	2.43E-04
OfuSP83	311.630	36.537	-3.092	7.07E-03	2.74E-02
OfucSP1	757.937	112.503	-2.752	5.05E-03	2.18E-02
OfucSP21	556.293	94.397	-2.559	6.84E-03	2.72E-02
OfuSP73	1757.440	325.443	-2.433	2.95E-03	1.48E-02
OfuSP102	4877.837	1177.067	-2.051	7.42E-03	2.76E-02
OfuSP39	912.603	223.933	-2.027	1.54E-03	8.61E-03
OfucSPH7	9436.540	2493.493	-1.920	1.48E-04	1.01E-03

OfuSP13	1118.313	364.687	-1.617	5.83E-03	2.38E-02
OfucSP15	1239.813	9656.507	2.961	1.22E-02	4.40E-02
OfucSPH3	3893.950	51511.220	3.726	1.78E-03	9.60E-03
OfucSPH4	867.793	16930.967	4.286	2.00E-04	1.32E-03
OfucSP17	1531.330	30044.073	4.294	1.29E-05	1.09E-04
OfucSP25	942.477	20489.110	4.442	1.30E-05	1.09E-04
OfuSPH6	83.357	1902.897	4.513	1.84E-03	9.60E-03
OfucSP4	82.487	2008.310	4.606	4.53E-03	2.07E-02
OfuSPH12	973.447	32890.653	5.078	2.87E-05	2.28E-04
OfuSPH14	1287.167	46456.707	5.174	7.86E-07	8.10E-06
OfuSP55	1651.990	64295.163	5.282	3.51E-07	4.82E-06
OfucSP24	618.773	29949.620	5.597	2.29E-07	3.45E-06
OfuSP86	275.037	13480.213	5.615	5.16E-07	6.49E-06
OfuSP37	448.793	23004.063	5.680	6.96E-07	8.09E-06
OfuSP22	104.207	5513.930	5.726	3.93E-03	1.85E-02
OfucSP9	1693.437	109906.117	6.020	3.04E-08	5.10E-07
OfuSP26	47.137	3502.330	6.215	3.45E-04	2.08E-03
OfuSP43	4087.850	313536.287	6.261	1.50E-11	3.79E-10
OfuSPH13	93.823	8054.007	6.424	2.44E-06	2.30E-05
OfuSP101	23.567	2792.290	6.889	1.15E-03	6.66E-03
OfucSP16	325.020	52076.620	7.324	1.72E-13	6.48E-12
OfuSP76	0.001	685.917	19.388	5.78E-03	2.38E-02
OfuSPH25	0.001	817.613	19.641	4.67E-03	2.07E-02
OfuSP49	0.001	3301.127	21.655	8.05E-07	8.10E-06
FB vs H	FB mean	H mean	log2(fc)	PValue	q-value
OfucSP16	52076.620	0.001	-25.634	2.70E-35	1.27E-33
OfuSP49	3301.127	0.001	-21.655	6.76E-12	4.76E-11
OfuSPH6	1902.897	0.001	-20.860	6.13E-13	5.40E-12
OfuSP32	418.317	0.001	-18.674	2.10E-04	6.89E-04
OfuSP1	232.473	0.001	-17.827	1.09E-03	2.94E-03
OfuSP27	133.907	0.001	-17.031	1.70E-07	8.27E-07
OfuSP28	114.533	0.001	-16.805	3.07E-05	1.20E-04
OfuSP90	99.670	0.001	-16.605	2.53E-02	4.89E-02
OfuSP103	77.153	0.001	-16.235	1.25E-05	5.49E-05
OfucSP27	39.370	0.001	-15.265	8.97E-04	2.48E-03
OfuSP101	2792.290	1.973	-10.467	2.48E-06	1.17E-05
OfucSPH7	2493.493	1.973	-10.303	2.53E-12	1.88E-11
OfuSPH16	1076.070	0.987	-10.091	1.15E-11	7.34E-11
OfuSPH31	441.380	1.210	-8.511	2.43E-05	9.79E-05
OfuSP43	313536.287	905.037	-8.436	1.98E-49	2.80E-47
OfuSP25	410.670	1.287	-8.318	2.25E-09	1.32E-08
OfuSP75	373.617	2.497	-7.225	4.46E-05	1.67E-04
OfuSP55	64295.163	453.897	-7.146	5.84E-20	8.23E-19
OfuSP62	435.373	3.947	-6.786	2.09E-03	5.16E-03
OfuSP14	409.263	3.947	-6.696	1.86E-02	3.75E-02
OfuSP74	473.910	5.533	-6.420	2.39E-09	1.35E-08
OfuSP72	610.380	7.893	-6.273	1.03E-04	3.73E-04
OfuSPH5	562.467	7.893	-6.155	1.42E-03	3.70E-03
OfuSP63	416.910	5.920	-6.138	3.17E-03	7.71E-03
OfuSP105	468.970	6.907	-6.085	3.59E-04	1.10E-03
OfuSPH2	61.530	0.987	-5.963	9.66E-03	2.06E-02
OfuSP99	54.277	0.987	-5.782	1.19E-03	3.17E-03
OfuSP76	685.917	13.173	-5.702	2.33E-04	7.47E-04
OfuSP80	484.317	10.370	-5.546	1.93E-04	6.49E-04

OfuSP33	38.130	0.987	-5.272	2.48E-02	4.86E-02
OfuSPH25	817.613	22.617	-5.176	5.60E-04	1.65E-03
OfuSP45	391.563	10.853	-5.173	5.05E-03	1.19E-02
OfuSP85	700.187	22.317	-4.972	3.95E-04	1.18E-03
OfuSPH13	8054.007	278.810	-4.852	1.59E-14	1.60E-13
OfuSP100	118.830	4.990	-4.574	1.52E-02	3.11E-02
OfuSP91	503.220	24.183	-4.379	6.09E-04	1.75E-03
OfuSP46	138.753	7.893	-4.136	2.07E-02	4.12E-02
OfuSP93	1166.403	76.610	-3.928	1.24E-14	1.35E-13
OfuSP79	100.710	8.253	-3.609	8.05E-04	2.27E-03
OfucSP1	112.503	10.590	-3.409	3.74E-03	8.94E-03
OfuSP30	156.403	17.957	-3.123	9.65E-03	2.06E-02
OfuSP86	13480.213	1698.770	-2.988	2.86E-09	1.55E-08
OfuSP40	430.363	69.773	-2.625	3.44E-04	1.08E-03
OfuSP97	148.447	30.220	-2.296	1.12E-02	2.36E-02
OfucSP25	20489.110	4688.073	-2.128	4.07E-06	1.85E-05
OfuSP50	336.047	80.340	-2.065	7.49E-03	1.68E-02
OfuSP37	23004.063	5924.137	-1.957	6.38E-03	1.47E-02
OfuSPH14	46456.707	21742.630	-1.095	8.21E-03	1.81E-02
OfucSP24	29949.620	78406.387	1.388	1.40E-02	2.89E-02
OfucSP15	9656.507	32427.150	1.748	4.50E-05	1.67E-04
OfucSPH4	16930.967	60597.620	1.840	2.00E-03	5.03E-03
OfuSP53	8028.020	36713.233	2.193	2.28E-05	9.58E-05
OfucSP14	403.887	2492.963	2.626	1.42E-10	8.73E-10
OfuSP73	325.443	2669.143	3.036	5.12E-13	4.81E-12
OfuSPH28	1107.333	13147.523	3.570	3.63E-08	1.83E-07
OfucSP5	10.643	144.097	3.759	1.83E-03	4.68E-03
OfucSP13	309.977	4406.767	3.830	8.03E-12	5.39E-11
OfuSP83	36.537	552.957	3.920	1.13E-04	4.00E-04
OfucSP19	1573.443	35292.847	4.487	6.73E-15	7.90E-14
OfuSPH10	612.150	14894.287	4.605	2.37E-33	8.36E-32
OfucSP7	80.543	2012.377	4.643	2.68E-08	1.40E-07
OfuSP13	364.687	9727.047	4.737	2.84E-21	4.45E-20
OfuSPH20	307.250	9826.557	4.999	9.09E-13	7.54E-12
OfucSPH2	505.083	16186.163	5.002	1.22E-30	2.86E-29
OfucSP2	2533.780	92120.467	5.184	2.24E-12	1.76E-11
OfuSPH22	422.783	21625.027	5.677	1.33E-36	9.40E-35
OfuSP38	1336.470	75847.843	5.827	5.40E-33	1.52E-31
OfucSPH6	227.567	15367.057	6.077	5.86E-25	1.03E-23
OfuSP87	38.130	3331.487	6.449	1.74E-17	2.23E-16
OfuSP31	270.207	30883.233	6.837	5.40E-25	1.03E-23
OfucSPH5	0.001	103.557	16.660	1.16E-04	4.00E-04
OfuSP59	0.001	152.060	17.214	2.31E-05	9.58E-05

[a]: The method to obtain q-value is based on Benjamini & Hochberg (BH) method[6,7].

[b]: Adequate hemolymph samples were obtained by cutting away the proleg of the 3rd instar larvae. Then the hemolymph bled onto Parafilm and were collected. The fat body is mainly distributed above the intestinal tract. We cut at the front of proleg and only collected hemolymph gushing out initially to ensure the samples were not contaminated by floating fat body.

The newick format of the phylogenetic tree in Figure 2.

(AmcSP1:0.373203186,(((((((((((((AmcSP10:0.0535965729,AmcSP9:0.0269348437):0.55989 58316,((((TccSP142:0.1701598525,TccSP173:0.1292699914):0.152986534,TccSP60:0.37498

99251):0.069243791,TccSP56:0.3121652823):0.1542078086,TccSP61:0.5211352349):0.1325
567102):0.0913051218,((((DmcSP28_Psh:0.2601256823,DmcSP31_Hayan:0.1214049814):0.
4876821474,TccSP66:0.4438516705):0.1260807905,(MsHP6:0.2542375257,OfucSP15:0.355
1545442):0.3143583712):0.1247648303,((((((MsHP13:0.4257676462,MsHP2:0.2795134586)
:0.2885294302,MsSP144:0.2469942765):0.0540947771,(MsHP21:0.0809015045,MsHP22:0.
1940166808):0.1894487759):0.0862984254,OfucSP11:0.3662532121):0.1340054947,((MsH
P18a:0.0084888089,MsHP18b:2.6331E6):0.3226622508,MsSP33:0.3513820677):0.1262342
488):0.2728103255,(TccSP177:0.3555336916,(TccSPH174:0.4472392115,TccSPH176:0.164
8956419):0.1452628232):0.2212064231):0.1143959054):0.0513946519):0.0503818089,(((M
sHP28:0.0946189156,MsSP30:0.2135833777):0.0452814685,OfucSP13:0.1818746048):0.01
2158313,OfucSP14:0.2171776283):0.4120903996):0.0904223288,(((DmcSP115:2.6331E6,D
mcSP26_snk:2.6331E6):0.8730583644,DmcSP33_spirit:0.3635001107):0.1822467596,(Dmc
SP42:0.2278525527,DmcSP48:0.1760928298):0.718653514):0.0900802326):0.0441787781,
TccSP44:0.3890992421):0.1557806909,AmcSP14:0.8735066448):0.1947926756,((((((((Amc
SP16:0.0474350604,((MsSP132:2.6331E6,OfucSP29:0.025282989):0.0497445443,TccSP126
:0.0609349088):0.0335105738):0.0193502953,DmcSP56_Sb:0.0599978837):0.2920996232,(
((AmcSP25:0.1191173657,DmcSP59_Np:0.0594411823):0.031188543,(MsSP143:0.0568847
754,OfucSP21:0.0392852802):0.0803790652):0.0463108487,TccSP86:2.2471E6):0.5425929
044):0.1731482223,(((AmcSP6a:0.1019386085,(MsSP131:0.0088657936,OfucSP27:0.00802
96817):0.0555644135):0.0341156194,DmcSP32:0.1261979427):0.0324583396,TccSP85:0.03
21104111):0.4254164018):0.1175211869,(((AmcSP33:0.1001179413,(MsSP142:0.10722692
3,OfucSP28:0.0304742416):0.0639152061):0.0287458199,DmcSP44:0.0691586768):0.0641
596534,TccSP87:0.0430286352):0.4725138748):0.176418647,((AmcSP26:0.0775061415,((D
mcSP54:0.1147272106,MsSP141:0.0940334825):0.0490148575,TccSP83:0.1010311856):0.0
15003446):0.2794425025,((DmcSP67:0.2499470339,(MsSP140:0.0479194442,OfucSP22:0.0
192723624):0.2033881964):0.1227292047,TccSP84:0.3646650159):0.518551936):0.4185320
006):0.1462170899,(((((AmcSP21:0.177481189,TccSP172:0.1129903349):0.0388450784,(D
mcSP19:0.1595069947,(MsSP52:0.0025813829,OfucSP3:0.0768225201):0.2881152146):0.0
270827599):0.4863473152,((AmcSP8:0.2485633133,DmcSP34:0.319377064):0.0784416793
,(((MsHP1a:0.0181444578,MsHP1b:0.1084849134):0.1543750456,OfucSP2:0.0749005112):
0.2731853996,TccSP52:0.571171022):0.0546579911):0.4862401407):0.0233830181,(AmcSP
7:0.0414631259,((DmcSP36:0.2837842886,(MsSP60:0.0681999135,OfucSP1:0.0871375746)
:0.0981777528):0.0517576608,TccSP53:0.1509081227):0.0196379448):0.4143104533):0.116
0560709,(((AmcSP3:0.3030089787,(((MsHP17a:0.022965764,MsHP17b:0.1630218798):0.1
290494207,OfucSP4:0.1719499521):0.1363839797,((((OfucSP5:0.024243512,OfucSP6:0.06
8642213):0.0242074404,OfucSP7:0.0286717631):0.0546360955,OfucSP8:0.0346866738):0.
4086644043,(OfucSP12:0.092359027,OfucSP20:0.0882495995):0.5000135261):0.35468975
07):0.1374499469):0.0704083886,(DmcSP18:0.4639446687,DmcSP232:0.6673675658):0.09
15309213):0.0556464022,(TccSP140:0.3309919334,TccSP55:0.2338189815):0.3164530207)
:0.1852576628):0.1069722221):0.058748367,((((AmcSPH19:0.1811724544,(DmcSPH66:0.2
295337555,TccSPH82:0.133639381):0.0954681664):0.1720303351,((AmcSPH37:0.3617443
167,DmcSPH101:0.2893094339):0.062310632,OfucSPH1:0.3820473161):0.3577943044):0.0
82592834,(AmcSPH41:0.0464686316,(DmcSPH79_mas:0.0247851607,((MsSPH53:0.00818
11319,OfucSPH9:0.0338543558):0.0166155144,TccSPH51:0.0080985748):0.00819561):0.01

18970734):0.9564145689):0.149586282,(AmcSPH39:0.6001026636,(((((((AmcSPH42:0.239
0526982,((MsSPH101:2.6508E-6,(MsSPH1a:0.097565331,(MsSPH1b:2.6331E-
6,MsSPH4:0.0250899316):2.639E-
6):0.016674585):0.1696745826,(OfucSPH4:0.086648937,OfucSPH6:0.4808077813):0.94153
80717):0.1429496214):0.0500201107,TccSPH2:0.2532467968):0.0406026195,(((TccSPH28:
0.215218409,TccSPH78:0.4717706218):0.0384133071,OfucSPH2:0.6789932745):0.0662139
651,((TccSPH29:0.3740032477,OfucSPH3:0.8405067957):0.087561422,TccSPH5:0.5058943
361):0.04043789):0.0934401781):0.090049183,(DmcSPH242:0.370990467,(MsSPH2:0.1771
474184,OfucSPH8:0.2221909522):0.2921942634):0.1039569217):0.0368988541,(DmcSPH3
5:0.3225893216,(TccSPH3:0.2159120119,TccSPH4:0.2639001316):0.1603887918):0.089925
9696):0.0879497839,(((TccSPH125:0.278233186,TccSPH59:0.4065978176):0.2820953333,T
ccSPH30:0.4631659923):0.0401888985,TccSPH34:0.8579083504):0.0620500549):0.060253
2644,((((AmcSPH55:0.9177884865,((DmcSPH142_scaf:1.02803094,OfucSPH7:0.51760433
2):0.1571762165,TccSPH164:0.4242441369):0.2567803456):0.9164567608,DmcSPH166:1.4
313130588):0.1684055941,((DmcSPH121:0.3197448965,DmcSPH58:0.3113429736):0.1828
393403,((DmcSPH125:0.7884790409,((DmcSPH156:0.7669684603,DmcSPH93:0.32480300
2):0.0824788856,DmcSPH94:0.5678483933):0.1831192435):0.0247103533,(DmcSPH128:0.
3672226045,(DmcSPH231:2.6331E-6,DmcSPH69:2.6331E-
6):0.4586822162):0.1614279902):0.2363018668):0.051208906):0.0227846051,(TccSPH1:0.5
074085205,TccSPH6:0.44134405):0.1536657479):0.0226870931):0.1502911341):0.1146002
324):0.227450921):0.0684943402):0.0538289263,(DmcSP12:1.0938052442,TccSP95:0.5226
966563):0.2109739384):0.0422260771,(((((((((AmcSP2:0.3502196342,((TccSP136:0.137390
7118,TccSP138:0.4773140008):0.0392454401,TccSP137:0.2201251199):0.0501633438):0.03
51616545,(MsHP8:0.232100222,OfucSP17:0.1323304505):0.3051941355):0.0862698928,Tc
cSP8:0.3072388324):0.0438208034,(MsPAP1:0.147287258,OfucSP26:0.1708095468):0.388
8510828):0.0929260758,(((((((DmcSP14:0.5221076677,DmcSP61:0.5031766778):2.6284E-
6,(DmcSP16:0.509209931,DmcSP38:0.3408204115):0.1064412308):0.1289906247,DmcSP2
29:0.4276074512):0.2135708056,DmcSP4_SPE:0.3325137234):0.158213685,DmcSP3_Ser7:
0.8307089662):0.0262616701,(DmcSPH9:0.5215769919,TccSPH35:0.9031903318):0.19884
4939):0.0197293193,(DmcSP24_ea:0.3360680681,DmcSP25_MP1:0.3617441437):0.093034
646):0.1549150872):0.0806406036,((DmcSP5:0.4766121943,((TccSP10:0.6076457431,TccS
P175:0.4544050769):0.1963393672,(TccSP171:0.5131711812,TccSP7:0.350222187):0.21547
41891):0.0220263733):0.1391083822,OfucSP25:0.7878821172):0.0751616143):0.03441449
01,((DmcSP10:0.2250420612,DmcSP11:0.4044548068):0.3036308307,DmcSP7_MP2:0.338
7075196):0.165346579):0.0742130801,(((((MsGP33:0.2636895052,(MsHP15:0.0685579088,
MsHP23:0.0798318622):0.0491301645):0.0762351813,((MsHP12:0.0665205182,(MsHP24:0
.02606788,MsHP26:2.6331E6):0.1167224805):0.1721930099,MsPAP2:0.1923611911):0.060
9847037):0.1946484277,((MsPAP3:0.1953464001,(OfucSP10:0.0203084344,(OfucSP18:0.26
70357846,OfucSP19:0.1282761425):0.0367274005):0.1283188034):0.1473284643,OfucSP2
4:0.5455730182):0.1026643744):0.1716129704,OfucSP9:0.338233067):0.0690306634,Ofuc
SP23:0.7949058632):0.1077920963):0.065077084,(((AmcSPH50:0.9867780069,TccSPH99:0
.1545074025):0.2513978643,(MsSPH42:0.1985109642,OfucSPH5:0.1830142165):0.288289
701):0.1518012386,DmcSPH64:0.4408862089):0.6076678905):0.0754336534):0.099370876
4,(DmcSP1_grass:0.5894959712,DmcSP8:0.8501805772):0.1929538586):0.1537060634,(Tc

cSP33:0.3998911776,TccSP90:0.3029217937):0.1636580057):0.0676267134,(((MsGP6:0.27 7378995,MsHP5:0.1104631174):0.1248653157,OfucSP16:0.1726188618):0.2026815852,((Tc cSP91:0.0559670308,TccSP92:0.3353055178):0.1350482338,(TccSP93:0.2241507344,TccSP 94:0.2175941758):0.0340531171):0.2158547569):0.0983455537);

The amino acid sequences of 177 *Ostrinia furnacalis* SPs and SPHs.

>OfucSP1

MNKMAMGTKLLILWMGLLALTCGSDSENSNGFSPKDGAFYTDDAVIINAAVSRARRDTTNST
RDGKQLLYFQTRSQTDGAFGMDCETTLGKKGTCKSFRECYPLIKVVDVSGVDGWVMGHYDT
CSYISADNMEVFGVCCTEPVGTPPQQEPDVQRLGILPAAAPMQLPPMQLAGLQRGVLSALSAQ
WPFGTNFNFRQIPANWPPTIPPLPTHPPDHTAPTHPPSLVAGVVTTRPPSATTSQTTWATRPQMTS
KPXKPTTKQTWPPMYPTQPSKPTQPQPVVDFSCGNKNAKIKEDERIVGGHNAELNEWPWIVAL
FNGGRQFCGGSLMDDRHVLSAAHCVAHMTSWDVARLTARLGDHNIHSNAETQHIERKIKRVV
RHRGFDMRTLYNDIAILTLDQPVKFTKAVRPVCLPSGGRAYAGLMATVIGWGSLRESGPQPATL
QEVTIPIWSNAECRLKYGAAAPGGIVEHMLCAGKANMDSCSGDSGGPLMVNEGGRWTQVGV
VSWGIGCGKGQYPGVYTRVTAFLPWIQKNSK

>OfucSP2

MISAELSVVNILRISLSASGGPRGLTDRVEEPTMLLVLVLCVAVVGVQGDGFQSQFMDPAWEEV
FSAGGILIGSGRAKRFVEMNANQPNEPHQACLLPNGKAGHCRHLHFCVQEDFKKDFTKFMDY
LCIIQRTSIGVCCPDELADLREASLAGDLPATAPREEANEAILKVTRAENRGCGLSTRPHGRVTG
ARPANPREWPWMASITPVGFEQYCGGALITDRHVLTAAHCTRRWKAEDLHVRLGEYDFHRTN
DSRSYDFRVVEIRQHVDFELPSYHHDIAILKLHRPAVFNTYVWPICLPPMGMDITDQNAIAIGW
GTQWYGGPHSTTLMEVTVPVWPQQSCVEAFVDSIFDESICAGGREGGKDSCQGDSGGPLMYQ
MDSGRWTVVGVVSWGKGCGERSHPGIYTRVDRYLGWIVDNVRF

>OfucSP3

MQQGNPPWHQFSSPRKRSPEFAPGHPYYNGEYNTQPTLYNQGPFGPRPDQQNLQRYPQNYES
RSSFDLVNSETRDSPNDGRLLSDSAFTRISETLGAINTVGHYLVDMVNENERDETDPNLKQLPQ
ALYTISKNVLGRNVTDKIAPIVKKALPRVLPDAPITKIATANEKDNTKSCTTPEGEEGICEDLSNC
PQLLLNLVNLRESLCFKDLFVPGVCCPKDAVVLATPLVEKPIVTTTTKPTYLVPVTTQRPQRPQK
PPKPTTTKKPSAILVLTTKRPKPATTTTRPTTTTAVAVTTPRPPPTSTFFTVPPPILTNFSNIVSMEEC
GQREDEGGRIVGGTESKPGAWPWMAAIYLHGSKRREFWCGGTLVNRRHVLTAAHCTRDSKQ
RPFPARQFSVRLGDVDLARDDEPSRPVTHRVTAVRAHEQFSRVGFYNDIALLVLADNVLKSKY
VIPICLPTGELSRQLFDGSLATVVGWGTTRYGGGESSKQLEAKLPVWRNEDCDRAYFQPITETF
LCAGYARGGVDACQGDSGGPLMLQVQGRWTQIGVVSFGNKCGEPGYPGVYTRVTHYNSWL
QQNLL

>OfucSP4

MAYVIKLLCVCLCVSAVTCQFGFFPQREDRRYSTPFQYGLNLLNPRRITNRLTGLLHVMPENEN
TNPAYVYPFRRPGISTENQNTNSYPYQNGQNYNQGNYGYQNQYPGYGYNNQGYNPNYGYNQ
NGGNPVVFPTSPPDGTAVNFVNNNGEYTESNPDINNSGTAVNFVNNNDGQYTESNPSNYYPGQ
FKDPAGNGNYQNNNGDNEQEGKDNVDINLPTGGAESNTPNASPAEIPNIPPPGIPIASPAEIPNVP
SPGIPSSSPAEIPTNPPAGIPNNSPAEIPNVPPPGIPNSPAEIPSVPPAGIPNKPPAEIPNLPPAGIPNAL
STKAPASYTELPVLTPVPDTEKRNNFNFGSAGLFKESCETVDQQIGSCISILQCAPYLQLLKESRT
NPQAVQVLRKSQCGFEGNYPKVCCPRPGIPDAPPTVPPTTTTTTTTVAPPVSNEPSNVNPEDLLG
SFPEPPVCGSSNATFSRVVGGIPAKLGDFPWMALLGYKRGVNTRWLCGGSLISSKHVLTAAHCI
HGHEIDLYLVRLGELDIEREDDGATPVDVLLKYKIKHEDYNPQAFTNDIGILVMDRDVGFTDLI
KPICIPKDSELRARSFENYTPIIAGWGDTEFRGPSASHLQVLQLPVVSNDFCAQAYVNYKNQRI
DERVLCAGYKKGGKDACQGDSGGPLMQPIWNNQDYTTHFYQIGVVSFGRKCAEAGFPGVYT
RVTHFVPWLEQKILGA

>OfucSP5

MAELLFVLLVVCLQSSLAEPEKCEIRGVEGECVPLPACEQYVALLKNTSSTAEGLQLTAERRCG
LDVRNAKVCCLVVQPTLVDFKVNEEDQDLYVDSFPGHDTCGRTDGYRIFKIVEQLTEEESVYD
DVPLISNGAPVITRDEDRILSEISELTEEEIKYDLKYVISNGTPVILRDMARYMALLGYKVNANV
LWQCGGSVITEKHVLTAAHCISASLHVVRVGELDLAFEEKDAQPIDFYIKKKIPHELYSKFQNDI
AIVVLDGSINFKEEHHVGPICLPFDTKKKVLPEYNFYNYKGTFVAGWGEAPEKRERPTYLAYA
HVNIVSTDKCKSLYESSTRMIDDRVFCAGDTALGHDSCNGDSGGPLVAEYKVEEFNRTFYYQL
GVISYGYRCGEGPFVGVLTNVTHFMPWIRKKVLGENL
>OfucSP6
MAELFFVLLVVCLQSSLAEPEKCEIRGVEGECVPLPACEQYVALLKNTSSTAEGLQLTAERRCG
LDVRNVKVCCPVDSVVQTQLLSFKQIEEDQDLYVDSFPGHDVCGRIRFHQYIDKYRILSEVRVR
KRVRRSDIFSVYNGTPVILRDKSLYMALLGYKLNTNLQWQCGGSVITEKHVLTAAHCISSSLHV
VRVGEIDLAFEEEDAQPIDFYIKKKIPHELYSKFQNDIAIVVLDGSINFKEELHVGPICLPFDAKK
KMLPEYKFNNYKGTFVAGWGEAPEKPERPTQLAYAHVNIVSTDECKSLYENYSRTIDDRVFCA
GDSDFKHDSCNGDSGGPLVAEYVDEFGYKFYSQLGVISYGYRCGEGPFVGVLTNVTHFMPWIR
QKVLGENL
>OfucSP7
MAVFFVLLVVYLQSSIAEPEKCEIRGVEGECVPLPACEQYVALLKNTSSTAEGLQLTAERRCGLD
EKNAKVCCPVVQTNLLRFKQNEEDQDLYVNAFPGHDLCGRIDKDRKDKNVHILSESEIKSGW
MFVISNGIPVISRDMSRYMALLGYKVNANVLWQCGGSVITEKHVLTAAHCISTSLHVVRVGEL
DLALEEKDAQPIDFYIKKKIPHELYSKFQNDIAIVVLDGSIKFKEEHHVGPICLPFDNKMKVLPK
YNYYNYKGTFVAGWGEAPEKRERPTYLAYAHVNIFSTAKCKRLYESPTRTIDDRVFCAGDSEL
EHDSCNGDSGGPLVAEYKVEEFNRTFYYQLGVISYGYRCGEGPFVGVLTNVTHFMPWIRQKVL
GEKL
>OfucSP8
MAELLFVLLVVCLRSSLAEPEKCEIRGVEGECVPLPACEQYVALLKNTSSTEEGLQLTAERRCG
LDVRNVKVCCPVEPVVPPPLIDFKLNEEDQDLYVDSFPGHDVCGRTDKSRILSTIRKVTDKEQK
LGFMYVIANGIPVLLRDMSRYMALLGYKVNSDVLWQCGGSVITEKHVLTAAHCISKSLHVVR
VGELDLASEEKDAQPIDFYIKNKIPHELYSKFQNDIAIVVLDGSIKFKEEHHIGPICLPLDTKKKV
LPRFDFFTHKGTFVAGWGEAPEKRERPTYLAYAHVNIISTDQCKKLYESSTRKIDERVFCAGDH
HLRHDSCNGDSGGPLVAEYCPV
>OfucSP9
MNTYIFVGVLASAVFIVHGQSSLGTCTRPNGEEGMCVRYHDCPSMVAMLASPTRNIGELKKYG
CGFDKKTPKVCCDHNCYTPEGNPGKCEEINNCKHLSDMVRSTNRNDIAYARNSRCLTGKRTVC
CGPPPAEELSRASADCPDMTTAFPPHIDSSCCGVDANSASRIFGGNDTAIDEYPWMALLEYKRK
DNGLIKTYCGGALISNRYVVTAAHCIKGKGAWPPINVRLGEYDTSSERDCVDSDCADPVVTIGI
EDIITHPEYNADNSRHDIALIRLASPAPYTDFIRPICLPASDISSQDPADLEAYVAGWGKVNMTTK
STIKQDLKVPIIKTEDCQKSFTSRLSKPPTLWSYQLCAGGEAGKDTCNGDSGGPLMLSTGTRHE
LVGVVSFGLVNCGTEGLPGVYTSVYPYSAWIRRHLRP
>OfucSP10
MKLLIFASALLAVACYVNGQSCTTATGDSGSCVTILKCPSLLQVINKPNRTPADLDMLRKSACG
FEGNTPKVCCPCHTPYGEPGKCVGIYSCPHIANLLAPPVTAQSMLLVQASRCEGPDAYSVCCGP
PPESISKGACQSRLSAFPPDPRTECCGLDGGADNKITGGTATTVDQYPWLTLIEYLGKDNRIKLL
CGGALISSRYVLTAAHCLAGAVLNHGTPKNVRLGEYDSSHDGPDCVPVEGGGEDCSEGVVILPI
EKTIPHVEYNPVTRRHDIGLIRTQQAAPYTDFIRPICLPVVDITVKPPPNFKLWAAGWGAINTTH

SKSTIKLHVDLPFVSQRECQPAYSVARRQVALWGGQMCAGGEAGKDSCKGDSGGPLMFENGK
IYEVLGVVSFGPTPCGMEDIPGVYSKVHSYLDWIKGNIQP

>OfucSP11

MIYKIAFCLLLCVFSNGADEGEVCNKNGTPGVCINIKDCKSAREDLRNKKFPQICSYIGSDPVV
CCVDNAPPPPPPTTTTTTTTTTTSRPPVITTNRPPNNGPSTPYIPVYDYVNNDGSPSGCEPLSPKL
TSPKIGQKAFDKCVEYQQSLIYPCKKGVALIGDFTRAHQCHHNAEDLIIGGDPAKKDEFPHMVL
LGFGNDIATVQFQCGGSLISEQFVLTAGHCTVSREGGPVTYALMGALKRSDPINNDLLRRVKRY
IPHDDYKPPHRYNDIALVELESPVKLSQFLVPACVHAGDSPEDRVLATGWGLTEHRGSTSDVLQ
KVILNQFTTEECSVKFPVHRHMKQGFDPNTQTCYGDKMERKDTCQGDSGGPIQLKSKKLHCM
YVVTGVTSFGKQCGNKGEPGIYTRVAHYVDWIEGIVWPN

>OfucSP12

MYLSCSFVVLCILNFHKVLTEDPEMCKAIGGQNGTCVPLPSCPQYVQLLKDSNTTQLGLNIVK
EYKCGFEGRNVKICCPYKIGSNESDQHKSFKTDDDDFVDEFPLVCGTTDTIRISCKHELGTFLN
AKPAYKGISRWMALLGYRNGMQCGGSIITQRHVLTAGHCITNSLYVVRLGVINLKEITHFVKDP
QSRDYPIKRTILHEGYLKEFSNTLNDIGLVVLAVDVEFTENISPVCLPLPSYYRRDIEDVTHGTAT
GWGETESGKLSSQLLYSSLKLYDNEECRKIYEPYYRIDRRVLCAGEHETDACHGDSGGPLVWL
HCVGPNADDKDPTVSYEGYDTYFQIGIVSSGFACENSAFRGIYVNVTYYLPWIEEKVLGKTTIK
V

>OfucSP13

MMILKLLLQVALFLVIKDSKAQRNDGDSCVDTYTGTLGVCRPAEACKQAKNDYEFSGIRPTFC
EYNAFGQNLVCCRDGKTILQTAKPRTVDPPPVWTEANDNRRVSEKKCDAYSRSVLSRVDASPL
SVDNDGLTFVASKCHYTSVELIIGGEDATRGEFPHMAAIGWANFDGGYDFNCGGSLLSRRFVLT
AGHCSKDPRARDPAPAVVRLGDQNIDSRVNDGADPIDVPIRHIHTHPDYKSPVKYNDIALIELAT
DVVFEDAIRPACLWTKDGFRDYEKAIATGWGVKDVVTKETSKELQKVSLSLLQNEQCDPLLN
HNRNWKGFVPQQMCAGELRGGKDTCQGDSGSPIQAASKQNPCIFYILGVTSFGKQCGKTGQP
AIYTRVSSYVDWIESIVWPGE

>OfucSP14

MISWNVIVFLVFALVESSYQVQVGDRCVDNYTQTIGKCTPAESCDSARKAYHENGVRPTLCLY
SPFGTTLVCCKDGIPAFNVVSQFNNVPRPQEDSPRPQDDSPRPQYDSPRPQDDSPRPQDVSPNPP
VITPSVENRFSTIPTDTRRVSEKKCDEYSKAVVEKVEFLPLLPNPERLSITSAKCDYIGVKLIVGG
EKASQGEFPHMAAIGWTNFEGGYDFKCGGALISNKFVVTAGHCIRDPSARYPEPAVVRLGDQN
IDDSVADGADPITVPIKQITKHPNYKPPGKYNDIALLLITEVTFTSNIRPACLWNRPDFAPHKSAI
ATGWGVIDPDTRQTSNELQKVSLTLFENDDCDNLLANIRNRLWRGFMHTQMCAGELRGGKDT
CQGDSGSPLQVPSKENQCIFNIVGLTSFGAKCAKTGQPAVYTRVYSYLDWIESVVWPGE

>OfucSP15

MCCKQFLVISFAIILKNIAAQDVGDECVPNNEVFDGVCTVITECEVALRAIQKRNFHNYQRCGF
SGSNEVVCCPRTTEKFGATNDNRGGNNSQRKAERECKKIIETSRPPLDLHIIGGEKATLGEFPHM
VAIGYDRGNGYQFDCGGALISDTYVLTAAHCIINLERVEPQMIRAGVIVLGDNTWNENSDYRV
ARSITHPNYTHSSKYHDIALLRLANPVEVSENLHAACLYTSASDPLGPLTITGWGRISTTQSLTS
NVLLKTKVNPVTRERCTPEYPGWRKLPNGIIEGQLCAGDPQGGHDACQGDSGGPLQISESDVT
YRLVGVTSFGKGCGTTTPGVYTRVAHYIDWIENIVWPN

>OfucSP16

MARASLTAGFLVALLLPVNTLFSGDSCYEDEQGGSCIPLTQCKPLMSEIQNAGHPMPLHIRRKL
HEIGCGFELDEPLVCCPSHSNIDNDITPGFDIGNWGNINPKPTSAVVPTPSQPHTRDTDNKKPST

GTENRSGQPPNVQGHRNLHLLPENCGIVDSDRIFGGNTTRLFEIPWMVLLSYSSPRGTKLSCGG
TLISERYVLTAAHCVSNLGERLKLNGVILGEHDVRKDPDCERVGEKLYCAPNVRNVSVEAVITH
QYNPETLVNDLALLRLSEPADFSLESMKAVCLPITPELQNQYLDDQPGTVAGWGATEDGLQSP
VLLSVELPIVSNAACTKVYKDNPRISERQLCAGGVQDKDSCGGDSGGPLMYPGNVLNRGERY
VQRGIVSFGSKRCGIGGFPGVYTRVAHYMDWILDNIHE
>OfucSP17
MHWYLLVSGAAGCAALPPLRNEAQAPTLASAASAFWIAIGAFGASATSLSRSSSPGRLSLVTAG
EGCPPSAPPAFKLHSREKCNGGAGCIELKECTSLYDQLRNQGNTPTLARLLRSLHCGFNGDQP
MICCPPGLGPPALDVDTAERAGITRVKPINLLPGLDQCGAQNDDRIVGGTRAEIDEHPWMTLLR
YDKPKGWGFYCGGVLISARYVLTAAHCVKGEDLPTNWKLSQVRLGEWNTSSAVDCIGDDCSG
PPQDIPVEEIIAHEKYDPADSNQHNDIALLRLEHNAQFNDFVRPICLPTMSDLRQNTLEGYDME
VAGWGKTETRSESEVKLKVSVPVVSNSECDSVFSRVNRRIVSQQMCAGGMAGQDSCRGDSGG
ALMGKVNKYNNWFAFGVVSYGPSPCGTPGWPGVYTRVTAYVDWILSKIKP
>OfucSP18
MKLLIFASALFVTASYVDGQSCTTTNGDSGNCVTIHKCPSLLQILNKPNRSPADVDLLRKSACG
FEGNTPKVCCPCYTPYGELGKCISIYSCPHLAKLLVPPVTTESMSLVEASSAVAAPQPFAFATVPV
PHASNLLLLSRCQGPDDYSVCCGPPPDSISEGSWGTETRVDEYPWMTVIEYLKTDNKIALLCIG
SLISSRYVLTAAHCLTGSVLKIGTPKNIRLGDYDLSHDGPDCVTVDGFALDCTDDIVVLPIEKTIS
HAEYNTITKHNDIGLVRTKQAAPYTDFIQPICLPVVDVTEKPPPNFKLWVAGWGDFNNTHTKS
MVKLHVRLPFVSQEECQPAYSVARRKIDLWSGQICAGGEARKDSCKGSSGGPLMFKNERIYEM
IGLVSFGPAPCGMDDVPGVYSKVYAYLDWIKGNTEL
>OfucSP19
MKLLILACAFFAATCCVSGQSCTTAKGDIGNCVTFKKCPSLLQIINKSNRSPAEVDLLKKSACGF
EGRTPKVCCPCHTPYGEPGRCVGIYSCPHLAEMMTPPVTSYNKLFIQASKCQGPDAYSVCCGS
PPESIQKDGCDSRLSAFPPDPRTECCGLDGGADNKITGMLIQPSFLVSNSEGGSATNVDQYPWLT
LIEYARSDNSIALLCGGALISSRYVLTAAHCITGSILKNGTPKNVRLGEYDSSHTGPDCILVEGGG
EDCTEGIVVLPIEKSIPHPEYNPITKRNDIGLIRTQRAAPYTDFIRPICLPVVDITVTPPPNFKLWAA
GWGAINSTVKKSSIKLHVDLPFVSQRECQPSYVKPGLKADLWNGQLCAGGEAGKDSCRGDSG
GPLMFENEKLYDMLGVVSFGPSPCGKEDAPGVYTKVYAYVDWIRENIYP
>OfucSP20
MYLPCFFVALCILHFNKVLSQDPEVCKAMGEQNGTCVPLPMCPQYVLLLKDPETIQLGLKITEE
FKCGFEGNSVKICCPFDGSNESDHLVSFKADDDDFVEEFPSNCGSTDTKRLFCKYETSFLLASK
PAYTAISRWMALLGYKDGLRCGGSLITQRHVLTAGHCITDSLYVVRFGVINLVEKTRFVKHPKS
RDYPIKRTILHEEFLKSYSNTANDIGLVVLAEDVQFTENISPVCLPLPTYYRRNIEGVTHGTVTG
WGETESGSLSSKLLYSSMKLYDNEECRKIYKHYYTIDERVLCAGEHGTDACHGDSGGPLVWL
QCVGPKGDDMPYDGIDLYFQIGIVSSGFACEYSAFRGVYVNVSYYVPWIEEKVLGRTTIKI
>OfucSP21
MGPPLSTWPYSHPHSPNRVCDSDNQKSYLVTLVDNFRVFCLNFTFALLVVQVLFSWNSVTAGP
VILNLSYAQGPVPLARNIRHLPCISRRTGQEGLCMFAIDCLKANGSHLGTCIDRFYFGSCCQIPD
KTILPQIIGNNIEDNSIDSANFVHPQTEDKVQSTVSKIPDIFPTRKTTTEKATEATVTEKSETVQDR
TEPVDLETKTDDVTTIDDKNDGKLTTMVESTTKAQATKPLATEVPIKLSTFQTVSGDNSVTEVP
KTETTEEEKQTTPTTTTTKKPKPLVTRKPVKPTYKPRPTYRPSNFTRPQFSPSTLKPRPTKPPLIF
NITRKPPPIKQPPKRNTTKKPLPSPPRLNITIIPQSTSSRPIFTRPLTTSITYINSTLAAEEKPTTTTTI
STTTSTTTPSTTTTEKLTTTTEKATTTTEKIVTTLPTERVTETETVTETSIVSSTEEMTERMTERTT

TMEPSSVQPSSTESSSEIPSSESSSVIPSSESSSEIPSSSESSPIEEPVQETSLTTPTEEIKETTSPITEKE
TIRPATEADIPVSSSSAAPTVVSTEFPPFVTWSTVDGGTKAPEGTKAPENLTTPVDDTWSPITPPD
GWVLISTVPPSSTSESTTSTTTSTTTTTEQPTTVEATQASQETTVQPETTEKQEPVTTPSTTTTMT
SSTTTMTSSTSTESTSAPSTTEAPILEFTVNVTLSPTVPTSTVSVTTMTPVTENVTSVTNATEADT
TTAVPVTTTLSSLNMSNYKEVCGRRLWPQGRIVGGQKSAFGQWPWQISLRQYRTSTYLHKCG
AALLNDNWAITAAHCVEHVPPSELLVRLGEHDLANDNEPFGFAERRVQIIASHPHFDPATFEYD
LALLRFYEPVTFQPNILPVCVPDNDDDYVGKTAYVTGWGRLYDDGPLPSVLQEVRVPVINNSL
CEAMYQDAGYNEHIPNIFICAGRRKGGADSCEGDSGGPMVVQRDRDNRFVLGGVISWGIGCA
EPNQPGVYTRISEFRDWINQILQF

>OfucSP22

MSAVRNEPKTKWTTENHRATDHKYKKVLRKRKKCVTDTDTTKCRAKIRWRISSRVLLPLMIL
NFTGYASAVESITSRVLASILGYPSTCNSGSEVRPCTLSLECWLRGGVRARGCGGWLFTCCMPP
TPPVADSFENSIPSSEWKYSKVVPPKLRQIPQRSVMPSNMFRRRADDDASQVDCGLPSSRILQK
RIIGGREARFAEFPWQAHVRISEFQCGGVLVSRWYVATAAHCVSRARPRDIAVWLGALDTTAG
ADTSRKLGVVQKILHPLFQFRMTQPDRYDIALLKLARPITYMSHILPICLPEGDIELRGRAGVIA
GWGKTDASNSHTGTNLLRAATVPILSKEQCITWHQSKQISVEIHSEMICAGHSDGHQDACLGD
SGGPLIVMDNGRYYLVGITSAGFGCGVDHQPGIYHNVKVTAGWIKGVISPSSNYVVDY

>OfucSP23

MFVSADDSAVVRDGSIGSTISMKCCVFICVLLFSWGTQAANKESCETCEPMDNCPFFKNLSEA
DQANWRERYDCEDASSNTKPIYGFSSSAKGDLVCCPENVWQVENPSPQPSSTERPKQISSTSTT
EQPVKMEKNSFLNQPNFNSPIYPGYPNGFTGNQFNPGFQGYPNTGGQYPNTGGQYPNTGAQY
PNTGAQYPNSGAQYPNSGAQYPNTGGQYPNTGGQYTNTPLPQFGNQRLTGNQFDNPGPNGN
GYQVYENDNEHGGLNAGNLGGQQGFGNGNSAGNQLGNQNGNPYDNGFEMRRGKGPDNNA
NAQNPYFLPANRKDNSLTGTNLGGQCPVTSFPPDPDSGCCGREATDPDSDAASANGYPQSNWA
PHRNQPSRGSRPLWPQSDRVKREDVNITLDNRIAGGKETELEQFPWTVLLKTIFVYPDKEVAFS
CGGSLISSRYVLTAGHCIVDSKGTVKDVEIYLAEYDKRTFPKDCKNVLGKGQVCVENIVMHAE
NLHLHPEYDDNRLHNDIALIRLRGNAPYTDYIRPICLPPINVDSPELSNLRLAVAGWGRNGQYK
SNIKQSTVVNLVPQSDCRSAYPGLTRMQVCAAGYTGEDTCKGDSGGPLMMMYAGRYFVSGV
VSGKRADAPCGTSVPSLYTNVYQYLPWIRSMMQK

>OfucSP24

MKWTLLIGLLAFVSHLHANEECPAGETCMSIKECPQDITDLARMRPKPKDILERLKKARCGKP
ADKKLCCKTPPECYTWDGRPGVCELYDKERTKKLNMTPWKIKMSKCINKNRLDICYSNEPER
QHANCESAFPASLDSGCCGIEKSAGDRIMGGTATKIDQFPWLALLKYPKPSMSCSGALISSKYV
LTAAHCVDNADKDQATDVVLGEYDKSTNYDCDDELCIENATVTAKILQIIIHPDFDQIHKNYDI
ALIRLEKAVPFTDFIQPICLPSHVADLTKNPPEKFKLVVAGWGDVDPNHHHSSNVKLHVQLPFV
DALACRRTVRELWTSQICAGGEAKKDSCNGDSGGPLMYEREDVVFEVLGITSYSEEKCGTAN
VPAIYTKVHSYMNWIHSKIRP

>OfucSP25

MWKSALNLLLLVYSCKAATNNDCITPLDTPGKCIMVSDCSYFTEALAGGQVEKRTLDFMKQS
KCGCDVTVPRVCCGPLPTEEQFESNKERLLQDIDPDSLEDSKLASEDKCGLDFSVFTNKIYGGQ
RTKTGELPWLALLWYSVQLEATNQLMYLCGGSIINQRYVLTAAHCIARRKYEVLDFVRLGEND
ISKNIECTGNVCADPPQDIKVLSVHPHAEYNRTKHVHDIGLIRLVKRIVYSEFVQPLCLDRMGQ
WNVDDQIVAAGWGQTQKSTLSLIKLKVKLPVVDRERCAKTFPSLNDGQVCLGGKKLSDTCQG
DSGGPAMRQRSDRRWQAIAVVSYGIGCGVEGRPAVYTAVHKYHDWIIDTLQATNLCPKAESRF

SDEPGE
>OfucSP26
MSGKSCTTPLGQPSQCISLYECQDLLSAFERRPLPSFVVTFLRKSQCGFLNRTTPAVCCGPLPEE
DSQVPVIPVPTTPAPNRQDDGFNADDANPSPVGECGVDTNGDRIYGGQFTELDEFPWMALLG
YRTKSGRITYQCGGVLINHRYVLTAAHCLIGAVEREIGKLVTVRLGEYDTQSEVDCSDGVCADP
PQEIPVAEKYPNRGYNDQNTNKKDDIGLVRLSQRARYTFTTPAYYFTECKCLFQNSTQYDLKLI
KQGKTIFVVNCLAISTKHGHRTVRYGNPWGRPLSSSGHPFQPTSKKEEVLNSTHYVKPICLPGA
STRLSPGYEVYVAGWGKTLQGTNSPVKLKLQLPVFEKNECVNKYKNLGANLIDKQICAGGNF
AKDACRGDSGGPLMVRTPQGHWESVGIVSFGYGCGRDGWPGVYTSVAAYKDWILSTMRSTN
S
>OfucSP27
MEHYVDGIQNFKIRRRTSGKMNLISVFVIFAAFSNCVLSSDVENDVLTLQDDLDLKDEAASDRR
RPSNEQEKDILLLDALGRRKGSYHHNAYASSQEDDSIMDLLGKMIPQTCRYRGARYPCGLSISC
VLGGGKPMDLCSGGMIWACCVDRDTTERPSEVAPVVHNASDLIELIGTFPHENYDYQNTNQFE
DPIIIYTDTNLPNPAHNRPKPDLNRPKPDLNQPEPDPPKPDWEYQNHYFHVKPSYHETTTQNTH
YNDGDDDYVPVHTNGHKPQRPSFPGCGEHFTRSNRIVGGHSTGFGSHPWQAALIKSGFLSKKL
ACGGALISDRWVITAAHCVATTPNSQLRVRLGEWDVRDAGERYSHEEFAVQRKEVHPAYEPAD
FRNDVALVQLDRGVVFKQHILPVCLPQKQMKLAGKMATVAGWGRTRHGQSTVPSVLQEVDV
EVIPNERCQRWFRAAGRRETIHDVFLCAGYKEGGRDSCQGDSGGPLTMKLEGRSTLIGLVSWG
IGCGREHLPGVYTNIQKFVPWIDKLVNPQ
>OfucSP28
FPDSRKLFGGYRITPSHCKASRAAKYNRGNKICMFNHECVQRGGEVVGTCMDGFLFGACCQL
KSDSQSHIPKGPGVVMTSYLDYPDAETETDDYDSDQLSALHNSFRPVVTPGYRPGFSSQVTITP
DVKTTEAMQQEMISEGFNQITNSLLHSPPKDNSIFGKPVKPEDIYSHSSIDHSVAETILLNENGSV
AENVVRPSDFNVQISSMQTKPTVSPISSTPTSTPKTVSTHHPVYTKPGFKPKPGNKNTSKNPSTE
NYVMVSTVTKESQKITELSSIDSIIQMLNDSTPSMKEEATSPASVDLDIIENKSSPGPTGTVAPISY
SSGYPSYYSSTGHYVTINPTPNPSTSYSKPSFTTKRPYTTPNSIPQGDKPTSKPYYSSPSPSQSTSQ
AIEAFNRYPTDPNNFNSLTTFSYVSSSTTVAPTTTSRRPPSTSYVTGAKPLRRPVTPPTKIVPGYD
VAPDTFSSVTPTVIVLNGFQTSKPETSTEEKEPQFVEISQEPFKKPVSQITVNNHIQSTNNIYMGK
PPQTFERPASPTVVITPKPPPVTSPYPLKASTKPPTVSTSPFPVFDSYTEYYAPTTFKPELQTSPDD
LINFPPVRNPMLNATGSNPALYNTSIALSDNSEELLHDVEFTTPTWQEDENLNEKMNLFVNKIV
GSLQGTFQELHDIVILDKKPNATRFTTPKPTKKTTTSKRPFLTSKKPVRVTTTTTRRPTVKTSKK
PTRPTTVAYRRTTTTTPPPTTTTTKKPVTTTSKRPKPSKRPITTTAPTTVTTVTTELSDEVTTESV
VYEQVDYNDKNLCGVRPLVKTGRIVGGKNAKFGEWPWQVLVRESTWLGLFTKNKCGGVLIT
SRFVTTAAHCQPGFLASLVAVFGENDIASDKEARRPVSRNVRRVIVHRQYDAATFENDLALLEL
ESPIKFDAHIVPICMPPDESDFTGRMATVTGWGRLKYGGGVPAVLQEVQVPVIENSACQEMFQ
TAGHAKKILNSFICAGYANGQKDSCEGDSGGPLVLQREDGRWQLVGTVSHGIKCAAPFLPGVY
MRTTFYKPWLKSITGVH
>OfucSP29
MTWWWALALLALATPARPAPRAGNGELIYSYQMSRKSCEAGGSRGSCMWVQECNRVGGKH
AGVCIDRFIIGSCCLLPEKPISIEETQSPVTMTEKPYTPPPSGHSTTHQQVEASTQTIARPNWQTQ
RPSFMTKPIDGAPTSYSYRPPEINLPSLDNLESSSEKNSDIVNKITYNSVNKYQNVNRPSEAETSP
HNKISSSLSILSGARPMAVSEQHAENSISSAQIMSRPSNLNTIHWQATTEPSFITKPRPSNWEKPA
GKPKPTKKFTTTTSKPHKNYQKPKDPYVKTDESTQAPIQTTAAATNNACGVTSMWPRPETRIM

GGKDSSFGKWPWQVSVRRNSYFGFSSTHRCGGAIINEGWIATAGHCVDDLLTSQIRIRVGEYDF
SSVSEQYPFVERGVARKAVHPKYNFFTYEYDLALVKLEAPVQFAPHISPICLPATDDLLVGENAT
VTGWGRLSEGGVLPSILQEVQVPIVSNERCKSMFLRAGRHEFIPDIFLCAGHERGGHDSCQGDS
GGPLQVKGKDQRYFLAGIISWGIGCGEANLPGVCTRISKFVPWILQTVNS

>OfucSPH1

MCLWLLTACLGILPANPSSPNIVIVQGVPAPIIVPTTATTAAATAATNATPTAAPTIQPTGQPTVQP
TGQPTGQPTGQPTGQPTAQPTGQPTGQPTGSPPTGSPPSGSPPTGSPGPLPIDPRLGTTPGSPAAL
QLPPITGYSAQVPLNDMAVRFPREKRSAEMNIKDFLDSISGSQRVHTIVKRQSCTCVPAGTCVR
TATNAGAGLIDVRIVTPGANTGQCPAGQEYCCTGTTSQTQIACGTLQTVPAVSVTPGSGQANYG
EFPWQALIMTKQNDYLAGGVLIDQLNVLTVTHRLVNYVVSGTAPNVKVRLGEWDAGATYEPI
PFQEYNVAKVFTHPSYVQNTLQYDITVLRLSTPVPLTPATGSVTTINRACLPPSSTSSYVGQRCM
VAGWGKNMFGLQGMYQQILKKVDVPIVASATCQTQLQTARLGANFVLDTTSFICAGGEANKD
ACTGDGGSGLVCQVNGQWVVVGLVAWGLGCANANVPGVYVNVAGLLPWIQQQVATA

>OfucSPH2

MMLKICALVVALTLGLADQTGAINPHHHSLAPLIDSVFFANDVIVARNQQGEREGGKQGDKTP
CEMPDGSSGYCVHSYLCESQQIVKDGAGLITERRSQCVAPKICCKHRKTNQETPHIGSRSNGDK
TPCELPDGSRGYCVNSYLCESQEIIKDGAGLITERRSQCVAPKVCCKHRKTNEGTPDISSKSNVR
KCKFSNGREGYCVNPNSCRNRKIMGGYSRTITSSDCLETEVCCEKDHENIESTTKSRTLVPSIPK
NLPNGAQCEAYDGSIGRCTLPHTCNNDSTILDGSGLIVERKVDPCGDWGNAIQICCPAKYLAG
KIPDIEATTVLTKSRSMDDEEEKDSSCLTGADNAGKCVPFHHCEDYDGADLLDKRTMRYCPAL
TTCCELDRINMESIPTNVKKEGCGVANPDGLWPAFKTSFLEAKFGEFPWVAAVMTKTSSFPVNR
NITVGSLIHPSMVLTVAHYVDRAVDGDVIVRVGEWNTLDVNEPLPHEEVAVKKIVIHPDFMRK
NYRNDIALLILEHPVKLAPHVSLVCLPEPGAVPAPDTDCVSNGWGKDAFGKKGAISSYLKKVP
LPWVEHGDCQNRLRTTRLDSFFELHEGFACAGGDKLDTCSLDGGGPLACPSESDPGRYILYGV
VSWGIDCHMPGIPGVYMNVAHYRTWIDQVMETEGV

>OfucSPH3

MNLNMNMLLVLAIYVCAAYGQSNPEDDIFDDSGPTVYTTVPPSPAAPSDCTLADKQQGSCVAP
SSCKTPNHGENRVHFRQNNCPENTMCCVLSDIISVTSRHQTPTPPIEATTPTVAPNIGGTQAPTK
GCGVRNRNGGGGFKIKDKIQRAMFGEFPWTVAVYEVKPDGSLSYTCVGSLIHPRVVLTVANRF
LEEDVTWKVRAGVLDLENKRRARYLDQLRDVVSAVTHDSFDSGIGIYDVGLLFLDSDILQDVH
IDYTCLPEPSVVTAPATKCVVSGWGSAEYMGPMENVQKEMNLPVVDRQTCQTSLRAALNNPQ
YDVDPTLICAGGNGKDTCTGDGGAPLVCPYPDGRFYQAGITSWGYLCYQKDVPGVYVDVAS
VRPWIDEQVKAKGYDTSYYTH

>OfucSPH4

MWTLTILLVACSSEIIWAQSSPPCILEGGGLGGCVETEQCDPNTNTIIEDGSNIINARNDGDVIKC
PQPQVCCAYMRSEMRSAATEEDLCTNSSTCCGVQRRVDIPGYVKQRTNEADPGEFPWVVDLM
MRSIDKVYQYAGTGSLIHTRVVLTAAHIVYGKDTNLLLVITGDHDQEKAVMKKKGKVVTEVVI
HPQFNRGNGDYNYALAFLRKSALADGLPHVGTVCLPPAGYVPTAGTRCIFSGWGVDSNLINQQ
QLVKTDVPMMAHGACEQRLQQDPSLGPGFRLLDSLTCAGGEVGRDICGGGGGSPLACPMEDY
PERYVQIGIVTWVTSGCGINGSPTVFADVSKVRSWIDQQLANRSFYIN

>OfucSPH5

MTCARVFVLLCLCVDWTVNAQFGSMTLNVNFDGSSNENSENIKGPCPPYTSCMAISSCPMLED
LFDFSCFSSDRYFHRLNELTCGHANSEDYVCCPSCEGDKIYKEGAEKCGQSMVRGINYNGLGA
HPWVARIGFKHKDTGNVKFACSGSIISKKVVLTAAHCALAEFEDYNQVSTVVVGEWDYAKSID

CNEYFCAPPTQAIKVASVVRHPGYEQKVFRHDIALIVLKKDIKFSVTAAPVCLTSNPEVVLNER
ALLVGWGTLYGQTNVVSRQQLLEVPLVSLEVCDRIFGKSVTIHEGQLCAGGEEGKDACSSFGG
SPLMVMRNGSYVQVGIVSFGSENCGSEGVPSVYINVAHYYKWIVDNSPS
>OfucSPH6
MWREQCILLITYFGVIWAQSSPACALKNGDLGVCISYERCDAATNTIIGDQSNIIKPGYEQNLIQ
CPQPKVCCSFTNNWTTTTTTTTTGTDSVEKCGVQKRAKVSNFHKTMTDEAEPGEFPWAVNLM
IVNNILATAAKYKYVGTGSLIHERVVLTAAHVLFGKNPDNLIAVIGEHDQKKKVMEKRSKTVQ
YCTIHEQYDGTNGDYNFALVFLAESALKDNLPSAGTVCLPPADYAPPAGTRCVFAGWGVDYH
LENQNQLVKVDVPMMGHAECERRFQQAPSLGPGFRFLPSLTCAGGEGTRNLCGGAGGSPLVC
PMEGQPGRFFQIGIVTWTTDCGKDGAPMAFANVAAVRPWLDEQLDNFVKELSIGMN
>OfucSPH7
MKPLQAVVAICLVLAIGARSETLENTKESTKDVISESEQSEPQARAERCTSCSGGVKLSFKSPND
VLAAIQALPGGEVHTQQSFEGCSSDKGCAGLKVKDGRVVEKFGNVDAFQAAAAADTGNEFQ
FHAGGGLGNVFEGGIPDGGPFWWMNQNSPFKNGAAGGNFEKFSKSSSSFTSSGTAGAGGVDI
AANPFLNGDFSKLAGGFAAGAESKPGFSSSSFESSSFSSSSKGDVDISKNPFLNGGVKFGQNGFA
AQGGFGAGSAQFGAGAAGAGQSGAGQSASQFGASQSAFSASNNAFGAQGSQGAGAGNSGLT
SGSAQGGAFGGSFSGSAFNSASNKFGASGYTGSSPSPFTASTGSNVNLIQSSQKGSEFDFEQQQ
QTQQNIDEAFQSTGNVHAHEHSGGDLQQTCAGQGYVCVHKAQCNNGVVNTNGAGVVQART
QKQYCNTRTEICCRIEVSSVGAGSVQGSSSFGAQGQSAFGATNNRGTSKFGVSGSVGTGLAPPA
NKINNVYKSTSQSNFVETDSFSAGSEVAGVFRPGAGPGLKPGIPYLPPVDTVTQPSFISSTSVPFT
TTPKPFVPVRPPVTPPRPYLPPVTVTTSTSAPGYLPPDVVPGESKDPPPYYVTGSLLKEPFLTTPS
PAIPDTPSGCAAALKCTPIEFCTAEGVISTTPVVLTRDQEAYRVPLTDCKDVGTGTIGKCCRDPLY
TDPWPTNQLGKWVPGVFGGNDGKYVPDSNPQSNNPRGQVTGRPPVTGSVIRNEYKPSTPGPY
GPNQVTPGFPSSTVSSFKGQGSVTSFGQGQYSQGGVGQYSKGGQGQYTVGGQGQYTVGGQG
QVSQGGKTQFVQREQTQFGQGGQGQVGIGGQGQVGIGGQGQFGIGQQGQIGIQQGGGQTQTA
QGTQFSTNTQSFGQKGQGQVVRQGQGSYVEQGQGSFGVGGRGQYTVGGQGQLEIGGGGAQ
GQFTQVQGETQKTFGQGSGFGIGIGQGQTGFGISTGFGQGVIQGQGQGVRQGFGSAIQQGFGQ
AIVQGEGETSASEGVYRVFLGRYSGGNGQCGLLNGQKPYGNRNDLEVDFAEIPWQAMVLLQT
NRSLLCGGVITRPDVVVTSASCVDGLDAKNVLIKGGEWKLGVDDEPLPFQIVQVKTIVKHPYY
KIGSLKNDAAILVLSENLRLAKNIQPICLPESGETLDAFYNGAGECIVTGWGKQVLQAHLAGSI
MHSLNVSLLNPGECQAKLSSDYPHLLEQYDQDSCACGQPLNPVNNICKVDIGSALACTTDNSH
YVLRGLYSWDSGCQTGNQIAAFYKFDLEWYEWAIGLIESTRFAKFAIGFKVTQNRFSSQIKGSS
SQFGGNSQYNSGVKGVTSSGIKGGVKGAGFQEQTGGLIAGAFQGSSATASATTGSSGTGFGSGI
SGSGFGNGAGFGSGAGFEGSAIKGGFGTGQFGFSQFGSXETCVQIFGLLSQRHLVCL
>OfucSPH8
MRAFILSVLVTGALAQITVDPKDLEEIFGRPDDPPTSSTTTTTTTTTVKPGPGLETFTVKPTDTP
TTMVDKDGNACKCVPYYLCDRDRNGINIKNASVTGWGELDIRFGEDKCQVTVELCCTEPKDE
ENVVIPDVDPSELKGCGYRNKKGLDFTISGGSGNEALFGEFPWVIALIDINGSYAGVGVLIHPQ
VVMTTAHVAYKYTPGNLKIRAGEWDTQTADERLKHQERVVSEIYIHKDFRKNNLFNDVALLRL
ESPVTLGQHINTICLPAQDEDFGQYRDCAANGWGKDVFGTQGLYAVILKKLEIPMVPYSRCQE
LLRRTRLGNNFKLHRSFVCAGGEEGRDLCTGDGGAPLACPIGGDRYKLTGLSAWGIGCGTKDV
PAVYASAPAFRRWVDEKMQEWGYDTQVYTI
>OfucSPH9
LPSGLLDTLDTQVDAKNCPGVCMHALASLICSNVLDEVECPKPSMKCCVDEPLGNDTLITTRR

PFTTRYTTTEAEEEDYDEPVTSPSVPQDKDSGNISCPGLCVDTSLTRYCISYLTSKSLCVPGRLCC
VSKEGYGDKPPADLVIPGEYHKNNKPTKRPTTTTTPPPRQKPGRKCNGDCVGGLFALLCEEVD
EDAVCPDEGTCCNNEPKAETTTRRPTTTPRPTQPPLRRCPGRCMLPLMMAFCEAPASLIPNTEC
KASGRICCDSVSVKRTKKPPTTTTTTTPAPPADARADCPGSCIVPYLSFTCFRNAEMTDVFKCK
KSGTQCCAPKLKIQEALGGRDRNDSFPLATTTPAYAPHTTPAPPYTTAISQYDQNYDSTLKIPEK
YNKYVCGVKGTSSRSGRVMGGADGERGEWCWQVALINSLNQYLCGAALIGTHWVLTAAHC
VTNIVRSGEAIFVRVGDHDLTRKYGSPGAQTLRVATTYIHHNHNSQTLDNDIALLKLLGKAELK
EGVCLVCLPARGVSHAAGKRCTVTGYGYMGETGPIPLRVREAELPIVSDAECVRKVNAVTEKI
FILPTSSFCAGGEEGNDACQGDGGGPLVCQDDGFYELVGLVSWGFGCGRRDVPGVYVKVSSFI
GWINQIISVNNL
>OfuSP1
MRVLLVSLFLVLTIAASHAVGPYKYHRRIGIPLAAKIRRTEEDAAKAGVTDLRIVGGSNVDISQV
PYQVGVINRIAFFLTSVCGGSLISNNRVITAAHCHDDGDIEALSHTVVLGSNTIFSGGVRQSTTNI
AMHHDWNPMTAFSDIAVIRIYDVTFTNVIQPIHLPSGMQLGNTFEGLIGTASGFGRTADGANIPN
NQVISWVRLPIISNNACAAVYGPFVHASTICTSGAGGMGTCQGDSGGPLVVEIMGEKLLVGVTS
FGAEAGCSAGFPAAYARITSFMNWIWAL
>OfuSP2
MKVLLGSVVLVLAIAASYAEGPFNYHQRIGIPEAAKIRRTEEDAAKAGVDLRIVGGSNVDISQV
PYQVGLVIQILWILTSVCGGSLISNTRVITAAHCHHDGTVTAQSHTVVLGSNTIFSGGVRQTTSDI
VMHPQWTPETVANDIAVIRINAVTFTNVIQPISLPSGSQLNNNFVGQVGIASGFGRTSDGANIPN
NQLVSWVRVPIITNQACASVFGPFILSSTICTNGSGGMGTCQGDSGGPLAVEVGNSRVLVGVTS
FGAAAGCQAGLPAAYARVTSFISWILAI
>OfuSP3
MKFLVVLLAVASLAHGKVVPDNHTAFGYLKKSIVEAEKIRVREEQYLQQQRIVGGQPANLGQV
PFQAGMLINIIGFEGRAVCGAVLISADRLVSAAHCWSDGQHQAWRVEVVLGSVTLFTGGNRQF
TSVFINHPSWFPLLVRNDIGVIYLPTSVSFSNTIAPVSLPQGAELQEDFAGASAIASGFGLTVDGG
SISSNQLLSQVRLNVLSNSVCRLGFPLILQDSNICTSGIGGVGTCSGDSGGPLYITRGNRNVLIGV
TSFGIALGCQVNFPAAYARVTSFMPFINQHLILLWKLVAFSQLGDMKYLVVLLSAITAIYAMDLA
RLAPEGASVYGYLTNIGIPEAERLQKAEKQYLAAESGARIINGQPSQLGFLPYQAGLVVSIVGV
VGVSLCGGALVSNNRVLTAAHCWFDGTNQASMFTVVLGSVTVFSGGTRIDTSSVVSHPEFSVN
PARNDIAVIYLPQRVSTTSIIAPIALPSGTELYESFSGNSAIASGFGMTSQSGSLTLDQFLNYVTLP
VISNMECLTTYPSTLQTTNICTDGEGPRGTCHGDSGGPLALFRNNRWILIGLSSFGSREGCEAGL
PTVFTRVTSYMTFIYQNL
>OfuSP4
MKVLLGSLVLVLAVAASYAEGPYNYHQRIGISEAAKIKSNEEDAAKAGVTGRIVGGSNVDISQV
PYQVGLVIQVLWILQSVCGGSLISNNRVITAAHCHHDGVITAQSHTVVLGSNTIFSGGVRQATTN
IVMHPQWNPQTAANDIAVLRINNVAFTNVIQPIALPSGSQLSNSFENQIGIASGFGRTVDGANIPN
NQVLSWVRLPIISNDDCAEIYGPFVHASNICTSGAGGMGTCQGDSGGPLAVEVSGSRLLVGVTS
YGAEAGCAAGFPAAYARVTSFTSWIWSIARIYSDLLKVLLGSLVLVLAVAASYAVGPYNYHQKT
GIALAAKIKSTEEDAAKAGVTGRIVGGSNVDISQVPFQVGLVIQVLWILTSVCGGSLITNNRVIT
AAHCHHDGVITAQSHTVVLGSNTIFSGGVRQTTTDIVMHHDWNPQTASSDIAVLRINSVTFTNV
IQPIHLPSGIQLGNTFDGIGTASGYGRTADGAGIPNNQVLSWVRLQIIHNNACAAVYGPFVHPGT
ICTSGAGGMGTCQGDSGGPLTVEIMGVRLLVGVTSFGAEAGCSAGFPAAYARITSYFSWIWSI
>OfuSP5

MSVALLLLLIFLDFANSEYDTRVITTLKYSKNYVHPTVINGIPAQTGQVPFLVSLKEILVKVSDLK
YIWTNICGGSIIRNTKVLTAAHCFEGKSFLYYHHPEYLRVVAGNFTTELIHTGNTDTTRRAQWR
KIKKVVLHRKFFFPENDLALVFVNEPFEYNINVGHVVIAKRRADYPNQCISAGFGSMNHSEDRI
TPILLVANMFLLSRRNCSKMWEMNMDKFICTYSFMSDMGRGDSGGPLACRGTVDPAEKKGLP
LLVGVVSGKNFDKTTLFTRVSAYKTWIANAKSAACAIGDYVSYCDIKYLVFVVVLYSFSCDLQ
STDVCG
>OfuSP6
MNVLLLLLIFLIHTTKCNMENRVITSLKYSIYRVKPVVVNGEPATNDRVPYLVSIKQPSGRIGTDIF
WTNMCGGSIVGEQKVLTAAHCFEGNSFYYVYNAHQLRVVAGSQNSNLQHSGATDTTPDLQVR
KITKAILNRGFHFPTHDIAIVFVDVPWNFTDTVNFIIPAKKTTDYPYTCISAGYGRIGHDTWSLES
DVLLIASIRVLSRWRCSTLWQMNMNTFVCSDSAITDVARGDSGGPLACYDTMDPEEVPGKDLL
VGIVSGKNFDKTTLFTRVSEYRDWIDSNFAYRLSSTFSILFTIISMGLSLNINMPSIS
>OfuSP7
MIPVLLILSVLGGVIEAALRIFGGRDADPMEYPYVVRLGIKYSLNQLDAYFEHFQFLCTASVLTP
VWTLTAGHCVDDAVLTFLQSSYPTASLKYVIMYGSATAHRSTTDNFSDIVATVKHPSYNSMALQ
LGASGSNDVALAKTTSTKLTKYAKLSAVEYPSLVGQAAIAVGYGITFEGDQVDHTLQLGKTLQ
VLDVMIGTCAKDFRKNSLSPIICLSRLCSQPATILCRGDSGGPLLHDSGVVGINHMVESDECTLR
SANSGLVKSSTVGINVPISPYVGWISNQIREKEESH
>OfuSP8
MCRAYNELARAGKDIDVFNSKFPQYKKAISDLLKNDETTASPGGNDQSHLTGYILNGVDVPIS
MYPYMALIGRFGRVHCGGSVVSDRVILTAAHCLCSSTGHILPAKTFDVYLGSAKALDGTKYKV
CKTLPHARYGTVLYGYDLGLMLLNKNIKFGATVQKIAPVSNSQWKKKASMLTVIGFGVTSNA
GKLPETLQMTKMYYINETQCFNSQHGMPFPIVLPSHMFCMIGERWTSDCFGDSGSPVIWKRNI
VGLVSSGRDNFCGSDMMPSMYTDVRHFNKWLDDNVRDLEENTECNIEQKQDPQPKDIRTYAN
DRYGEKLAARYTKQTIELVLNFD
>OfuSP9
MRIWLCLQSVIIAVYSQEKGIDIKGNANVTAEGKIVGGKPVTRQQYPSACLFFNVGAQCAGTII
NTWTILTAAHCFDDNKDKDQMVIELESRYLYDFTAREYDVSSFVIHENYNKSAKFACDIALIFL
KKRIKFGSKAKKGVLVNHKRWMNVKEEKFVATGWGWTSYGGQLSDQGLMMTELQFVDSKT
CSRMHNVTITPDMFCLYGKGIRDTCKGDSGGGILWNNMVVGITSHGDGCARVNKPSVYANVF
YFRKWISDQIENFMIRYCNRTTKKP
>OfuSP10
MYRPSIIMALLVFSTEFDLLLCHLKDLGEKNDTMSVLFQEFEDRVLGGESTTLHEYPFNVQFFN
YGGMCGGSILTRKTVLTAAHCFDHNKNVAEMTVISNSLYIRHARTSRRHPVWDFVIHEKYDDPI
KYANDMAMIIIHDVFNLTETVQMAVLTNSIAWMNETKAKFIATGWGIVEMRRRALQKTTLRYV
PNKECSDMNALQLTEGMFCLYGDQQRDTCRGDSGGGIVWNK
>OfuSP11
MAITRFWCIFELISQQYVVHGILGGHRIRIEDAPFQVNYGDICGGVMIHAVWAMTSAHCGTEEN
YIRVGSRHRFNGLKIKILSHQSHPNFKQNHEYDYDVQLLRLYGNVNFNRKVKAIDIGSEHGRHI
LVAGWGYPREKSDYTDVLHQVKLYRVQMSRCQKVDKKWYNHTLTARMFCAGGMGRDACQ
GDSGGAAVSRGRVVGISSFGYGCGRYNIPGVYINISEENIREWIRGYTGV
>OfuSP12
MFFQFVLVVLVVAYNVDVAQTESYDLIIIPVPKASKHPTRSKKIIEVKLPTLIRYSQSQHRKGQN
KLDIEQYEEPTELPDIDNFQYKERDGYRGSDSTFHGYDMYSNYGEDKIVGGFEVDINLYPYHV

AYGTNCGGAIIDKKWVITAGHCGKKPYIRVGSKYLNQGRKVDIKNYHVHPTWSASNKEHPFD
YDFQLLELKEPLKFDENIQPVKIAHIEDMVIGKVVTVTGWGNTEENGPYSNVLRAVRVPIISKE
HCQNVPFPYFRGGLTARMFCAGFSEGKKDACQGDSGGPAVSYDRILGMVSFGYGCATPGSYG
VYSKIAKVRSWIEQTTGIKFD
>OfuSP13
MARRTIILLSIFSMTNNVHSQFYDLFSSYQPYNLQTARPNIYPQEFLENRPLFEGFIRNDSDEERR
PPSITNDRREPSRSTTKTRRKTTTTAQIFNLNFFTTPKPVRKHKNSSHRNSSNKNKNQSLLLNNN
KRPFYDESSNNVADYKPNLVTEKPVFAINRPTANSRPNNVNNKPNYEPKIEYDDNRSEYNLFTT
HNPLYHRPGVKPAPVDPNQPPITNKPATVRPDFPRPDTQRPEVARPVNRRPEVARPITIKPATTRP
FAVYPKPTDSSAIRYPVEKDTSPEVVVGADEDKMSSAEKRRYIDLAERMCDKYKALNVKKVE
AIPLLPSPQPVQVNVTTCSPTKIPLVVGGRVVTIEEFPHMALLGWTRLQGGGYSWKCGGSLISD
QYVLTAGHCAYQEKDDTVVIGAPRVVQLGSSTLDDTGALVMKVLSVVTHPKYVMTRSYYDIA
LVKMVRPVTFSQVVKPACLGVPPGVGEPIIASGWGRTEFGGTQSEELRSVSVPIWDISECGRVL
GTSRKLPNGPSPDSQVCAGEKQGGKDTCQGDSGGPAQIQDGCVWRVVAVTSLGRSCGAPNTP
ALYATLHRAFIAAQVFGKASGTSTTNHNSDRNENNNNPRPPVNNNWNNNNNRENTNNNRTPR
PPVGNNNDNRENTNNNHAWNTNQQNNNHNGNGGYINNQNWGNSQTTNKFNNPDWNANNN
DYTNNNQNSQNNGGYSIVTDSSNYGNNNRQQFNNDRLVNNDYNVNNDHKIVYPEVSGYNQG
NINNNNNYGINSYTTHRPSQNGGWWSGYDY
>OfuSP14
MRSFIVFAITLAVAAATETSERIVGGSLTTIEEYPEMVALLRVWGNHYGQDCGGSILNNRAILTA
AHCTEVTIPAGRRVRVGSSYRSSNGTVYTIERIIIHPGYAFNWPAQPDNDVSILWLTGIIPVTSTSR
PALIAGPNYNLADNQAVWATGWGWTQYGNFTSQSEQLRHVQVYTVNQEICRQRFANLVINGV
PMPYTITDNMLCSGILDVGGRDQCYGDSGGPVFHHGVQVGICSFGHECALGEFPGVNVRVSQF
TNWISENRQQITNLVINGVPMPYTITDNMLCSGILDVGGRDQCYGDSGGPVFHHGVQVGICSF
GHECALGEFPGVNVRVSQFTDWITDDAAGRIIGGSVTTIEQYPEMAALLSWSGTNFRQDCGGT
ILNNRAILSAAHCTVITAASNRRVRVGSSFRSWDGTEYSVARIINHPDYNRPTPMDNDISILWVN
GHIPTTTRTRPAAIAGSNYNVADNQVVWATGWGRTIAGDSNSMSEQLRHVQVWTVNQAICRQ
RYLNRVINGVHRPAFITDNMLCSGWLDIGGRDQCQGDSGGPLYHNGVVVGVCSWGEGCAHP
QFPGVNTVSYLINKMRFLIVVVLCLAVASAAKDNGRIIGGSLTTIYDYPDMVALLRGSVFYHQD
CGGVILNNRAVLSAAHCVGAWGIHARRIRVGSSYANNGGTVITTERLIVHPEYNVPTGHNNDIG
ILWTTEEIPVTAASRPAWIAGPNYHVADDQEVWAAGWGRTIANDGGSVSEQLQHVKIWTVNQ
ETCRLRYLQRSPIPNFITENMLCAGWIDVGGRDQCQGDSGGPLYHNGVVVGITSWGEGCADPF
FPGVNVRVSQYTSWIDSNR
>OfuSP15
MRGILVLLWGLAAVSAAPKSVERIIGGSLVSISQYPEMVGLLYSLTGVGHRQWCGGTTLNSRAI
LTAAHCTMEDPPFRWSARVGSNNANSGGTVIASQQIINHPQFTRADFNNDICVIITSTNIPFSANV
QPGRIAGTNYHLADNEPVWAAGWGTTWQGGWNSEQLRHVQVYSVNQEACRQRYAVTNNVV
NDNMLCSGILDVGGRDQCQGDSGGPLFHHGVVVGVCSWGRGCAQPQFPGVNTRVSRYTDWI
RQHAENILF
>OfuSP16
MHALVVLLMGLAAVSADAPAPVIQRIVGGSLTTINQYPEMAALLFSSSGFGHQQGCGGTILNN
RAILTAAHCTIGHTIARWRARVGSNFANREGTEINTAQIINHPSYNPWTYDNDISILRMASTIPIGS
NTVQAGRIAGATYNLADSQVVWAAGWGRTSANGQASEQLRHVQIWTINQAICRQRYATVGDT
ITDNMLCSGWLDVGGRDQCQGDSGGPLYHNGVVVGVCSWGRGCASPFFPGVNARVSRYTN

WITQNA
>OfuSP17
MRAFVLIIMGLAAVSAAPKNNRIVGGEVTDISLYPEMVAVLFSQTSVNHRVWCGGSILNNRAIL
TAAHCTYNRANSNFISRVGSTHVHFNGTLLVTQQIINHPNYNEYGFDYDISIIRTTTEIPFGANVQ
PGKFSGTNYHLADNQEVWATGWGAISMSGSHSEELRHVQIWSINQAICRQRYSALPNGMSWDI
TDNMLCSGWLDVGGRDQCQGDSGGPLFHNGVVVGVCSFGYGCALPGFPGVNARVSSFINWIS
SNA
>OfuSP18
MRTFIVLLLGLAAVSAYPKNIQRIVGGSVTSINQYPEMASLMFSWGTSGHRQACGGTILNNRAI
LTAAHCTIGDAAARWRTRVGSTNANSGGTELATQTIINHPNYNAWTIDNDVSIIRTASIIPIGSST
VQAGRIAGAGYILGDNQVVWATGWGTTSSGGSASEQLRHVQIWTVNQAICRQRYATVGDTIT
DNMLCSGWLDVGGRDQCQGDSGGPLFHNGVVVGVCSWGRGCAQAFFPGVNARVSRFTSWI
QNNA
>OfuSP19
MRAFVILILGLAAVSAAPRNDHRIIGGSITDISLYPEMVAVLFSETNVNHRQWCGGSILNNRAILT
AAHCTVNRANLNFRFRVGSTYAHSNGTVLDTQQIINHPNFHGLTVDNDIAIVHTTTTIPIGTTNV
QPGRFSGANYNLADNEEVWATGWGTTSMTGPGSEELRHVQIWTINQEICMQRYSGFYAVITAN
QLCTGWLDVGWRDQCSGDSGGPVYHNGVVVGICSWGFGCGWPQFPGVNTRVSRYIDWISQN
A
>OfuSP20
MRAFVILILGLAAVSAAPRNDHRIIGGSITDISLYPEMVAVLFSETNVNHRQWCGGSILNNRAILT
AAHCTVNRANLNFRFRVGSTYAHSNGTVLDTQQIFNHPNFHGITIDNDIAIVHTTTAIPIGTNNV
QPGLFAGANYNLADNEEVWATGWGSTSMTGPGSEELRHVQIWTINQEVCRQRYTGFNAVITD
NQLCSGWLDVGWRDQCSGDSGGPLFHNGVVVGVSSWGFGCGWPQFPGVNTRVSRYINWISQ
NA
>OfuSP21
MKWFMLFWISCVAVNAFPERQTKIVGGTETTVNQYPEVASLLYSYWGIFYNQACGGSILNTRSI
LTAAHCVVGDSASNWRIRVGSTYANSGGTVLSVGSIAYHGSYDSQTNDYDVAILRSATTIVFGT
LVKQSSIAGPSYNLADNQAVWAVGWGTTSSGGSASEKLRHVQIWTVNQAVCRQRYAVWGNTI
TDNMLCSGWLDVGGRDQCQGDSGGPLFHNSVVVGICSWGRGCALAAYPGVNTRVSRFTAWI
QANA
>OfuSP22
MKSSLLFLLVVAVAAAELLQPNTRYHETEGIPKFQLMKQLEEGTDFDGGRIWGGQAVSGGTHP
HLGGLWITLTTGQNSICGSSLVSNTRSVTAAHCWRTSTLQATMFTIVWNSNSIFWGGTRINTNQ
VIEHPNYNVWNLNNDVAVIIHNHVNFNNNIQPIALATGSQTYAGTWAVAAGYGQTGDGNSPSG
TKFQANLLVITNSACQGTWMPGIVIASTLCVSTAHGSSTCPGDSGGPLAVGSGNNRQLIGITSFG
TQWCAQHHPAGFARVTSFASWFNSHM
>OfuSP23
MRSFIVFAITLAVAAATETSERIVGGSLTTIEEYPEMVALLRVWGNHYGQDCGGSILNNRAILTA
AHCTEVTIPAGRRVRVGSSYRSSNGTVYTIERIIIHPGYAFNWPAQPDNDVSILWLTGIIPVTSTSR
PALIAGPNYNLADNQAVWATGWGWTQYGNFTSQSEQLRHVQVYTVNQEICRQRFANLVINGV
PMPYTITDNMLCSGILDVGGRDQCYGDSGGPVFHHGVQVGICSFGHECALGEFPGVNVRVSQF
TNWISENR
>OfuSP24

MLFWLAKTAAFVSLFVTTVHGDAALESWVPNHSAWEKLNALDCGESAADRIIGGTNAALGQF
PWIVRLGYVFPYDTETDWMCGGALVTDRHVVTAAHCIPTPDDEYTLKYIRLGEHDTRTDPDCE
LSVCAPPVQDREIKNISKHPDFNQPPFHNDMAIIELDTPANLNDYVTPICLPQKGDQLNGVHVG
ELVSAAGWGKMNMTTEERADVLQVVALPIVEPDMCDLFGQEFKVSKSEICAGAQYNKDACG
GDSGGPLMKVFDTSDGPKNFLVGVVSFGPTVCGIRKPGVYSSVVHFLKWILDNIDYNPMALD
KIWIVSAVIIASWKARSHETIPNNTVTPYSNLTRPFLENDDQTYRHISDVSETKNNNVYKQDKE
NRAYEVKNTHPNRLDGAVKLPDRSVCGPLSEEERIYGGENTAIDEFPWLVRIKYILDNGKEVYA
CAGSLLTDNYVLTAAHCAVNLTIKEVRLGDWNLKTEYDCQGSVCIGHAVDVKVVKVIPFPNYT
KMDTFKGDISLLKLQRPVNFTDFIRPICLPTTEFVANQDYSPGSTYWTAGWGKTEFEKKPAIKR
KVELNAVPVPECRRKQPILSDDTVSFTICAGGTDGKDTCVGDSGGSLVKQVTENSTTNWFLMG
VTSYGYKECGSRPGLYARITPYMDWIIHNIGSYFKTTFILVFLL
>OfuSP25
MWVAVALCLLVQFLSSEAKVAVLPMLRHNDGIVGGEDIDISEAPYQVSLQVNGRHSCGGTLVA
KHIVITAAHCIGTLDASKYQIRAGSSFSGRDGELYPVAKVLRHPNYGQNNFDCDIAIMWLSKPV
RLSDKIETVEMKGVGEEVPDGDIVQVTGWGQTTPERSWVYNDRMLQRVLIPKIADSKCRDAY
DDLFTDNMLCAGLPEGGKNACFGDSGGPLIHNGKLAGVVNFGYGCARPEWPAVYAKVSALR
QWVDEQLKLTVPDL
>OfuSP26
MFQLLCSVAFFGLIVASPTSRVHDGRIVGGEDIDITEAPYQVSLLYRGRHSCGGTLVANDIVLTA
AHCIMGSDPTNYQIRAGSSYSEREGVVYPVGEILAHPDFSFSKMDNDVAIVWLSQPVTFSDRIA
VVEMASQGDEVEDGELTEVTGWGNIREGGGIPTMLQKVLVPKVNSMACGKAYAPMYTITPRM
LCAGAPGGGKDACQGDSGGPLIHNGKLTGVVSWGLGCARPEYPGVYAKVAALRRWIDEHILY
LRLKNIMRW
>OfuSP27
MWVAVALCLLVQYLSSEAKVAVLPMLRHNDGIVGGEDIDISEAPYQVSLQVKGRHYCEGTLVA
KDIVITAAHCLNSYFHLFKCLIFRKVTKLNLKFLVAIYAQFPVSIEKLFCFSLDASIYQIRAGSSFS
DRDGELYLVAKVLPHPKFDYEEFDSDIGIVWLSEPVEVSDKIATVEMKECVDEVPDGDIVQVTG
WGWTEPIRSLAHNDLMLQRVLIPKIADSKCRDAYDHLFTENMLCAGLPEGGKNACFDSGGPLI
HNGKLAGVVSFSRGCARPDYPAVYAKVSALRKWINEQLNGDKKP
>OfuSP28
MWVAVALCLLVQYSSSEAKVAVLPMLRHNHGIVGGEDIDISEAPYQVSLQVKGRHYCGGTLVA
KDIVITAAHCLNSLDASNYQIRAGSSFSDRDGELYPVAKVLPHPKFDYEELDSDIGIVWLSKPVE
VSDKVGCWLTPVEMKGVGEEVPDGDIVQVTGWGSTEPARSLAHNDLMLQRVLIPKVADTKCR
DAYGKYFTGNMLCAGLPEGGKNACYGDSGGPLIHNGKLAGVVSFGIGCARPDYPAVYAKVSA
LRGWIDEQLK
>OfuSP29
MASFKDWLSVTWAQGGMQLQAAAAGDDIDSVKGRCGRAGSLAATPGEKLMELLRPSAGTPR
RHSTAACAPQPPRPSGFVYCPSDALPYCPPSRITPQRPILKLQPPPPQIQQPPAPPQPPPVPQRTAP
VPPAPLPAPPPPPPRRASPQPPRRAPPPTPPRPPATSPADPNGNRRTNPQLPRLDCNRNPQPPVPRRP
PQKLPDSYTPPPPPPQNNQTIKRSPSSGSNISVRQDSNVSSDSFSQTSSPSYTTKTMEAPLLPHQH
VNKSLNAKIARGLLLKEQQEKEAGNSSITKSMSTPASLQTIVRFQNGSNMSLHHRMLRDMRNA
GTEGSPHKFRVLQLALNAVALLAITGALFAYFRANPAVQYVSQVVNRSTVVTWPAPTEPPGAR
NPAPGVCLPVIVSFCQQHRVTYNFTVFPNYIGHFGQRDAQQDLEIYDAVVDVRCYELTALFLCS
LFVPKCGPLGHMVRPCRSLCQETMRRCGFFLEVFGLSMPDYLQCEIFPESTDTDVCLGNREVK

EARFRAAKPVCPTGFQCDVKRCIPHDWRCDGHVDCADRSDELNCRVCKRSGDVHCGNQRCIS
QAHMCDGRVDCPWGQDERNCLRLSEANGDIGRGELQVYRAVNQSWYPACLGALDNNTALKL
CSMLGYSLLNKSFVQGGLPAGSRAGRASLSGVAQTYRAFQRSEGGLLRELKECRHDTAKVHL
VCNNYECGRRRTVGGASKRIVGGVEASPGDWPFLAAILGGPEEVFYCAGVLIADQWVLTASH
CVGNHSDVNGWTIQLGITRRRSHAYYGQKVKVRRVVPHPQYNIGVAHDNDIALFQLAVRVRY
HEQLSPVCLPPADRALAPGTVCTVIGWGKRDDKDSQYRSMSEYEPAVNEVEVPVLNRDLCNQ
WLEHRDLNVTAGMICAGYPEGGKDACQGDSGGPLLCKDPDDPSRWFVGGIVSWGIKCAHPRL
PGVYAYVPKYIPWITDQIRAYNDDHARSDDV
>OfuSP30
MLVFVILCFLSVDVTDSALRVLLGRDAEQTEFPYVVRMEIRHKAKNKTQLVLQNSHMCTASAL
TPTWSLTAGHCIKHLNLIITEKWVIDPKGVVRYGSPSGIPSQNDKFSDIVSYVMHPAYLSMYAM
NGVRVHNDIGLLKTLPVKLEHYAKLSAVDYNTMAGQAAKALGYGLMLMEKEIKNTLQLGRA
LQVLDVVVQDCAFFIKKNKMIYPGLCLNRRCGNPSSLCKGDSGGPIMHFSGILGVISTGISSDC
KVKHVKKGISSNIVGLITPVSPFINWITDHIKTTSDQT
>OfuSP31
MNPSLAERFQDTDQNNEDYKADDPCNYDRVNHTRPESSRKGKRVSDIKCDEYSWDYWTRIIE
SDRRAEECKQQLKDPSTVFGGMDAKYGDFPHMGALFGISKSNKTVFECGCSLISYKFALTAAH
CVTIMEYRPYRIRFGNTSGSEGKGVSLDVGIKNIFIHPNYSQPYKYYDIALMELERELKTQAHIR
PACVWSGAVGNKWVANITGWGKTTPESNSASNNLNYAEIDIWEKGSCNKSLKGRPNRLWKDG
LKDHQFCAGKLGVDTCQGDSGGPIGIRRPSNYGESGFMHHVVGITNFGVKCGTKLPGVYTNT
TTFLCFIEKTVWPEEPNEFLTYVCIYAQKQATNSLKERSQFAPRQFSSNVTMSVSVFIAAIILAVG
TQVLTQNDSELDYNFIFEEDKIVPIPLVTTEKSNNTPKAVVATTTNVPQVAKPGVSVSGIAPTHLN
GSEITTESSNLTSKKFDFDDWDLMNENETMYDGFNPEEVEKLRARKRTKAPTTTTTQPPPRYTT
PLVLLVIDGLDLESEESDQKTTPRMTPSTTISTSSTPRITTPPVTDGSGGEDDERAGPGDDGEGIQ
PITDEPDYDSEEKKDDPCNYEKYPHPLPNIIRTNRRVSAIKCEEYMWHYKIEEIDRERNRLKCKE
NSPDDTSEIFGGAKALQGDFPHMGAVGWVSLDGGMKWMCGSTLISPMFTLTAAHCRLASRR
MPLKYFDPKIVRFGSVFLYDTNRTRYKDVKIKEFLVMDEYRKNPLQKYNDIGLIRLEEEVKMT
LTIRPACLWSGYLVGEVANITGWGKVTTNEDSASKYLRYAEVDVYGVDTCKKYVRGRNNRK
WPKGLVEHQFCAGKLEGGIDACQGDSGGPLQIRQPMPMDNTHIFHYVAGITSFGIKCGIPNLPG
VYTNVSHHSFMCWIEKTVWPEEKNDFC
>OfuSP32
MAVRILLFFFCLVMTTHGEETAFGYHAKIGIPKAKKLLMAESMTRIIGGSAVSANTAHPHQAGLI
ALLTNRATSICGGSLISNTRILTAAHCWDDGQNRATQFTVVLGSTTIFTGGTRVVTKDVTVHAN
WNTQSVTNDIMIARITSVTFSNSIQAIALPTAAEASLNFAGLTGTVAGYGKTKDAQSSFPTTTTL
HSVNLPIITNAVCQSSFQMALHASHLCTSGAGAKGTCDGDSGGPLTVVSNNRLVQVGIVSFGLS
DSCQSGHPSVYTRITSFLSWISANM
>OfuSP33
MVKPVFVNESSLIPRIVNGYPAALGDIPYQLSFKQRYSRHSKVYDTFCGGSIISPSKLVSAAHCF
DHNKKSGCFTETIILSTKEVGVYFAVAGTLVSKERYRPQDNPSGAQWRRLKNVIYPSGYDFPEH
DVAVVTTLQPFVFNAHIAPIPFAKRWRDYRGECVASGFGLISHIPEIDSSKLMIAKLELVPSYWCS
KKLSADMKTFVCTSGVKTDVGKGDSGGPLVCAGTGEKSEGPNGILVGIACANILKRSIGGSIFT
RTSTYTRFIDKNEANSSPFTKVQQKIVLV
>OfuSP34
MLFIRYNVGPVLTLLCLFEASWSQILEYPVFVNLSDLIPRIVNGQPAALGDIPYQISFKMLLRRTA

GANRYYSFCGGAIISRKKLLTAAHCFEAKQSACCTSTITQTGKAVSKYFAVAGSLKNIEVFRGAN
PKEGGQWRRLEKVVYPSSYKFPKGDIAVVFVKNNFRFNDNVKPVSYERRFRDYKGSCMMSGF
GFVDRKNTKMSDQLLYAELPLMTSFWCNKNHNMNMRKHICTSSEISDTAKGDSGGPLVCSFP
NQNESKVLVGVLNGKLLYNTKRLAGGTIFSRVSAYSKLIRKSKACSYNIASWAQILEQPVFVNV
SDLVPRIINGKPAALGDIPYQISFKRLQKRDKSKNMYSSFCGGAIIGTKKLLTAAHCFKSDESNC
FSTYIVQSGKTVEKYFAVAGTLKNEEAYTGDDTSGGAQWRRLKAVTYLGSYKFPKDDIAVVSV
KEPFSYTTNVKSIAYARRYMDYRGSCLMSGYGRISESVCD

>OfuSP35

MYWFSGCGGALITLKFVMTAAHCFVGPDFPKIKNNIRAVAGSTRTDASLYTMMREHWRRIKH
YYPHKHYDPSTYMHDLAILEVMTPFFLSVRVKPIRIHSYEIRLKMSEGMNCLVTGYGYISKTKR
ASRLQKVCVPLVTRYNCSFYYHPKYLHDSVFCAGTKGKDSCNGDSGGPIVCHGVLVGLVSYG
GECGVDPGVYTRLSSYTHGEPIPFILKQDNASNGISLNRIVLLFLLAINIL

>OfuSP36

MPPWAMYPIRIQTQRHISQFFAVAGTLLNKDVYKPKDNPSGAQWRRLKMVISPSTYKFPKDDI
AIVITTEPFHFNALIKPIPYATRYIDYKGECLVSGYGRIHSIKPISSEKLLLARLELLPTYWCTNLH
GRNMRRFICTNTKVSDVAQGDSGGPLVCRSTGEKHEDSTFGILVGIVSGTRLLRYKGKSSFFTR
VSYFSKYITRENGAEGSTFVYVYHYVIVLNTFYTVF

>OfuSP37

MTTAFPPHIDSSCCGVDANSASRIFGGNDTAIDEYPWMALLEYKRKDNGLIKTYCGGALISNRY
VVTAAHCIKGKGAWPPINVRLGEYDTSSERDCVDSDCADPVVTIGIEDIITHPEYNADNSRHDI
ALIRLASPAPYTDFIRPICLPASDISSQDPADLEAYVAGWGKVNMTTKSTIKQDLKVPIIKTEDCQ
KSFTSRLSKPPTLWSYQLCAGGEAGKDTCNGDSGGPLMLSTGTRHELVGVVSFGLVNCGTEGL
PGVYTSVYPYSAWIRRHLRP

>OfuSP38

MLARANPSVLAAALAACAALATARPDYANSNTNRYVPANNANLNAVLPLTSTDLANTPAAYLF
EEPKTKSIPNETPRRVTEVTVTDEKGSENNIPTSILATTDNGETKATVQADGSTKPLFHPGSIAGK
PSGVNPFSSVEPSQVPSLFRDDSKPVNLRYPHAVLFGGTCGGTIIHPKWVLTAAHCTIHSSTLFT
GGRYVLAGTNNSDDGSGVTKKVKKLHIHPLFTVGPYWLNAKNYNITQVAARWDFLLAELEEP
FKLDGVNIAAVRLEDEIKQSPDMYVGYAGYGTEHHGGFMRSEMHAMHLRTQSDATCKKLKQ
YNPHDMLCARGYAPNYDSACNGDSGSGLVNGNQKLLGVASWVENDSIECRNGNLVVFAKVA
RAREWIRDIAGV

>OfuSP39

MFWELENSMSLTKEQKYTEEERKCEEFYVKTTTRDKEGRYIVKLPFKTENPECKRGETRTPFL
AVRTLQQVAKDEGLNYPLAVDKVKENFYMDDMMSGCESVTEGIELYTQMKSLLNKAGFRLQ
KWASNDEELLKMIKSRENEQENGKEKEECKDGITIKMDEVIKILGLTWERHSDHFRYKVDLPE
LLPPITKRKIIADISRLFDPLGWVTPCIIIAKVLIQKLWIAGIDWDDEAPSKILQEYCTYREDLKSIS
EVKIPRWLGTRLNDVKRELHGFCDASKVAYAAVVYIRTIDAEGNIHVSLVTAKSKVAPIKQVSIP
RLELCGAVELTRLMLETSKVLNIEHTNLHAWTDSTIVLAWLNSLPSRWKVFVANRVSEILTNLN
PNQWSHVSTQENPADYASRGVTPSELKNKSLWLNGPEFLMTKNINYKKPTELTTEIEAIKVHNI
TTEEDFFERFSKLRRLIRVTAYCRRFLQMKKPKSERKDNEYLTTKEMEKALEVCIIRDQKIKFEE
EIEAIKKNIPIKNNSLKSLNPFIDETNLLRVGGRLEKSHMASSRKHPIVMAKSSKLTALLIADAHH
QTLHGGPQLTLNYLQNKYYIIGAKQLVKTHVRKCVDCVKNKGQTYQQLMGQLPAVRVTPARP
FLRSGVDFAGPIQIRTTRGRGYKSYKGYICLFVCMATKAVHVEAVSELTSHGFLQAFKRFVARR
GPCSDIWSDNGTNFVGAAAEIQKLFSNEKDSILPEIAEQLANNNTTWHFIPXWRRSMESAPQNR

RGSTRRIVSGRNTSIAAVPWQVSLREKTYPICGGSVITDLWLLTAAHCLLRARSSELSVRLGSSW
KTHGGEMYDVKECFVHPRYVSKTKINDVGLVRLYSPLRFSEKVLPIKLVAREARLPADVPAIVS
GWGKLKEGGPSATYLQSSTIKTVAMKLCKHSGLDRKAIDPPSMFCAGSFTQPSPDACQGDSGG
PIVSDGVLIGVVSWGLGCARGNFPGVYTRLSHHIIWDWVHQHITRKPDT
>OfuSP40
MRLTLLFLAIFSLSQSWAWDSEADTLNVVDLSQVNPDSRIIGGTSTTIERFPYAVQVQRSNQLTC
GGTLLTNRAVLSAAHCFVDRFTPRPDPSQYTIRAGTALRGSGGTTSRVSVIVVHQSYNTVTHDA
DVALMVLASRLSFSTRINRASLPMQGQSLPVNTGLIHVGWGTTIAGINALPTTLQEVTVRKVN
WTTCAERYRYLQAITGEPFLVTTNMICAGLLDVGGADACQGDSGGPLLYGNIVVGITSWGYSC
GHPSYPGVSARVASFTNWINATVNQYSSSPRLSGSTTATSLLLVTSLLASFTLMIKDF
>OfuSP41
MSAKIYFVADLVKVLIFFLCLRISHSGRIRRIVNGMPVLCGEQPRVASIRNSTTGQHLCGITLVSP
EFAITAAHCVLQPVDQYVLRLNNYCVGENETYPEAEVVDIIIYDQYDRYSSTHDIAILQIRLELN
NVTWLNESVLPASSFGLSSDDCSIYGYGYTNPLTAEVSERLLEGRVETMSLDECIERLGPYVAPA
YDSGMMCAVGESGADACQGDSGGPLFCGNSTILQGVSSYGMSCGVSGLPGVYTSIGAHLNWI
RTVLNKGTQNQTTE
>OfuSP42
MCNEIPRGAPPAVQPKENNQRDLILTVLTAAHCNAAVFSFGSLSSSLRVTVGTNNWNQGGVAYA
LARNVTHEHYVSQIIKNDIGVLITSSPVVFTNLVQPITVSYDYAGAGIQSRAAGWGRIRAGGPIS
AQLLELTVTTISGDQCVRDVAQASVDFNVAAPPVEPHVELCIIHSPNHGMCNGDSGSALVRLDR
GTQIGIVSWGFPCARGAPDMFVRVSAFQDWVARHFVA
>OfuSP43
MALKYLALLVLCVVKVNCMALKGPPLTRYDPCGLGVIHFDRLMTNYWQSVLHLGLYKNLLE
VEIEIYFEKKVTIFGVSHNSSVLSLGEHGFRIQPRGPVPQQFKFYMILEGLGENDPDVPTVTKFT
MNNVTLCSADIKKNQTVESLDVTKKDEKHHKHICGRRSLDHTELVSVRTEAKAGDWPWHVAI
FIKDLASNTMSYYCGGNIISKTAIVTAGHCVIKDGQRVTADRIVVVAGTNNHKEVGQIGRQSLP
VKEVVLHPNYDGQATSDVAILKVDRFKYTAYVQPICVWGPVYDKQNLIGRHATVVGFGHDVD
NNPSDVLRAAYIMVQNDTTCVDYSPSIYKDLLNEFTFCAGYGPTSGNNPRNGDSGGGLVVPV
MQLDHKVSWFLRGVLSKCGVAPGHTECDPRYYVVYTDVGPHYGWIYHHSGLVFGTNVLSDH
A
>OfuSP44
MCKVSYLALGLVLCVAINNDVLGKRHGHAVKNNGGKIVGGYNDTIQNVPYIVYLLVLMGTDY
YQCGGSIISSRYILTAAHCLTGVSRVYVRAGSDDSESGGKMSSTSVYRQHPQYNPATSDYDVAV
VRLTRPLTLDGTTMKAVTLPEAGQEVPAGTELFVSGWGDTTENGQTSRYLMSVKIPTVSTADC
RQAYGQNAITERMICAGVPEGGKDSCQGDSGGPAVNDATGLQVGVVSFGTGCARPGIPGVYT
NVSSVRGWIKRNTGV
>OfuSP45
MVLTSDMAKFLVLAVALLAVSSCSAFHRIIGGQEATIEQYPSIVQVEFSNFLGTTWSQSCAANIL
NVLYVLSAAHCFEGATYSPRLRRIRSGTATRNNGGAINYIEREINHPEYRVAARYDADITVVRLV
TPFVYSLQVQQGVIVYQDATIPDGLEVVHAGWGTTVAGDSSTMSSVLLDTIIYTVNNNLCRER
YLTLPNPGFVTANMICAGLLDVGGRDACQGDSGGPLYYGNILVGVVSWGHGCANETFPGVST
NVASYTNWIAATAV
>OfuSP46
MVRLVLLTLALFAGCCYAAPRIVGGQETTINEYPSIVQVEFLGIFSQAWSQSCAANILSSRYVLS

AAHCFAGIFYSPSLRRIRAGTTFRNSGGFTRNVANEYNHPTYGLLGADGDITVVRLAEPLEYNP
VVQAGYIANPNTVIPDNQPVIHAGWGHTQFGGHPSDVLRHVTIFTINHAICRERYATLGWPVTE
NMICAGLLDVGGRDACQGDSGGPLYHGSTLVGVVSWGEGCANATFPGVSTAVAPYTSWIVSV
AV

>OfuSP47

MASVFVLALALFAATASANPARIIGGSSTSIETWPSIVQVESTVGGIFWSHHCAGSILNSVSILSA
AHCFAGTGYDASNRRIRAGSSTLESGIILYVAQENNHPSYGQNGNDGDITVVRLSSALIFSSTIA
AGSIVAAGTTIPGNLPVTKLGWGITAVGGDRAEELQSIQVLTLDNSVCASNYATLSSSPQVTSNM
ICAGSSTADACVGDGGGPVYFGNIIIGVISWGNGCLQDGFPGVNTAVSPYTPWIVATTSSNVCH
QRLVVEGSVKMASSLVLIFALFLAAAQSAPERIVGGTTTVIENYPSIVQVEFRTGILWGQSCAAN
ILNTRYVLSAAHCFSGLFFSVRNRRIRAGTTNRNNGGTTISVDAVFNHPTWGSLDNDGDITVVR
LSSALVYSDRIQQVAIVNQGWEAPDNSPVVHAGWGTISSGGLASALLRHVEINTINRELCRARY
APRPRSSITDNMICAGVLDVGGRDACQGDSGGPLYFQGILIGVVSWGRGCADAFYPGVSAAVS
PYTDWILATAV

>OfuSP48

MSLFKIIIVTGTFAMARLLALSLLFLAGACYASNRIVGGNPTTIEQYPWMLQVEGQVIWSGAW
VQFCAGNILNQAFVLSAAHCFDGPLYAPHLRRVRAGTTYQETGGVLAYVDTVFNHPSYGLIGY
DGDITVVRLSNFLPLTPVIQQATLIYQGAVIPDNVPVVHAGWGAIAHNWIDSEVLLDVQIYTINN
DLCRERYEMLDEPWYVVTENMICAGVLDVGGRDACQGDSGGPLYFQNILIGIVSWGHGCANE
TFPGVSTSVASYVDWIVETAVL

>OfuSP49

MNIQVLLLMILTLCAARISAATARLVGGKDAPTHFGRFHASLQNFTGHHVCGGVIVSHHHIVTA
AHCVLGAEPQYIKAVVGTTNLDYGGQHYDVSSIYIHDEYNITSRINDIAVIKVIGQFNLRYVDIL
TFYENELEEGDQVILSGFGAEMPNGESSRTMHVLNLPVFNQETCRYAMRYSREVTDTMFCTFT
QIGEGTCHGDSGGPLIKNYQLVGLVSWGIPCAVGFPDVHTRIRPYVSWIEEQIK

>OfuSP50

MFTRKLHMYQIVIIVIVNIVIVNDVLCIEFIQLGDSGGFEESPVGKQYESIYQKDEPEFLIEDKVPI
IREGFNYDSKEDDDIKTLLEEKYKFKNDEMSTRYRISNDEGGYPDDRNIAWAFRSYAVRRIVGG
METSISMYPYNVAISRNGKHWCGGSIIDEQWVLTAGHCLESAYDGDKKKLQPFIVRAGSSFHN
RGGYQARVNKVFFPKHYSPGSADFDYSLLRLDRPMPIGRNIAVLNLPSKDYVVKEEDILIVTG
WGSTDESGFGHIPDRLRFVPVPVMVIDDCQKSYRFYITPRMMCAGYATGGKDACNHDSGGPA
VRDGVLLGIVSFGGKQCGDPRSPGVYSRVSEVTDWVEETITKNEAQNVPELQAKIKKARMRE
RELQKFKARVEDKKNKIKSWLRDTLKSPTFIKLAKKKLKEAGYQTRRFHVVETNASALDDQA
MDEINLSNLIHERIIEGDDGNEAEKLLRTLALQEVMLNNNSEEIFYKDMESESMETFSLCVYCQ
HDKNGEVERQNRDILKRLKISQVEKKNWKEALLEYMAMYNSTPHTVTGKTPAELFFRRQFRD
KLPMIQDMTHSSADLEMRDRDKELKEKGKEYADMKRRATGCELEVGDKVYVKNMTKQNKL
SLNYEPETHTVEENKGGDVSLRNDETGQRIRRNVVHLKRVEGQWKVLDEENNASDAQTNDS
GDVNQSQLGWIN

>OfuSP51

MVFLKCIILFGLLGHIANGSPLKSEIMDLNPHIVGGVDAPDGYAPHSMALTSGYWVNSLMGASI
ISECHVLTATHCIEALLDISGQLLSTLHGVYATNEWQSTKHRTKFSGFINHEKYDAFLYKYDIGV
LFLDGKLTPSDKWAIIALNFDWIDAGDKSSVTGYGRLWNWGPIATRLQLLYLKTIGEKECADG
VSAAMPPGWWIPPVDQRVELCTLKGEGYGLCNGDSGSALVSLKTNTQTGIVAWGFPCARGAP
DMFVRVSAFKDFLLNITKTCDKC

>OfuSP52
MKLHLLLIVLATASAHYHARIGVPAAARIWQLENGGETRITGGLPSYRSLHPFFAGLLIALPMN
LTSVCGSTLLTDTKLITAGHCWNDGERQAILFEVVLASVRLFTGGTRIITADVVPHEAYVPETIY
NDIAMITVPPVQFSELIRPISLPVENLFSSHEGRVATVVGYGKTGDASEITGSQSLRFAFVTVKPK
EECAIYGNLFGASMICTNGIYGGSCGGDSGGPLFISERSGEKYLIGVVSYGSAYGCEAGHPTVF
TRISSFIDWIYDKICY
>OfuSP53
MLNLHVALIFCAISAASCLTVNVAESSCSSSYSEFQCSDGRCINVTSACDGTDDCQDGSDESKC
DSVHESTLESNSVSLWSDNQSPCGKNEWQCKNGLCINIKGKCDGIRDCPDASDETVELCSGNQ
CPPNAYRCYYGACVDRSALCSGVKDCADDDEASTRCNNKTQAFSTRDKREVLRRWKRQAGC
SKNQWQCRDGTCIAFYGKCDGIRDCPDGSDETFPLCRNSICQSNWFRCTYGACVDGTAPCNGL
QECVDNSDELLPRCRNESAVSAGKFRCDNGQQIAAYLLCDGVRDCGDGSDETVRACAGNICS
AHLFQCAYGACVDSGADCNGKQECADGSDESDELCNRILPVKPTPAPGGACVLPPYPDHGSYT
VVGKPTAVPGQAFPTITYNVTCYPGYSIIGDTTERLCLEGVWSPEVGDCVQFCRLNKDQSVTY
RCLLTGDSEGFRPCGEYEPAGTVIRVDCRTPDYYYNGNLPYMRCIAGSWDYTARCLPECGVVA
IGDGLISGGVAAEKAELPWHAGIYRTTSTPYRQICGGSLVSTKVVISAAHCFWNNTIVKAQPAS
NYAVALGKLYRPWLLEEDKRVQKSHVADIKMPPHFLGGATNYQDDIAILLLQTTITYSNLVRPV
CVDFGVLFEKRQLKEQSLGKVAGWGLTGADEPESPFLKVVHLPYISISECQSKAPPDFRAYITG
DKICAGYENGTSLCKGDSGGGLAFPDSDKGTIRYYLRGIVSTAPDDDHLCNVNWLTTFTQISRH
ENFIKEYI
>OfuSP54
MLNLYFFLIFCASCLTVNAAQSPCSKNEWQCKNGLCINIKGKCDGTRDCPDASDETVELCSGN
QCPPNAYRCHYGACVDRSALCTGVEDCADDAEASTRCNNKTKAFSTRVKRDVLGRWKRQAG
CSKNEWQCRYGTCIDFYGKCDGIRDCPDGSDETFPLCRNTICDSSWFRCTYGACVDSRAPCNG
LKECTDNSDELLPICRNETAVSGGKFHCQNGQKIAAYLLCDGISDCGDGSDETVRACAGNICST
HLFQCAYGACVDPGADCNGKQDCVDGSDESDELCNRIWHPNSTPAPGGACVLPPYPDHGSYV
VIGKPNAVPGQAFPSVAYNVTCHPGYSIIGDKTERVCLEGAWSPEVGDCVQFCRLNKDQSVTY
RCLLAGTSEGFRPCGEYEPTGTVIRVDCRAPNYYYNGNLPYMHCEAGTWDYTARCLPECGVV
PVKHSLTSGAGGVAAMTAELPWHAGIYRTTSTPYRQICGGSLVSAKVVISAAHCFWNNTIGKK
QPARNYAVALGKLYRPWMLEEDKRVQKSDVADIKLPPHFLGGATNYQEDIAILILKTTVTYSIY
VRPVCVDFGVLFEKRQLKEQSLGKVAGWGLTGPDGPESPTLQVLHLPYVSIEECQSKAPPGFTT
YITGDKICAGYENGTALCKGDSGGGLAFPESDKGTIRYYLRGIASTAPDDDHLCNVNWLTTFT
QISRHENFIKEYI
>OfuSP55
MLSAIIITFFATTVTSAVVTESTNSCSASEFKCGDGSCIGLMSVCDGRRDCMDSSDEERCGGVGS
NSMLSDGVLHRWKRQATSRCSKSQWQCRDGTCIGFDGKCDGVKDCPDGSDETHALCRKSHC
QSNWFRCTYGACVDGTAPCNGIQECADNSDELLPRCRNETSEVRGKFKCLDGTTISASLHCDG
VADCPDGSDETVRSCAGNVCPAYLFQCAYGACVDMGSDCNGIQECADNSDESDELCNRTTTV
TVAPPVTKKPVTGGACVLPPYPAHGTYVVNGVPNAAPGQAFDTIGLNITCYPGYGLVSKKRDR
ICLYGLWSDEVTDCVRFCRLNKDPSVDYRCRLTGAAEGYRECEDYEPDGTIVRPECRSPNYYY
SGVLSDMHCIDGSWDYVARCLPECGLVTPKGVELVIGGRPAERGELPWHAGIYRKTSTPYMQI
CGGSLVSAKVVISAAHCFWNDTSKAQPASNYAVAMGKLYRPWNSPGDLEVQKSDVATLKMPE
RFLGGTTNFQDDIAILQLVTTIVYMPHIRPVCVDFNVVFENRQLHEGSLGKVAGWGLTGENGE
ASQVLKVVELPYISIQECQSDSPLDFREYITGDKICAGYRNGTALCKGDSGGGLAFPEPDKGTIR

YYLRGIVSTAPNNDNLCNAYWLTTFTQISRHERFIKEYL
>OfuSP56
MRSFIVFAITLAVAAAAKTSERIIGGSLTTIEEYPEMVALLRVWGNYYAQYCGGSILNNRAILTAA
HCLELPIPAGRRVRVGSSYQSSNGTVYSIERIIIHPGYANNWPAEPDNDLSILWLTDIIPVTPTSRP
ALIAGPNYNLADNEPVWAIGWGWTEYGNFTSHSEQLRHVQVYTVNQEICRQRFANRVVNGVP
MPYTITDNMLCSGILDVGGRDQCYGDSGGPVFHHGVQVGICSFGHECALGEFPGVNVRVSQY
TNWISDNR
>OfuSP57
MWFAVILFLQAQFFNGHAGVVGSFSRGNNEIVGGEEIEITDAPHQAGLLFKGDFICGGSIVAKD
VVITAAHCLSTTNLLDYTVRVGSSYSGSGGQLVRVAKVLSHPDYNFNNDIAIVWLSKPVTFSDK
VAAIPMKDSNEEVPDGAMTQVTGYGATDPWFKLQNYNVKLRRVMVPKVNHDVCQKAYEPT
KITPQMLCAGFPEGGKDSCQGDSGGPLIHDGKLAGIVSFGAGCARPDYPGVYAKMSALRKWV
DDQLHLRKLEQILRADSN
>OfuSP58
MYSENAWKVILLVAATAAAGEPSRALSLVYQKPFRNVMFVPRKLYRTRSRQSRVLFLDDNENH
QPFGRGEIDLTLDKNSKVKFDDASDYEFTSPEDGDTTTKSQLDETPSGNERFYEPLPRFRPYQSF
PSYYQGRFSRPYAPTFGFDPYQPSFAPAVRNGLYSGYNGWKARSPRVVFPYASDSVNSVLHTNS
HAGPGAFDNVVFRDQNFGINDVGTDEQGLQDINGGNDAFTERGCGIAVGKQTAQRRIVGGDD
AGFGTFPWQAYIRIGSSRCNMLMSIFELIGWKQCWIERCGGSLISRRHVVTAGHCVARAQPRHV
RVTLGDYVINSAAEPLPAYTFGVRSIKVHPLFKFTPQADRFDVAVLTLDRNVHYMPHIAPICLPE
RGSDFLGQYGWAAGWGALSPGSRLRPRTLQAVDVPVLDNRVCERWHRANGINVVIYPEMLC
AGYKGGGKDSCQGDSGGPLMLERSGRWYLIGVVSAGYSCASRGQPGIYHRVAHTVDWISHAT
TLS
>OfuSP59
MRLVVVLAVVLSVSTAADFESEHGSRQKRQDDRWIWGPFNDAPVVRAQSEQITATEAPAPAKS
NPCDPFNPVKPDFRAPGRRKSDVKCFEYIWEMKRRNDQQESHKKCQEYLVESGKLPFFKHLVI
GGKETLPGEYPHMGAIGWKASIGTWIFKCGGSLISEKFILTAGHCSKASSRDTSIADVDPKIVRL
GDKNILDISYNNLGPRDYNISRVIVHPLYKPPKKYNDIALIELATPVDFDKFTQPACLWGRPDLQ
GINSAFVTGWGVVETAGKTTSPELQAASVDLVDSQQCDSLLRPSCNRHWCGIQNHQICAGKLT
GGVDACQGDSGGPLQLKVSLPINDQGSMNQVIGVTSFGIGCGLPNLPGIYTKVSSFIDWIENIV
WP
>OfuSP60
MKFFAICFLLVTTAVCLPASPEELSARIVNGFEVDITQVPYQATLQRSVSGGWAHSCGAVIISSRA
VLSAAHCVVNFVNQPSLIRVSVGTSRRLSGGTRYDVSKILSHTGYSVTTLEHDIAVIVTSTNIVF
SSSVAPIDLVPSGLTFPDGYEALVTGFGTVASNGSASSVLLAARVNLIGQAACVRAYLRIASITSG
MICASGFNPPRDACQGDSGGPLVADNFVIGIVSFGEGCAEAAYPGVYTRVSEYYSWILQQLSQI
>OfuSP61
MMKWNWLVFLAAVAVEAGPFKGSQWSYHESVGIPRSEQLKKLELAANSTRIVGGTPVPLGRH
PFMVGLVIHLNYVDWFTSMCGASLLTHTRSLTAAHCWDDARMFTLVFGSQTLHTGGLRIDSED
VEVHPDWNPDNLHNDVAIIRHDWVQFNNAISPINLPIEDANNDFLGMWAVAMGYGRISDTNPN
PANPELREANLQVISNEECVQRYPNNVVSSTLCTLAPVGINTCVGDSGGPLVAGSGPDKVQIGI
VSFVVWGCEDGFPHGFARVTSFLPWILSRQ
>OfuSP62
MKWCLLLFLVAVVVEAVPFEGPRWSYHEKVGIPMSEQLKKFELAANSSRIVGGTSVPLGKHPF

MVGIVIHLFTPATSVCGASLLTHTRSLTAAHCWYDGWRWARMFTMVFGSQRLHIGGLRIDTED
VEMHPDWNTLNLNNDVAIVRHDWVAFNDITRPINLPIDQANNDFAGSWAVAVGYGRQFDGQV
PTFNPDLREAHLQVVPNEVCWQLYPSLVADSTLCTRGPIGTNICQGDSGGPLALETGDDRILIGV
VSFGAISCEGGHAAGYARVTSFLPWILSRN
>OfuSP63
MITVARMKSCVLLLALAALVAAEAAAGLQEPMLFYHSAAGMAEAARIRQHEAALDFDGSRIT
GGQSVNAGAHPHLGGLVITLTNGQQSVCGSSMLTNNRAVTAAHCWWDGQNQARQFTVVYGS
NRLFSGGFRITTSNILMHASYNFRTLHNDVAIIVHQWVAFGTTINRIALPSGTQLNNNFAGQTAT
AAGYGRTGDNAAIGQNQDKRQVSMPVITNAACASTFGSSTVIASTLCTSGAGARSVCPGDSGG
PLSVGSGTGRILIGITSFVASAGCARGFPAGFARVTSFNSWILQRL
>OfuSP64
MKWFLLVFVAVVAVEAGPFNGRQSYHESVGIPLSESIKQRELAADSSRIVGGTVVPLGQHPFMV
GIVIYLHNSPSTSMCGASLLTHTRSLTAAHCWHDGWLSAYLFTLVFGSQTLHTGGLRIDTQDIE
MHPDWSTMTLHNDIAIVRHDWVDFNDIIRPINLPTNQANNDFAGSWAVAVGYGRQYDTQTPIF
NPDLREAHLQVIPNQECNAIYPGMIVDTTLCTRGPVGTNICLGDSGGPLALGTGSDQILIGVVSF
GAGACEGGHPAGYARVTSFLPWILSRD
>OfuSP65
MKWSWLVFVAAAAVVGANPIEGPQWYYHDAVGIPLSARLMELEKQVNYTRIVGGTASNLGA
HPFMVGIVSHFAGTSATSVCGASLLSNTRSLTAAHCWFDGWRWAQRFTMVFGSQRLHFGGIRI
DTSDIVMHPSWNVNNYQNDIAIVRHNWVTFTNIISPINLPTGQQNNNFAGTWAMAMGFGMTS
DFTPPAQNQELRQANLQVITNAQCLPRWPNIVASSLCTTAPVGVNVCVGDSGGPLVVGSGNSRI
QIGVVSFGAHSCEAGQFSVFARVTSYLSWILSTA
>OfuSP66
MQSSLLFLLVVAAAAAEQLQPNTRYHETEGIPRFLEVQRLEEGSDFDGGRIWGGQAANAGAHP
HLGGLQILLTDGRQSICGSSLISNTRSVTAAHCWRTNSMQARQFTVVWGSNRLTTGGTRVVTT
NVVVHPQYNANTLNNDVAVIRHNHVNFNNQINRIALATGSNSFAGTWAVAAGFGRTGDGLLAS
SGTKFQANLLVITNDVCRQTFGNTIVASTLCVSTAHGSSTCPGDSGGPLAVGSGNNRQLIGITSF
GTQWCARNFPAGFARVTSFAAWLSSQ
>OfuSP67
MKSAVLFLLVVAAAAAERLQPNTRYHETEGIPRMQEIQRLEEGTDFDGGRIWGGQAVGAGAHP
HLGGLVITLTTGQLSICGSSLISNTRSVTAAHCWRTNSMQARQFTVVWGSNALMSGGTRVVTT
NVVVHPQYNANNLNNDVAVIRHNSVAFNNVINRIALATGSNSFAGTWAVAAGYGRNGDGSGSS
NPGKFQANLLVITNDVCRQTFGNTIVASTLCVSTAHGSSTCPGDSGGPLAVGSGNNRQLIGITSF
GTQWCARGFPAGFARVTSFASWLSSQ
>OfuSP68
MQSPVVWFLALAVAAAVAAAGAFEAPLLYYHDSAGVARAALIRQRERALDFDGRVAGGQAVS
AGAQPHLGGLVIALADGRQSVCGSSLLTSSRAVTAAHCWWDGRNQARQFTVVYGSNRLFSGG
VRITTSNVQVHQNWIPTIVMNDVAIIVHNHVAFTNTIRPINLPTGSDSYSGQWAVAAGFGLTADN
AQITQNQDKRQVSLQVIGNDVCRGTFGPLILSSTLCTSGAGGRSVCPGDSGGPLAVGSGDSRVL
IGITSFGSTEGCTRGLPAAFARVTSFAAWIRARI
>OfuSP69
MTSGFVLVCFGIVFLNGVDMQFYRVVGGLPTTIDQYPIIAQLLLDVWGTQNFIQHCAGVILTTR
HVISTAHCFQFSPNTGLNYSQPKFWKIRVGSSYRTRGGSLYNLKTIIPHEAFDKNFYTNDIAVLV
LSKQIKLNSNVKQSTIIRPNVEVKPYSLCTLVGWGTEEVGGPQPNQLHHTAILTVDQTVCRDRY

STINAVIADSMMCAGRLDVGGIDGCFGDSGGPLIYRGIVVGLVSFGYSCGDPYFPGVYTKLSKY
TNWIVKTVAANKT
>OfuSP70
MSLCYLLLCVFFGSVFTIEPRVFSADDLRFDVHKYLVKLKVFHENHTGMCSGSILDNKWVITSA
HCFKGGEKYVSVFHQTDKGQRIIAKVDPVHIHIHPNWVVGNVSITNRVNDLALLKTTNSIKFSD
DVQSIKLSRTFPRSNQSGIIAGFGESETDLEPPREGFVTIDRCYFGIPGLLCSDNTVRAGSGDSGG
SLVSNGRLVGVTSASCKNVEEPKVCTTVYVSIAAHWNWIREMLLGK
>OfuSP71
MIAFLLIISLCGFSVHGSAIPTYDIRLQSPRIVGGLDAPDGGIPYQASLRTIFDSHFCGGSILSKNF
VLTAAHCTVGQYPAFIKVTVGTNSLVSGGQSYAVSKLIVHEGYDASLIANDVSLVKLAQDIVFS
NRVQPIELPAGDTEAGADLVLTGWGRTSYPGELPDNLQIINLKAVSVETCQSLYSGINQVFSSQI
CSLTKSGEGACHGDSGGPLVENGKVVGIVSWGMPCARGYPDVYTRVHSFKDWIVKNQQ
>OfuSP72
MKSSLLLMVCAMAAAAMVAATAGATASAPQPLQGYHEAAGVPLAARVRQHELALASGRIIGG
VPVNAGAHPYLGGLIITLVDGRTSICGASLLRPRKALTAAHCWWDGRSQARLFTVVYGSERLF
VGGTRVSTSDVEIHASFNPVTYANDIAIITHPYVEYTAYIQPIQPASGSLTYADTWATAVGYGRSS
NEVMSVENSDKRQVMLHVMTNANCRLTWATVMIGNGVLCTSSAGGMSVCAGDSGGPLVIGA
GADRTLIGIVSFGADAGCSFGFPAAHTRVTAYSAWIDARL
>OfuSP73
MRTVVVLCVIIVSTSCFTTDDVESLRREKRQEASWNWGSGGDETPSIRNSESQKLVATEPTTTTE
EPNPCLNRYKPEVKDFNRPGRRLSEVKCYEHIYDIKVREETRKSSDACLAYEIKHPGPYGPYYA
IGGVSAKPGEFPHMGAIGWKAAVGTWIFKCGGSLISDKFVLTAAHCTKASERDTSVADVVPKI
VRLGSNNIADDYLNDTLPTQANIKRIIEHPNYKAPMKYYDIALMELQNPVQFDNYVQPACLWS
GESSPLIGQKATLTGWGVVETVSRTTAPDLQAAVVDVFESDLCDKLLKPSCNRHWCGMQDHQ
LCAGKLAGGVDACQGDSGGPLQIKIPLPPTTNGRMYYIVGVTSFGIGCALPNLPGIYTRVSSFID
WVEDIVWKYVVMRSVVILFVLLVSSNCFTADDVETLRREKRQETGWNWGTDGDETPLDRNS
ESQKLSTEPTTPTTTTVEPNPCLNMYKPEVKDFNRPGRRLSEVKCYEHIYDIKVRAETKKTEDA
CVAYNIKHPGPIALGGQFAIGGKSAEPGEFPHMGAIGWKAAVGTWIFKCGGSLISDKFVLTAAH
CTKASERDTSVADVVPKIVRLGSNNIADSYANDSVPVTAIIKRIIEHPNYKAPKKYFDIALMELQ
NQVQFDNYVQPACLWSGESSPLIGRKATLTGWGVVETVSRTTAPDLQAAVVDVFESELCDKLL
KPSCNRHWCGMQDHQLCAGKLAGGVDACQGDSGGPLQIKIPLPPTTTGRMYYIVGVTSFGIG
CALPNLPGIYTRVSSFIDWVEDIVWK
>OfuSP74
MAALKSFLLLTACAAALAGRAPQAYYHEAEGIPAMARIHAQEQALRDSRISLGIQVPRGTHPY
MAGLVIHLVDGRTSMCGAAMLSHTRAATAAHCWWDGRAQARHFTVVYGADSLTVGGLRLLT
SDVEMHASFNPLTYANDIAIITHPYVEYNDYINRILVPTGTLQYVDTWAVAVGFGRTTNDPTIAQ
SFDKRQVMLQVIANLTCRNVWAASLVGNGVLCTSSAGGMSVCAGDSGGPLVIGAGADRTLIGI
ASFGTDAACGLGFPAGFTRVTSYSAWINARI
>OfuSP75
MSWKLVFVVALAVTQVPARPDVAQLAEPSYVENVRSNDGSRIVSGWEAYPGQHPHHVSLRMV
NPDGAAFSCGGSLVAKNWVISAAHCTAGRASILVRAGVVDVSHPEFTSETTEWYNYPTFVEEM
PQFVQPNDISLVKMQDSVTYTRLTQRIRIQPGVDAFRNYEGLVVIASGHGRTWTDGSTSQNLN
WVYLRTISNDACAGTFGNSLINNNAICARYFNVTSQSTCQGDSGGPLVHTDVDGVPTLIGVASF
VAGGSFGCHSGLPGGFIRPGPFHDWFTQISGLDFDNLVEEDDVTEPPPTEPPAISTEVPTTTTQAP

TTTTTQAPPKPDTEEPENETEAPESEEDTNEDSNEESGDSDSSEDDEDDDPELKDLLKRLEVLV
KVKVKMSKYGNKHKHEIKHKHNKSISHRH
>OfuSP76
MSWKVAFVIALAVGQVPARPDTIENVEPTFLENVQSDSDSRIVSGWDAMPGQFPHHVAVRMIN
PNGASLCCCGGSLVAKSWVISAAHCTAGRARLQIHGGVIDVNNPEFTSESTEWFNYPTFIDELP
NIVQPNDISLVKLKDSVRYTRLTQPIRIQPRAEVNRNYENLVLIASGHGATWTGSAISQKLQWV
YLFGVSNEACGRNFGTALITNNAICARYFNVTSQSTCQGDSGGPLIHVENGVPTLVGIASFVAG
GTFGCHSGLPAGFIRPGPFHDWLTQISGLDFENLNEDDEEALPTVPPTITTEVPPTDPPTTTTEAP
TTTTEPPTEPDTEEPEKEEESNEESNEDSDDSSDSSEEDEDDPELKDLLKKLEVLVKVKVKMSK
HGNKHKHVIHHNKTITHHH
>OfuSP77
MLGPKLHCGGAIITDMHILTAGHCITFGVHFRDLAVYVGMHDRLDNSFVTLRVTNGVKHPQFT
SNAVRDINDIAVLTLNKKLRFSDKVRPICLPSEDMDFHNLPLTVAGWGKTRQGALTSSRYLLET
KVQIVDTERCRKSSIYRDNLVPDTMMCAYSLGKDACQGDSGGPLFSTNRKTHNKKWYQVGIV
SWGIDCAMPDYPECGKPSDTVVSMRIVGGRRAAPHSHPWTVAILKNNRMHCGGAIITNKHVL
SAGHCFKWDNFKTMQVLIGLDNLDNLNNVAHRNISDVVIHEGFTSTAVRDENDIAVATLNEPV
EFGQTIVPICLPTPGQEFASRVGTIVGWGRMGVEKSSSKFLLKALLRILSDQECMESQLKQHLK
PTMMCAFSKGKDGCQGDSGGPLLVFENSGRYVQAGVVSWGIGCADPRYPGVYTKVSNYIDWI
RQQSKDAITCD
>OfuSP78
MSSPAHNSEVSQTTNMWWIFLCLFMCFTTGLNINKDGLQFTEDSYTLPSSVPVQRSLPLCRDCS
CGERNEEPRVVGGLGSSVNAFPWLARLIYHKSFGCGASLINDKYVISAAHCIKGFMWFMFRVT
FGEHDRCDTSVRPETRYVVKMYAHNFSLTDLQNDVALLKLNQPVTYSHAVRPVCLPINEAKTY
VGATAIVAGWGATGESKNWSCSLLQADLPVLSNQDCQATSYNASKIKDAMMCAGYPATAHKD
ACTGDSGGPLITENEDHAYELIGVVSWGYGCARKGYPGVYTRVTKYLDWIRDNTQDACYYIV
ETYEESASKMWKAIIVVLICFSFFVRAENVTTNIDNDVKIEEKRTCNCRCGERNEASRIVGGVE
TAVNEFPWAVRLSYFKKFYCGGMLINDRYVLTAAHCVKSFNWFMIKVTLGEHDRCNATRRPVT
RFVVEMVVHNFTYSNFKDDIALLKLNEPVNVTDTVKPICLPKDDGQTYAGVKAIATGWGSLTE
EKNHSCKLMEVEVPVLSNDECKKVKYMPNMIADTMLCAGYLKEGGKDTCQGDSGGPLCAER
KDNKYELLGVVSWGVGCGRPGYPGVYTRVNKYLDWIRENARQGCYCSD
>OfuSP79
MCLWIVPIITVVLFNSADTREISSPDGATLDNHVSSGNRSQRFLFDAIFGLEVPILEEQSIEDDDD
DPQVQKCSCECGRANPLPRKMECGGSNQENRIVGGMPAGANRYPWMARIVYDGQFHCGASL
LTKEYVLTAAHCVRKLKRSKIRVILGDHDQTITTESAAIMRAVTAIVRHRSFDADSYNNDIALLK
LRKPVNFSKIIKPVCLPPANVEPSGKEGIVVGWGRTSEGGQLPAIVQEVKVPILSLTQCRAMKYR
ASRITNNMLCAGRASTDSCQGDSGGPLLIQTGDKFQIVGIVSWGVGCGRPGYPGVYTRITRYLP
WLRANLRDSCLCIFEFRIFISILFINIDKMWKCIVLVLAVTTFTYSEGDIVRDTRGVFTKNIFGGV
WGNRPPLMEANLAKTTCTCKCGERNDAPRIVGGQDAGPHEFPWMARLTYFKRFYCGGMLIN
DRYVLTAAHCVKGFMWFMIKVTFGEHDRCNSTTRPETRFVLRAISNKFSLSNFDNDIALLRLNE
KVQMSGAIKPICLPSNDRSLYVGVKAIASGWGTLTEEGKVSCTLQEVEVPVISNQECRSTKYTA
SMITDHMMCAGYPGTGLKDSCQGDSGGPLITERKSDKRYELIGVVSWGNGCARPGYPGVYTR
VTHYLDWIRDNTRDACYCTD
>OfuSP80
MKFSLLLLLTIVSAVLCTSQGPDTRIVGGYPATIYNYPFVVSIVYTNPETGEQTPRCVGSIISSWH

VLTTAHCFSDPNVENYKLRVGSTSSIEGGTLFDIYYIIIHPDYVESPRTADAAIVVLTQHVTISESV
RVIYIPPQETYIPDGYTVRAIGWGAEEEDGALLDTLKVFDTRTIPLQQCIEAFADDDNINVYDQV
ICTQAAGRSMCRGDSGAPIIIGEVLVGIASYSSSCDDSTPDTFTRIDRHTGWILQEAVPPYGRSAA
SPVRVAPVVSWSATRTIIIIGEVLVGIASYSSSCDDSTPDAFTRIDRHTGWILQEAVPPYSRSAASP
SNWIITNMAFKVLCALALLSAASAAPGSRLTGGSPLPVEEFPSVVAITYYYPRPQITVQRCVGSL
VSSIHVMTAASCFDGADLDNMRVRAGSAQPLSGGTDVGVTHFIAHPDFEEYPRTADIAVLFLEQ
FLPITSTVNILYLPPPGYDVPDGISLRVASWGFESLDGGPLNDLKTALLPKIPLAQCQQAFADSEE
ASITPSVVCTFAPGQGICHGDVGAPAVISNVLVGIASYYENCDGTHPDVWTRVDSYTTWIMQVT
AGASRMLTV
>OfuSP81
MAILFVVIVNATSHPKFRVIQGVDDEDNKYPYVVSLEGLQSKVPGLEFYRMCTGTLIAPEWVLT
AGHCLLPQLEIVRYGNMSIPRNATESTRRILKRIPHPNYKGALFIKSIAIIKNDIGLVLVEEIQMMV
LGKLSAVDYRTLNGEKVTYAGYGGTGETLDVYKPLQLGESMVRSCLQSNIPDPRIPFDIRDWG
PTLCAVPKCTLKDHAPGAGDSGGPVFLNDKVVAVISGPF
>OfuSP82
MLCALLLITWLKSTHSIGEKAHQACRNYGQSMLECHNVLFPMSRVRVPIDFLLYPHMVLIGFR
HNINDIPAWKCGGTLISEKWVLTAAHCREDPNSGKASIMRVGTATFEFDEIDEMAQERDVAEIIC
HPEYKPPSKYNDIALMRAEPNFILSRDIRIACLETRNDVKYKRLTAIGFGVTMSGGRSGSQTLM
KVDVDIVDNVTCNMTMRFMIKRKILAQGIVEDQLCAGDYENGGRDTCQGDSGGPLQVMEDR
ADCVKTFPLHKVVGVTSFGRDCGRKMAPGVYTRVSRYIEWIEQIAWP
>OfuSP83
MKLFVLLCLCEAVAGSVLDLTKNDHKCGVEASTNPLHHEPWLVHLEYYRRGARADIRCGGTLI
SKRHILTAAHCVNKGVGASKPTSLVARLGEYDLSTAVDCADGVCAHLPVKINVAAIEVHPLYDD
RDHDVAILTLERDAPYTDTIRPVCLPSGKLPENAVLTASGWGEDFTKGVYSNVKKNLHLPYWS
TSRCKSAYKHLLLPEPIICAGGEKDVDTCRGDSGGPLTWGVNRKELWGVTSTGNTICGTKGYP
GIYTSVVHHLEWIEKIINK
>OfuSP84
MLSVLCACVVVSVGCASAQDLAAPSETKLYTGSLSPECVSSYHQIFVTRRVPPRNYQYHHEVA
DLETKAVPFKWRIVGGTEVDITNIPYQVMYGLYCGGSLIAPIWVLTAAHCRDKEKFVLAGATR
RSQATRYKVCAHFIHPRWDDENKIHPQDFDYQLVLLETPVPVTPSSRPIAIGNVEEVTPGAVVSV
SGWGYTRAKERHMQEILRRVYVPIMSHDQCVSLPNQNYRTISPRMFCAGFINGTKDSCQGDSG
GPAVHNGKLIGLVSFGVGCAQKDQPGVYSNVPQVRDWIRSVTTLPL
>OfuSP85
MNARQLIQRIAKPRFSSVCVVRVVIFKSNMWISLAFALVLTIGTSSAQDANCDFVSNVQAGQTY
YVYSPNYPQNYRPGVQCRWVGICPSGYNCRLDCNDISLPQTSGCSLDRLLISKSGDPQLTSADY
YCGTGTVTAVSTGQRISVGLITSTQSPGGRFMCQLTAQAATTNPTCSCGYKKTNRIVGGQQTGV
NEFPMMAGLAYRDIGQIKCGAVIISKRYVMTAAHCLTGQSLSNLAIIVGEHDVTVGDSPATQGF
QVISAIIHPNYTPSNYDYDIAILKTNADITFSDRVGPVCLPFKFVNTDFTGSKLTILGWGTQFIGG
PTSNYLQKVDVDVISQSSCRNVVPTLTARQICTYTPGKDACQDDSGGPLLYTDSSNGLLYSIGIV
SNGRFCAGANQPGVNTRVPALLSWIQTNTPDASYCYK
>OfuSP86
MIRHITCLLIVALAVPSYEQDVPISPCPNVFMYEPLGAEKNRWYGVVNLSTDSTLHGLWLNIVL
DGKADILGNWIGDVSTSDNKDFKVENSDMMIHPGPAVAVRFFVQYNILNPTPKLKTIRLNGREI
CNADIRSPVTPDTNWATPKPTKAPTRPVPDRTTERVVTQRPITERPVTDWAQEHPVYRPPSGGS

PNPVDIAGGHDTSNGIKIVDWPPRRPGTTTEADDSQEKGGGGFGGGLPVVFVPGRPTSRPPMF
DEGNNNKPQVQCGSVVKQNPKILNPLIVNGVPTYEGQWPWQVALYQTQVTVDNKYICGGTLV
SERHIVTAAHCVTQKGSNRLTDKNTLTVYLGKHNLRTSVEGVQIRLVSHIHKYPEYNGSSYQM
DLAILVLREPVFFTDWVRPVCLWPDSDTSLNTIVGKRGSVVGWGFDETGVATEELSLVEMPVV
KTETCIRSYSEFFVQFTSEYTYCAGYRNGTYDERTGRITSTSVCNGDSGGGMVFKINGLWYLR
GLVSLSVARQNEYRCDPSHYVVFTDIAKFLPWIKARLSE

>OfuSP87

MQYFRNTDTCEPASPRRPDMKIGKIAYHKCVDYQEQIKFPCVGYTYYAQFNGSTQTRYTRRDG
CSDPPSLVIGSSGVDASRGQFPHMALLGYVKDGSKVEWLCGGTIISEQFILTAGHCTSHKQLGP
VSLARLGILKRSDPDKVARDYKIKRFLLHPEYKPPAKYNDIAVLETEKRIVFLNKHIVPACLDAG
DADVESDAEMASATGWGALGDGEPNADVLQRMDIVKFSTEECSALYKPYRLLKTGLDNSTQI
CYGHRQIPRDTCKGDSGGPLQVEYREVNCMHKVIGVTSFGRACGNTGQPGVYTRVRPYVPWI
ESVVWPAQGTDKCVCF

>OfuSP88

MLAYVGFLLLQMLFSMLVPAITNTLSFIQERSRELDDKAQVPRIAYGAPADITEHPYFAILEGCG
AAIISEEWLVTAAHCVLPHGYGHYEQHVTFVGSHTLENSVPVFIEEIVVHPQYTDLGFAIVNDIA
LIRLVKPLKFSDRIQPLELPDEKYELEDDSKHVFVGVGLDETGKPSKQLMKIDVNGYSTSTCYA
RYFGSMYWSVAPFMSWLDETTICARRVENLVGAGYGDSGSPMVKDNVLVGIASYIMLDNDCH
SCHSRTLYLANVAHYVPWILSHTGKL

>OfuSP89

MRLVFVIVALVSSVVGSVEVFGPRGYHEEVGIPLAKELKAAEEHNVASIARLANEFEDRVVGGV
AAPIAAHPYLGGLVISFFNIAGNSVCGSSLVSATRLVTAAHCWFDGVNQANLFTVVLGTNFIWS
GGLRISTSNVFMHPQWTPSNLSNDIAVIYLPWAISFSNVIQPIALPSSWDLHQTFEGQWAVAAGF
GKTTDSQVGPSAQINQVNLQVISEQACRNVYGANFVFPSTLCTSGLGFVGVCSGDSGGPLFVT
RNGQRTLIGVSSFVASNNCQGGHPSAFARVTSFVNFIQQHMW

>OfuSP90

MYFGTVIFVAIVASASALLEVTPSRGYHEEVGIPLAASIRKAEDEALAKASEADNNIHRIVGGVL
APANEHPYLAGLLIDLVNTAGQSVCGSSLLSTNRLVTAAHCWYDGVGQAWRFTVILGTQFLFW
GGRRISTSEVFMHPQWNPSNLSNDVAMIYLPVDITFSNNIQPIPLPDATQLRNTYTGQWAVAAG
FGRTSDSQVGASSVISHVSLQVISETQCRVVFGEGFVFPSTLCTSGIGGVGLCGGDSGGPLVVTQ
DGQKHLIGISSFVAANNCQGGHPSAFARVTSFMNFIQQNLW

>OfuSP91

MRLILVIVVALVASASAFVEVLGPRGYHEEIGIPLATALKEAEEQMIASMARQNFDNRVVGGVI
APANSHPYLGGLLINFVNIAGTSVCGSSLLSSTRLVTAAHCWFDGRNQASMFTVVLGTQMLWA
GGQRITTSNVIMHPQWNPWNLSNDVAMIYLPFGVTFTTTIQPIALPNNWELSQTFVGEWAVAA
GYGKTSDSQVGASAVVSHVSLQVITVQACRNVFGSTFVFDSTLCTSGAGFVGVCGGDSGGPLF
VERSGQKLLIGISSFVAANNCQGGHPSAFARVTSFINFIRQHIHLTKYPPVLKIVNIMASVVGSVE
VFGPRGYHAEVGIPLAKELKAAEEQRIASMARLAGTMDEKVVGGVAAPLHAHPYLGGLLIHF
WNTLDQSVCGSSLVSANRLVTAAHCWTDGFITGRMLTVVLGSEELWDGGLRISTSVVVTHPQ
WNPWNYSNDVAVIYLPWSISFSNVIQPISLPSDWELHLTFEHEWAIAAGYGKTSDSQVGASNVV
HHVNLWVITEAACRHVYGEEYIFSSTLCTSGFGFVGVCGGDSGGPLFVQRNGQRVLIGISSFVA
KDNCQGGHPSAYARVTSFIPFIRQHMCTIVVTVPIMRLVFVIVALVSSVVGSVEVFGPRGYHEEV
GIPLAKELKAAEEHNVASIARLANEFEDRVVGGVAAPIAAHPYLGGLVISFFNIAGNSVCGSSLV
SATRLVTAAHCWFDGVNQANLFTVVLGTNFIWSGGLRISTSNVFMHPQWTPSNLSNDIAVIYLP

WAISFSNVIQPIALPSSWDLHQTFEGQWAVAAGFGKTTDSQVGPSAQINQVNLQVISEQACRNV
YGANFVFPSTLCTSGLGFVGVCSGDSGGPLFVTRNGQRTLHISTTIFSGDTLKMLGKIIFLILPVV
LSATKFCADESCGSSSQRIGAGVITERNSRPFQVALYSRVGTTGELGFCGGALIDQQWVLTAAH
CCFHGGQQVDNVQAILGGHSLYDRYENGRRVVNVDSITIHPDWDPDTFAHDLALLHLANTVQI
TDMIDVVRLPYGTMIAANFAGQGATASGWGIAAEGVTFISPTLRQFFMTVMTDTLCNSLYANM
LPPNTVCGFSVTSGTCKGDNGGPLTIFNNATEETILIGITSFIDLSGCNVGVPSVFTRVQMHLNWI
NEVTGVPL
>OfuSP92
MLGYIVLVLAVAGAPAFAGDQLTFPEVARGAARIVSGWEAQEGQIPHQISLRMVNPGGGVSSC
GASLIHHEWALTAAHCTAARVSLVLRFGTVNLTRPALIQESTEYYNHPSYNEAFPGVVQPNDIG
VIKLRRPVEYTDLIKPVRIQRSADKDKSYDRVQLTASGWGLLWTQATSPENLNWVYLLGVENS
YCRVRYGFSSIIQDSTICASAYNVSSQSTCQGDSGGPLTVVDVDGQLSVVGVTSFVSATGCHTD
FPAGFIRAGYYHDWYYQLTGINFDWDPEEAGSGEGSSEGSNENSNEDSNESSNESSNENSNESS
NENSNESSNENSSESSNGSSESTSEEDK
>OfuSP93
MDLVPDDPIQVSTLNSNLTLMARVSNGECNHFRVPTYSPWGRWGACRLGRRVRRRHCVRREI
CGDSVRIEVASCRKRKSRMKILTHGITNGFYNRAPHMEKYQRRQLDSNEIQKRFRGFSPWGPW
SSCSRKCTTVRRRYCQKRIFCGRKVIRQSAYCYVEGSYCQHWIRTRMQRRKDPGVGYRIVESM
SPPAPAIHPEDSRYLHHGPLECGRLGHYRGSAAARMRARMKDMIRIIGGRPAPPGKWPWQVVV
LNRYKEAFCGGTLVSLRWVVTAAHCVRKKLYVRLGEHDLLTRGPGEMELRVTEAVVHPHYDP
DTVVNDVALLRLPMPARPDLGHGIACLPPAYQLLPPHTTCVILGWGKKRATDVHGTRLLHEAQ
VSTIQQGVCRRSYWQYAITDNMVCAGRGRKDSCAGDSGGPLLCRDRDMRYYLQGITSFGDGC
GKRGKYGIYTRTAGYVGWMQDIMHNRYFD
>OfuSP94
MKLLLLLLCAAAYVSCHSLESPLKSQDGAQDKGFIDWITNLLGGPSTTLRPVQDPPDDCPVCQC
GIARTRRRIVGGYETKKLEFPWMAVLMYNGRFYCGGSLINDLYVLTAAHCTAGFRKEKITVRF
LEHDRSVANETKIVDRSVAQIIRHLRYNPSTYDNDIALLKLSNRVDLSSALKKRVRKDETGSGS
GSGSSEEESDDVGLRPVCLPTAGLSYSNYSGVVAGWGTTEEGGSVSNTLQEVYVPIISNADCRK
TAYKQRITENMLCAGEPDGGRDACQGDSGGPLHIVNTTNAQFQEVGVVSWGEGCARPDRPGV
YTRVNRYMTWIKSNTRDACYCQ
>OfuSP95
MFRALFILAVVAVARACKPCTCGVARGARVVGGVAVTPGELPWLAALVRDGKVICGATVVARD
HLITATHCVHEVEASRLSVLVGVYDVNNTHHSPGYEVSHVTQHPDFNRYNYDNDIAVLRLAEP
LPDNLFRPACLPDDEEVLAGADAIVSGWGSTVEKGPASNIPMKADVQIWTQEDCAGAGYGRG
KVTPRMLCANAPERDACTGDSGGPLLVSQPYYTIVGIVSWGRGCARQGYPGVYARVDRFLPW
LRVALRHACTCTSPF
>OfuSP96
MNVLGVVFVLVSVGHASSGYKFGIGKDELDTSSIILSERVPVEPSGPGLADEYPRPQGSDRIVG
GQETTIEEHPYQVSFIVNNSYFCGGFIVSEDYILTAGHCAQNVDPSTVVLRAGSTWRRNGTVIPI
ASVTPHPNYDEPAFDKDVAVMKTAEKINFTEAIQPIPLAPLGRPMRGGSDIVVSGWGRTQFGAA
TIPERLMDVQIPVVSHLQCRLVYFSVLTDNEFCAGNFFLGGKGTCQGDSGGAAIQDGMAVGIV
SYGRGCGQALSPSVFADIASKPIRDFIKEQTGL
>OfuSP97
MYLLVSVIVVGLISSGDGQSPCPGTFEYINDELGIHGLIQIQPNGPVSAITTRVNFTIAARLPSNY

VGQIKPVDEEHSLQRFNQGAPLTYRVHFPVTSPLPKLTSLVVNNQELCSGPPDFSQPGQYISTISL
QHMLYLREGSPNSVLYPYEPTIPEKPIYNQPVYSKPVYEQPVYNQPVNKQPSFGRPDVTGIFNG
PITITGVPTNTAGAPTQKFTLQGSNGAYYTLVVNKTQGSSQPGGGQNFQVFLETTTTSRYPQTP
PAQFYPGFPENPTRFDQPPPDFNPYETERPPVRPIKTTPPPPPKQETPLTPPPDYNPYETTKRPVY
RPSKPDQLDGQNAKPSLIPDSNNECGVIAGGNEHQGLIYNGQAYDRGQWPWLVALFKQRATTL
SFICAGTLISPKHVVTAAHCMQQKNTKLATQDIVVKVGAYNVKDWGDDISQTRTLVSAVIHER
YNASTLANDILMLTLDRSVEFNTNIRAACLWTGKTDQNRIIGEAGVVAGWGNSELGLAGEALP
RMARVPIVSTAMCRASNADFHKLTSEYTFCAGDRNGVGPCLGDSGGGLYIIEDGRWRVRGIVS
LSLRQENGDDTCNLNNYIVFTDAAKYDKWIRNIVQQSSLF
>OfuSP98
MNQPGEGQPQAAPQGSEHQLPADQGCDDQPLAGPSCADQFSNVGEVMLRAEEAVADGEQAA
VEDAARSGTASAKVSAALRVIHGTPDVNNEFPYVVSLETYRQLNINWKIYRVCSGSLVTANWV
LSAAHCLVPAVQVIRYGDMTVERNATDSINQVIKMFPHPNYKIMLTPPVMLVNDIGLALVEKVP
VSPGKLSAVDYKGLIGFEVRYAGFGLTHEKEHMYNKQNMEKDNMKPLQVGMGVVIICPTIDY
TWNPGVCVAPKCSNKHHQPLPGDSGGPLFFDGKIVAVASGDFGSSITIHTPVSPYLIWINDVITK
ESHHVRINS
>OfuSP99
MRVIWILSLLFVVACSASEDGSNAHYQEGYHQSIGIKDAASRKRYEDQIMALGPIVGGVIAPVA
AHPYLVGILIDVIGMPSQSACGGSLISASRILTAAHCWNDGRFQAWRFTAVLGSPFLFHGGLRLF
PHGIAVHPNYDKRTLANDIAVLYLPGNVPLSSSIQPIRLPHGALLNHDLTGAWSVASGYGRYSD
AVSPTTNTMARFVSLQVISLDQCRGVFGNAVLDSNICTNGYGGVGICRGDSGGPLTVTYHGEKI
LVGVSSFVARYGCELGFPSVFARVTSFMDWIQRHM
>OfuSP100
MQTISIDVLLLLVSAVGTRNWPTPRVRDGQDDVLNEFPYVVSLQSAYFGRNDEDQEQYISTLLR
GCTGTLIGPNWVLTAAHCLDPYLSFVRFGNMTIPPSATSLRKILKMISHPSFKQVENFPDYSCQN
DIALILIESLHMPSVGILSAVDYKTLVGRKVRYAGFGATVSSSDYDNRLDDEFRPLQLGEGVVVS
CGHNLLEWRPAVCVAPKCSNARHDTMPGDSGGPLFHDEKIVAVVSGGESEPEGIYTPVSPYLT
WIKTVMTENRS
>OfuSP101
MKWCLLVCLVAVVVEAVPFEGPRWSYHEKVGIPMSEQLKKFELAANSSRVVGGTSVPLGRHPF
MVGVVIHLFTPFTSMCGASLLTHTRSLTAAHCWYDGWRWARMFTMVFGSQTLHIGGLRIDTE
DVEMHPDWNTVNINNDIAIVRHDWVAFNDITRPINLPIDQANNDFSGSWVVGVGYGRQYDGQ
VPIFNPDLREAHLQVIPNEECRQAFPNFIIDSTLCTTGPIGINVCQGDSGGPLALGTGDDRILIGV
VSFGTFSCEGGHPSAYARVTSFLPWILSRA
>OfuSP102
MHTMDTMGYGHKKYRPPISPTNFYQHQHDYYSRHTTLRPQSEYRFAGGGYPRASSGGGRSSG
FCSAALVGGALLAAFAVLAVAALAFYMGALRPDNGEPMMTFEGSFRVTRGDVYGGAPDSPAW
RERARRYGAALRQVYAGPSPLRHAFAGALVTGFGDRRLDVHFRLYLDRRKIPSTVSNIEETLKN
VLVQDLLSKHPAFGQTIKVDTTSIVIKRDLEHTYHSESYVKEAMNESMATSYPKSLSPQNSKDK
ALQSRIGVVRKTTVKPNQSNKGEVEEPEIDIENIPVVQGTFQITKTEADITENKKNSSPVRGGTH
KPVPVAKPSPTTRAPTTRPPTTKPTTNRMTTKRISTTTPKPRPFTITSTLNVKTKTDATPKRDFDN
NFKRSTTTSSTTTSTSTTAPLTTTVETSTNNVSQILLDLLTNENHYKELPKIDSLFTVPHVIDNEP
WKPITRPYYETTSKTAPPSEEVIDQNVEDRIGVAEVVDDVSVLESMLTPIPPVKNRDRTTRRPGV
YVDPDLAADVYVPSPVYTSFTLPTFAPPVNGMETLGSGYVKPHPIPVDKISSVVEVPAEISPDDE

DDGKPVQRPPKDKTTSIVLNVAQSDKQDTNFEKIAFDGASIMKKPNATTTSTTSTTSTTTSTSTS
TTTTSSTTTSSSPPSTIAESDSFEKSANNTELLEELNKNFTKEISTKRPNNKVSIIPTTSGPLHTWE
LVNTSTNDNDTFNKNSPEKYYNDTLQAIITKNDAVFPNTTPRFQNKVSILRNLTEIIKRYSQNST
EKPIKETESTERIEDRQGHNKMTGSVEVVLDDEIQITTASVITLLPAKSNLGVNRPLRPRPKIKAE
TQPSKESIRSFFRNDPETQKIIKELTTENYVQVVQVSEETSKEPEIVTSSLVFETDGNDSDDSMAL
ATVSDNIKVNSENIPSKNRLPKSSDDLKKATDEVPDDGKLETNIPQGTYRVSYHVTGSVSSKQA
NKTQSLPAYELALEPDVVLEIPYNQTNSLTIDKLKQLANLATISDSNNNTLFRSPGGVISTKAIPS
SYTLNQAGFKILTKTYNKLQPGTKQDDNSLDKPEKTYSKPYNKPLKKEEKEIVKDIVKEEECSN
VTSFRCASGACLPLTSRCNRLIDCPGGEDERACTCADYLRAEFSQSKICDGVVDCWDYTDENK
CEWCEDGQYVCANAKQCIEMYRVCDGTPDCPLGDDEKSCVALADELDNNEVVPYNEEGFVM
VRKRGVWGRLCVESFTEAVQQAHSPLKLPDLGKAVCRALTYHDSPWSREAREGRKASSVGY
WEVWHNANARSSDTRLTFRRASCPRRRALRVRCQDLDCGIRPHADAQQPRVVTERVARVRWS
RVVGGAGAAAGAWPWQAALYRDGDFQCGATLISPQWLISASHCFYQATEAHWVARLGALRR
GAWPRGPWERVARVRQVLLHPRYAPRGFRNDIAMLRIDPIALHARLRPACLPPSRAQPPAGHHC
TVVGWGQLYEHERVFPDTLQEVELPVISTAECRRRTRLLPLYRVTEDMFCAGYERGGRDACLG
DSGGPLMCQEQDRWYIYGVTSNGYGCARANRPGVYTKVSNYIEWIDSVIAAHTPQPHHNNTQ
SDEYESDDEFYADLEAAENKRATRIDTCKGYRCPLGECLPPSSVCNGFIECSDGSDEWQCSKRN
SSIHDPD

>OfuSP103

MTYYAGIRVILVRCEKLKMIFVIFMLINTVCGEVYLNTVNHMEYYRNRTRPSKAFSSFSTNYFN
QNSIKNPDKTEKVQCGTRTVDFNPRRTGKIVGGTETPYGAFPWQVEIQMLNVDNLNFEHHCG
GAVIAERLVLSAAHCFDKQPLQLDHIRLVVGEHRLKFQDKHENRFLAEKVVPHPDFRKNGPHS
NDIALVVVSRAGSGVQFNTHVRPICLPEERDASAGRWCAVSGWGYQAESTESFAPVLRAASVP
VLDLATCRKNQVLGGRQQAILDSMICAGVLSGGVDACRGDSGGPLACKPSNRWQLHGVVSW
GSGCARRARPGVYTRVASYIRWIKSTAAALGHKIAA

>OfuSP104

MKRKKRNVEDIDDTDRLLDKLSDEEYKKARDIIMNHKTLCNTENKNEICKDLVGKLKAITEGN
KASDRAEQGSKMHPVVVPENKGQAKQSFRANNIPIDMTKREVFPLPLTDELTGLSNRDAIGYG
AEPYQHVPSYSLPYSHQPQLLDPSCLLARLLKKGHPLGGYESHVPEYAPNTGYTQYPTYHGISN
QYRESEDHEDQPIRQIHPQDEAIFAEFLKQYAYLDEQKRLQEKNSTAPREAQCPEGTISCFNGEG
CIPESQWCNGHVDCSDVSDEAKCSCKSRVDKSRLCDGYFDCPFGEDEMGCYGCSENAFSCED
LDINSKSSCFSKEQRCNNIMDCPNNRDEIDCNMLSPSLHNNPLFATSNTEGFLQRNFKGEWYAV
CKNPYMWAHDACRRETGLIIRPPYIQVLPVDPLLRLGYLNTGPEGMIHTSNTCFNSSAIYVTCP
ELLCGTRISSTSQLMKENSAIENHLFGRNKRFLLNGRPYPMSFYDPLKRNIRNNALITEQKESKK
VTSKTKRAQARVVGGRPSKPAAWPWMAALYRNGMFHCGGVIVTQHWVLSAAHCVHEFWSH
YYEVQVGMLRRFSFSPQEQNHRITHVIVNQNYDQADMKNDLSLLRVKSSIQFSRWVRPICLPG
PNTAGPEWRWGPAPGTVCTAVGWGATVEHGPDPDHMREVELPIWAKCKHSEDRDGKEICAGP
VEGGKDACQGDSGGPLVCRSPLNSQQWYVAGIVSHGDGCARKGEPGVYTRVSLFVKWIRYHI
GSKILPTIQPQQKCPGFKCTSGLSKCLPDKRVCDKTVDCLDGEDEMNCNSMRSFDNIFLARTSS
DNTNANPSTARESNNNDSVKNFQSSESSDKEQWLGNEETAKKQLNDDMLHASTIEISIPNSSEH
MRDNSEQDIDPPSFGRESDFIAVSSQNSHPLESRSSLLEKENINDDEAVNMQSRASDIQEDWATT
PPSESSTIMSSMSVKTNSPITEQTTSTMPITTPLPVLSTTSSMLETTSILDSEQSKRKINNREDDES
ESKSLLVELLPHSENVDDESEKITGESNVIEKKEISPSAKIIDDFSSKKISTFDDKLDIIHKIEDLVL
SELQPAKIRKKHLTPKEFECRRIYQTIPYSHRCDHKADCEDGTDELSCTCADYLHAFDDTLLCD

GIFDCADGQDEADCFSCPEDRFLCRKSQICLPMKHVCDGKPQCPQGEDEVDCFALSNGKELKY
DIDERPKTRLEGYLTKKQKNKWQIICEDNLSTEQQEQAAAHICHYLGFSSANRYLLKHLNIDDS
ILKSNEFRKRRDTSFSAPVHFTYREASSSEDNARHIVIKEPQLLKEKCLPNIKKTCMSLYVYCDH
SLFTDFDFSPNLLYSRAVETTTNEMWPWVAKVYVEGNYKCTGVLVDLQLVLVSHSCLWDSSE
KNLICFLLFSMSRHHITVVLGSHRTLNATHGPYEQILPVDGKRDMYRSNVALLHLKEPAQYSTM
VKPMVVQSTVMPENRNSTCVAVGQDGKNKTISVFVEETRENCHHHNKCFKLRSKSKSLCLPG
MVSSQQWAGIISCHSERGWYPAATFVDIRGECGLQDRIIGTGIEDLKIEIQNAGINGAIIKKQDLA
DKCHGIRCGRGKCIDLLHVCNGVRDCEDGGDESEESCKTKHLFCEKDPYDKNCECSTGQFRC
HNGVCISKEQFNDGKDDCGDGTDEPEQLSCAKYLARVMPSRLCDGVIHCKDRSDEDPMFCKC
FAKHTYQCGRTADVEHCVAPDVVCDGINDCPNGEDESTCVGLYAPQGTGYGKGQVIVRSHGV
WHTKCYPNKKHSKSELEAICRELGFISGHAKELEISNKFKPHPYNNVLVDPFSDVLLNNRTQVK
LRNSHAPLARAIFDDQLNNCHPVFIECM
>OfuSP105
MTLLTAVCSLLLLAGACYASDRIVGGSPTTIEQYPWMLQVEGQSLYNGAWVFMCGGNILNQAF
ILSAAHCFDGSFYAPEWRRMRAGSTYIETGGVIAYVDKEFNHPSYGLNAYDGDITVVRLKNFLS
LTPSIQQTTLIYQGAVIPDNVPVYHAGWGAIGYYQPESEVLLDVQVYTINNDLCRERYETLDEP
WYVVTENMICAGILDVGGRDACQGDSGGPLYFQNILIGVVSWGHLCANETFPGVSVAVASYTN
WIVETAVL
>OfuSPH1
MRGISLVLLLLACSALGKTVPPDNGVYDYHLKVGMHEADRIRKAENEQRVAGGSTTTVAAVP
YQAGLIITFRLIQTSVCGGTIISDTRILTGAHCNNDGNNIANSITVVVGSNFLFAGGTRVVYTGVT
MHPGYNPWILANDVAVVFIPRISFSLFIQPINLPTGSELYSNYVGTTGLASGFGITRDGDSVGLTQ
MISSVNLRVIPNDECASTYGGVIQPSHMCTSGAGGVGTCLGDTGGPLVATVNNRRVLIGISSFTP
RDGCQRNLPSGFSRVTTFASWIREQ
>OfuSPH2
MKLSILFIGFFISLCKGDGRIAGGQPTSISQYPFVVPLLTNSGAGNVFTHACGGTILTNNAVLSSA
SCFFTGTVADPVSQWRVRVGSSFRNGGGVIFVVNRITPNPNFNTGTLNNDIAVIRTRFNMNLQP
GVAEAATIAGAGYTFVQNAVVYGVGWGKTSPTGDWSDEIRHVEIFVVNQQTCAGRYSELGFS
VTNNMVCAGWLDVGVRGQCEGDNGGPLVDNGMVVGVFSWTRSCADYWYPAINTRVGSYT
GWIVATAIAS
>OfuSPH3
MVLLIITVLTQTASSFRYMPREDAYRAPSKPALHKHIARIQDIPHHALIVYGTYFCSGSLIRSRIV
VTTASCFNKRSRQNTVVKVGASTVTGMGQVIPVKEIKIHEYYKHDSAANNDIALLALKDVAVF
GDDVKKISLIDPEVALRVGTTIEVTGWGHSNLPQRFLNNLVWSEMVLIDTNVCVKHYGGLISAS
NFCAKYQIDRRLSDNGGPAVYRDVLIGMLSFGGTNIEEPHIAVFTNASYFNRWIMLNSRRLVEK
YFVYRDSGGSKINH
>OfuSPH4
MRCIVVLVILGTVAVSGQADLRIQGGSATSIATYPYTAALLLRSPTAANFRLTCAGVIVNTRSILT
AASCVAGDSTSRWRVRVGSSSSISGGRLLTINLIRVHSSYRAATRDFDIAMLRTTTSFVQTHLIRA
ARFASSGYTLVAPQTVTAVGWGATVSGGAPVSTIRHVRLNIINQTTCANNYRGRGITVTANMV
CAGWIGGRGQCTGDTGGPLVHNGVVVGIFSRSIGCGQANLPGINTRISRFAGWITMNRF
>OfuSPH5
MRVSSVLSLFIVGLAAVSAAPSSRIIGGSAATIAAYPEMASLLYSATNSGHRQLCGGTILNRRVIL
TAAYCTLGSGINRWRTRVGSALANSGGTVVNTQQIIAHPQYNPFTRDNDISLIRTSANLPSNNN

VIPGSISGPNYVLPDNSPVIAVGWGRINQIGQVTEQLQQVQMWTVNLNVCRQRYAMANLAVT
DNMLCAGWLDFGGRDQCYGDEGGPVFHGRVVVGITSWRIGCGSPQFPGVNTRVSNYANWIA
SNA
>OfuSPH6
MSVTSVLSLFVVGLAAVSAAPSTRIIGGSPAAIGTFPEMASLLYSDSNSGHRHRCGGTILRRRVIL
TAAYCTLGSGINRWRTRVGSALANSGGTVVNTQQIIVHPQYNPFTRDNDISLIRTSANLPSNNN
VMDASISGPNYVLPDNSPVIAVGWGRISQTGQVTEQLQQVQMWTVNLNVCRQRYAMANLAIT
DNMLCAGWLDVGGRDQCYGDEGGPVFHGRVVVGITSWRLGCGAPQFPGVNTRVSNYTAWIT
SNA
>OfuSPH7
MKGFALVLLCAVAASQARSTGVEIGREAASLGENPWLVHLRLAVTNSGGLLNSCIGSLVDPRW
VLTSASCVNSVNFVWIRYGAVDVFTPELVTEGGAAAGHLVQRHPQYDPVTGANDIALIRVNRY
VLNPPSANIAVVELAAADAEVPSSGKVCTYGADADGAAGEFLSCVDADVSTDDDGVISADAE
STIFDLGSPLVKDGVQYGILTRAGDSSSFINPAAYRDWIEQTAEIQLPGGDNNGDDSDGNGDDS
DDNGDDSDDNGDDSDDNGDDSDDNGDDSNDSDDSEDSEGLKINFVN
>OfuSPH8
MKPANNSLRNAPKPANGSPRNVPKPTNVTGHAKSQVVRLRILAIDVATTIPPKVQDILKNTNYT
DVKQLLNNSRRIIVGSEVHHNRPYMVYLKLPANNPKYKDYRRWLCGGVIVHEEYILTSAACIE
DAKHFYVVSGTYRHNERDDRYSNACIKNGAKKAIWKCIPRNYVFDGHENDNIRWMNNDIAV
VKVEDEFDFTRRVRGCDFIPRPICYNNESSKLENPGTVASIAGWGSTEKYNDWSSPQQDLLDTT
VRIISKNRCKRRWGRRYHNIIDNYMICSKDLVPDLSEICAEKYVDCTDIMYSEESGEDARRVLA
PTNLVLHSAHNDSSRRQAGPGSGGFCENDHGGPLVYGDGVNSVVIGIISACLVKERTNKCYGPF
LYTSVYKNRVLINCAIYKDVAQDCNKLFRSSITHTEVEMSWVDHPDGPSKNEIAKMRRKQSEG
NSTNEGHLQKVALDKAEQHEGVIVRAFTGQPEETSTKANTTNV
>OfuSPH9
MIRLVTFSVLFFAGASLASQRISGGNPTDVENVPWIAQVDFHLHYTGSWYQLCAASILNSRFVL
SSAQCFSGYFYAPEYRRIRAGATYRNAGGVLVNVERAYTHPSFDLYQAGETRTPDGDIAVVKLS
DVLYLSPTIQQSLIIYQDAVFPENVPVVQAGWGRMSESGYDPSPVLLETQVYTINREYCQARYS
ELFEEDVPFITDNMICAGAPGGGRGACAQDEGGPLYYDNVLIGIISFNNECGNSTYPDVHTRVA
SYTNWIIETAVL
>OfuSPH10
MSCRLELFSLFAVLILNLVKADVCKDTKPDFSAPGQRISEQKCREYNWERKRLEKCSTDADSFE
FPYVGAVGYKEEESKDWVFACGTFLISERYTVTAAHCSRRRPDSRPEIVRFGNKNIVDDVKRLD
VKIRSVIVHPQYKAPIKNFDIALIQLESEVPFSRSIYPVCLWSKNLDSFKFAFNPAWGVEGKEYKI
RKEIAVMNVQILDHDSCVEATKPWITRLFRELSLHQICGDEQKSAVKNCQGSGGSPLITKGSLD
DSDEDQIPYAYGISSFGRGCGNMTIYTRISSFIDWIEETVWPKTTPANDAIHFVE
>OfuSPH11
MLIVQDASNVTNDQYPLVTIRPRQFYGKEVKIGELKHHVFIKKLVNKWKMMYRAICSGNIITQ
ERVLTSSHCFLTNRKKYRLSLKYLKVVGGTLSTYQQHPMHDSIQQWRSIKHVYSQKFYRFPAY
NLAVVVVEKPWDFNQYVNKIPYASKDHDFDGICMAIAVRSVKSWSTSKNLFSEEFRMIKRFDC
ERTLLRSCRLYFCTGTDTSKLLLDSIESEGGGLICHGTGDPQEVNEEQGLLVGVTSLIYTKLPTL
HHKVGLFHKWVTDEAHTNVEVHNVLMLFCLLIKLCIY
>OfuSPH12
MKQYILVLALAVSCQAFFKPGAQAPMEDEQVDEDYIFRDDEITEIAPIVPKSSTGQQKCGVRHN

KGEFRIGGTTKALFTEFNWVVPIMEVLWNGTEEYVCVGSLIHPEVVMTVAHYFTAKKNLIIKA
GDYDISHTKERHPHQVRDVSSVKLHPKFKLGTLKYDIALLFLSTPVNPAPHVGFACLPPALSMT
PADTACYVTGWGMETYDKNGEISHVLKKVEVPAVADSHCQAVFRDKIGPAFNLHKTWMCAG
GEEGRDACLRDGGGPLVCPYKNPKVENQYFQAGIVSWGNNCGLKGVPGVYSNVAALRQWID
DTMAAGGYTTEYYTP
>OfuSPH13
MGHMKVHRPMAKIRRDKIWWRWRCQWSIVTIAAVALVVYNAAANVWLLRPPACPAPATPPA
AEPPSCEPCLDTQNPALDDDPIARLDLRLGRWDGSRSYRMFDYATVGDMYAEVSMNRRVCLA
TQSSIERLHELLRIAAHWTGPISVAVFVAGDELRLLRAFATWLFRCQPDVYSRLALHVATSAERP
GVQGAVPNWARDCEAKPLPPGERRADTVAWRARHPYPQNHLRNLARRNCQTPYVFLVDVDIV
PSRGMSEALDKFLATAPRCPLCAYVVPTYELDRRVANFPANKSELLRLSKNKLAIPFHRKVFIYN
QYASNFSRWEASGGNESLETHVSHNVTNFELLYEPFYVAPDTVPPHDERFLGYGFTRNTQPRG
RRIPRIMMEARVPVVPHWLCQLSYGDALTTNMFCGGHFLIGGVSSCQGDSGGPAVFRGTAYGV
VSFARGCALPLSPTVFSNVAALRDWVTQTTGV
>OfuSPH14
KEGCGWRNTKGVGFMTTGDTDGEAKFGEFPWMVAILKIEPVNNNEPGGQQLNVYVGGGSLI
HMRVVLTTAHYVAKANKLRVRAGEWDTQTTKEIYPYQDRDVDSIEIHKDFNAGMLLKEGCG
WRNTKGVGFMTTGDTDGEAKFGEFPWMVAILKIEPVNNNEPGGQQLNVYVGGGSLIHMRVV
LTTAHYVAKANKLRVRAGEWDTQTTKEIYPYQDRDVDSIEIHKDFNAGNLFYDIALLFLNKPM
DPAPNIGVACLPPPKRLQTAGTRCFASGWGKDKFGKAGRYQVILKKVELPVVKHSTCQGLLRK
TRLGRLFELHRSFMCAGGEAGIDTCKGDGGSPLVCPMEVRQKKERKK
>OfuSPH15
MASAFVLALALFAAAASANPARIIGGSSTSIETWPSIVQVESTVGGIFWSHHCAGSILNSVSILSA
AHCFAGTGYDASNRRIRAGSSTLESGIIVYVAQENNHPSYGQNGNDGDITVVRLSSALIFSSTIA
PGSIVAAGTTIPGNLPVTKLGWGVTAVGGDRAEELQSIQVLTLDNSVCASNYATLSSSPQVTSN
MICAGSSTADACVGDGGGPVYFGNIIVGVISWGNGCLQDGFPGVNTAVSPYTPWIVATTSS
>OfuSPH16
MHVRYLFLICALAVNNVHCDCDQYIECFGDFLRDFFGNIHFVQTDKVNVTNSNKTLNYEFSEP
EDASIDKVLSDLTSLIKQIDGNIKESEVLIHDQNNTKIVVRTFELDDSVKNKEHIDSEDLILTTEIN
LAEDISFKDLTVTTPSESPTTEDAFKVIPLESKSEKDNEINDDIDQSYEEADEKKYVTDYPDIDYI
EIYKDTTNEHVSYQPTEANLFSESNELRSEINKQETEIENLLNHSLNLANEEALAEVVENVTNH
NIQTSTTEDEILKGMAPWIATIFLKNETGSQFDYYCDGALVSDRAVLTAARCVSNATGSYSPESF
IVILGKTSLRISGSQEKILRVQDVKTHVYFTIASSVAQNDLAVLTLEEPVLFNEGISKANLENMEI
NEEDGESSVSVTTAWGLSGDIALIYFDKEKSKACDSNNKVENTFCATYGDDVALCPSYGGLYV
TKQANKLYLAGIRTGDPTDRGICFIKNVNYTSLKNNTQWVSENIDV
>OfuSPH17
MFSLNCVILALVSFAIQTRGLVELPKVDDNNILVTEAAIRDTINTLFYKLGLRGIKTKPRLKEYST
VHNHLVSFTLQDYTNLKEIAKLTRVDKTKINNETRSFMTVINEILNEDNDDGRTEAIITEANDES
VIKYIDQFLTKLEDLKEQYGVINERFPIKEDLYKVLNDNEVLPYNFTMDQSYSSDVDENDTLID
GSNYWKPDMSGRRIYRGTRTKIKHFPFMASIQIFNRFHCGGSIIKSDLVITASSCLQLAWNNRFF
RENPAFLSVRVGSNFYNGGGENIPVLEVYFHPQYNPKNLWNNICILRLVRRLRFRKRGKKVKKI
DFDRNPANLPLTTDGITIVGWGAKGMSNVVKNPWENALSFSVLDIYPLRECQDIYSKEYVTKK
HFCAGFFSKGGGACNHDVGGPGVVKGFLMGVVSFGSPVCGTPDAPTVFTKLGYYADWIEEIM
EQEVPNINQRTTLRRKTIVTLGQYYQPIQPTTFKIPPLSGKIDPIPISEVDALRMLKSGKIFQEFLN

TMFGSSEAAQYQDLLTDVQPRNDQVMPAAESTTNSPPIPIQVPETTTPMQASIEDTAQVVTEIG
VVDPDFTTEAQLMKVTALDENFAISSRKDDDLEKNLVNLIDDIDLKEIIDGMPSVEEEKLLDDE
KIMDMLKDKDLEDKISKIDDSVLTLLYLSDAEKAERIGDSNNGEVNSASSAVDKSDDGLDNYY
GEAEERGLSIPTDNFQDLTYRNNNASVAVDQAISNDQLYYLISEINDNDAKNIKIKY
>OfuSPH18
MEKKLDKDPALKTSYEELTGEEKNPQVVATLKVIPQVTPDPKRFSDWTRLLRSTARIFQFIDLLR
ETFYDVRLNRRSLYTREWQYEGLPIEFPNRRSVQKVATYDETTTTTANPPIDPRYRRVYNPDEV
ASVVDHPFMAALLVNKQLWCGAVIIDRDTVLTAAHCLQLQYNNRFFREYVKMLSVRVGSTNA
TAGGEVLRVVEIFFHPNYKPQTLEFNFAVVKLHKNMTFGRHDLIADMVPYSKIKVVPVDNVITF
LGWGSVLVHGGNGGSVLLQKVELPVYDLADCQEVYGRNLVSRNNFCAGYITIPKNVCNHDA
GGPAIMDGQLVGILSFSSKRCDQPDHPAVFSTVGVIAPWLEKLGEKKVQLRNSAIKSPEAVHRQ
T
>OfuSPH19
MLNQSAIVPDADQKLTNINRRIYNSKRTKIKKYPFMASVHLSGDFVCAGSIISRDLIITSASCLQI
AYLHRHNQHFKSNVSVRIGSDFATHGGEYVAISQIYFHPLYNPENLKNNLAILGMNKHQHFSKK
RKIRRIVYDKTTGDLANNVNRVTIVGWGAKNELNLQDPSTKLSLGHLDLYDLGECKAVYSTFY
VNEQNFCAGFISKGSGACDKDVGDPAVVAGVLVGVVSFGPPLCGTPDAPTVFTRLGMYVNWI
DGIIRMVTISDPTKITKSKPILRMPIVRSSTQKRKGVYGLGRISFDYPEDFFILNKILTDISRPNAYE
VKDLINEGFNKEIFEDMVTPRMDMRHHPLANRSVFGMSGVNFPDQKYATKQKKIVLGTTPNV
LGINLAGPKIDFNDYVDTKKLGEMLEVLYHQHMSHLLPTSKHPVPVTTRHKWIGRNGDVDIN
RPMMTTPLFDEDDGDVGKGSDASSENYEETDSDEGMSIEVKRATIKPIYGTTQLVTDSGENNN
KDYDDKNVKTTGKLSPTKYDDYLNIFFALGKKAIEDSDRTNNEDEYSNPNNVVWISEDEFNFS
QDPESIDLNIANTFTRAR
>OfuSPH20
MSVRVRNLCIFALICISIHHVAGTTTTSTPSPPRYIAKPISIKRVPYMASLHECPRGACLASVIHKR
FLLTAASSLVLDWKGNLANLTKARVGSKYLDKGGQLYAIEKLIPHEKYLTFVGDYNIGLVKLA
MKIDFGSTVDRIKLGKKVIIGDLVDIIGWGVGENLQNSTKGAVHEVPSLPVLVPRRCQGALLGR
IIHPRNMCVEIAGVPPGDFVCDEGGPVVVNNSIVGVGSTFWGCGLLPGVYTRVSRVKRWIRQAI
DNNTYIDF
>OfuSPH21
MSVSSDIRFTRRKLSRPCRGCCAALASLLVLLLLAAVAVYLGHMYLFGDPLNRQTFRGSFVVGS
WGERPEGRDLHNDTREARLQRALVDLYQDSELRSCFVSAEILALDNVEEGERVHFEVSFEPIFT
AVTTGEVAAVLSRELAAPTHLQLVSLPETLHIEENSVISSLELENTETTLDDEPTTEAMTTEVEEI
SKCYPKTLSICSHLPYNTTTYPNLVGHTSKDAILRDLVAFRELFDAECSHLAQDFVCQMLQPRC
DLGRLVRPCRSYCRAFHAGCGARLPERLRAYFDCSHFPDYFGPGSCMPEPDCLGGLNRLALSR
RACDGIPDCADAADERGCAHCGAGGGLRCALHPHCLPSHLHCDGTPDCPDGSDEVGCLWITR
SLAAWRRERGEATLGAVRGRGGYAVWSERGRDGKICAAPYEADKRALKSVATSLCRAMSFSA
ALNVEVVSDAEELKSEHPEYVEVVDPFAPEISFLKSECPQRKVIKVICDQLECGIPSARVARARS
GVEGLPRSAQPGDWPWHAALLRANVHACDAALVHASWLLTTGSCFQGQPNVEWIARFGTVR
IQSTTPWQQERRVAEVIPSPVEGSMLALLRLEKPVEMTDFVRPVCLPENTTSADHAICNTLSWT
RNRDQLQRVQVITSPMNKCENVSIATVNGICSERLYDSDDCDEEEYAGSSMMCFDEKTKHWSL
VGVSGWRIACSKIGLGRPRIYDSIASHLDWIKQSISHYPR
>OfuSPH22
MNNSIVVIAIAIATLLSCAFAQNDRLCKLRKDPSVTYYCVEGDGSRVCEESVPSGTVVRPECSA

PVYYYSGTFPFMRCIDGSWDYIAKCLPECGTPSPMPIPLDSGSDSPDSTELPWHGAVYKKTTTP
YQFRCAVTLVTKKVVLSAAHCFWDGVKETALKAEDFAVAVGKTHRPWAAPEDQHAQKSDND
DDDDDLLCSALKNIFSQVAEIKLPARFRGGVTNYQDDLAVLVLAKEITFSPHVRPVCLDFNVIFE
RQQLKENSLGKVASWGGIKPSKTYKVTEMMYFPIEQCIAESPPDFREFLTSDKICTGFTNGTGL
CRGDGGAGLSFPSMERGRTRYYLRGLVSTAPTSSEDFLCTENSLQTYTQISKHELFIKENINTAK
>OfuSPH23
MRWFTVTCILLPLIIASTSAEWSWGQESKAEGKQENAQQTTDLLQGEELPKAEEKSSDSNSTVL
DDIVDELISSKQGRSLGGFDDDVYSDPTIKEALDAGDDAEARNLIKGRLCTLGLIQCDEDDTQE
KRTYLSPDELIYAQPVDIKPIGKPIASIPVRGPPRAYGPPRPMPTYHTRPQKVPPKRVGYGGNFRP
GFSEKYGVAGNNFQFSQSSSGGLFNGHEANYVTKPPTYANDSPYNFDNNKPSYNKGSSQSGTK
TDSVVQQHVHHHYVHSDGDKDPKVIIKPVAIPVGSVGHLASQSPQTFGHQSSDIITAGGGDYSS
LSSGGFKPMTGDFGGLNSKPVYESDTIYGSQYGHSGFNKEGSSILSQGLPNQYQNNLFEDQQK
YGNSLGSYGSQNSEFYKKELNVGSANNLYNQGGSFGANGAYQESYHVPKAQGLDCVCVNYD
QCPSQEVIGRRDDLYLPIDPRNKASEITALTDEQIDNITKLDAAEQANQNATTETKKVSKRETKE
ADDNKAEEASKQIEARLIGLAGYGGNGGNSNKQVQPTFGVSFGLPQPSHGGYPINPFNSNPLY
NPYGPALNSGGLNLGLVSVNPLLAVQVTKNDYGEKIVKPFVNLHVTPNEHVVNKLSNIFHEKK
LYLLNKHEHYHHFNPHHDHHFNPYHDHHYPVPHHPIQPIYSHNHHNPPPHYSHHGPPPVYSPQ
YNQYKPSYGHIHPYQSHEPPSYNDDYYDDNENVPNHFDDGADYNNVYQGGFYGPGFERNAN
ISANDRQGTYANRHGYSRSLSLPQNRPGANRGGQTIRFPENRKKRETTVEKSVENIQERQGYFG
RPPVQQCRQNQVCCRRPLKPQAGNRGQCGIRHSQGINGRIKTPSYVDGESEFGEYPWQAAILK
KDPKESVYVCGGTLIDANHIMTAAHCIKSYKGFELRIRLGEWDVNRDVEFFPYIERDVVSVHV
HPLYYAGTLDNDLAILKMDHPVEWTKYPHISPACLPDKYTDYAGQRCWTTGWGKDAFGDYG
KYQNVLKEVDVPILSHGQCQQQLKQTRLGYNYELNQGFLCAGGEEGKDACKGDGGGPLVCE
RGGTWQLVGVVSWGIGCGQPGVPGVYVKVAHYLDWISQITGKFSPY
>OfuSPH24
MKVVPFVLLFVHLQDVISQDQQPAKAFVQSYSKQLPKKNLQIVISRKFLNSKPLPQPKRPAPIN
KGYTAFPAIGSAEKKLYPVPESPYFPIDTNEQRVRVKKVLPDYVIGAVRRKRSSDLRPKVIELNL
KEDDNNNKTEVEPTKSAPKKKKRSKSAKKKRNAKARQSRNNTLTKTKSRKLKNQMNRSRNG
TKIRKKPKQLKYTKRSKRGHKNKTRSADKNITRSGDKKSVNKRHKPTSGDGHSKKAKEVGEA
RRLIAARDALIEDYPYVVSIQKNSEHWCAGALLNPRLVITTANCVWKSSRISRLRVRAGSRHTD
HGGQTAKIQEVMKHPQWSIRKNPDYDVALLLLDRNIKFSDSVHGVDLPNRVMLPAFEDAWVT
SWGAERRDGVVDKKDMSLQVFHTRLMDRGKCNNVTQRFGVTVTDNFICLSQMGRRAPCTR
DTGAPAVSDGVLWGLASWGIRRLCGTDRFPAMFSYLASQNNMDFIINATHILMADERYYPFPD
R
>OfuSPH25
MAAAYVFGFLLVFGLAGAFHPVLKNEQPAFMEDLRGVSSRIVAGWPAVDGQIPHQVSIRMVSA
AGAVFSCGGSVIHHNWVITAAHCVANRITFVIRFGLTNITQPQIILESTRKYIHPGYIEILAGVQPD
DIALVGIDQHIPYGPYIQPCRLQNSEDKNQDYTGHRLTVSGYGMTDDSWNGGTTSEILLWTFLR
GISNAECRSWYISGIVQDTTICAEFYNNTAQSSCQGKYQGRDKVHTGGYFMDREET
>OfuSPH26
MLFLPLLCFAFLQVQNATALFDFLLGRQKICVCRCGIQRPVTQSWLSRDSVSPNQYPWLAAVE
VGSRYISGVLLSDSHIVTAASPLYGVSSSEVTVTLGSRDRCGYGDDTSSHGADSILIHPSYSTTTS
DNDIALIKLRNKVSFTNLISPVCLPIIGSDRSGKVASVASWGNYSSGSSCLPKVASLPLLSSDTCL
TSSINVSMVTSDKGCLGPAGTTSVICSDDVGAPVMTQLLKNGAFRLVGIISSASCEQETTPLYTSI

SSHAAWIRQQIRSDCQCF
>OfuSPH27
MNRDDILKIIAERGQDKKIVKSARKAMGEDYFGSNKAEAILDDVVDKLLSETPRDKHKFNMN
ANDSKYWDPQGELEDLQEYHRHDGRRIFKGERTTVRYYPFMVSVHVMGRFWCGGVLYWHD
LVLTSAACLQLMHNNRFFRENPRVLQVRIGSNHSRIGGEMVDALEVQTGLFISATQNSTSILESA
KINDIAKALIKVLGVNPDVSNLTRIEELKEKLKKIDNYAIDKDKYALLLTNVTMETTLNETNFFK
SLYEDDSLKFLQNTESKNLFLKLKEKMNRDDILKIVAERGQDKKIVKSARKAMGEDYFGSNKA
EAILDDVVDKLLSETPRDKHKFNMNANDSKYWDPQGELEDLQEYHRHDGRRIFKGERTTVRY
YPFMVSVHVMGRFWCGGVLYWHDLVLTSAACLQLMHNNRFFRENPRVLQVRIGSNHSRIGGE
MVDALEVYFHPGYNPRTLRNNIAVIRLRRHLHFNYHRIPKIIAISDSPYTVAPTAEVLLLGWGVT
KMSQKMSYEPVYLQRKFLPVYPNTFCKEVYGDKFITQTMFCAGTFTTGEGACDHDAGGPAIL
AGKLVGIISFGPSVCGYPNAPTVFTLVGAFSDWIETVNETMPDYYRARARSTTTTRRPIFEYLGT
IFNTPPPTEAQGIEIHPLKLITLEATPPTVPTEVTTDYVEEETTEPPGLRFLKDQFSDADSWGDKK
>OfuSPH28
MLAKMYWLAVIAVCAYVAHAQTILPPNNTGLAPTPTTPRECGTGMVQGLDLGLQSDNGEAWL
GYGEFPWNVVIFKVAPASDDSNETFNVFLGGGSLIHMRVVLTAAHYVVTAEANTLRVRAGEW
DMGSTKELYPHQDRDVSSIEVHKDFIRGTLYYDAALLFLSSPVDPAPHINVVCLPPPKRLQVEG
TRCFASGYGKRYTNDTFQSLPKKVEVPVVRHSTCQKQLRSTRLGAIFELHRSFMCAGGEPGVD
TCKGDGGSALVCPMQYQPDRFVQAGIVAWGVGCGGGVPGVYADTSVIRDFIDDKMAAVGLG
DEMVYARY
>OfuSPH29
MGNQARSGATAFCTPRGGSVKDSALCFAASNCLLRARSSELSVRLGSSWKTHGGEMYDVKEC
FVHPRYISKTKINDVGLVRLYSPLRFSEKVLPIKLVAREARLLADVPAIVSGWGKLKEGGPSATY
LQSSTIKTVAMKLCKHSGLDRKAIDPPSMFCAGSFTQPSPDACQGDSGGPIVSDGVLIGVVSWG
LGCARGNFPGVYTRLSHHIIWDWVHQHITRKPDT
>OfuSPH30
MTIVTDGLGSGRRSHGPKNALRIRNDYGMMVDDRGNLRIRGGNVTDTKNFPYMAAIIINGRL
WCAGAIVDENWVITAAHCLNYVLHVSPLKSLGQYVKVRVGSSRPHEGGTLVDVVGAVRHPKF
EEEPVPHADVALLKLAENLEFHDQVNLIKIYEGVKEPYAQSFVVVSGWGATRGSDTAFRDHTP
DLLTARLKVRTQHYCVDAYQLVNGFQFTQDFFCASLRNGTRDACLFDAGAPAVQQNRLMGV
MSFGPERCGHEYQPAVFIKAFYFRDFVKSTIASYKSTADLIEAMQEADPVVTSHHLQPVHAGH
DGYDGRPEDVTLPDDRHD
>OfuSPH31
MRTFIILLLGLAAVSAYPKKIQRIVGGSVTSINQYPEMASLLFSQGTGSLYRQACGGTILSFRSILT
AALCTIGHAPIRWRARVGSTFANSGGFVLDTQTITNHPNYNGWILDNNLSIIRTFRDIPIWNNST
VQFANIAGANYQLADDQVVWATGWGATSQGGSSSEELRHVQIWTINQAICRQRYATTGDTITD
NMLCAGWLDVGGRGQCQGDTGGPLFHNGVVVGVGSWGRGCGQPFFPGVYTRVSRYTNWLS
WA
>OfuSPH32
MERARPATWPARAYVQFATPSISEQRVTTLVIKYVGPVAVPANDTCKDCLASLFEKRFFPKNGDI
PMLNRKVFRATLVPIAEHPYVVSVRRQYAHYLTGTVLTKNTILSVAHPLYRVGTYELAVVVGES
YADRGTSLHSVILIIIHEDFDRFTLKADIALLRLYEELSYRVSIKPISLSSSFSPKYGRTAFVTGWG
RCDRMGKELCLPRSSRFFRWEKLDPMLRTIKFTMTHSEHCNEYTMHELKMDDRMLCAGPARV
DEILCPCMAVPGAPLVVDAKLVGIQSWGFSCGYLRDMPVVYTRLEAYHGWLSHNIPHLRRIHK

ENLTQLFDATKVKRLTDWLVKSRVRMPLLRKIHNHDLQILPIDHQLTLLRGVVYDIRDFLRRGA
YHEAKTALYNSIRLSTEKVKAMRDNTMMKSNVEVVPFLSNSTLDDIGLVLRNGSLIDEASGEE
FDEVEEASITAKFSSSRNISLIDEDSSDESVEVDGTDSTPSLSTIAYDSDESDEN
>OfuSPH33
MFPFVENITSNYYNCEEKKTSSSSEEDRCKSTLVNYPYSVSIQKKGAHYASGALINAQSVLTTA
GVFYHVREAIKLFRVRLGSVDCKKGGIIVPIKRVEIHPSYEFGKPSFDLALIKLARPVNNTDYIKP
IQLSRIKQGVVSAKFMTTYWPRLIVNGNVLPHTAKERVKYQSMRVSKQKLIPWKTCAAMMRA
RSAVLDETSLCLKPIATHHSVCMPDVGAPVVAEDGLWGITSGWTSKQCSIYPSPTILTRTAMPPV
RTWLESVL
>OfuSPH34
MEEDLTHVRRKRLTTKNGLVCLEDYPYTVSIRLHGKHWCGGAIVSKLWILSAAQCFDYVTKEE
VSVRVGSVFRDFGGRILAVTDIRRHPEYKMDQYYPEHNLAMIKINLPITPSTRMQPVGLAIADA
DLPPMFGETVVTGYGSVVTGQIREGENQELRRMIAREVPRAECRLVYGNDYSLQHDHMCLQS
VVKGVALCAGDTGDPAVHFSGLNRAGTLYGIALFSGTEECAYKSKPGIYAKVSYSRQWIDKLIG
ETPDRTESADSGENAIPLDME

Reference.

1. Shen, D., Liu, Y., Zhou, F., Wang, G. & An, C. Identification of immunity-related genes in *Ostrinia furnacalis* against entomopathogenic fungi by RNA-seq analysis. *Plos One* **9** (2014).
2. Chu, Y., Hong, F., Liu, Q. & An, C. Serine protease SP105 activates prophenoloxidase in Asian corn borer melanization, and is regulated by serpin-3. *Sci. Rep.* **7** (2017).
3. Ross, J., Jiang, H., Kanost, M. R. & Wang, Y. Serine proteases and their homologs in the *Drosophila melanogaster* genome: an initial analysis of sequence conservation and phylogenetic relationships. *Gene* **304**, 117-131 (2003).
4. Perona, J. J. & Craik, C. S. Structural basis of substrate-specificity in the serine proteases. *Protein Sci.* **4**, 337-360 (1995).
5. Feng, C. *et al.* Clip domain prophenoloxidase activating protease is required for *Ostrinia furnacalis* Guenee to defend against bacterial infection. *Dev. Comp. Immunol.* **87**, 204-215 (2018).
6. Benjamini, Y. & Hochberg, Y. On the adaptive control of the false discovery fate in multiple testing with independent statistics. *J. Edu. Behav. Stati.* **25**, 60-83, (2000).
7. Reiner, A., Yekutieli, D. & Benjamini, Y. Identifying differentially expressed genes using false discovery rate controlling procedures. *Bioinformatics* **19**, 368-375, (2003).
